# SIRPγ promotes CD8⁺ T cell exhaustion via metabolic-epigenetic reprogramming in tuberculous pleural effusion

**DOI:** 10.1038/s41467-026-77779-4

**Published:** 2026-09-25

**Authors:** Yuening Yang, Qianqian Xue, Sidao Wang, Wenbei Peng, Linlin Ye, Bohan Yang, Zihao Wang, Xuan Xiang, Haolei Wang, Yiran Niu, Hai Huang, Chengqing Yang, Qiong Zhou

**Affiliations:** 1 Department of Respiratory and Critical Care Medicine, Union Hospital, Tongji Medical College, Huazhong University of Science and Technology, Wuhan, China; 2 Department of Respiratory and Critical Care Medicine, The Central Hospital of Wuhan, Tongji Medical College, Huazhong University of Science and Technology, Wuhan, China; 3 Wuhan Pulmonary Hospital, Wuhan Institute for Tuberculosis Control, Wuhan, Hubei China

**Keywords:** Infection, Infectious-disease diagnostics, CD8-positive T cells, Tuberculosis

## Abstract

Previous studies have shown that signal regulatory protein γ (SIRPγ) is an important regulatory factor for T cell function. However, its expression and biological role in tuberculous pleural effusion (TPE) remain unknown. In this study, we find that the level of SIRPγ is significantly upregulated in TPE compared to non-TPE. Furthermore, we demonstrate SIRPγ involvement in TPE pathogenesis through the induction of CD8⁺ T cell exhaustion. Mechanistically, SIRPγ interacts with CD47 to activate the phosphatidylinositol 3/kinase-protein kinase B/mammalian target of rapamycin (PI3K/AKT/mTOR) pathway, leading to the upregulation of phosphoglycerate dehydrogenase (PHGDH) and the enhancement of serine metabolism. Serine, in turn, facilitates S-adenosylmethionine (SAM)-dependent trimethylation of histone H3 lysine 4 (H3K4me3) epigenetic modification at the NFATC2 locus. This modification, in coordination with TCR-induced calcium signaling, promotes NFATC2 activation, ultimately driving the expression of exhaustion-associated transcription factors and immunosuppressive receptors. Together, our findings identify SIRPγ as a regulator of CD8⁺ T cell exhaustion in TPE and suggest its potential as a predictive biomarker for TPE diagnosis.

## Abstract (web summary)

Tuberculosis pleural effusion (TPE) is the most common manifestation of extrapulmonary tuberculosis. Here the authors characterise the involvement of signal regulatory protein γ (SIRPγ) and show that this protein is associated with TPE and involved with pathogenesis via promotion of changes of T cell metabolic pathways and TCR signaling associated with exhaustion of T cells.

## Introduction

Tuberculous pleural effusion (TPE), the most common manifestation of extrapulmonary tuberculosis, accounts for up to 30% of all tuberculosis (TB) cases in high-prevalence region^1^. This high prevalence underscores the urgent clinical need for rapid and accurate diagnostic methods. However, TPE is difficult to diagnose. Conventional microbiological techniques, such as smear microscopy and culture, are poorly sensitive to *Mycobacterium tuberculosis* (Mtb) in pleural fluid, due to the low burden of Mtb in pleural fluid. As a result, clinicians frequently depend on invasive procedures to obtain pathological or microbiological confirmation, which are time-consuming and carry certain risks^2^. These challenges have sparked growing interest in non-invasive biomarkers for identifying TPE^3^. Although biomarkers such as adenosine deaminase (ADA) and interferon (IFN)-γ have demonstrated high diagnostic accuracy for TPE, they serve primarily as diagnostic indicators without providing mechanistic insights into T cell dysfunction, a key feature of the local immune response^4^. These limitations have driven the search for novel biomarkers that not only aid in diagnosis but also provide insights into the underlying immune mechanisms of TPE.

In recent years, biomarkers derived from immune cells have been increasingly utilized as a valuable tool in diagnostics^5,6^. TB is a chronic inflammatory disease driven by complex interactions among diverse immune cells, cytokines, and Mtb. Although CD4⁺ T cells are widely recognized as playing a dominant role in controlling Mtb infection, accumulating evidence underscores the critical role of CD8⁺ T cells in host defense^7,8^. Studies in non-human primate infection models have demonstrated that CD8⁺ T cells are essential for controlling early-stage TB and maintaining the containment of latent infection^9^. CD8⁺ T cells contribute to antimicrobial activity through cytolytic mechanisms and the production of IFN-γ^10^. However, in the context of TPE, CD8⁺ T cell responses are often overlooked due to their functionally exhausted state^11–16^. These exhausted CD8⁺ T cells exhibit impaired cytotoxic function and proliferative capacity, which may compromise local anti-mycobacterial immunity and thereby contribute to disease persistence. Therefore, identifying this distinct population of exhausted CD8⁺ T cells in TPE and elucidating the mechanisms underlying their dysfunction are essential for discovering novel diagnostic biomarkers and developing targeted immunotherapeutic strategies for TPE.

Signal regulatory proteins (SIRPs) are a family of transmembrane glycoproteins associated with immune inhibition, comprising three main members: SIRPα, SIRPβ, and SIRPγ^17^. Among them, SIRPγ (also known as CD172g or SIRPβ2) is specifically expressed in primate T lymphocytes and activated natural killer (NK) cells. Under chronic stimulation, it regulates T cell activation through interacting with CD47^18,19^. Furthermore, abnormal expression of SIRPγ has been reported in several immune-related disorders, including systemic lupus erythematosus (SLE) and type 1 diabetes (T1D), with its expression negatively correlated with levels of IFN-γ and tumor necrosis factor-α (TNF)^20,21^. These findings suggest that SIRPγ plays a regulatory role in adaptive immune responses and may serve as a promising target for immunotherapy. However, its role in CD8⁺ T cell function in TPE remains unexplored.

In this study, we demonstrate that the level of SIRPγ is significantly upregulated in TPE compared to non-TPE. Moreover, SIRPγ serves as a regulator of CD8⁺ T cell exhaustion. Mechanistically, SIRPγ interacts with CD47 to activate the phosphatidylinositol 3/kinase-protein kinase B/mammalian target of rapamycin (PI3K/AKT/mTOR) signaling pathway, leading to the upregulation of phosphoglycerate dehydrogenase (PHGDH) and the enhancement of serine metabolism. In turn, elevated serine levels promote S-adenosylmethionine (SAM)-dependent H3K4me3 epigenetic modification at the NFATC2 locus, which, in coordination with TCR-induced calcium signaling, drives NFATC2 activation. Ultimately, this signaling cascade induces the expression of exhaustion-associated transcription factors and immunosuppressive receptors.

## Results

### SIRPγ serves as a diagnostic biomarker for TPE

**Fig. 1:**
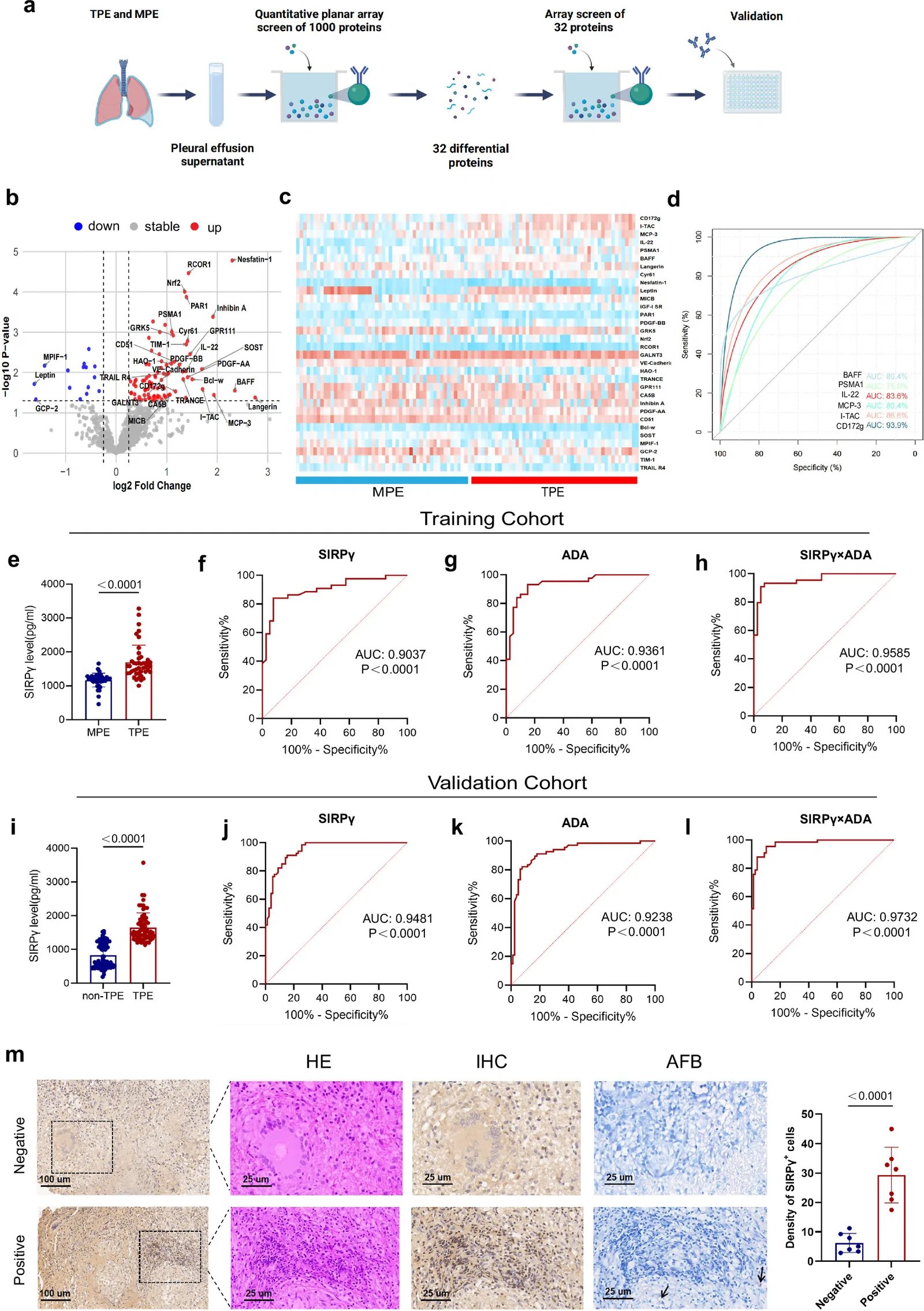
SIRPγ serves as a diagnostic biomarker for TPE. **a** Schematic workflow for screening differentially expressed proteins between MPE and TPE using a high-throughput cytokine array. It was created in BioRender. Yang, Y. (2026) https://BioRender.com/036n0bt. **b** Volcano plot identifying 102 differentially expressed proteins from the initial high-throughput cytokine array screening (8 MPE vs. 8 TPE). **c** Heatmap depicting expression profiles of 32 proteins in 100 pleural effusion samples (50 MPE vs. 50 TPE) from the secondary antibody microarray validation. **d** The ROC curves of CD172g (SIRPγ), I-TAC, MCP-3, IL-22, PSMA1, BAFF. **e** Comparative analysis of SIRPγ levels in TPE (*n* = 44 patients) versus MPE (*n* = 40 patients) from training cohort. **f–h** receiver operating characteristic (ROC) curve analysis evaluating the diagnostic value of SIRPγ (**f**), adenosine deaminase (ADA) (**g**), and the combined SIRPγ × ADA index (**h**) in the training cohort. **i** Comparative analysis of SIRPγ levels in TPE (*n* = 66 patients) versus non-TPE, including MPE (*n* = 35 patients), parapneumonic pleural effusion (PPE) (*n* = 27 patients), and miscellaneous (*n* = 16 patients) from the validation cohort. **j**–**l** ROC curve analysis evaluating the diagnostic value of SIRPγ (**j**), ADA (**k**), and the combined SIRPγ × ADA index (**l**) in the validation cohort. **m** Representative hematoxylin and eosin (H&E), immunohistochemistry (IHC) detection of SIRPγ, and acid-fast bacteria stain (AFB) stains in pleural tissues are displayed with corresponding quantitative results of SIRPγ^+^ cell density (*n* = 7 patients). Statistical analyses were performed using unpaired two-tailed *t* tests (**e**, **i**, **m**). Data are presented as mean values ± SD (**e**, **i**, **m**).

To characterize the regulatory protein profile within the TPE immune microenvironment, the high-throughput cytokine array was employed to identify differentially expressed proteins between TPE and MPE (Fig. 1a). In the initial screening, 1000 proteins were profiled across specimens from 8 TPE and 8 MPE cases. This analysis generated a heatmap illustrating the expression differences among all analytes (Supplementary Fig. S1a). A subsequent volcano plot underscored notable differences in protein expression (Fig. 1b). This initial screening identified 102 proteins that met the significance threshold (*p* ≤ 0.05). To further explore key differentially expressed proteins between TPE and MPE microenvironments, we selected 32 proteins based on more stringent criteria (p ≤ 0.05 and FC > 2) for validation in an expanded cohort (50 TPE and 50 MPE samples) using a high-throughput cytokine array. This validation confirmed 17 proteins with significantly altered expression between TPE and MPE (Fig. 1c and Supplementary Fig. S1b). Of particular interest, SIRPγ (CD172g) levels were markedly elevated in TPE (*p* < 0.0001; AUC = 0.939), highlighting its strong diagnostic potential for distinguishing TPE (Fig. 1d). These findings were further validated using ELISA. Compared to MPE, the training cohort confirmed that SIRPγ levels were markedly higher in TPE (Fig. 1e). As expected, ADA, a well-established biomarker for TPE, was detected at markedly higher levels in TPE than in MPE (Supplementary Fig. S1c). The ability of SIRPγ, ADA, and SIRPγ × ADA to discriminate between TPE and MPE was evaluated through ROC curve analysis (Supplementary Table S1). At a cutoff value of 1324 pg/mL, SIRPγ demonstrated strong diagnostic performance, with an area under the curve (AUC) of 0.9037 (95% CI: 0.8375–0.9698; *p* < 0.0001), yielding a sensitivity of 84.09% and specificity of 90.00% (Fig. 1f). Similarly, ADA exhibited robust discriminative capability, achieving an AUC of 0.9361 (95% CI: 0.8840–0.9882; *p* < 0.0001) at a threshold of 24.2 U/L, with sensitivity and specificity of 86.36% and 87.50%, respectively (Fig. 1g). To assess whether combining SIRPγ and ADA could enhance diagnostic accuracy, we analyzed the product of their concentrations (SIRPγ × ADA). This combination significantly enhanced both sensitivity and specificity compared to ADA alone. Using a cutoff value of 39,414 ng·U/L², the SIRPγ × ADA product achieved an AUC of 0.9585 (95% CI: 0.9190–0.9980; *p* < 0.0001), with 93.18% sensitivity and 92.50% specificity (Fig. 1h). To further evaluate the diagnostic specificity of SIRPγ, we expanded the validation cohort to include TPE, MPE, parapneumonic pleural effusion (PPE) and miscellaneous samples. The analysis revealed that SIRPγ, ADA, and SIRPγ × ADA consistently maintained strong diagnostic performance (Supplementary Table S2). SIRPγ achieved an AUC of 0.9481 (95% CI: 0.9162–0.9801; *p* < 0.0001), with a sensitivity of 82.09% and specificity of 91.03% (Fig. 1i, j). ADA produced an AUC of 0.9238 (95% CI: 0.8778–0.9699; *p* < 0.001) (Fig. 1k and Supplementary Fig. S1d). Notably, the SIRPγ × ADA product yielded a significantly higher than ADA alone (0.9732; 95% CI: 0.9510 - 0.9954; *p* < 0.0001) (Fig. 1l). Interestingly, a positive correlation was observed between SIRPγ levels and lactate dehydrogenase (LDH) expression, suggesting a potential association between SIRPγ and the degree of inflammation of TPE (Supplementary Fig. S1e).

To further evaluate the expression pattern of SIRPγ in pleural tissues from TPE patients, histological and immunohistochemical analyses were performed. These analyses revealed that tuberculous lesions exhibited higher SIRPγ expression compared to adjacent non-lesional areas, with predominant localization at the periphery rather than the core of the lesions (Supplementary Fig. S2a). Notably, among pleural specimens from patients clinically diagnosed with TPE, those positive for acid-fast bacilli staining (AFB) showed elevated SIRPγ expression, suggesting a potential correlation between SIRPγ levels and microbiological confirmation (Fig. 1m). Collectively, these findings support the potential utility of SIRPγ as a diagnostic biomarker for TPE.

### SIRPγ is highly expressed in exhausted CD8^+^ T cells in TPE

**Fig. 2:**
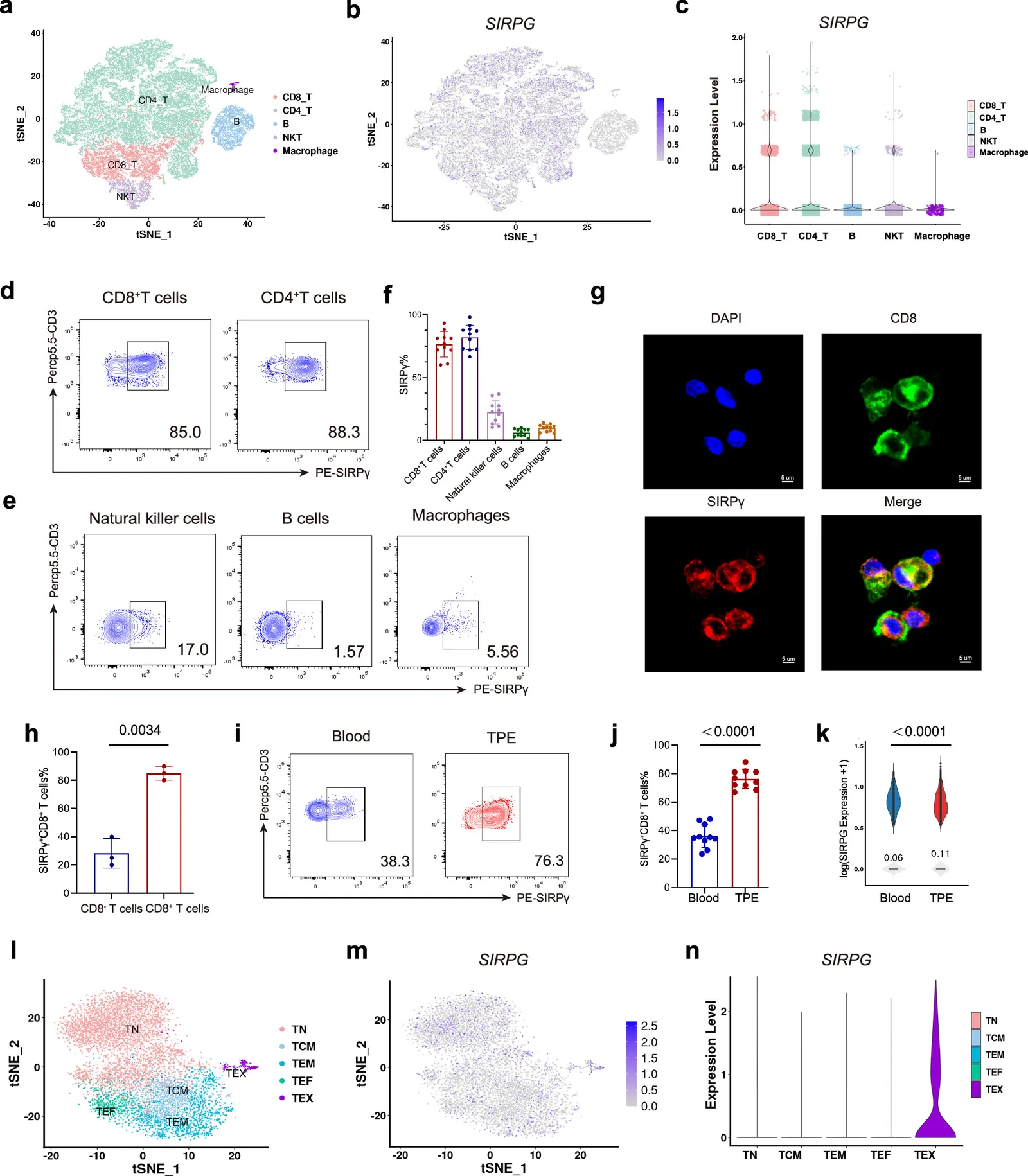
SIRPγ is highly expressed in exhausted CD8^+^ T cells in TPE. **a** The t-distributed stochastic neighbor embedding (t-SNE) map identified five distinct immune cell subsets based on single-cell RNA sequencing, including B, NK, Macrophage, CD4_T, and CD8_T. **b** Expression levels of the *SIRPG* gene visualized within the t-SNE space. (c) *SIRPG* gene expression across distinct cell subsets. **d–f** Representative flow cytometric plots and corresponding statistical analyses showing the proportions of SIRPγ^+^ cells from TPE, including SIRPγ^+^ B cells (CD45^+^ CD19^+^ SIRPγ^+^), SIRPγ^+^ natural killer cells (CD45^+^ CD56^+^ CD3 SIRPγ^+^), SIRPγ^+^ macrophages (CD45^+^ CD68^+^ SIRPγ^+^), SIRPγ^+^ CD4^+^ T cells (CD3^+^ CD4^+^ SIRPγ^+^), and SIRPγ^+^ CD8^+^ T cells (CD3^+^ CD8^+^ SIRPγ^+^) (*n* = 11 patients). **g**, **h** Representative images demonstrating the colocalization of SIRPγ and CD8 in CD8^+^ T cells from TPE (**g**) alongside the corresponding statistical analysis (**h**) (*n* = 3 biologically independent samples). Scale bar 5 µm. **i**, **j** The proportion of SIRPγ⁺CD8⁺ T cells were determined by flow cytometric analysis in paired clinical samples collected from TPE and peripheral blood. (**i**) presents representative flow plots, while (**j**) shows the corresponding statistical analysis (*n* = 10 patients). **k** Expression levels of *SIRPG* in CD8⁺ T cells derived from TPE and peripheral blood were assessed through single-cell RNA sequencing analysis. **l** Transcriptional classification of CD8⁺ T cell clusters into five functional subsets, including naïve-like T cells (T_N_), central memory-like T cells (T_CM_), effector memory-like T cells (T_EM_), effector memory-like T cells (T_EFF_), exhausted T cells (T_EX_) populations. **m** *SIRPG* gene expression visualized within the t-SNE space. **n** Violin plots illustrating *SIRPG* expression across five cell populations, including T_N_, T_CM_, T_EM_, T_EFF_, and T_EX_. Statistical analyses were performed using paired two-tailed*t* tests (**h**, **j**). Data are presented as mean values ± SD (**f**, **h**, **j**).

SIRPγ is primarily expressed on T cells, certain B cell subsets, and activated NK cells, and has been reported to exert both stimulatory and inhibitory effects on these lymphocyte populations^22^. However, its functional significance and signaling mechanisms in human TB remain poorly characterized. To investigate the distribution of SIRPγ among immune cell subsets in human TB, previously published sc-RNA seq data from TB-infected human lungs were analyzed^23^. The result revealed that *SIRPG* (encoding SIRPγ) was primarily expressed in T cells (Supplementary Fig. S3a–d). Immunofluorescence staining further demonstrated that SIRPγ was highly expressed on T cells, whereas macrophages showed little to no expression of this protein (Supplementary Fig. S4a, b). Consistently, scRNA-seq data^24^ specific to human TPE confirmed tha*t SIRPG* transcription was primarily restricted to T cells in both PE and peripheral blood (PB) (Fig. 2a–c and Supplementary Fig. S5a–c). These multi-omics observations were subsequently validated through flow cytometry and immunofluorescence (Fig. 2d–h and Supplementary Fig. S5d, e). A similar expression pattern was also observed in MPE (Supplementary Fig. S5f). Comparative analysis of immune cell subsets between TPE and MPE revealed higher percentages of SIRPγ⁺ CD8⁺ T cells, CD4⁺ T cells, and NK cells in TPE (Supplementary Fig. S5g), reinforcing the potential of SIRPγ as a biomarker for TPE. Furthermore, significantly elevated expression of SIRPγ was further observed on CD8⁺ T cells in TPE compared to PB (Fig. 2i–k). In contrast, SIRPγ levels on CD4⁺ T cells remained comparable between TPE and PB (Supplementary Fig. S5h–j). These findings imply a potential CD8⁺ T cell-specific role for SIRPγ within the TPE microenvironment, warranting further investigation.

To further characterize CD8⁺ T‑cell heterogeneity in TPE, CD8⁺ T cells were grouped into 12 clusters through unsupervised clustering and dimensionality reduction, representing five phenotypically distinct subsets (Fig. 2l and Supplementary Fig. S5k, l). Cluster 9 constituted the exhausted T cells (T_EX_) population, exhibiting elevated expression of exhaustion markers including *LAG3*, *HAVCR2*, *TIGIT*, *CTLA4*, *CXCL13*, and *TOX*. Clusters 3 and 8, designated effector memory-like T cells (T_EM_), highly expressed characteristic effector memory genes (*CCL5*, *CD69*, *HLA-DQA1*, *DUSP2*). Clusters 1 and 10 comprised central memory-like T cells (T_CM_), defined by *GZMK*, *CD44*, *EOMES*, *RPS26*, and *CLDND1* expression. Clusters 0, 2, 4, 5, 6, 11, and 12 represented naïve-like T cells (T_N_), characterized by *CCR7*, *SELL*, *TCF7*, *LEF1*, and *MALL*. Cluster 7 was identified as effector T cells (T_EFF_), demonstrating expression of effector-function genes (*KLRG1*, *PRF1*, *GZMB*, *IFNG*, *GNLY*, *NKG7*). Notably, *SIRPG* expression was significantly enriched in the T_EX_ subset, correlating with exhaustion status (Fig. 2l–n). To elucidate the dynamic transcriptional landscape of CD8⁺ T cells in TPE, we performed trajectory analysis. Unsupervised pseudotime analysis using Monocle2 revealed that T_N_ occupied the earliest position along the differentiation trajectory, whereas T_EX_ emerged at the terminal stage (Supplementary Fig. S6a, b). This continuum reflects the dynamic adaptation of CD8⁺ T cells to the persistent inflammatory milieu characteristic of TPE. During this differentiation process, *SIRPG* expression was predominantly observed in the T_EX_ subset rather than the memory T cell (T_M_) and T_N_ population. Moreover, *SIRPG* expression increased markedly along the trajectory toward T_EX_ differentiation, accompanied by the upregulation of exhaustion markers such as *HAVCR2* and *CTLA4* (Supplementary Fig. S6c).

### SIRPγ serves as a potential mediator of T cell exhaustion in TPE

Recent studies have indicated the presence of partial T-cell exhaustion during Mtb infection, characterized by increased expression of multiple inhibitory receptors and impaired cytokine production^11,25,26^. The molecular mechanisms driving CD8⁺ T cell exhaustion remain poorly understood. To address this, freshly isolated peripheral blood mononuclear cells (PBMCs) from purified protein derivative-positive (PPD + ) donors were stimulated with the recombinant fusion antigen E-C, which comprises early secretory antigenic target‑6 (ESAT‑6) and culture filtrate protein‑10 (CFP‑10). Following immediate stimulation, reduced secretion of interleukin (IL)-2, TNF, Granzyme B, and IFN-γ was observed in CD8⁺ T cells, along with significantly impaired proliferative capacity and elevated expression of inhibitory receptors, including T cell immunoglobulin and mucin domain-3 (TIM-3), cytotoxic T-lymphocyte-associated antigen-4 (CTLA-4), T cell immunoglobulin and ITIM domain (TIGIT), and programmed death-1 (PD-1) (Supplementary Fig.S7a–e). Consistent with a previous finding^25^, these results suggest that immediate stimulation by TB antigens induces exhaustion-associated features in CD8⁺ T cells, impairing T-cell-mediated control of Mtb infection. Importantly, we observed elevated SIRPγ expression on CD8⁺ T cells following TB-specific antigen stimulation (Supplementary Fig. S7f), implying a potential role for TB antigens in promoting CD8⁺ T-cell exhaustion via SIRPγ.

**Fig. 3:**
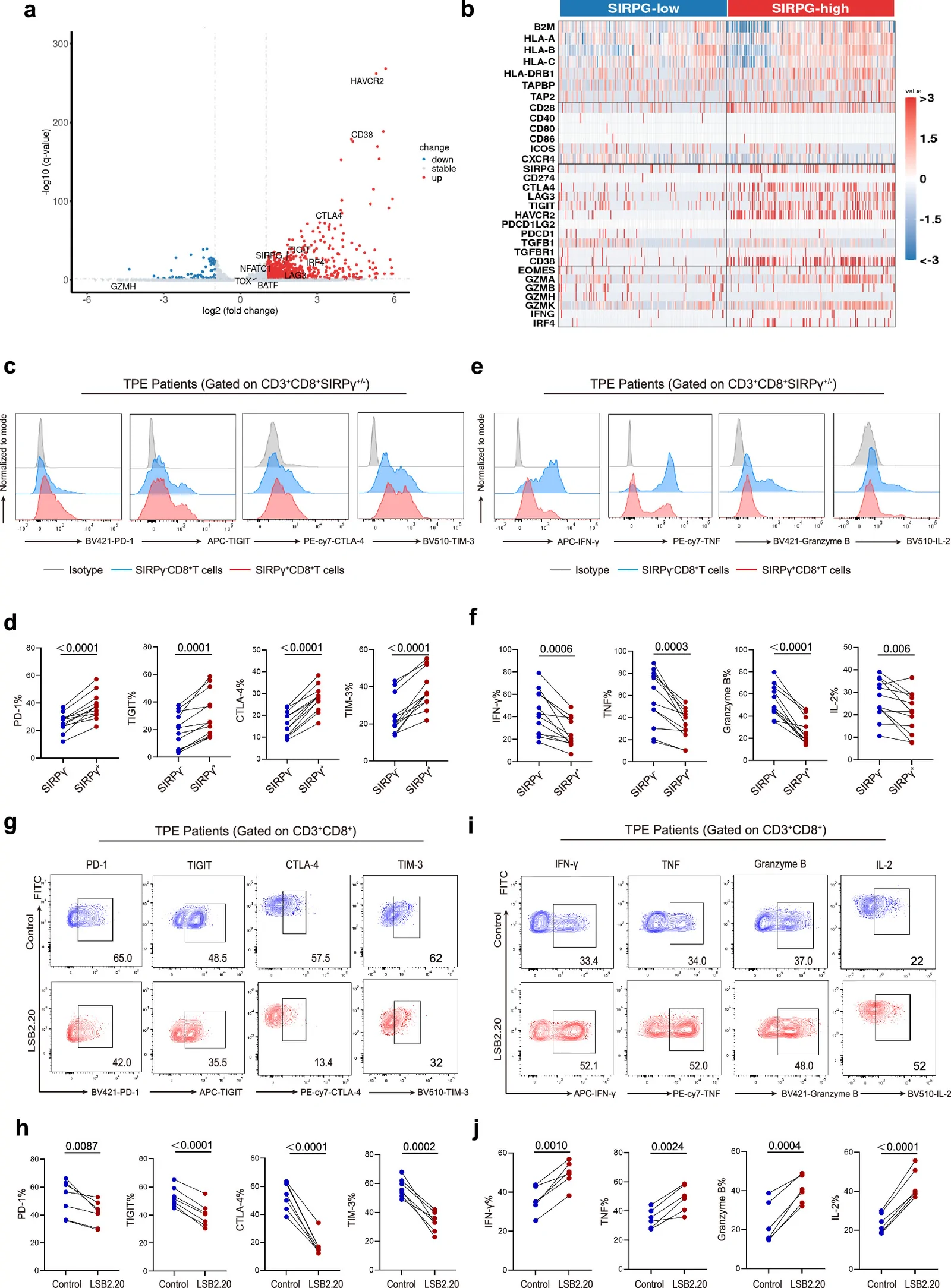
SIRPγ serves as a potential mediator of T cell exhaustion in TPE. **a**, **b** Differentially expressed genes (DEGs) between SIRPγ⁺ and SIRPγ⁻ CD8⁺ T cells are shown in a volcano plot (**a**) and a heatmap (**b**), with the latter highlighting representative genes involved in MHC presentation, immune stimulation, immune suppression, and cytotoxicity. **c**, **d** CD8⁺ T cells isolated from TPE were analyzed to quantify inhibitory receptor expression (TIGIT, TIM-3, CTLA-4, PD-1) on SIRPγ⁺ and SIRPγ⁻ subsets via flow cytometry (*n* = 12 patients). **e**, **f** Cytokine production (IL-2, IFN-γ, Granzyme B, TNF) in SIRPγ⁺ and SIRPγ⁻ CD8⁺ T cells isolated from TPE was assessed by flow cytometry (*n* = 12 patients). **g**–**j** CD8⁺ T cells isolated from TPE were activated with anti-CD3/CD28 antibodies for 24 h, followed by treatment with the anti-SIRPγ monoclonal antibody LSB2.20 (10 μg/mL) for 48 h. Expression of inhibitory receptors (**g**, **h**) (*n* = 7 patients) and cytokine production (**i**, **j**) were then analyzed by flow cytometry (*n* = 6 patients). Statistical significance was determined by paired two-tailed *t* tests (**d**, **f**, **h**, **j**).

We next investigated the association between *SIRPG* and T cell exhaustion. Given the low overall expression of *SIRPG* in sequencing data, CD8⁺ T cells were stratified into *SIRPG* ^low^ and *SIRPG* ^high^ subsets for comparative analysis. DEGs identified by scRNA-seq were categorized into three functional groups: MHC molecules, immunostimulatory molecules, and immunosuppressive/cytotoxic molecules. *SIRPG*^high^ CD8⁺ T cells displayed increased expression of exhaustion markers (*TIGIT*, *HAVCR2*, *CTLA4*, *LAG3*) and exhaustion-associated transcription factors (*TOX*, *NFATC1*, *NFATC2*, *BATF*) (Fig. 3a, b, Supplementary Fig. S8a, b). Flow cytometry confirmed that SIRPγ⁺ CD8⁺ T cells from TPE expressed higher inhibitory receptor levels (CTLA-4, TIM-3, PD-1, TIGIT) and demonstrated reduced production of IFN-γ, TNF, Granzyme B, and IL-2 compared to SIRPγ⁻ CD8⁺ T cells in both PB and PE from TPE patients (Fig. 3c–f and Supplementary Fig. S8c–f). A comparable expression profile was detected in PB from patients with pulmonary TB (Supplementary Fig. S8g–j). To examine the association between SIRPγ and CD8⁺ T cell exhaustion, SIRPγ was selectively blocked using the monoclonal antibody LSB2.20 in CD8⁺ T cells isolated from TPE (Supplementary Fig. S9a). SIRPγ blockade significantly attenuated the exhaustion phenotype in CD8⁺ T cells, as evidenced by reduced expression of inhibitory receptors (CTLA-4, TIM-3, PD-1, TIGIT), increased production of cytokines (IFN-γ, IL-2, TNF, Granzyme B), and enhanced proliferative capacity (Fig. 3g–j and Supplementary Fig. S9b). Furthermore, this intervention did not significantly change the proportions of naive (CD45RA⁺CD8⁺) or memory-like (CD45RA⁻CD8⁺) T cell subsets (Supplementary Fig. S9c, d). Collectively, these findings suggest SIRPγ promotes CD8⁺ T-cell exhaustion in TPE.

### SIRPγ drives serine metabolism reprogramming in CD8^+^T cells

**Fig. 4:**
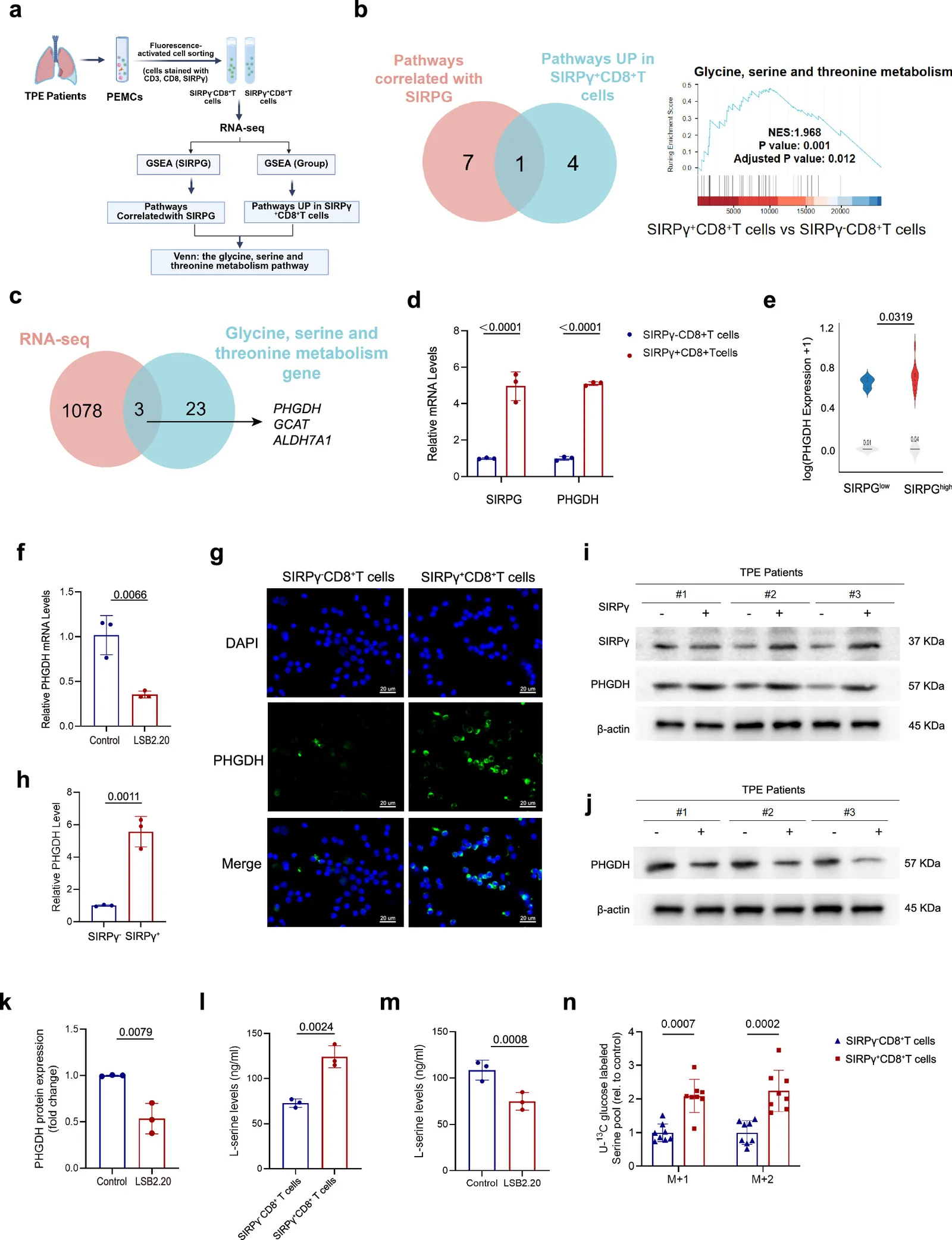
SIRPγ promotes CD8^+^ T cell exhaustion by reprogramming serine metabolism in TPE. **a** A schematic diagram of RNA-seq analysis was created in BioRender. Yang, Y. (2026) https://BioRender.com/77uhcxh. **b** Venn diagram highlighting overlapping metabolic pathways from differential gene KEGG analysis (SIRPγ⁻ vs. SIRPγ⁺ CD8⁺ T cells) and Single-gene KEGG targeting *SIRPG*. GSEA revealed significant enrichment of the glycine, serine, and threonine metabolism pathway in the same comparison. **c** Venn diagram demonstrating intersection between SIRPγ⁻ and SIRPγ⁺ CD8⁺ T cell differentially expressed genes and the glycine, serine, and threonine metabolism pathway genes (*PHGDH*, *ALDH7A1*, *GCAT*). **d** Relative *PHGDH* and *SIRPG* expression in sorted SIRPγ⁻ and SIRPγ⁺ CD8⁺ T cells from TPE assessed by qPCR (*n* = 3 biological replicates) (**e**) PHGDH expression SIRPγ^low^ and SIRPγ^high^ CD8⁺ T cells derived from single-cell RNA sequencing data. **f** CD8⁺ T cells isolated from TPE were activated with anti-CD3/CD28 antibodies (24 h), followed by 48-hour treatment with anti-SIRPγ monoclonal antibody LSB2.20(10 μg/mL). *PHGDH* gene expression was analyzed by qPCR (*n* = 3 biological replicates). **g**, **h** Immunofluorescence of PHGDH in sorted SIRPγ⁻ and SIRPγ⁺ CD8⁺ T cells (*n* = 3 independent experiments). **i** Western blot of SIRPγ and PHGDH in sorted SIRPγ⁻ and SIRPγ⁺ CD8⁺ T cells (*n* = 3 biological replicates). **j**, **k** CD8⁺ T cells isolated from TPE were activated with anti-CD3/CD28 antibodies (24 h), followed by 48 h treatment with LSB2.20(10 μg/mL). PHGDH protein was analyzed (*n* = 3 biological replicates). **l** Intracellular L-serine levels measured by ELISA in sorted SIRPγ⁻ and SIRPγ⁺ CD8⁺ T cells from TPE (*n* = 3 independent experiments). **m** L-serine quantification after LSB2.20 treatment in activated CD8⁺ T cells CD8⁺ T cells (*n* = 3 independent experiments). **n** Incorporation of U-[^13^C]-glucose carbon into serine in sorted SIRPγ⁻ and SIRPγ⁺ CD8⁺ T cells derived from TPE (*n* = 8 patients). Statistical significance was determined by unpaired two-tailed Student’s *t* test (**d**, **f**, **h**, **l**, **m**) and paired two-tailed *t* tests (**k**, **n**). Data are presented as mean values ± SD (**d**, **f**, **h**, **k**–**n**).

Distinct T cell differentiation states (e.g., effector, memory, and exhausted subsets) require compartmentalized metabolic reprogramming to sustain functional plasticity in chronic inflammatory infections. Transitions among these states are governed by dynamic metabolic rewiring, particularly substrate utilization shifts^27^. Thus, this study specifically focused on the metabolic pathways distinguishing SIRPγ⁻ and SIRPγ⁺ CD8⁺ T cells. SIRPγ⁻ and SIRPγ⁺ CD8⁺ T cells sorted by flow cytometry were subjected to RNA sequencing. Differential gene expression analysis followed by GSEA revealed pronounced upregulation of key metabolic pathways in SIRPγ⁺ CD8⁺ T cells. Single-gene GSEA targeting *SIRPG* further demonstrated a strong correlation between these metabolic pathways and *SIRPG* expression. The intersection of these analyses revealed that the upregulated glycine, serine, and threonine metabolism pathway may be functionally associated with SIRPγ-mediated T cell exhaustion (Fig. 4a, b). To identify key regulators of this pathway, glycine, serine, and threonine metabolism pathway–associated genes were intersected with differentially expressed genes. This integrative analysis identified *PHGDH*, *ALDH7A1*, and *GCAT* as candidate genes potentially involved in SIRPγ-mediated T cell exhaustion (Fig. 4c). These candidates were validated by qPCR (Fig. 4d and Supplementary Fig. S10a). Intriguingly, scRNA-seq revealed that *PHGDH* and *GCAT*, but not *ALDH7A1*, were differentially expressed between SIRPγ⁺ and SIRPγ⁻ CD8⁺ T cells (Fig. 4e and Supplementary Fig. S10b, c). It was further found that SIRPγ blockade selectively reduced *PHGDH* expression without significantly affecting *ALDH7A1* or *GCAT* (Fig. f and Supplementary Fig. S10d, e). These results indicate that SIRPγ might promote T cell exhaustion through the PHGDH-mediated glycine, serine, and threonine metabolism pathway.

PHGDH, the first of three sequential enzymes in the serine synthesis pathway (SSP), drives endogenous serine biosynthesis^28^. An immunosuppressive phenotype in tumor-associated macrophages has been shown to be promoted by PHGDH-mediated serine metabolism^29–32^. Moreover, PHGDH-overexpressing tumor cells exhibit enhanced proliferation and a heightened propensity for lymph node metastasis^33^. In TB, D-serine derived from Mtb impairs CD8^+^ T cell function, thereby compromising control of bacterial pathogenesis^34^. However, the contribution of PHGDH-mediated serine metabolism to CD8⁺ T cell function within TPE remains poorly defined. Taken together, these findings suggest that serine metabolism, particularly its rate-limiting step mediated by PHGDH, is likely to represent a central link between SIRPγ signaling and the metabolic reprogramming of T cell function. Supporting this hypothesis, immunofluorescence and western blotting confirmed elevated levels of PHGDH in SIRPγ^+^CD8^+^ T cells (Fig. 4g–i and Supplementary Fig. S10f). And SIRPγ blockade decreased PHGDH expression (Fig. 4j, k). To validate the activation of serine metabolism pathways, intracellular serine levels were quantified via ELISA in FACS-sorted CD8⁺ T cell subsets. Higher serine levels were observed in SIRPγ⁺CD8⁺ T cells relative to SIRPγ⁻CD8⁺ T cells (Fig. 4l). Consistently, SIRPγ blockade substantially reduced serine levels of CD8⁺ T cells (Fig. 4m). To further investigate the origins and metabolic flux of metabolites in SIRPγ⁺ CD8⁺ T cells, we performed metabolic flux analysis using U-[^13^C]-glucose tracing on sorted T cell subsets. Following 6 hours of culture, SIRPγ⁺ CD8⁺ T cells exhibited enhanced glycolytic flux along with increased activity in its branch pathways, including de novo serine synthesis and one-carbon metabolism (Fig. 4n, S10g, h). Glucose-derived isotopic labeling was significantly higher in SIRPγ⁺ cells than in SIRPγ⁻ cells, as reflected in the serine pools (Supplementary Fig. S10i), indicating enhanced flux through the serine synthesis pathway and into one-carbon units. Notably, no significant increase in the glycine pool was observed (Supplementary Fig. S10j). Collectively, these data suggest that SIRPγ reprograms serine metabolism in CD8⁺ T cells through the upregulation of PHGDH expression.

### SIRPγ drives T cell exhaustion through PHGDH-mediated serine metabolism in TPE

**Fig. 5:**
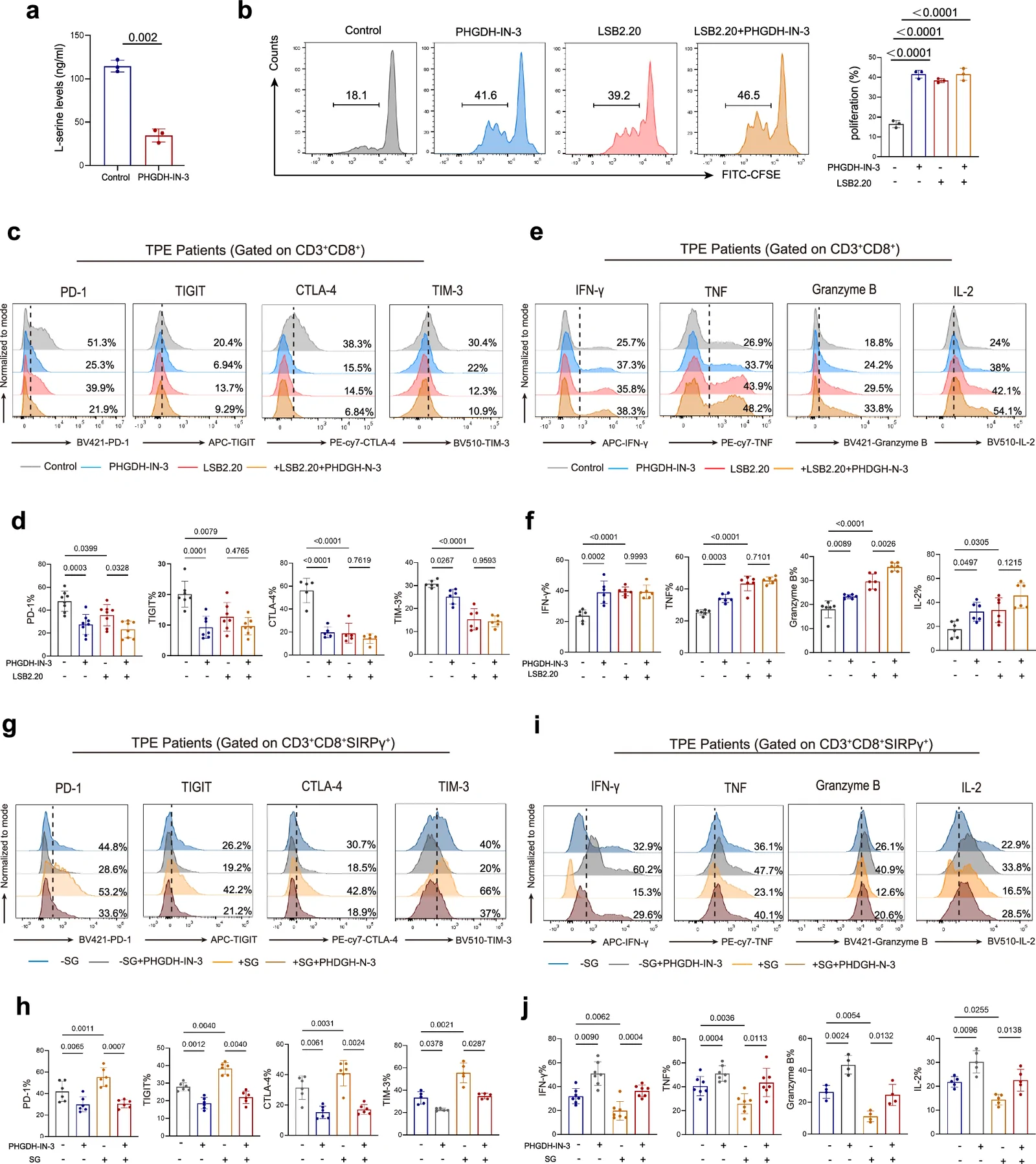
SIRPγ drives T cell exhaustion through PHGDH-mediated serine metabolism in TPE. **a** CD8⁺ T cells isolated from TPE were treated with PHGDH inhibitor PHGDH-IN-3 (10 μM, 48 h) following initial activation by anti-CD3/CD28 antibodies (24 h). Intracellular L-serine levels were quantified by ELISA (n = 3 independent experiments). **b–f** TPE-isolated CD8⁺ T cells were activated (anti-CD3/CD28, 24 h), pretreated with or without anti-SIRPγ monoclonal antibody LSB2.20 (10 μg/mL, 24 h), followed by PHGDH-IN-3 (48 h). Proliferation was assessed via CFSE dilution (**b**) (*n* = 3 independent experiments). Flow cytometry quantified expression of inhibitory receptors (TIGIT, TIM-3, CTLA-4, PD-1) (**c**, **d**) (*n* = 6 patients) and cytokines (IL-2, IFN-γ, Granzyme B, TNF) (**e**, **f**) (*n* = 6 patients). **g**–**j** TPE-isolated CD8⁺ T cells were treated with or without PHGDH-IN-3 (24 h), with or without serine/glycine (SG) deprivation (3 d). Expression of inhibitory receptors (TIGIT, TIM-3, CTLA-4, PD-1) (**g**, **h**) and cytokines (IL-2, IFN-γ, Granzyme B, TNF) (**i**, **j**) were analyzed by flow cytometry. The number of independent replicates is indicated as follows: *n* = 7 (IFN-γ, TNF), *n* = 6 (PD-1, TIGIT, CTLA-4), *n* = 5 (TIM-3, IL-2), and *n* = 4 (Granzyme B) per group. Statistical analysis was determined by unpaired two-tailed *t* test (**a**) and one-way ANOVA (**b**, **d**, **f**, **h**, **j**). Data are presented as mean values ± SD (**a**, **b**, **d**, **f**, **h**, **j**).

To investigate whether SIRPγ drives CD8⁺ T cell exhaustion through PHGDH-mediated serine metabolism, CD8⁺ T cells derived from TPE were treated with the PHGDH inhibitor PHGDH-IN-3. PHGDH-IN-3 reduced serine levels (Fig. 5a) and augmented the proliferation of CFSE-labeled CD8⁺ T cells (Fig. 5b). Furthermore, PHGDH-IN-3 downregulated immunosuppressive receptors (CTLA-4, TIM-3, PD-1, TIGIT) while enhancing effector cytokines production (IL-2, TNF, Granzyme B) (Fig. 5c–f). Notably, the combination of SIRPγ blockade and PHGDH inhibition did not produce additive effects beyond those observed with PHGDH-IN-3 alone, suggesting that SIRPγ likely promotes CD8⁺ T cell exhaustion predominantly through PHGDH. In addition, in the absence of E-C fusion antigen stimulation, PHGDH-IN-3 exhibited minimal effects on CD8⁺ T cells isolated from the PB of PPD^+^ volunteers. In contrast, PHGDH-IN-3 markedly suppressed exhaustion in SIRPγ⁺CD8⁺ T cells under E-C treatment, as evidenced by reduced immunosuppressive receptors (CTLA-4, TIM-3, PD-1, TIGIT), increased effector cytokines secretion (IFN-γ, IL-2), and enhanced proliferative capacity (Supplementary Fig. S11a–e). These findings demonstrate that SIRPγ promotes CD8⁺ T cell exhaustion via upregulating PHGDH in TPE.

We next assessed whether serine availability regulates CD8⁺ T cell exhaustion. Treatment with L-phenylglycine (L-phg), a selective inhibitor of alanine-serine-cysteine-threonine transporters^35^, significantly reduced intracellular serine levels in CD8⁺ T cells (Supplementary Fig. S11f). Although L-phg did not alter CD69, PD-1, TIGIT, or IFN-γ levels, it significantly decreased exhaustion markers (CTLA-4, TIM-3) while enhancing effector cytokines (IL-2, TNF, Granzyme B) secretion capacity, and proliferative potential (Supplementary Fig. S11g–k). In line with these findings, serine and glycine depletion (-SG) in culture medium reduced intracellular serine levels in CD8⁺ T cells derived from TPE (Supplementary Fig. S12a). SG starvation in SIRPγ⁺CD8⁺ T cells markedly downregulated immunosuppressive receptors (CTLA-4, TIM-3, PD-1, TIGIT) and increased production of effector cytokines (IL-2, TNF, IFN-γ, Granzyme B) (Fig. 5g–j). Furthermore, SG starvation enhanced proliferation of CD8⁺ T cell (Supplementary Fig. S12b). Notably, co-treatment with exogenous SG depletion with PHGDH-IN-3 synergistically attenuated SIRPγ^+^CD8⁺ T cell exhaustion, surpassing the effects of PHGDH inhibition alone. This indicates a complementary interaction between serine metabolism and PHGDH activity in regulating T cell exhaustion. Similarly, this combination significantly suppressed exhaustion in SIRPγ⁺CD8⁺ T cells derived from the PB of TPE patient (Supplementary Fig. S12c–g). Collectively, these findings underscore that SIRPγ drives CD8⁺ T cell exhaustion in TPE through PHGDH - regulated serine metabolism.

### Serine promotes SAM-dependent T cell exhaustion

Serine is a key contributor to one-carbon metabolism, thereby supporting the biosynthesis of nucleotides, SAM, glutathione (GSH), and nicotinamide adenine dinucleotide phosphate (NADPH)^36–38^. Consistent with this, our data reveal that SIRPγ⁺CD8⁺ T cells possess elevated serine levels and enhanced one-carbon metabolism. Recent findings highlight the serine-dependent generation of GSH for IL-1β production in macrophages^31^, the production of NADPH to maintain redox balance in stem and cancer cells^39^, and the provision of SAM for epigenetic regulation of macrophage polarization^32^.

**Fig. 6:**
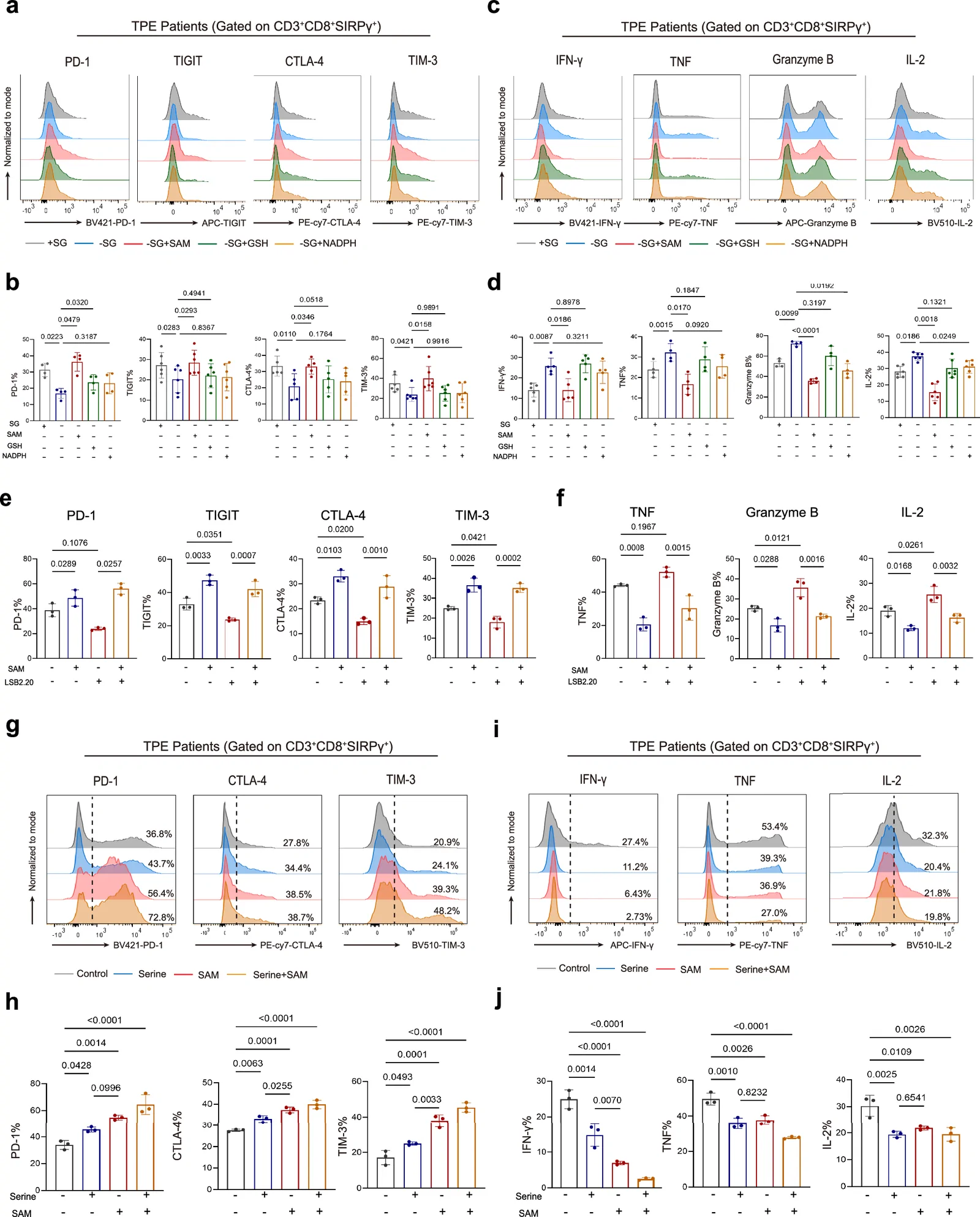
Serine promotes SAM-dependent T cell exhaustion. **a**–**d** CD8⁺ T cells isolated from TPE were subjected to serine/glycine (SG) medium starvation for 3 days. Cells were subsequently treated with 400 μM NADPH, GSH, or SAM for 48 h. **a**, **b** Flow cytometry was conducted to evaluate the expression levels of inhibitory receptors (TIGIT, TIM-3, CTLA-4, PD-1) (**a**, **b**) and cytokines (IL-2, IFN-γ, Granzyme B, TNF). **c**, **d** The number of independent replicates is indicated as follows: *n *= 6 (TIM-3, TIGIT, IFN-γ, IL-2), *n* = 5 (CTLA-4), and *n* = 4 (PD-1, Granzyme B, TNF) per group. **e**, **f** CD8⁺ T cells from TPE were treated with the specific anti-SIRPγ monoclonal antibody LSB2.20 (10 μg/mL) for 24 h, followed by 400 μM SAM treatment for 48 h. Flow cytometry was performed to assess the expression levels of inhibitory receptors (TIGIT, TIM-3, CTLA-4, PD-1) (**e**) and cytokines (IL-2, IFN-γ, Granzyme B, TNF) (**f**). *n* = 3 biological replicates per group. **g**–**j** CD8⁺ T cells with SIRPγ blockade obtained via the above method were treated with or without 400 μM serine or SAM and cultured for 3 days. Flow cytometry was employed to measure the levels of inhibitory receptors (TIM-3, CTLA-4, PD-1) (**g**, **h**) and cytokines (IL−2, IFN-γ, TNF) (**i**, **j**). *n* = 3 biological replicates per group for (**h**) and (**j**). Statistical analysis was determined by one-way ANOVA (**b**–**f**, **h**, **j**). Data are presented as mean values ± SD (**b–f**, **h**, **j**).

Next, we sought to identify serine-derived metabolites that regulate CD8⁺ T cell exhaustion. To rescue the exhaustion phenotype reduced by SG deprivation, the cultures were supplemented with NADPH, GSH, or SAM. Notably, only SAM supplementation reversed the beneficial effects of serine deprivation, as evidenced by increased immunosuppressive receptors (CTLA-4, TIM-3, PD-1, TIGIT), reduced levels of cytokines (IL-2, TNF, IFN-γ, Granzyme B) and impaired proliferative capacity in SIRPγ⁺CD8⁺ T cells (Fig. 6a–d and Supplementary Fig. S13a). As anticipated, we further found that SIRPγ⁺ CD8⁺ T cells showed increased levels of U-[¹³C]-glucose-derived SAM and a significantly higher labeling fraction of glucose-derived SAM (Supplementary Fig. S13b, c), whereas SIRPγ blockade reduced SAM abundance (Supplementary Fig. S13d). Similarly, SG deprivation combined with PHGDH inhibition significantly decreased SAM levels in CD8⁺ T cells from TPE (Supplementary Fig. S13e). Notably, SAM supplementation rescued the alterations in immunosuppressive receptor expression and effector cytokine production induced by SIRPγ blockade (Fig. 6e, f and Supplementary Fig. S13f, g). Intriguingly, exogenous serine only partially reversed SIRPγ blockade-reduced exhaustion, with a less pronounced effect than SAM (Fig. 6g–j). This discrepancy may be attributed to the fact that, while serine contributes to SAM biosynthesis, a portion of it is diverted toward the synthesis of other metabolites. Consequently, serine alone leads to only a modest increase in SAM levels. Collectively, these data underscore the pivotal role of serine-derived SAM in modulating CD8⁺ T cell exhaustion.

### Serine-derived SAM promotes an active epigenetic state at the NFATC2 locus to drive T cell exhaustion

Growing evidence suggests that epigenetic modifications shape differentiation trajectories and govern CD8⁺ T cell exhaustion^40^. Notably, there is substantial crosstalk between epigenetic regulators and key metabolic intermediates, including acetyl-CoA and SAM^41–43^. As a principal methyl donor, serine-derived SAM plays a pivotal role in modulating chromatin states and gene transcription^44–46^. Specifically, SAM enhances the N6-methyladenosine (m6A) methylation of immune checkpoint transcripts, including PD-L1 and V-domain Ig suppressor of T cell activation (VISTA), thereby promoting their translation^41^. Moreover, SAM contributes to T cell dysfunction by reshaping genome-wide chromatin accessibility^47^. However, the precise role of SAM in modulating histone methylation within CD8⁺ T cells remains incompletely understood.

**Fig. 7:**
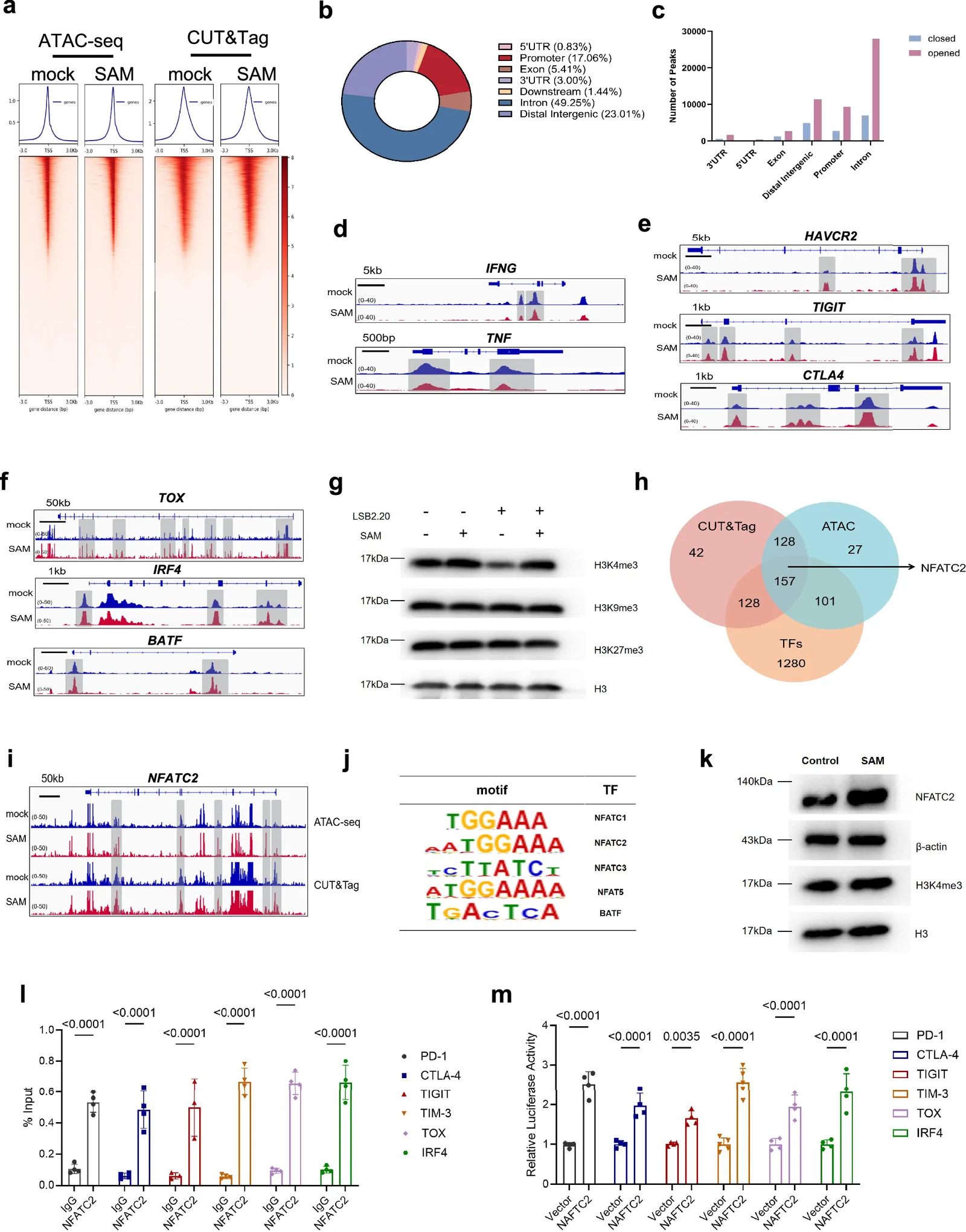
Serine-derived SAM promotes an active epigenetic state at the NFATC2 locus to drive T cell exhaustion. **a** Heatmap illustrating ATAC-seq and CUT&Tag-seq coverage at loci exhibiting differential chromatin accessibility or altered H3K4me3 modifications in SAM-treated CD8⁺ T cells derived from TPE compared to the mock group. **b** Pie chart illustrating the genomic distribution of ATAC-seq peaks in mock- or SAM-treated CD8^+^T cells. **c** Quantification of treatment-induced ATAC-seq peak alterations. **d**–**f** ATAC-seq coverage at *IFNG* and *TNF* (**d**); *HAVCR2*, *TIGIT*, and *CTLA4* (**e**); and *TOX*, *IRF4*, and *BATF* (**f**). All peaks were highlighted in gray bars. **g** Western blot analysis was performed to assess histone modification markers in CD8⁺ T cells derived from TPE. Following stimulation with anti-CD3/CD28 antibodies for 24 h, the cells were treated with the anti-SIRPγ monoclonal antibody LSB2.20 (10 μg/mL), and then supplemented with SAM (400 μM) for 48 h (*n* = 3 independent experiments). **h** Venn diagram identifying overlapping upregulated genes from transcription factor libraries, CUT&Tag-seq, and ATAC-seq in SAM-treated CD8^+^T cells versus the mock group. **i** Integrated ATAC-seq and H3K4me3 CUT&Tag-seq coverage are shown across the *NFATC2* genomic region. **j** Transcription factor binding motifs with increased chromatin accessibility, as detected by ATAC-seq, are enriched in SAM-treated CD8⁺ T cells. **k** Protein expression of NFATC2 and H3K4me3 was analyzed by Western blot in mock- or SAM-treated CD8^+^ T cells (*n* = 3 independent experiments). **l** The binding of NFATC2 to the promoter regions of TIGIT, TIM-3, CTLA-4, PD-1, TOX, IRF4 was determined by ChIP-qPCR in CD8^+^ T cells isolated from TPE. *n* = 4 biological replicates per group for TIM-3, CTLA-4, PD-1, TOX, IRF4 and *n* = 3 biological replicates per group for TIGIT. **m** Transcriptional regulation of key immune-related genes (TIGIT, TIM-3, CTLA-4, PD-1, TOX, IRF4) by NFATC2 was evaluated using luciferase reporter assays in 293 T cells. *n* = 5 biological replicates per group for TIM-3, TIGIT, PD-1, TOX, IRF4 and *n* = 4 biological replicates per group for CTLA-4. Statistical significance was determined by two-way ANOVA (**l**, **m**). Data are presented as mean values ± SD (**l**, **m**).

To explore the epigenetic regulatory role of SAM, genome‑wide chromatin accessibility was initially assessed by ATAC‑seq in SAM‑treated CD8⁺ T cells derived from TPE. Comparative analysis revealed 33,440 differentially accessible peaks between SAM-treated and mock-treated cells. The majority of these changes (over 72%) occurred in intronic or intergenic regions, indicative of enhancer activity, whereas roughly 17% were situated within promoter regions (Fig. 7a left, b, c). Notably, while SAM reduced chromatin accessibility at genes associated with effector functions (*IFNG*, *TNF*) (Fig. 7d), it concurrently increased accessibility at intergenic regions near exhaustion-associated loci, including *HAVCR2* (encoding TIM-3), *CTLA4*, *TIGIT*, *CD38*, and *LAG3* (Fig. 7e and Supplementary Fig. S14a). This pattern mirrors chromatin changes characteristic of exhausted T cells^48,49^. Furthermore, SAM enhanced accessibility at genes encoding transcription factors known to drive exhaustion, including *BATF*, *IRF4*, *TOX*^50–53^ (Fig. 7f). These findings suggest that SAM induces epigenetic reprogramming at loci linked to both exhaustion and effector function. Notably, SAM also activated chromatin accessibility at genes downstream of T cell receptor (TCR) signaling (*ZAP70*, *NFKB1*, and members of the nuclear factor of activated T cells (NFAT) family (*NFATC1–4*)) (Fig. 7i and Supplementary Fig. S14b, c). Concordantly, GSEA analysis demonstrated marked upregulation of TCR signaling pathways and histone modification processes in SAM-treated CD8⁺ T cells (Supplementary Fig. S14d).

Given that SAM serves as a critical methyl donor involved in histone methylation, it is essential for the regulation of chromatin structure and transcriptional activity in T cells and macrophages^32,44,54^. To investigate whether SAM modulates histone modification in CD8⁺ T cells, we performed Western blot analysis using antibodies specific for common histone marks. SIRPγ blockade selectively reduced H3K4me3 levels, without affecting H3K27me3 or H3K9me3, and this reduction was rescued by SAM treatment (Fig. 7g and Supplementary Fig. S14e). Similarly, PHGDH inhibition and SG deprivation led to decreased H3K4me3 levels, which were restored upon SG supplementation (Supplementary Fig. S14f). To further identify transcriptionally active regions marked by H3K4me3, we employed CUT&Tag using H3K4me3-specific antibodies in CD8⁺ T cells derived from TPE. The results revealed that SAM treatment predominantly increased H3K4 methylation at promoter regions and intronic sites across the genome (Fig. 7a right, Supplementary Fig. S14g). Considering that transcription factors play a central role in driving T cell exhaustion, we next examined whether SAM influenced their expression. A multi-omics analysis of SAM-treated CD8⁺ T cells was conducted by integrating data from a curated human transcription factor database, ATAC-seq, and H3K4me3 CUT&Tag-seq. This analysis identified 157 genes exhibiting both enhanced chromatin accessibility and elevated H3K4me3 levels (Fig. 7h). Notably, we observed increased chromatin accessibility and elevated H3K4me3 levels at the promoter region of *NFATC2 (NFAT1)* (Fig. 7i). Intriguingly, transcription factor motif enrichment analysis revealed that the open chromatin regions were enriched for canonical motifs associated with NFATC1, NFATC2, NFATC4, NFAT5, and BATF, further supporting the notion that SAM modulates gene transcription networks through epigenetic mechanisms (Fig. 7j).

Indeed, the NFAT family, well-established as essential downstream effectors of TCR signaling in T cell activation, has also been strongly associated with the induction of T cell exhaustion under conditions of chronic infection, with NFATC1 and NFATC2 serving as key transcription factors that mediate the expression of inhibitory receptors^55–57^. Under chronic stimulation, these factors are persistently upregulated in exhausted T cells and exhibit enhanced binding to the promoter regions of *IRF4* and *TOX*, thereby driving their transcriptional activation^58–60^. Consistent with these observations, SAM-treated CD8⁺ T cells displayed elevated *NFATC2* expression, accompanied by transcriptional upregulation of inhibitory receptors and exhaustion-associated transcription factors, including *TOX*, *BATF*, and *IRF4*. This transcriptional program was mirrored at the protein level, as Western blot confirmed that SAM increased H3K4me3 and upregulated NFATC2, TOX, and IRF4 expression (Fig. 7k and Supplementary Fig. S14h, i). ChIP-qPCR and luciferase reporter assays were employed to investigate the regulatory relationship between NFATC2 and immunosuppressive receptors, as well as exhaustion-associated transcription factors. These experiments confirmed that NFATC2 binds to the promoter regions of multiple target genes, including *PD-1*, *HAVCR2*, *TIGIT*, *CTLA4*, *TOX*, and *IRF4* (Fig. 7l, m). Moreover, SAM-cultured CD8⁺ T cells were treated with the specific NFAT inhibitor cyclosporine A (CsA) to determine the functional requirement for NFATC2. CsA treatment effectively abrogated the SAM-induced upregulation of inhibitory receptors (PD-1, CTLA-4, TIGIT) and restored the production of effector cytokines (IFN-γ, TNF, Granzyme B) (Supplementary Fig. S15a–d), indicating that NFAT activity is necessary for SAM to promote the exhaustion phenotype.

Considering that NFAT activation has been reported to be classically dependent on calcium influx downstream of TCR signaling^61^, we sought to determine whether SAM influences this key signaling axis. Notably, we found that SAM not only enhanced TCR signaling (Supplementary Fig. S14d) but, more importantly, directly augmented calcium flux and promoted NFATC2 nuclear translocation, even under conditions of weak calcium stimulation (Supplementary Fig. S15e, f). Together, these data support a model whereby SAM promotes CD8⁺ T cell exhaustion by inducing an active epigenetic state at the NFATC2 locus and synergizing with TCR-dependent calcium signaling to promote NFATC2 activation.

### CD47-SIRPγ interaction promotes PHGDH expression via PI3K/AKT /mTOR pathway

**Fig. 8:**
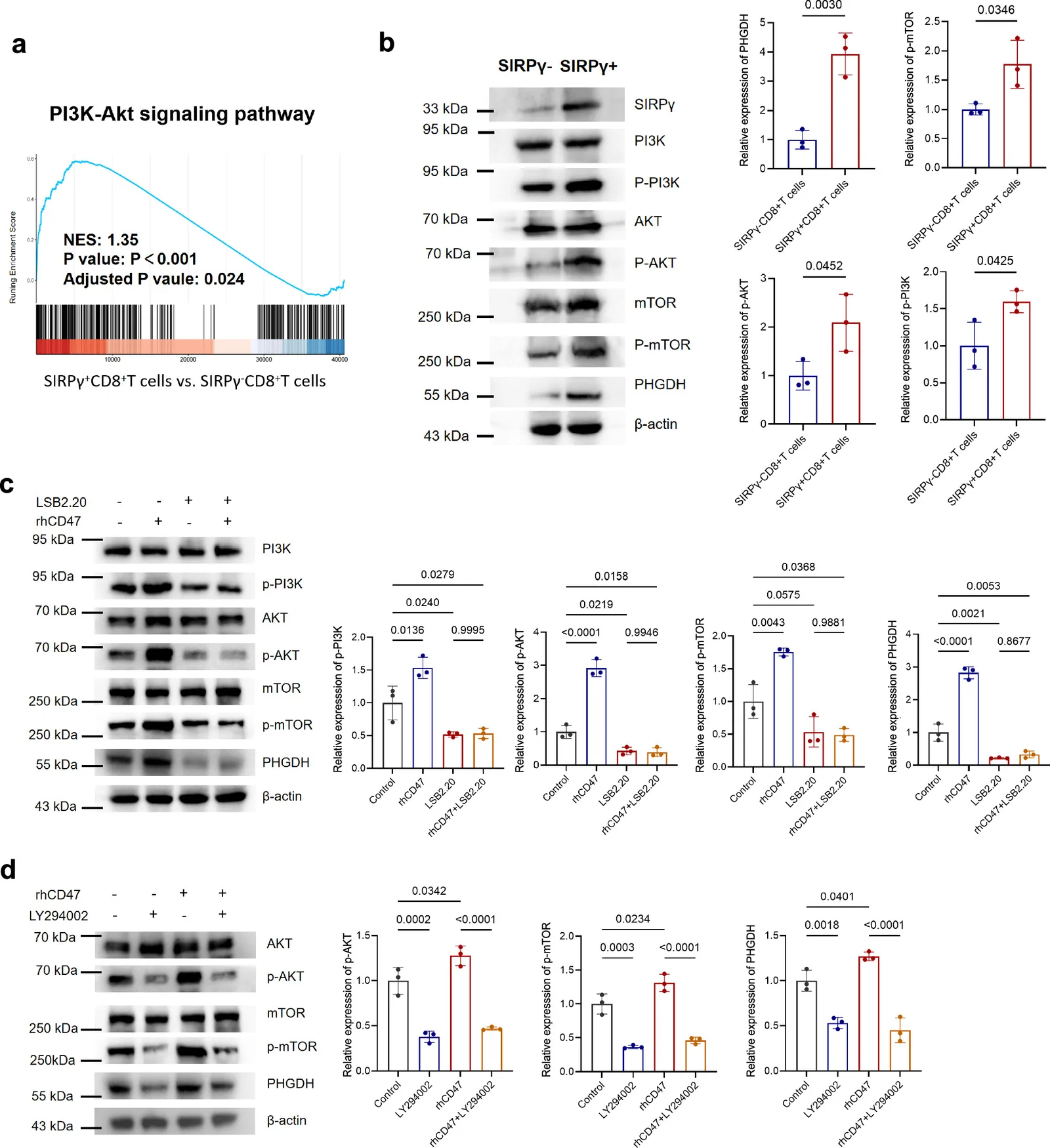
CD47-SIRPγ interaction promotes PHGDH expression via PI3K/AKT/mTOR pathway. **a** Gene-set enrichment analysis was performed on gene sets of PI3K-Akt signaling pathway. **b** Protein expression levels of SIRPγ, total and phosphorylated forms of PI3K, AKT, and mTOR, as well as PHGDH, were assessed by Western blotting in sorted SIRPγ⁻ and SIRPγ⁺ CD8⁺ T cells derived from TPE (*n* = 3 biological replicates per group). **c** CD8⁺ T cell isolated from TPE were treated with either recombinant human CD47 Protein (rhCD47) (Sino Biological, 10 ng/mL, administered 6 hours after LSB2.20 treatment) or anti-SIRPγ monoclonal antibody LSB2.20 (10 μg/mL) for 48 h. The expression levels of SIRPγ, total and phosphorylated forms of PI3K, AKT, and mTOR, as well as PHGDH, were then measured (n = 3 biological replicates per group). **d** CD8⁺ T cells isolated from TPE were treated with either recombinant human CD47 protein (Sino Biological, 10 ng/mL) or a PI3K inhibitor (LY294002, 10 μM) for 48 h. The expression levels of SIRPγ, total and phosphorylated forms of AKT, and mTOR, as well as PHGDH, were subsequently detected (*n* = 3 biological replicates per group). Statistical analysis was determined by unpaired two-tailed Student’s *t* test (**b**) and one-way ANOVA (**c**, **d**). Data are presented as mean values ± SD (**b**–**d**).

We previously demonstrated that SIRPγ promoted the expression of PHGDH, although the underlying mechanism remained elusive. Next, we observed that the PI3K-AKT pathway was markedly upregulated in SIRPγ⁺CD8⁺ T cells compared to SIRPγ⁻CD8⁺ T cells (Fig. 8a). This pathway is known to induce PHGDH expression^62–64^. Consistent with this, higher protein levels of PHGDH, p-PI3K, p-AKT, and p-mTOR were confirmed in SIRPγ⁺CD8⁺ T cells by Western blotting (Fig. 8b). Current evidence indicates that SIRPγ is primarily expressed on T cells and interacts with its ligand CD47, thereby initiating the activation of downstream signaling pathways^22^. Based on this, we hypothesized that CD47 binding to SIRPγ activated the PI3K-AKT pathway, thereby promoting PHGDH expression. As expected, treatment with recombinant CD47 protein increased the levels of PHGDH, p-PI3K, p-AKT, and p-mTOR in CD8⁺ T cells isolated from TPE. Notably, this effect was abrogated upon SIRPγ blockade (Fig. 8c). Likewise, treatment with the PI3K inhibitor LY294002^65^ led to significant downregulation of PHGDH expression (Fig. 8d). These results indicate that CD47–SIRPγ interaction promotes PHGDH expression through the PI3K/AKT /mTOR pathway.

## Discussion

**Fig. 9:**
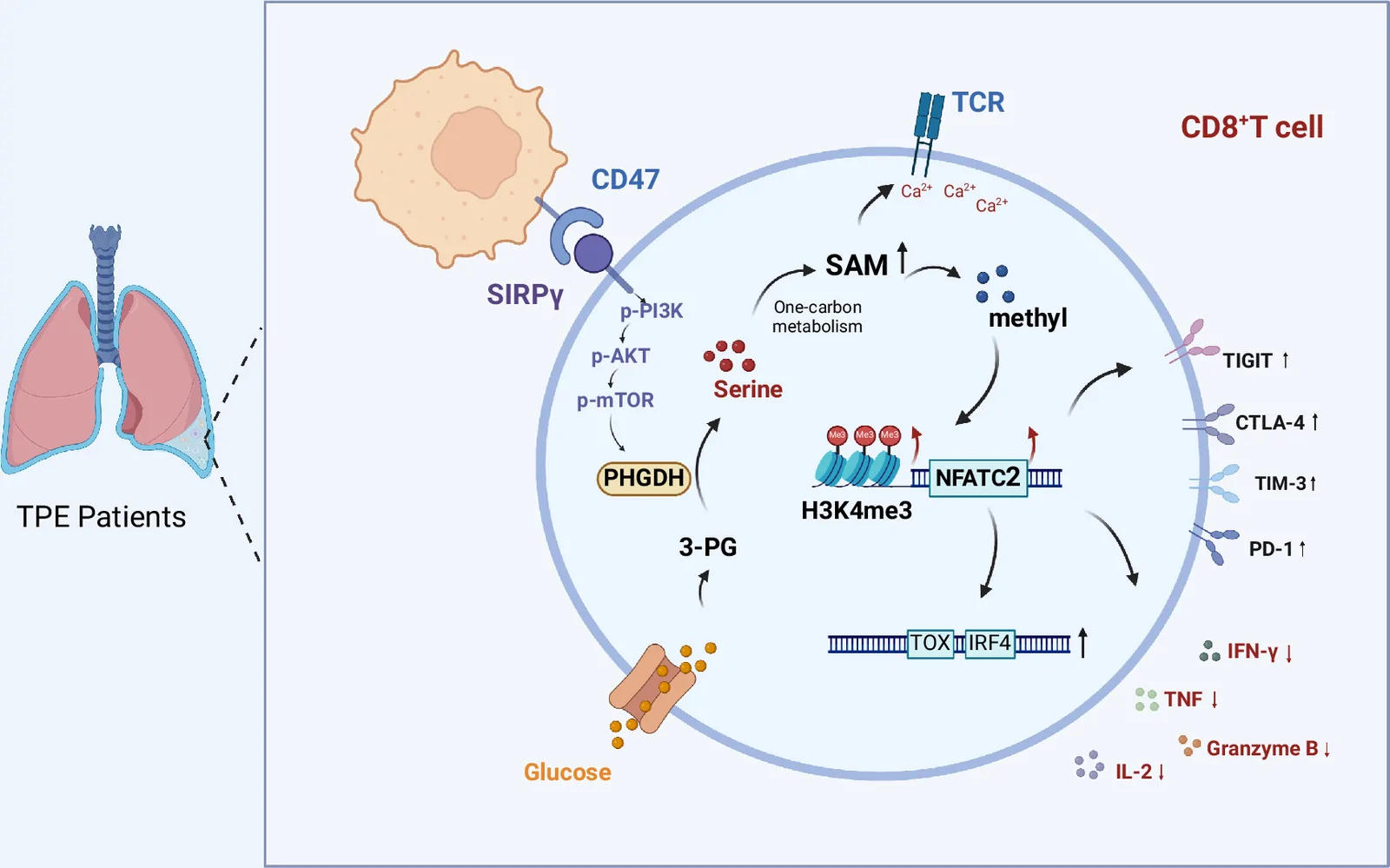
SIRPγ induces CD8^+^ T cell exhaustion through serine metabolism reprogramming in TPE. SIRPγ promotes CD8⁺ T-cell exhaustion via interaction with CD47, leading to activation of the PI3K/AKT/mTOR pathway. This cascade enhances serine metabolism by upregulating PHGDH and subsequently promotes SAM-dependent histone H3 lysine 4 (H3K4me3) epigenetic modification at the NFATC2 locus. This modification, in coordination with TCR-induced calcium signaling, drives NFATC2 activation. NFATC2, in turn, promotes the expression of exhaustion-related transcription factors (TOX, IRF4) and immunosuppressive receptors (TIGIT, CTLA-4, PD-1, TIM-3), and reduces the production of cytokines (IL-2, TNF, IFN-γ, granzyme B). The schematic diagram was created in BioRender. Yang, Y. (2026) https://BioRender.com/87zw3r0.

In this study, we identified SIRPγ as a potential biomarker for distinguishing TPE from non-TPE through high-throughput cytokine array analysis. A diagnostic threshold for SIRPγ was established using a training cohort and subsequently validated in an independent validation cohort, confirming its diagnostic utility. Notably, we further demonstrated that SIRPγ acted as a key regulator of CD8⁺ T cell exhaustion. Mechanistically, SIRPγ interacted with CD47 to activate the PI3K/AKT/mTOR signaling pathway, leading to the upregulation of PHGDH and the enhancement of serine metabolism. In turn, elevated serine levels promoted SAM-dependent H3K4me3 epigenetic modification at the NFATC2 locus, which, in coordination with TCR-induced calcium signaling, drove NFATC2 activation. Ultimately, this signaling cascade induced the expression of exhaustion-associated transcription factors and immunosuppressive receptors (Fig. 9).

The high-throughput cytokine array analysis was performed on supernatant samples to investigate potential biomarkers distinguishing TPE from MPE. The results revealed that soluble SIRPγ (CD172g) was significantly elevated in TPE and exhibited promising diagnostic utility. This finding was subsequently validated in both an internal and an external cohort, confirming its capacity to differentiate TPE from non-TPE, including MEP, PPE, and miscellaneous. Moreover, combining SIRPγ with ADA further enhanced the diagnostic accuracy achieved by ADA alone. Nevertheless, current studies indicate that SIRPγ, as a transmembrane protein, lacks a known secretory form, our sequencing data revealed its presence in the pleural effusion supernatant. This may be attributed to proteolytic cleavage or its incorporation into exosome vesicles by T cells. Whether SIRPγ exerts a direct influence on the TPE microenvironment remains unclear.

Our study demonstrated that SIRPγ was predominantly expressed on T cells, particularly within the CD4⁺ and CD8⁺ subsets, with low levels also detected on NK cells. Notably, SIRPγ expression on CD4⁺ T cells, CD8⁺ T cells, and NK cells was significantly higher in TPE compared to MPE. Further analysis classified CD8⁺ T cells in TPE into distinct subsets, including T_N_, T_EM_, T_CM_, T_EFF_, and T_EX_ populations. In TPE, the presence of T_N_ cells likely reflects recent recruitment from peripheral circulation and limited activation within the pleural microenvironment. Upon antigen stimulation, T_N_ rapidly differentiate into T_EFF_, which contribute to anti-mycobacterial immunity through cytotoxic activity against infected macrophages and production of IFN-γ and TNF^66^. Regardless of whether the antigen is completely cleared, a fraction of T_EFF_ persists as self-renewing memory T cells, enabling rapid immune responses upon re-exposure^67^. In the context of persistent infection, CD8^+^ T cells progressively differentiate into T_EX_, characterized by reduced proliferation, diminished cytokine secretion, and upregulation of inhibitory receptors. The decline in IFN-γ and TNF levels, together with impaired cytotoxic function, may compromise local anti-mycobacterial immunity and thereby contribute to disease persistence. T cell exhaustion is thus a hallmark of TPE and represents a potential target for immunotherapeutic intervention^26^. Notably, SIRPγ expression was predominantly localized to T_EX_, which exhibited impaired effector function. Its expression levels were positively correlated with multiple immunosuppressive receptors (CTLA-4, TIM-3, PD-1, TIGIT) and negatively correlated with effector-associated cytokines (Granzyme B, TNF, IFN-γ, IL-2). Furthermore, the proportion of SIRPγ⁺ CD8⁺ T cells was significantly elevated in TPE compared to PB, whereas no such increase was observed in CD4⁺ T cells. These findings suggest that the significance of SIRPγ extends beyond its diagnostic contribution; it also reveals key immunological features of the TPE microenvironment and identifies a potential target for modulating CD8^+^ T cell dysfunction. Thus, SIRPγ may serve not only as a complementary biomarker in cases with equivocal ADA results, but also as an indicator providing insight into the extent of local immune exhaustion in clinical practice.

To uncover the mechanisms by which CD8⁺ T cell exhaustion is regulated by SIRPγ, differentially expressed genes between SIRPγ⁺ and SIRPγ⁻ CD8⁺ T cells were identified through RNA-seq analysis. Given the distinctive metabolic dysregulation observed in T_EX_ cells, we focused on the significantly altered metabolic pathways. SIRPγ⁺ CD8⁺ T cells exhibited significantly enhanced serine metabolism, accompanied by elevated expression of the key enzyme PHGDH. Given the persistent inflammatory response and potential hypoxic niches within the TPE microenvironment, activated T cells generate reactive oxygen species (ROS), thereby inducing a requisite antioxidant GSH response. Notably, serine-derived GSH serves as a critical antioxidant^68^, suggesting enhanced serine metabolism in CD8⁺ T cells may represent an adaptive response to oxidative stress within TPE, albeit at the cost of effector function and promotion of exhaustion. Thus, we proposed that SIRPγ facilitated T cell exhaustion through PHGDH-mediated serine metabolic reprogramming. Serine has been shown to drive enzymes of the serine-glycine-one-carbon (SGOC) metabolic network in activated T cells, rapidly funneling serine into one-carbon metabolism, a process essential for T_N_ and T_EFF_ cell expansion^69,70^. Consistent with this, our experimental results revealed that PHGDH-IN-3 exerted minimal effects on CD8⁺ T cells isolated from the PB of PPD+ volunteers in the absence of E-C fusion antigen stimulation. These findings indicate that serine plays a dual, context-dependent role in chronic infections such as TB. While serine supports T_N_ and T_EFF_ expansion during early phases, SIRPγ upregulates PHGDH expression, amplifying serine metabolism. This elevated serine metabolism contributes to SAM production, which is integral to establishing key exhaustion features, including the high expression of inhibitory receptors, along with impaired cytokine secretion. In the context of TB, serine metabolism must be finely balanced to sustain the proliferative capacity of antigen-specific T cells while limiting their effector functions under prolonged antigen exposure. It is noteworthy that glycine, serine, and threonine metabolism are metabolically interconnected, with glycine and serine interconverted via one-carbon metabolism. While our stepwise screening identified PHGDH-mediated serine metabolism as a key node downstream of SIRPγ, the potential contribution of threonine metabolism to CD8⁺ T cell exhaustion cannot be excluded and warrants further investigation.

Serine-derived SAM, as the principal methyl donor for histone methyltransferases, has been implicated in shaping T cell fate decisions. The present study identifies a previously unrecognized mechanism by which SIRPγ signaling mediates CD8⁺ T cell exhaustion through serine metabolism-dependent SAM production. Integrated ATAC-seq and CUT&Tag analyses revealed that SAM treatment significantly enhanced H3K4me3 enrichment at the NFATC2 promoter region, thereby poising this master regulator for rapid transcriptional activation. Further investigation demonstrated that NFATC2 serves as a key transcriptional regulator of multiple immunosuppressive receptors (TIGIT, CTLA4, HAVCR2, CD38, LAG3) and exhaustion-associated transcription factors (IRF4, TOX). Importantly, inhibition experiments confirmed that NFATC2 is necessary for SAM-driven exhaustion phenotypes. However, given that NFATC2 activation is classically dependent on calcium influx, epigenetic modification alone is insufficient to fully explain SAM-mediated CD8⁺ T cell exhaustion. Notably, SAM-treated CD8⁺ T cells exhibited significant enrichment of TCR signaling pathways, accompanied by upregulated expression of downstream genes, including *NFAT*, *NFKB1*, and *ZAP70*. This observation suggests that SAM may intersect with additional signaling cascades to facilitate calcium influx, thereby synergizing with epigenetic priming to promote full NFATC2 activation. Consistently, SAM treatment directly enhanced calcium flux and promoted NFATC2 nuclear translocation, even under conditions of weak calcium stimulation. Collectively, these findings suggest that SAM facilitates exhaustion through a dual modality: it establishes an epigenetically permissive state at the NFATC2 locus via H3K4me3 enrichment, while concurrently cooperating with TCR-NFAT signaling to lower the threshold for calcium-dependent NFATC2 activation.

SIRPγ is predominantly expressed on T cells and interacts with its ligand CD47, also known as integrin-associated protein (IAP). At inflammatory sites, CD47-SIRPγ interactions may facilitate T cell transmigration across vascular endothelium into tissues^71^, which may underlie the elevated presence of SIRPγ⁺CD8⁺ T cells in TPE. Furthermore, under chronic stimulation, CD47-SIRPγ binding stabilizes immunological synapses, enhancing TCR-antigen recognition and co-stimulatory signaling to potentiate T cell activation^72^. Our study further demonstrated that SIRPγ engagement with CD47 activated the PI3K/AKT/mTOR signaling cascade, resulting in PHGDH upregulation and enhanced serine metabolism. This metabolic shift subsequently induced NFATC2 expression via SAM-dependent H3K4me3 epigenetic modification, ultimately driving the expression of exhaustion-associated transcription factors and inhibitory receptors in CD8^+^ T cells. CD47, a member of the immunoglobulin superfamily (IgSF), is widely expressed across various cell types, including Treg and CD8⁺ T cells^73^. However, the specific cellular source of CD47 that engages SIRPγ in this context remains unidentified. Given that the PI3K/AKT/mTOR pathway and downstream serine metabolism are induced upon CD47–SIRPγ engagement, we speculate that the relevant interaction likely occurs in trans, for instance between CD8⁺ T cells and Tregs or between CD8⁺ T cells and antigen-presenting cells, where SIRPγ on one cell engages CD47 on a neighboring cell. This trans interaction may facilitate metabolic reprogramming in CD8⁺ T cells, contributing to the exhaustion phenotype observed in TPE. Alternatively, cis interactions on the same CD8⁺ T cell cannot be formally excluded. Distinguishing between these modes will require further investigation using cell-type-specific genetic or blocking approaches.

This study has certain limitations. First, we employed high-throughput antibody microarray technology to identify differential proteins between TPE and MPE, validating SIRPγ as a diagnostic marker for distinguishing TPE from non-TPE. However, further validation using independent external cohorts is necessary to fully assess the diagnostic utility of SIRPγ across diverse clinical settings. Further investigation using spatial and functional assays will be essential to determine the cellular context of CD47–SIRPγ interactions and their role in modulating CD8⁺ T cell behavior in TPE. Moreover, although our study establishes that SIRPγ mediates CD8⁺ T cell exhaustion in TPE, its potential as a therapeutic target remains unvalidated in humanized mice models. Future studies incorporating in vivo evaluations of SIRPγ are crucial for determining the therapeutic potential of targeting SIRPγ in TB, especially TPE.

## Methods

### Patients and samples

All procedures involving human samples adhered to the ethical standards outlined in the Declaration of Helsinki. All participants provided written informed consent. The study protocol was approved by the Wuhan Union Hospital ethics committee and Wuhan Pulmonary Hospital ethics committee (Ethics protocol approval number: 2020-43, 2021-0663, and 2025-0404). Participants were diagnosed with TPE if they met one of the following criteria: (1) a positive Mtb culture from pleural fluid, or (2) histological evidence of granulomatous pleurisy on closed pleural biopsy, provided no other granulomatous diseases were present. TB was diagnosed based on one or more of the following criteria: a positive Ziehl–Neelsen stain for acid-fast bacilli in sputum or pleural fluid; isolation of Mtb from sputum specimens by positive culture on Löwenstein–Jensen medium; or confirmation by histopathological examination. The diagnosis of MPE was confirmed by the cytopathological identification of malignant cells in either pleural fluid or a closed pleural biopsy specimen. Pleural effusion samples and PB were collected from the Wuhan Institute of Tuberculosis Prevention and Control, and the Union Hospital, Tongji Medical College, for experimental purposes. To avoid potential confounding effects of anti-tuberculosis therapy on immune parameters, only treatment-naïve TPE patients were enrolled in this study. Clinicopathological data are summarized in Table 1. In addition, 88 pleural effusion supernatants were obtained from the Union Hospital, Tongji Medical College, to serve as a training set (44 TPE and 40 MPE). A separate validation set consisting of 144 pleural effusion supernatant samples was also acquired from the Wuhan Institute of Tuberculosis Prevention and Control, encompassing cases of 66 TPE, 35 MPE, 27 PPE, and 16 miscellaneous. These samples were employed to evaluate the diagnostic value of SIRPγ. Corresponding clinicopathological data are presented in Supplementary Table S3. Furthermore, given that PPD^+^ healthy donors exhibit more robust Mtb-specific immune memory responsiveness, blood samples were also collected from 10 PPD + healthy volunteers (2 males and 8 females, aged between 20 and 40 years).

**Table 1:** Clinicopathological features of 60 TPE patients and the infiltration levels of SIRPγ^+^CD8^+^T cells

Parameters	Group	Cases	SIRPγ^+^CD8^+^T cells high	SIRPγ^+^CD8^+^T cells low	*P*- value
**Sex**	Male	17	11	6	0.4518
	Female	43	32	11	
**Age**		60	58.24 ± 22.11	56.83 ± 20.33	0.800
**Smoke**	Yes	18	8	10	0.5731
	No	42	22	20	
**Pulmonary disease**	Yes	23	13	10	0.6963
	No	37	19	18	
**TPE volume**	Low	19	9	10	0.8518
	Medium	21	12	9	
	High	20	10	10	
**AFB**	Positive	22	16	6	0.0063
	Negative	38	12	24	
**T-spot**	Positive	47	30	17	0.5123
	Negative	13	7	6	
**Xpert MTB/RIF**	Positive	30	18	12	0.3015
	Negative	30	13	17	
**ADA (U/L)**		60	46.41 ± 21.18	36.80 ± 20.40	0.0787
**LDH (U/L)**		60	491.9 ± 278.3	451.9 ± 338.5	0.6220
**Glucose (mmol/L)**		60	7.120 ± 3.980	7.501 ± 3.200	0.6846
**Protein (g/L)**		60	45.59 ± 6.523	44.03 ± 6.876	0.3842
**Cell counts (cells/mm3)**		60	2251 ± 1649	3386 ± 2157	0.0527
**Lymphocyte (%)**		60	72.34 ± 25.18	70.21 ± 20.88	0.7437
**Total**		60	30	30	

*P* < 0.05 represents statistical significance; Comparisons of data between SIRPγ^+^CD8^+^T cells high and SIRPγ^+^CD8^+^T cells low groups were performed using an unpaired two-tailed *t* test (age, ADA, LDH, glucose, protein, cell counts, lymphocyte), two-sided Fisher’s exact test (Xpert MTB/RIF, AFB), Fisher’s exact test (volume), and two-sided Chi-square test (T-spot). Values are shown as mean ± SEM. TPE, tuberculous pleural effusion; LDH, lactate dehydrogenase; ADA, adenosine deaminase; AFB, acid-fast bacillus; T-spot, T-cell spot test for tuberculosis; Xpert MTB/RIF, Xpert Mycobacterium tuberculosis/Rifampicin.

### Sample collection and processing

Following collection via standard thoracentesis from TPE patients (within 24 h of hospitalization) and peripheral blood draw (10 mL from TPE/TB patients), all specimens were immediately placed on ice. After centrifugation (1500 × *g*, 6 min), the supernatants (from pleural effusions) and serum were aliquoted and stored at − 80 °C. Mononuclear cells were then isolated from all specimens, resuspended in 1×phosphate-buffered saline (PBS, Servicebio, Wuhan, China, # G4202) using a Lymphoprep™ (StemCell Technologies, Vancouver, Canada, # 18061).

### Cell culture

PBMCs were collected from three donor groups: PPD^+^ volunteers, patients with TPE, and those with active pulmonary TB. In addition, pleural effusion-derived mononuclear cells (PEMCs) were obtained from TPE and MPE patients. All cells were maintained in RPMI-1640 medium (Gibco, # 11875119) containing 10% Fetal Bovine Serum (Gibco, # A5670701), penicillin-streptomycin (100 μ/mL and 100 μg/mL), 2 mM L-glutamine, and 10 mM HEPES. For time-course experiments, cells were plated at 2 × 10⁵ cells/mL in flat-bottom 48-well plates, with 0.5 mL per well. Where applicable, PBMCs from PPD-positive volunteers and TPE patients were stimulated with E-C (10 μg/mL) at 37 °C for 20 h, after which the antigen-containing medium was removed, and cells were cultured for special hours before analysis. The recombinant fusion antigen E-C, derived from the RD1 region of the Mtb genome (which is specific for Mtb infection and absent in BCG), was used to stimulate antigen-specific T cell responses. The recombinant fusion protein was provided by the State Key Laboratory of Agricultural Microbiology at Huazhong Agricultural University in Wuhan, China.

Human embryonic kidney (HEK) 293 T cells (American Type Culture Collection, # AT-1067) were cultured in RPMI-1640 medium (Gibco, # 11875119) containing 10% Fetal Bovine Serum (Gibco, # A5670701), penicillin-streptomycin (100 μ/mL and 100 μg/mL), 2 mM L-glutamine, and 10 mM HEPES.

### Flow cytometry and cell sorting

PBMCs and PEMCs were isolated from patients with TPE or MPE. Immune cells were resuspended in staining buffer and incubated with Fc receptor blocking reagent (BioLegend, # 422302) for 10 min. Surface markers were then stained with fluorochrome-conjugated antibodies (30 min, 4 °C, dark). Next, cells were fixed and permeabilized using Fixation/Permeabilization solution (eBioscience, # 00-5521-00; 1:3 concentrate: diluent ratio for 30 minutes at 4 °C in darkness, and washed twice with 1 × permeabilization buffer. Intracellular staining was then performed using specific antibodies under the same conditions (30 min, 4 °C, dark). Cells were finally fixed in stabilization buffer and acquired on a BD flow cytometer. For cytokine detection in CD8⁺ T cells, samples were pre-stimulated with Cell Activation Cocktail (BioLegend, # 423304) for 4–6 h prior to staining. Data analysis was performed using FlowJo software (BD Biosciences). For the identification of SIRPγ-expressing subsets, cells were stained with an anti-human SIRPγ antibody (clone LSB2.20, BioLegend, PE-conjugated). After excluding doublets and dead cells, immune cells were gated based on forward/side scatter. SIRPγ expression was then analyzed on macrophages (CD45⁺CD3⁻CD68⁺), B cells (CD45⁺CD3⁻CD19⁺), NK cells (CD45⁺CD3⁻CD56⁺), and CD8⁺ T cells (CD45⁺CD3⁺CD8⁺). The cutoff for positivity was defined using isotype control background. Gating strategy is provided in Supplementary Fig. S16, and the panels were listed in Supplementary Table S5.

CD8⁺ T cells were isolated from TPE via magnetic-activated cell sorting (MACS) with a human CD8⁺ T Cell Isolation Kit (STEMCELL Technologies, # 17953). Subsequently, the purified CD8⁺ T cells were further sorted on a BD FACS Aria II system into SIRPγ⁻ and SIRPγ⁺ subsets by fluorescence-activated cell sorting (FACS). All antibodies and reagents are cataloged in Supplementary Table S4.

### RNA extraction, real-time quantitative PCR (RT-qRCR)

Total RNA was extracted from cells using TRIzol reagent (Invitrogen, # 15596018CN), and concentrations were determined with a NanoDrop spectrophotometer (NanoDrop Technologies). The purified RNA was then reverse-transcribed into cDNA using HiScript qRT SuperMix (Vazyme, Nanjing, China, # R223-01). Quantitative real-time PCR (qPCR) was then carried out on an ABI 7500 Real-Time PCR System using 2 × TSINGKE® Master qPCR Mix (Tsingke Biotech, #TSE201). Gene expression was normalized to *GAPDH* and calculated using the comparative 2^–ΔΔCT^ method. All primer sequences can be found in Supplementary Table S6 and were purchased from Sangon Biotech (Shanghai, China).

### Western blotting

CD8⁺ T cells isolated from TPE were lysed on ice for 30 minutes using radioimmunoprecipitation assay (RIPA) buffer containing protease and phosphatase inhibitor cocktails. After centrifugation (12,000 × *g* for 15 min, 4 °C), the supernatants were collected as soluble protein extracts. Protein concentrations were quantified using a BCA assay kit (Beyotime Biotechnology, Shanghai, China, #P0011). Equal protein aliquots (20 μg/lane) were separated by sodium dodecyl sulfate-polyacrylamide gel electrophoresis (SDS-PAGE) and electrophoretically transferred to polyvinylidene fluoride (PVDF) membranes (Millipore) at 100 V for 60 min (4 °C). After blocking with 5% non-fat milk in TBST (Tris-buffered saline with 0.05% Tween-20) for 1.5 h at room temperature (RT), the membranes were incubated with primary antibodies overnight at 4 °C. After three 8 min TBST washes (RT), membranes were incubated with horseradish peroxidase (HRP)-conjugated secondary antibodies (1 h, RT). Chemiluminescent substrate (Servicebio, Wuhan, China, # G2014) was used to detect protein bands, which were imaged using a UVP ChemiDoc system (Upland, CA, USA). Complete antibody specifications are provided in Supplementary Table S4.

### Measurement of SIRPγ, serine and SAM concentrations

The concentrations of SIRPγ in PE were measured using enzyme-linked immunosorbent assay (ELISA) kits (GenMed, Wuhan, China). L-Serine concentrations in lysates of SIRPγ^+/-^CD8^+^ T cells under various treatment conditions were quantified using a human L-serine ELISA kit (Camilo, Nanjing, China, # 2H-KMLJh315544). Similarly, the SAM concentrations in CD8^+^T cells were detected using a General SAM ELISA Kit (ABclonal, Wuhan, China, # RK04519). All samples were analyzed in triplicate.

### Proliferation assay

CD8⁺ T cells isolated from PBMCs and PEMCs of patients with TPE were pre-stimulated with anti-CD28 (5 μg/mL) and anti-CD3 (5 μg/mL) antibodies (BioLegend). For proliferation analysis, cells were labeled with carboxyfluorescein succinimidyl ester (CFSE; BD Biosciences, # 565082) at a 1:1000 dilution. Following 72 h of culture under experimental conditions, CD8⁺ T cells were harvested, and proliferation was assessed based on CFSE dilution profiles via flow cytometry.

### Histology

Pleural tissue specimens, including tuberculous lesions and adjacent normal pleural tissues, were collected from 14 patients with TPE. Tissues were fixed overnight in 4% paraformaldehyde, embedded in paraffin, and sectioned into 4-µm-thick slices. For histological analysis, sections were stained with hematoxylin and eosin (H&E) according to standard protocols. Immunohistochemical (IHC) staining was performed to detect SIRPγ expression. Briefly, paraffin-embedded sections were deparaffinized, rehydrated, and subjected to heat-induced antigen retrieval. Endogenous peroxidase activity was blocked with 3% H₂O₂, and non-specific binding was blocked with 1% bovine serum albumin (BSA) for 30 min at RT. Sections were then incubated overnight at 4 °C with primary antibodies against SIRPγ. After washing with PBS, sections were incubated with HRP-conjugated secondary antibodies for 1 h at RT, followed by streptavidin-peroxidase solution for 15 min. The immunoreaction was visualized using 3,3’-diaminobenzidine (DAB) substrate, and sections were counterstained with hematoxylin to visualize nuclei.

Quantitative analysis of SIRPγ-positive cells was performed on stained sections. Stained sections were evaluated independently by two investigators blinded to the clinical data. For each sample, 10 random high-power fields (400× magnification; each field area = 0.237 mm²) were selected per section, and the number of SIRPγ^+^ cells was counted. The mean cell count per mm² was calculated for each sample. Inter-observer variability was assessed, and discrepancies were resolved by consensus to ensure data reliability.

### Immunofluorescence staining

Immunofluorescence (IF) staining was employed to detect and localize specific intracellular proteins. The experimental workflow was as follows: PEMCs from TPE patients seeded into 24-well culture plates containing cell-crawling coverslips. CD8⁺ T cells were further isolated from TPE and sorted via flow cytometry into SIRPγ⁻ and SIRPγ⁺CD8⁺ T cells. These cells were evenly distributed on glass slides at appropriate densities. Cells were first fixed with 4% paraformaldehyde for 15 minutes and then washed with PBS. Permeabilization was conducted using 2 mL of 0.2–0.5% Triton X-100 (diluted in PBS) for 10 min, followed by blocking with 2% BSA for 30 minutes. After three washes, slides were incubated with primary antibodies overnight at 4 °C. Following further washes, secondary antibodies were applied and incubated (37 °C for 1 h). Following another round of PBS washes, nuclei were counterstained with 4’,6-diamidino-2-phenylindole (DAPI; Sigma-Aldrich, St. Louis, MO, USA). Imaging analysis was performed using a confocal microscope (Nikon AX; Nikon, Tokyo, Japan).

### LC-MS analysis of metabolites

For U-[^13^C]-glucose labeling, SIRPγ^+^ and SIRPγ^-^CD8^+^T cells (5 × 10^6^) from TPE were cultured in no glucose 1640 containing 10% dialyzed FBS and U-[^13^C]-glucose (15 mM) (Cambridge Isotope Laboratories Inc., catalog CLM-1396-10) for 6 h, the cells were then washed with cold PBS, and metabolites were extracted using cold 80% methanol. LC-MS was performed by LipidALL Technologies (Changzhou, China), and metabolite abundance was expressed relative to the internal standard according to standard protocols. To correct for inter-sample variations in ionization efficiency, L-Leucine-d10 was used as an internal standard for normalization of peak areas across samples.

### The high-throughput cytokine array screen

Pleural effusion protein profiling was performed using the G-Series Human Cytokine Antibody Array X00 (RayBiotech, Guangzhou, China). All effusion samples were clarified by centrifugation prior to array analysis. Sample processing occurred in two batches. Batch 1 included 8 TPE and 8 MPE samples screened for 1000 pleural effusion proteins. Batch 2 contained 50 TPE and 50 MPE samples analyzed for 32 target proteins. Each protein-specific capture antibody was printed in quadruplicate on glass slides. For hybridization, each well received 100 µL of sample dilution buffer and was incubated with shaking (1 h, RT). After buffer removal, 70 µL of undiluted sample was added per well and incubated overnight at 4 °C. Following washing, 80 µL of pre-diluted detection antibody mixture was added per well and incubated for 2 h at RT with shaking. Post-wash, each well received 80 µL of pre-diluted Cy3-conjugated streptavidin and incubated with shaking under aluminum foil protection (1 h, RT). After final washing, fluorescence signals were acquired using an InnoScan 300 scanner (Cy3/532 nm channel, 10 µm resolution). Data analysis was conducted with GSH-CAA-X00 software.

### Chromatin immunoprecipitation

Isolation of CD8⁺ T cells from TPE was performed using MACS, yielding 1 × 10⁷ cells. Subsequently, chromatin immunoprecipitation (ChIP) assays were conducted on these cells as described below, using an Enzymatic ChIP Kit (Cell Signaling Technology, # 9003) in strict accordance with the provided protocol. Anti-NFATC2 antibody (1:1000 dilution, Abcam) was used in the assays, with normal IgG serving as the control. qPCR was subsequently conducted to validate genes containing NFATC2 binding elements. Binding sites of NFATC2 within the promoter regions of inhibitory receptors (*CTLA4*, *HAVCR2*, *PDCD1*, *TIGIT*) and transcription factors (*TOX*, *IRF4*) were predicted using the JASPAR database (http://jaspar.genereg.net). Primers were synthesized by Sangon Biotech (Shanghai, China), and their sequences are detailed in Supplementary Table S7.

### Luciferase reporter assay

Luciferase activity was performed using the dual luciferase reporter system (Promega, Madison, Wisconsin, USA). To construct firefly luciferase reporter genes, mRNA sequences of inhibitory receptors (*CTLA4*, *HAVCR2*, *PDCD1*, *TIGIT*) and transcription factors (*TOX*, *IRF4*) were cloned into the pMIR-REPORT vector by NovoPro (Shanghai, China). For luciferase activity assessment, 293 T cells were co-transfected with the resulting firefly luciferase reporter plasmids and a Renilla luciferase vector to serve as an internal control. Additional transfections were carried out using Lipofectamine 3000 to introduce firefly luciferase reporters together with *NFATC2* overexpression plasmids (Vigene Biosciences, Shandong, China) into 293 T cells. Luminescence readings were obtained using the dual-luciferase assay kit (Vazyme, Nanjing, China, # DL101-01).

### Single-cell RNA sequencing (scRNA-seq) analysis

ScRNA-seq was conducted on SIRPγ⁻CD8⁺ and SIRPγ⁺CD8⁺ T cells utilizing publicly available datasets (Project IDs: HRA000910 and HRA000363). These datasets originated from PBMCs and PFMCs obtained from four treatment-naïve patients with TPE. Furthermore, the published single-cell RNA sequencing dataset of tuberculous-infected human lung (Project IDs: GSE192483) was employed to examine the expression distribution of *SIRPG*. Raw sequencing data were processed in accordance with standard workflows to remove low-quality cells. Initial bioinformatic analysis was performed with the Seurat R package (version 5.2.1). The data were normalized using a scale factor of 10,000, and the top 2000 highly variable genes were selected with the FindVariableFeatures function for subsequent dimensionality reduction through principal component analysis (PCA). The first 40 principal components were then employed as input for the FindNeighbors algorithm. Cell clusters were identified using the FindClusters function and visualized via t-distributed stochastic neighbor embedding (t-SNE). Differentially expressed genes (DEGs) across clusters were detected using the FindMarkers function.

### RNA-seq analysis

RNA sequencing was conducted on RNA isolated from flow-sorted SIRPγ⁻CD8⁺ and SIRPγ⁺CD8⁺ T cells. First, quality-controlled RNA was reverse-transcribed into cDNA and amplified via PCR. Next, these samples were prepared into sequencing libraries. The libraries were sequenced on an Illumina PE150 platform, and raw reads were processed with fastp (v0.23.2) to generate high-quality clean data. Clean reads were aligned to the human reference genome (GRCh38) using HISAT2 (Hierarchical Indexing for Spliced Alignment of Transcripts). Differential gene expression analysis was conducted with DESeq2 (v1.42.0), applying a threshold of |log2FC | > 1 and *p* < 0.05 for identifying DEGs. Gene set enrichment analysis (GSEA) demonstrated significantly upregulated key metabolic pathways in SIRPγ⁺CD8⁺ T cells. Single-gene GSEA focused on *SIRPG* further supported a strong correlation between these metabolic pathways and *SIRPG* expression. Integration of these analyses highlighted the glycine, serine, and threonine metabolism pathway. To identify pathway-specific DEGs, an additional intersection analysis was performed using genes associated with this metabolic pathway.

### CUT&Tag sequencing library preparation

CD8⁺ T cells were collected from the TPE of three patients, sorted, and allocated to either the control or the SAM treatment groups. To reduce biological variability, cells from triplicate samples were pooled prior to analysis. For each assay, approximately 5 × 10^5^ CD8⁺ T cells were pelleted by centrifugation at 600 × *g* for 3 min at RT. Cells were first bound to concanavalin A-coated magnetic beads and permeabilized with digitonin (0.01% in wash buffer). Subsequently, an anti-H3K4me3 antibody was applied and incubated with the cells (2 h, RT). The cells were then bound to concanavalin A–conjugated magnetic beads and permeabilized using digitonin (0.01% in wash buffer). Subsequently, an anti-H3K4me3 antibody was applied and incubated with the cells (2 h, RT). Protein G–Tn5 (pG-Tn5) transposase complexes were then added to leverage Protein G–antibody binding for targeted Tn5 recruitment. Transposase-mediated fragmentation was triggered by the addition of 10 mM Mg²⁺, releasing antibody-bound DNA fragments. The cleaved DNA was purified, PCR-amplified, and size-selected using AMPure XP beads (Beckman Colter). Final libraries were sequenced on an Illumina Nova-Seq platform.

### ATAC-seq sequencing library preparation

CD8⁺ T cells were collected from the TPE of three patients, sorted, and allocated to either the control or the SAM treatment groups. Following isolation, the three samples were pooled into a single composite sample. For each condition, approximately 5 × 10^5^ CD8⁺ T cells were used to prepare cell suspensions. Cellular membranes were then lysed to extract nuclei, and the DNA was subsequently fragmented using Tn5 transposase. The resulting DNA fragments were amplified via PCR and used to construct sequencing libraries on the Illumina PE150 platform.

### ATAC and CUT & Tag data processing, and differential peak accessibility analysis

Sequencing-derived raw image files were processed through base calling to generate raw reads, which were subsequently output in the standard FASTQ format. Following adapter trimming and low-quality read filtering, these Raw Reads yielded Clean Reads. Clean data were aligned to the hg38 reference genome using Bowtie2. Subsequent processing with Samtools and Picard removed low-quality alignments, PCR duplicates, and organelle-mapped reads, generating final high-confidence data for downstream analysis. This curated dataset was subjected to peak calling using MACS2 to identify significantly enriched regions (peaks), followed by differential peak accessibility analysis. We then employed Deeptools to visualize enrichment intensities across peaks, transcription start sites (TSS), and gene body regions. Peak annotation was conducted with ChIPseeker, followed by pathway analysis and motif enrichment examination of the most dynamically altered peaks across conditions. All identified peaks were visually inspected using the Integrative Genomics Viewer (IGV).

### Statistics

Statistical analyses were conducted using Prism v10 (GraphPad Software. Appropriate statistical methods, including two-tailed *t* tests, one-way ANOVA, two-way ANOVA, and Pearson correlation, were applied based on the experimental design and data structure to determine significance. The normality of the data distributions for SIRPγ, LDH, and ADA was confirmed using appropriate normality tests; consequently, parametric statistical methods were employed. All data are expressed as mean ± standard deviation (SD). To evaluate the diagnostic performance of SIRPγ, ADA, SIRPγ × ADA in discriminating between TPE and non-TPE, receiver operating characteristic (ROC) curve analyses were conducted. A *p*-value less than 0.05 was considered statistically significant.

### Reporting summary

Further information on research design is available in the Nature Portfolio Reporting Summary linked to this article.

## Supplementary information

Supplementary Information

Reporting Summary

Transparent Peer Review file

## Source data

Source Data

## Data Availability

The raw RNA-seq, ATAC-seq, and CUT&Tag data generated in this study have been deposited in the NCBI Sequence Read Archive under accession codes PRJNA1440653 (RNA-seq) and PRJNA1440899 (ATAC-seq and CUT&Tag). Direct links to these datasets are available at: https://www.ncbi.nlm.nih.gov/bioproject/PRJNA1440653/ and https://www.ncbi.nlm.nih.gov/bioproject/?term=PRJNA1440899. All data needed to evaluate the conclusions are present in the article, the supplementary materials and the source data file provided with this paper. Further information and requests for resources and reagents should be directed to and will be fulfilled by the corresponding author. Source data are provided in this paper.

## References

[1] Shaw, J. A. & Koegelenberg, C. F. N. Pleural Tuberculosis. *Clin. Chest Med.* **42**, 649–666 (2021). 10.1016/j.ccm.2021.08.002 34774172

[2] Chan, K. K. P. & Lee, Y. C. G. Tuberculous pleuritis: clinical presentations and diagnostic challenges. *Curr. Opin. Pulm. Med.* **30**, 210–216 (2024). 10.1097/MCP.0000000000001052 PMC10990028 38323466

[3] Shaw, J. A., Diacon, A. H. & Koegelenberg, C. F. N. Tuberculous pleural effusion. *Respirology* **24**, 962–971 (2019). 10.1111/resp.13673 31418985

[4] Light, R. W. Update on tuberculous pleural effusion. *Respirology* **15**, 451–458 (2010). 10.1111/j.1440-1843.2010.01723.x 20345583

[5] Aggarwal, A. N. et al. Pleural fluid interleukins for diagnosis of tuberculous pleural effusion: A systematic review and meta-analysis. *Cytokine* **159**, 156019 (2022). 10.1016/j.cyto.2022.156019 36054961

[6] Shu, C. C. et al. Diagnostic role of inflammatory and anti-inflammatory cytokines and effector molecules of cytotoxic T lymphocytes in tuberculous pleural effusion. *Respirology* **20**, 147–154 (2015). 10.1111/resp.12414 25355638

[7] Darrah, P. A. et al. Prevention of tuberculosis in macaques after intravenous BCG immunization. *Nature* **577**, 95–102 (2020). 10.1038/s41586-019-1817-8 PMC7015856 31894150

[8] Mogues, T., Goodrich, M. E., Ryan, L., LaCourse, R. & North, R. J. The relative importance of T cell subsets in immunity and immunopathology of airborne Mycobacterium tuberculosis infection in mice. *J. Exp. Med.* **193**, 271–280 (2001). 10.1084/jem.193.3.271 PMC2195922 11157048

[9] Winchell, C. G. et al. CD8+ lymphocytes are critical for early control of tuberculosis in macaques. *J. Exp. Med.* **220** 10.1084/jem.20230707 (2023). PMC10579699 37843832

[10] Lin, P. L. & Flynn, J. L. CD8 T cells and Mycobacterium tuberculosis infection. *Semin Immunopathol.* **37**, 239–249 (2015). 10.1007/s00281-015-0490-8 PMC4439333 25917388

[11] Chen, J. et al. The elevated expression of LAG-3 on CD8+T cells correlates with disease severity of pulmonary TB. *Micro. Pathog.* **179**, 106089 (2023). 10.1016/j.micpath.2023.106089 37004963

[12] Kalokhe, A. S. et al. Impaired degranulation and proliferative capacity of Mycobacterium tuberculosis-specific CD8+ T cells in HIV-infected individuals with latent tuberculosis. *J. Infect. Dis.* **211**, 635–640 (2015). 10.1093/infdis/jiu505 PMC4351361 25205634

[13] Pan, Y. et al. Overexpression of LAG-3: a potential indicator of low immune function in tuberculosis. *Front. Cell Infect. Microbiol.* **14**, 1410015 (2024). 10.3389/fcimb.2024.1410015 PMC11217189 38957797

[14] Suarez, G. V. et al. PD-1/PD-L1 Pathway modulates macrophage susceptibility to mycobacterium tuberculosis specific CD8(+) T cell induced death. *Sci. Rep.* **9**, 187 (2019). 10.1038/s41598-018-36403-2 PMC6336852 30655556

[15] Wang, X. et al. Elevated expression of Tim-3 on CD8 T cells correlates with disease severity of pulmonary tuberculosis. *J. Infect.* **62**, 292–300 (2011). 10.1016/j.jinf.2011.02.013 21382414

[16] Zhou, J. et al. TIGIT blockade improves anti-Mycobacterium tuberculosis immunity. *PLoS Pathog.* **21**, e1013228 (2025). 10.1371/journal.ppat.1013228 PMC12173411 40526725

[17] Hatherley, D. et al. Paired receptor specificity explained by structures of signal regulatory proteins alone and complexed with CD47. *Mol. Cell* **31**, 266–277 (2008). 10.1016/j.molcel.2008.05.026 18657508

[18] Brooke, G., Holbrook, J. D., Brown, M. H. & Barclay, A. N. Human lymphocytes interact directly with CD47 through a novel member of the signal regulatory protein (SIRP) family. *J. Immunol.* **173**, 2562–2570 (2004). 10.4049/jimmunol.173.4.2562 15294972

[19] Piccio, L. et al. Adhesion of human T cells to antigen-presenting cells through SIRPbeta2-CD47 interaction costimulates T-cell proliferation. *Blood* **105**, 2421–2427 (2005). 10.1182/blood-2004-07-2823 15383453

[20] Sinha, S. et al. An autoimmune disease risk SNP, rs2281808, in SIRPG is associated with reduced expression of SIRPgamma and heightened effector state in human CD8 T-cells. *Sci. Rep.* **8**, 15440 (2018). 10.1038/s41598-018-33901-1 PMC6194019 30337675

[21] Sharp, R. C., Brown, M. E., Shapiro, M. R., Posgai, A. L. & Brusko, T. M. The immunoregulatory role of the signal regulatory protein family and CD47 signaling pathway in type 1 diabetes. *Front. Immunol.* **12**, 739048 (2021). 10.3389/fimmu.2021.739048 PMC8481641 34603322

[22] Barclay, A. N. & Brown, M. H. The SIRP family of receptors and immune regulation. *Nat. Rev. Immunol.* **6**, 457–464 (2006). 10.1038/nri1859 16691243

[23] Wang, L. et al. Single-cell RNA-sequencing reveals heterogeneity and intercellular crosstalk in human tuberculosis lung. *J. Infect.* **87**, 373–384 (2023). 10.1016/j.jinf.2023.09.004 37690670

[24] Cai, Y. et al. Single-cell immune profiling reveals functional diversity of T cells in tuberculous pleural effusion. *J. Exp. Med.* **219** 10.1084/jem.20211777 (2022). PMC8789099 35061012

[25] Jayaraman, P. et al. TIM3 Mediates T cell exhaustion during Mycobacterium tuberculosis Infection. *PLoS Pathog.* **12**, e1005490 (2016). 10.1371/journal.ppat.1005490 PMC4788425 26967901

[26] Zou, L. et al. Landscape of T cells in tuberculous pleural effusion. *Clin. Respir. J.* **19**, e70066 (2025). 10.1111/crj.70066 PMC11962214 40170555

[27] Zheng, K., Zheng, X. & Yang, W. The role of metabolic dysfunction in T-cell exhaustion during chronic viral infection. *Front, Immunol.* **13**, 843242 (2022). 10.3389/fimmu.2022.843242 PMC9008220 35432304

[28] Lee, C. M. et al. PHGDH: a novel therapeutic target in cancer. *Exp. Mol. Med.* **56**, 1513–1522 (2024). 10.1038/s12276-024-01268-1 PMC11297271 38945960

[29] Cai, Z. et al. Targeting PHGDH reverses the immunosuppressive phenotype of tumor-associated macrophages through alpha-ketoglutarate and mTORC1 signaling. *Cell Mol. Immunol.* **21**, 448–465 (2024). 10.1038/s41423-024-01134-0 PMC11061172 38409249

[30] Chen, S. et al. Serine supports IL-1beta production in macrophages through mTOR signaling. *Front. Immunol.* **11**, 1866 (2020). 10.3389/fimmu.2020.01866 PMC7481448 32973770

[31] Rodriguez, A. E. et al. Serine metabolism supports macrophage IL-1beta production. *Cell Metab.* **29**, 1003–1011 (2019). 10.1016/j.cmet.2019.01.014 PMC6447453 30773464

[32] Shan, X. et al. Serine metabolism orchestrates macrophage polarization by regulating the IGF1-p38 axis. *Cell Mol. Immunol.* **19**, 1263–1278 (2022). 10.1038/s41423-022-00925-7 PMC9622887 36180780

[33] Rossi, M. et al. PHGDH heterogeneity potentiates cancer cell dissemination and metastasis. *Nature* **605**, 747–753 (2022). 10.1038/s41586-022-04758-2 PMC9888363 35585241

[34] Cheng, H. et al. Mycobacterium tuberculosis produces D-serine under hypoxia to limit CD8(+) T cell-dependent immunity in mice. *Nat. Microbiol.* **9**, 1856–1872 (2024). 10.1038/s41564-024-01701-1 PMC11222154 38806671

[35] Foster, A. C. et al. Phenylglycine analogs are inhibitors of the neutral amino acid transporters ASCT1 and ASCT2 and enhance NMDA receptor-mediated LTP in rat visual cortex slices. *Neuropharmacology* **126**, 70–83 (2017). 10.1016/j.neuropharm.2017.08.010 28807674

[36] Locasale, J. W. Serine, glycine and one-carbon units: cancer metabolism in full circle. *Nat. Rev. Cancer* **13**, 572–583 (2013). 10.1038/nrc3557 PMC3806315 23822983

[37] Pan, S., Fan, M., Liu, Z., Li, X. & Wang, H. Serine, glycine and one‑carbon metabolism in cancer (Review). *Int. J. Oncol.* **58**, 158–170 (2021). 10.3892/ijo.2020.5158 PMC7864012 33491748

[38] Yang, M. & Vousden, K. H. Serine and one-carbon metabolism in cancer. *Nat. Rev. Cancer* **16**, 650–662 (2016). 10.1038/nrc.2016.81 27634448

[39] Ye, J. et al. Serine catabolism regulates mitochondrial redox control during hypoxia. *Cancer Discov.* **4**, 1406–1417 (2014). 10.1158/2159-8290.CD-14-0250 PMC4258153 25186948

[40] Yerinde, C., Siegmund, B., Glauben, R. & Weidinger, C. Metabolic control of epigenetics and its role in CD8(+) T cell differentiation and function. *Front. Immunol.* **10**, 2718 (2019). 10.3389/fimmu.2019.02718 PMC6901948 31849941

[41] Li, T. et al. Methionine deficiency facilitates antitumour immunity by altering m(6)A methylation of immune checkpoint transcripts. *Gut* **72**, 501–511 (2023). 10.1136/gutjnl-2022-326928 PMC9933173 35803704

[42] Pietrocola, F., Galluzzi, L., Bravo-San Pedro, J. M., Madeo, F. & Kroemer, G. Acetyl coenzyme A: a central metabolite and second messenger. *Cell Metab.* **21**, 805–821 (2015). 10.1016/j.cmet.2015.05.014 26039447

[43] Tran, T. Q., Lowman, X. H. & Kong, M. Molecular pathways: metabolic control of histone methylation and gene expression in cancer. *Clin. Cancer Res.* **23**, 4004–4009 (2017). 10.1158/1078-0432.CCR-16-2506 PMC5553983 28404599

[44] Yu, W. et al. One-carbon metabolism supports S-adenosylmethionine and histone methylation to drive inflammatory macrophages. *Mol. Cell* **75**, 1147–1160 (2019). 10.1016/j.molcel.2019.06.039 31420217

[45] Li, S. et al. Serine and SAM responsive complex SESAME regulates histone modification crosstalk by sensing cellular metabolism. *Mol. Cell* **60**, 408–421 (2015). 10.1016/j.molcel.2015.09.024 26527276

[46] Mentch, S. J. et al. Histone methylation dynamics and gene regulation occur through the sensing of one-carbon metabolism. *Cell Metab.* **22**, 861–873 (2015). 10.1016/j.cmet.2015.08.024 PMC4635069 26411344

[47] Hung, M. H. et al. Tumor methionine metabolism drives T-cell exhaustion in hepatocellular carcinoma. *Nat. Commun.* **12**, 1455 (2021). 10.1038/s41467-021-21804-1 PMC7935900 33674593

[48] Philip, M. et al. Chromatin states define tumour-specific T cell dysfunction and reprogramming. *Nature* **545**, 452–456 (2017). 10.1038/nature22367 PMC5693219 28514453

[49] Sen, D. R. et al. The epigenetic landscape of T cell exhaustion. *Science* **354**, 1165–1169 (2016). 10.1126/science.aae0491 PMC5497589 27789799

[50] Hirsch, T. et al. IRF4 impedes human CD8 T cell function and promotes cell proliferation and PD-1 expression. *Cell Rep.* **43**, 114401 (2024). 10.1016/j.celrep.2024.114401 38943641

[51] Huang, Y. J. et al. Continuous expression of TOX safeguards exhausted CD8 T cell epigenetic fate. *Sci. Immunol.* **10**, eado3032 (2025). 10.1126/sciimmunol.ado3032 40053604

[52] Liu, Q. et al. BATF Potentially mediates negative regulation of PD-1/PD-Ls pathway on T cell functions in mycobacterium tuberculosis infection. *Front. Immunol.* **10**, 2430 (2019). 10.3389/fimmu.2019.02430 PMC6803382 31681314

[53] Zhang, X. et al. Depletion of BATF in CAR-T cells enhances antitumor activity by inducing resistance against exhaustion and formation of central memory cells. *Cancer Cell* **40**, 1407–1422 (2022). 10.1016/j.ccell.2022.09.013 36240777

[54] Bian, Y. et al. Cancer SLC43A2 alters T cell methionine metabolism and histone methylation. *Nature* **585**, 277–282 (2020). 10.1038/s41586-020-2682-1 PMC7486248 32879489

[55] Lu, P. et al. Blimp-1 represses CD8 T cell expression of PD-1 using a feed-forward transcriptional circuit during acute viral infection. *J. Exp. Med.* **211**, 515–527 (2014). 10.1084/jem.20130208 PMC3949569 24590765

[56] Martinez, G. J. et al. The transcription factor NFAT promotes exhaustion of activated CD8(+) T cells. *Immunity* **42**, 265–278 (2015). 10.1016/j.immuni.2015.01.006 PMC4346317 25680272

[57] Oestreich, K. J., Yoon, H., Ahmed, R. & Boss, J. M. NFATc1 regulates PD-1 expression upon T cell activation. *J. Immunol.* **181**, 4832–4839 (2008). 10.4049/jimmunol.181.7.4832 PMC2645436 18802087

[58] Khan, O. et al. TOX transcriptionally and epigenetically programs CD8(+) T cell exhaustion. *Nature* **571**, 211–218 (2019). 10.1038/s41586-019-1325-x PMC6713202 31207603

[59] Man, K. et al. Transcription factor IRF4 promotes CD8(+) T cell exhaustion and limits the development of memory-like T cells during chronic infection. Immunity **47**, 1129–1141 (2017). 10.1016/j.immuni.2017.11.021 29246443

[60] Trefny, M. P. et al. Deletion of SNX9 alleviates CD8 T cell exhaustion for effective cellular cancer immunotherapy. *Nat. Commun.* **14**, 86 (2023). 10.1038/s41467-022-35583-w PMC9895440 36732507

[61] Chen, W. L. et al. Differential regulation of calcium-NFAT signaling pathway by Akt isoforms: unraveling effector dynamics and exhaustion of cytotoxic T lymphocytes in tumor microenvironment. *J. Immunother. Cancer* **13** 10.1136/jitc-2024-009827 (2025). PMC11951004 40139836

[62] Wang, Q. et al. Increased expression of PHGDH under high-selenium stress in vivo. *Biol. Trace Elem. Res.* **202**, 5145–5156 (2024). 10.1007/s12011-024-04079-7 38277119

[63] Zhan, S. et al. Activation of the de novo serine synthesis pathway and disruption of insulin signaling induced by supplemental SeMet in vitro. *Biol. Trace Elem. Res.* **203**, 4087–4099 (2025). 10.1007/s12011-024-04492-y 39789350

[64] Zhan, S. et al. The inhibitory effects of NCT503 and exogenous serine on high-selenium induced insulin resistance in mice. *Nutrients* **17** 10.3390/nu17020311 (2025). PMC11767638 39861441

[65] Ikezoe, T. et al. Longitudinal inhibition of PI3K/Akt/mTOR signaling by LY294002 and rapamycin induces growth arrest of adult T-cell leukemia cells. *Leuk. Res.* **31**, 673–682 (2007). 10.1016/j.leukres.2006.08.001 17007924

[66] Li, X. et al. Pleural mesothelial cells promote expansion of IL-17-producing CD8+ T cells in tuberculous pleural effusion. *J. Clin. Immunol.* **33**, 775–787 (2013). 10.1007/s10875-012-9860-3 23299924

[67] Henning, A. N., Roychoudhuri, R. & Restifo, N. P. Epigenetic control of CD8(+) T cell differentiation. *Nat. Rev. Immunol.* **18**, 340–356 (2018). 10.1038/nri.2017.146 PMC6327307 29379213

[68] Pompella, A., Visvikis, A., Paolicchi, A., De Tata, V. & Casini, A. F. The changing faces of glutathione, a cellular protagonist. *Biochem. Pharm.* **66**, 1499–1503 (2003). 10.1016/s0006-2952(03)00504-5 14555227

[69] Ma, E. H. et al. Serine is an essential metabolite for effector T cell expansion. *Cell Metab.* **25**, 345–357 (2017). 10.1016/j.cmet.2016.12.011 28111214

[70] Ma, E. H. et al. Metabolic profiling using stable isotope tracing reveals distinct patterns of glucose utilization by physiologically activated CD8(+) T cells. *Immunity* **51**, 856–870 (2019). 10.1016/j.immuni.2019.09.003 31747582

[71] Stefanidakis, M., Newton, G., Lee, W. Y., Parkos, C. A. & Luscinskas, F. W. Endothelial CD47 interaction with SIRPgamma is required for human T-cell transendothelial migration under shear flow conditions in vitro. *Blood* **112**, 1280–1289 (2008). 10.1182/blood-2008-01-134429 PMC2515120 18524990

[72] Dehmani, S. et al. SIRPgamma-CD47 interaction positively regulates the activation of human T cells in situation of chronic stimulation. *Front. Immunol.* **12**, 732530 (2021). 10.3389/fimmu.2021.732530 PMC8671138 34925315

[73] Lindberg, F. P., Gresham, H. D., Schwarz, E. & Brown, E. J. Molecular cloning of integrin-associated protein: an immunoglobulin family member with multiple membrane-spanning domains implicated in alpha v beta 3-dependent ligand binding. *J. Cell Biol.* **123**, 485–496 (1993). 10.1083/jcb.123.2.485 PMC2119833 7691831

