# Supplementary material for: SIRPγ promotes CD8⁺ T cell exhaustion via metabolic-epigenetic reprogramming in tuberculous pleural effusion
Source: Nat Commun. 2026 Sep 25;17:11144. doi: 10.1038/s41467-026-77779-4 (PMC13710978; doi:10.1038/s41467-026-77779-4)
Supplement: Supplementary file 1 [file 41467_2026_77779_MOESM1_ESM.pdf]

Supplementary Information

SIRPγ Promotes CD8$^+$ T Cell Exhaustion via Metabolic-Epigenetic Reprogramming in Tuberculous Pleural Effusion

Yuening Yang[1, #], Qianqian Xue[1, #], Sidao Wang[1, #], Wenbei Peng[1, #], Linlin Ye[1], Bohan Yang[1], Zihao Wang[1], Xuan Xiang[1], Haolei Wang[1], Yiran, Niu[2], Hai Huang[3, *], Chengqing Yang[3, *], Qiong Zhou[1, *]

[1]Department of Respiratory and Critical Care Medicine, Union Hospital, Tongji Medical College, Huazhong University of Science and Technology, Wuhan, China, 430022

[2]Department of Respiratory and Critical Care Medicine, The Central Hospital of Wuhan, Tongji Medical College, Huazhong University of Science and Technology, Wuhan, 430000, China

[3]Wuhan Pulmonary Hospital, Wuhan Institute for Tuberculosis Control, Wuhan, Hubei, China

This supplementary PDF file includes:

Supplementary Figures 1 to 16.

Supplementary Tables 1 to 7

Supplementary Figure 1

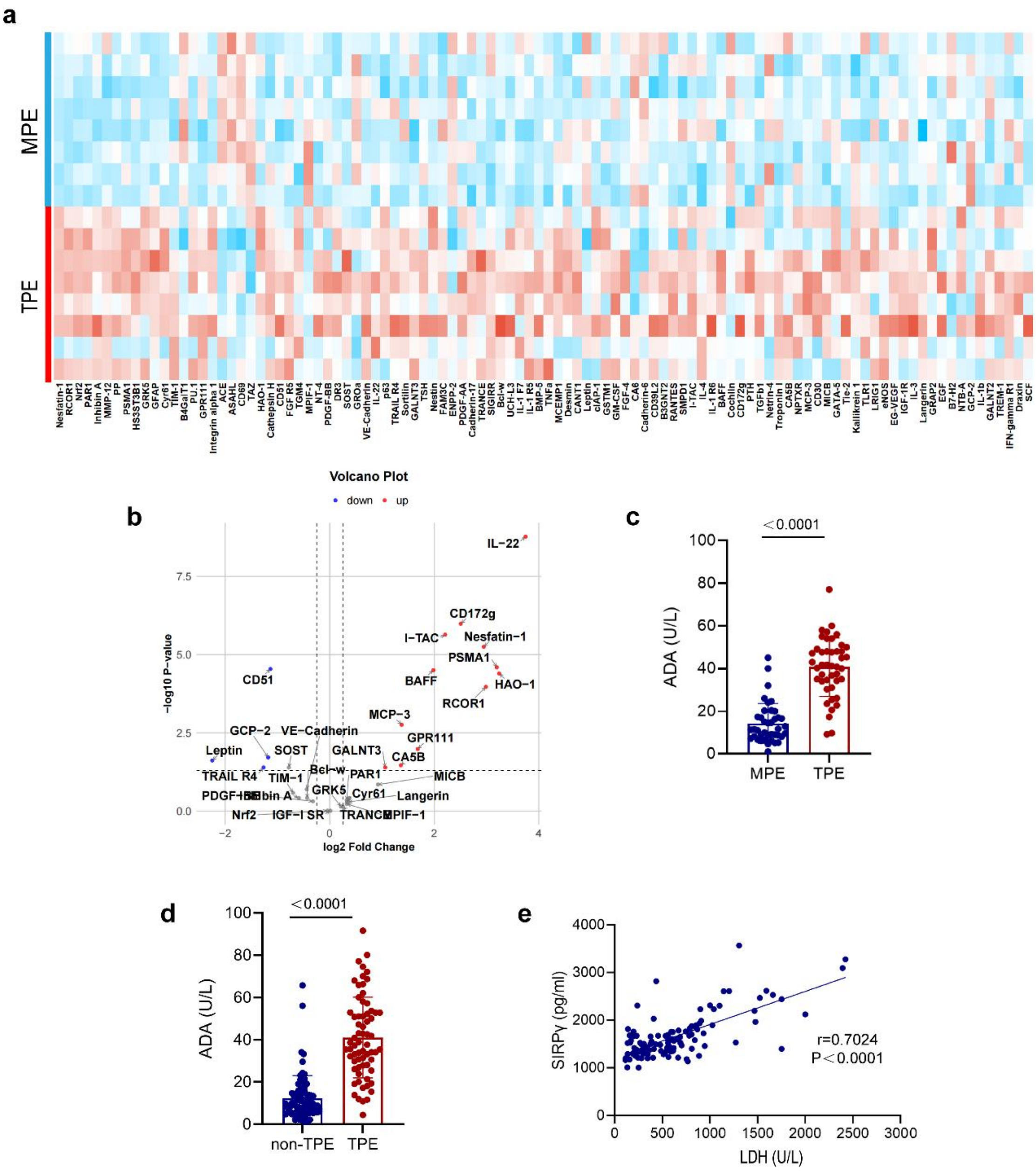

Supplementary Figure 1 SIRPγ serves as a diagnostic biomarker for TPE, related to Figure 1. (**a**) Heatmap depicting protein expression profiles in 16 pleural effusion samples (8 MPE vs. 8 TPE) from the preliminary high-throughput cytokine array analysis. (**b**) Volcano plot identifying 17 differentially expressed proteins in the secondary antibody microarray validation cohort (50 MPE vs. 50 TPE). (**c**) Comparative analysis of ADA levels in TPE (n= 44 patients) versus MPE (n= 40 patients) from the training cohort. (**d**) Comparative analysis of ADA levels in TPE (n = 66 patients) versus non-TPE including MPE (n = 35 patients), parapneumonic pleural

effusion (PPE) (n = 27 patients), and miscellaneous (n = 16 patients) from validation cohort. (**e**) Correlation between pleural fluid SIRPγ levels and LDH concentrations ($Y = 0.6938 \times X + 1216$) (n = 111 patients). Statistical analyses were performed using unpaired two-tailed t-tests (**c**, **d**) and Pearson correlation coefficient (**e**). Data are presented as mean values ± SD (**c**, **d**).

Supplementary Figure 2

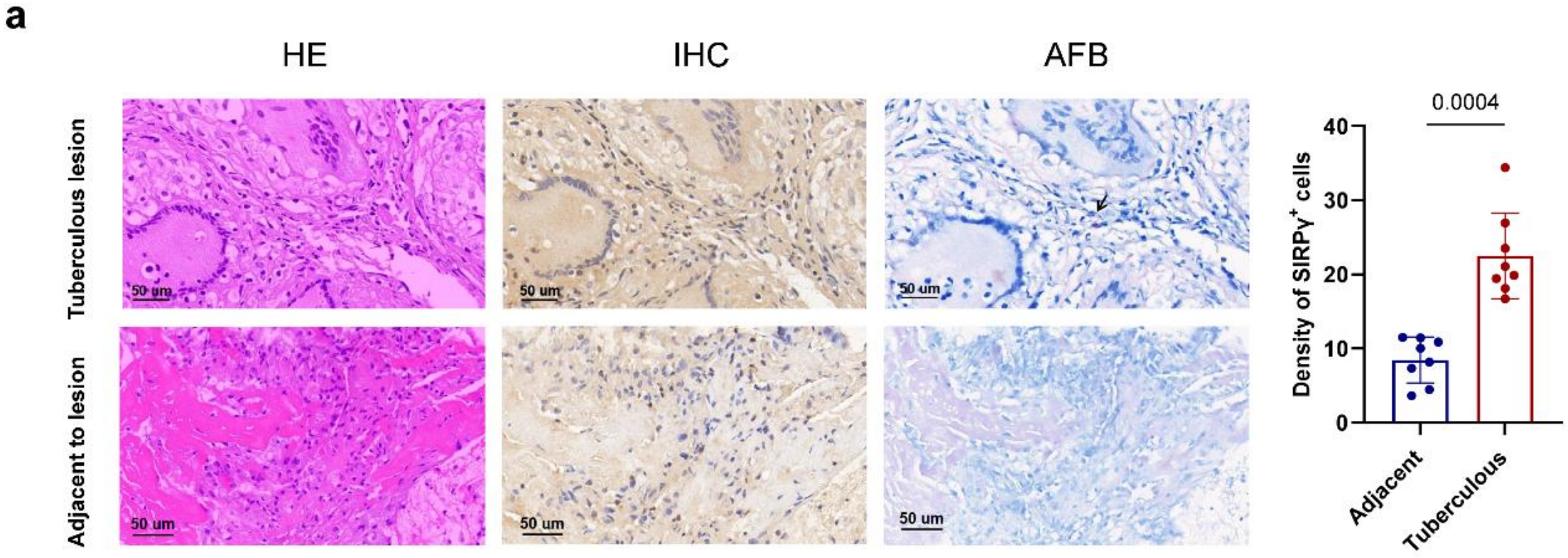

Supplementary Figure 2 SIRPγ serves as a diagnostic biomarker for TPE, related to Figure 1. (**a**) Representative hematoxylin and eosin (H&E), immunohistochemistry (IHC) detection of SIRPγ, and acid-fast bacteria stain (AFB) stains in pleural tissues are displayed with corresponding quantitative results of SIRPγ^{+} cell density (n = 8 patients). Statistical analyses were performed using paired two-tailed t-tests (**a**). Data are presented as mean values ± SD (**a**).

Supplementary Figure 3

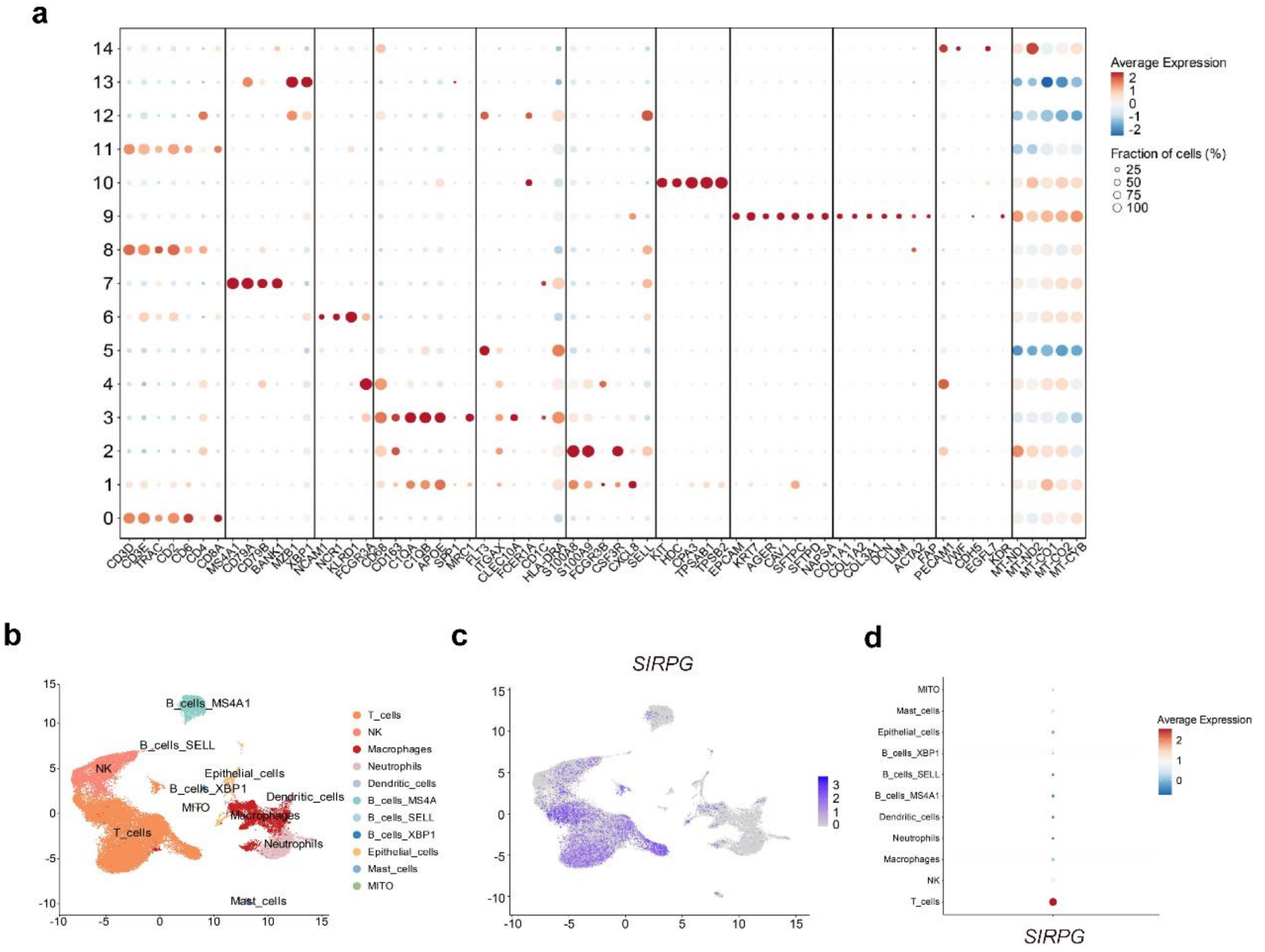

Supplementary Figure 3 SIRPγ is highly expressed in exhausted CD8⁺ T cells in TPE, related to Figure 2. (**a**) Gene expression dot plot showing unique expression patterns to define 11 distinct cell populations in (**b**). (**b**) UMAP representation of cell clusters from combined 5 pulmonary tuberculosis tissue samples. (**c**) UMAP feature plot showing the expression of *SIRPG* of all cells. (**d**) Dot plot showing *SIRPG* expression patterns of the annotated cell populations. NK, natural killer cells; MITO, mitochondrial transcripts.

Supplementary Figure 4

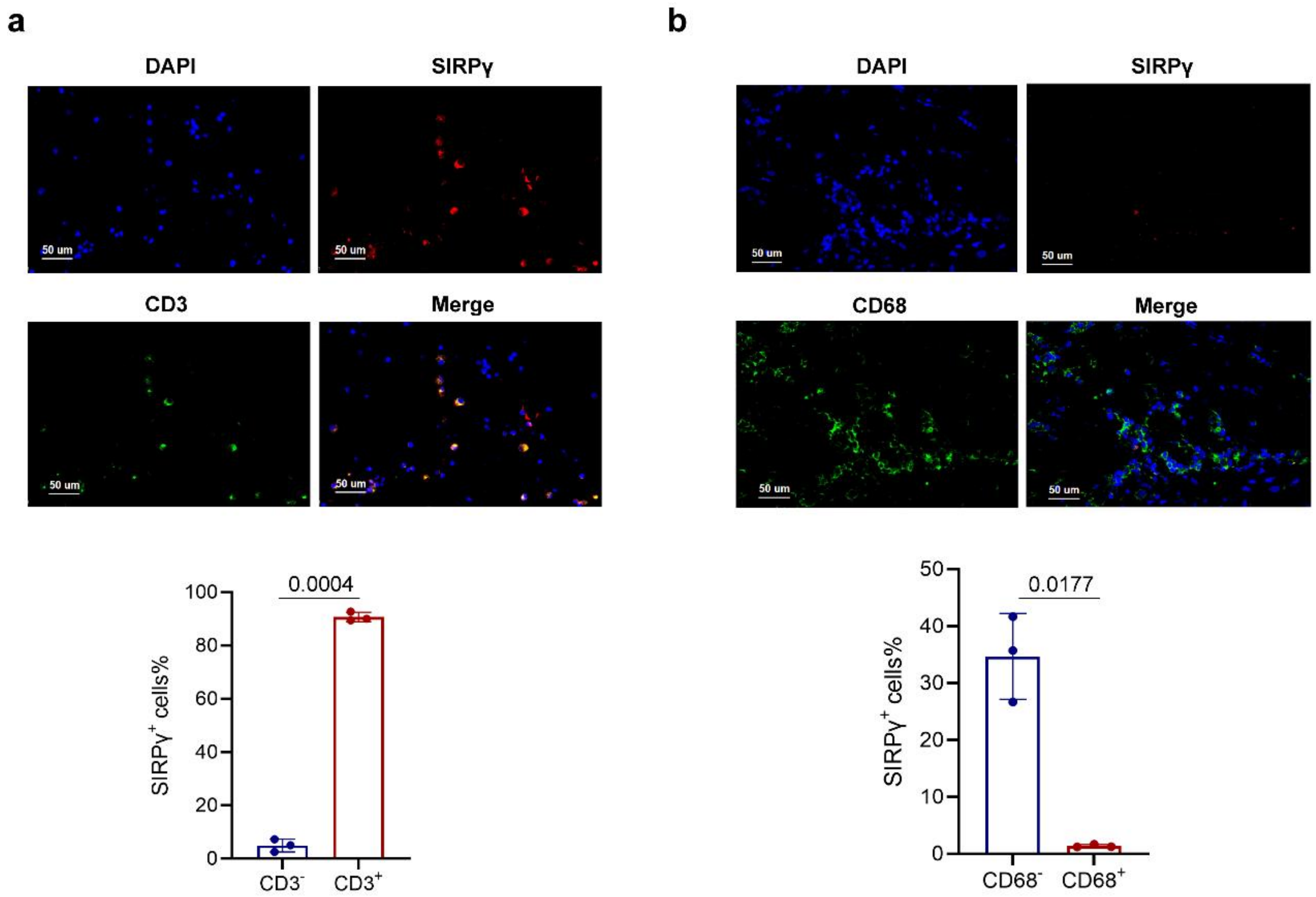

Supplementary Figure 4 SIRPγ is highly expressed in exhausted CD8$^+$ T cells in TPE, related to Figure 2. (**a**) Representative images and quantitative analysis demonstrating the colocalization of SIRPγ and CD3$^+$T cells from tuberculous lesions. Scale bar 50 μm. (**b**) Representative images and quantitative analysis demonstrating the colocalization of SIRPγ and macrophages from tuberculous lesions. Scale bar 50 μm. Statistical significance determined by paired two-tailed t-test (**a**, **b**). Data are presented as mean values ± SD (**a**, **b**).

Supplementary Figure 5

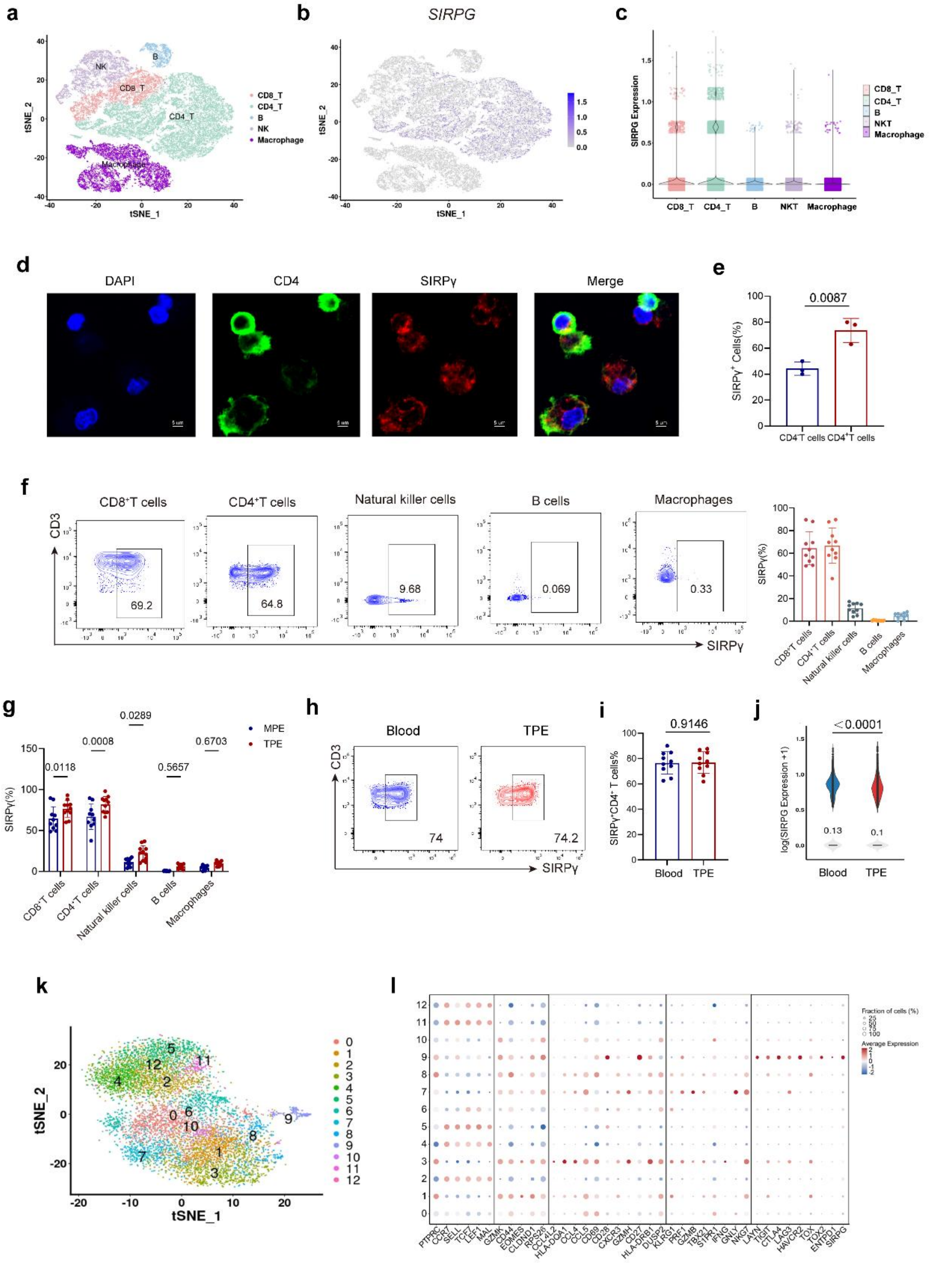

Supplementary Figure 5 SIRPγ is highly expressed in exhausted CD8⁺ T cells in TPE, related to Figure 2. (**a**) t-distributed stochastic neighbor embedding (t-SNE) projection of single-cell RNA sequencing data demonstrates five immune cell clusters in peripheral blood, including B, NK, Macrophage, CD4_T, and CD8_T. (**b**) Expression

levels of the *SIRPG* gene visualized within the t-SNE space. (**c**) Comparative *SIRPG* expression levels across blood immune subsets. (**d**, **e**) Immunofluorescence co-localization of SIRPγ and CD4 in TPE-derived T cells: Representative images (**d**) and quantitative analysis (**e**) (n = 3 independent experiments). Scale bar 5 μm. (**f**) Representative flow cytometric plots and corresponding statistical analyses showing the proportions of $SIRP\gamma^+$ cells from MPE, including $SIRP\gamma^+$ B cells ($CD45^+$ $CD19^+$ $SIRP\gamma^+$), $SIRP\gamma^+$ natural killer cells ($CD45^+$ $CD3^-$ $CD56^+$ $SIRP\gamma^+$), $SIRP\gamma^+$ macrophages ($CD45^+CD68^+SIRP\gamma^+$), $SIRP\gamma^+CD4^+$ T cells ($CD3^+$ $CD4^+SIRP\gamma^+$), and $SIRP\gamma^+CD8^+$ T cells ($CD3^+$ $CD8^+$ $SIRP\gamma^+$) (n = 10 patients). (**g**) Comparative analysis of SIRPγ expression levels in different immune cell populations between MPE and TPE (TPE, n = 11; MPE, n = 10 biologically independent samples per group). (**h**, **i**) Flow cytometric quantification of $SIRP\gamma^+CD4^+$ T cells ($CD3^+CD4^+SIRP\gamma^+$) frequencies in TPE versus matched peripheral blood: Representative plots (**h**) and statistical analysis (**i**) (n=10 patients). (**j**) Single-cell RNA sequencing analysis comparing *SIRPG* expression in $CD4^+$ T cells from pleural effusion versus peripheral blood. (**k**) t-SNE representation of $CD8^+$ T cells revealing 12 clusters. (**l**) Bubble plot visualizing subset-defining gene signatures across $CD8^+$ T cell subpopulations. Statistical significance determined by paired two-tailed t-test (**e**, **i**) and two-way ANOVA (**g**). Data are presented as mean values ± SD (**e-g**, **i**).

Supplementary Figure 6

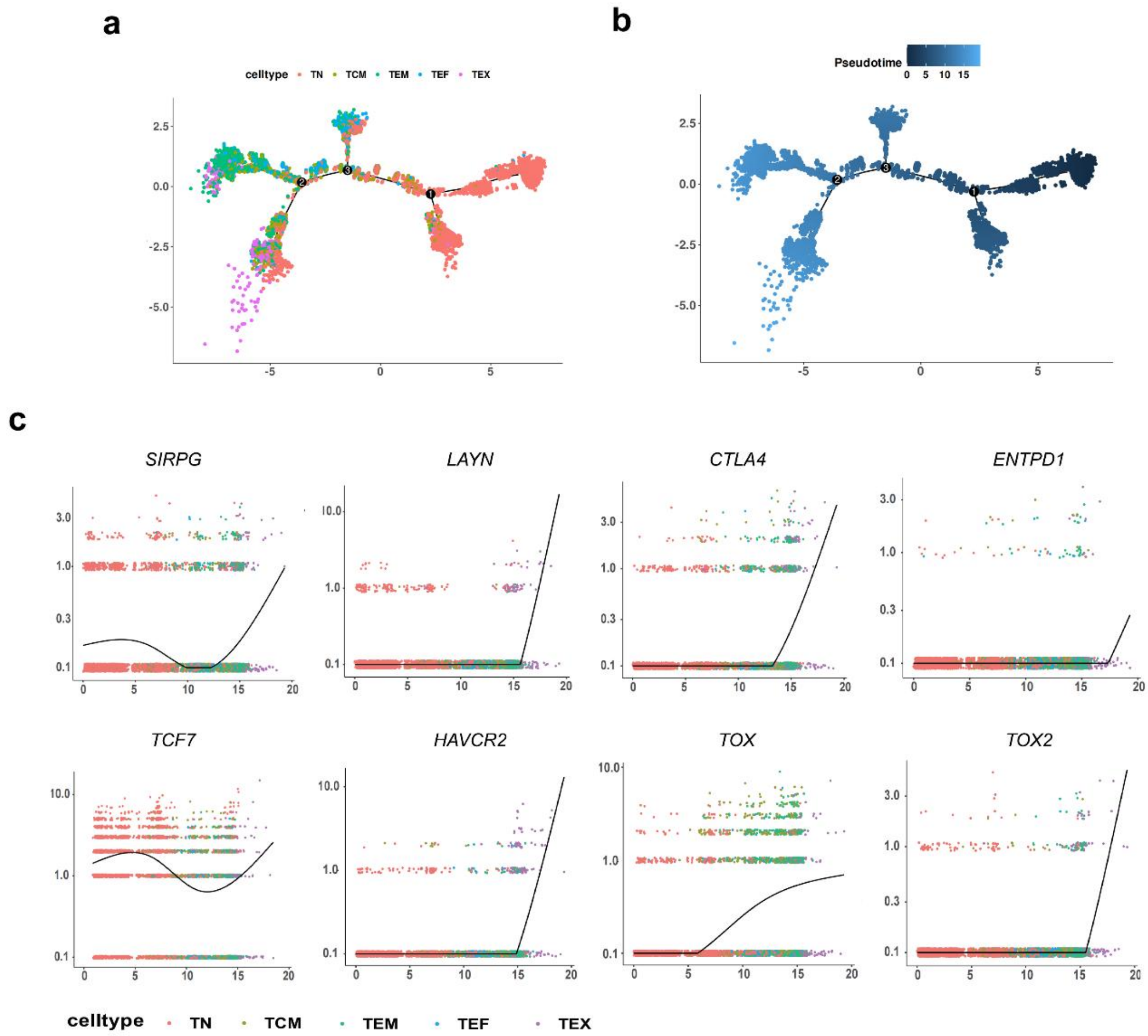

Supplementary Figure 6 SIRPγ is highly expressed in exhausted CD8⁺ T cells in TPE, related to Figure 2. (**a**)The differentiation trajectory of $CD8^+$ T cells clusters. (**b**) Monocle2 pseudotime analysis showing the transition tendency among different sub-clusters of $CD8^+$ T cells. (**c**) Expression dynamics of marker gene signatures of exhausted $CD8^+$ T cells along the pseudotime axis. T_{EX}, exhausted T cells; T_{EM}, effector memory-like T cells; T_{EFF}, effector T cells; T_{CM}, central memory-like T cells; T_N, naïve-like T cells.

Supplementary Figure 7

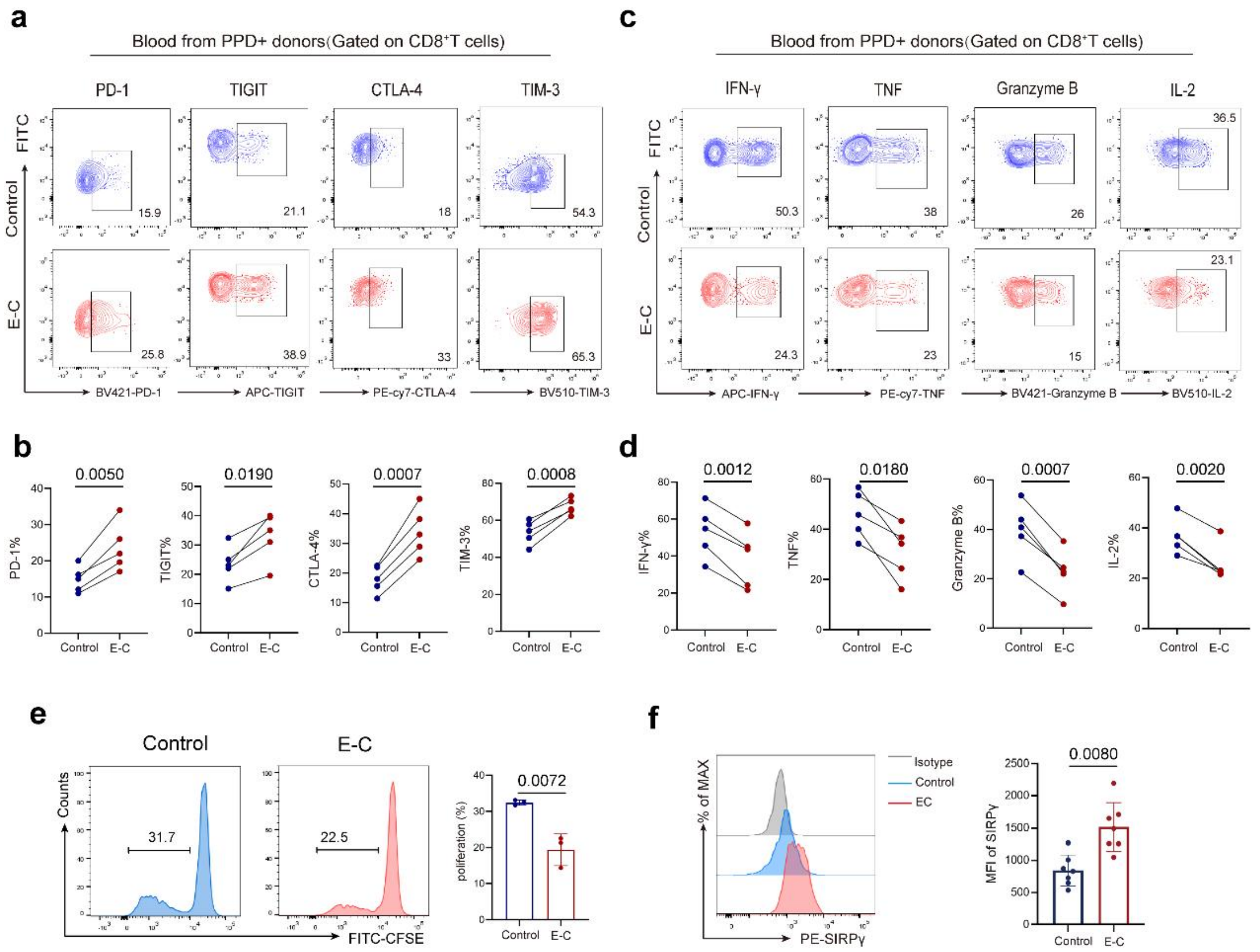

Supplementary Figure 7 SIRPγ serves as a potential mediator of T cell exhaustion in TPE, related to Figure 3. (**a–f**) Peripheral blood mononuclear cells were isolated from PPD+ donors using MACS and activated with CD3/CD28 antibodies for 24 hours. Subsequently, cells were treated with recombinant fusion antigen ESAT-6-CFP-10 (E-C). Flow cytometric analyses were conducted to measure the expression levels of inhibitory receptors (PD-1, TIGIT, CTLA-4, TIM-3) (**a**, **b**) and cytokines (IFN-γ, TNF, Granzyme B, IL-2) (**c**, **d**) (n = 5 biological replicates). Proliferation was evaluated using CFSE fluorescence labeling (**e**) (n = 3 independent experiments), while flow cytometric analysis was used to detect changes in SIRPγ expression in $CD8^+$ T cells following E-C treatment (n = 7 biological replicates per group). Statistical significance was determined by unpaired two-tailed Student's t-test (**e**) and paired two-tailed t-tests (**b**, **d**, **f**). Data are presented as mean values ± SD (**b-f**).

Supplementary Figure 8

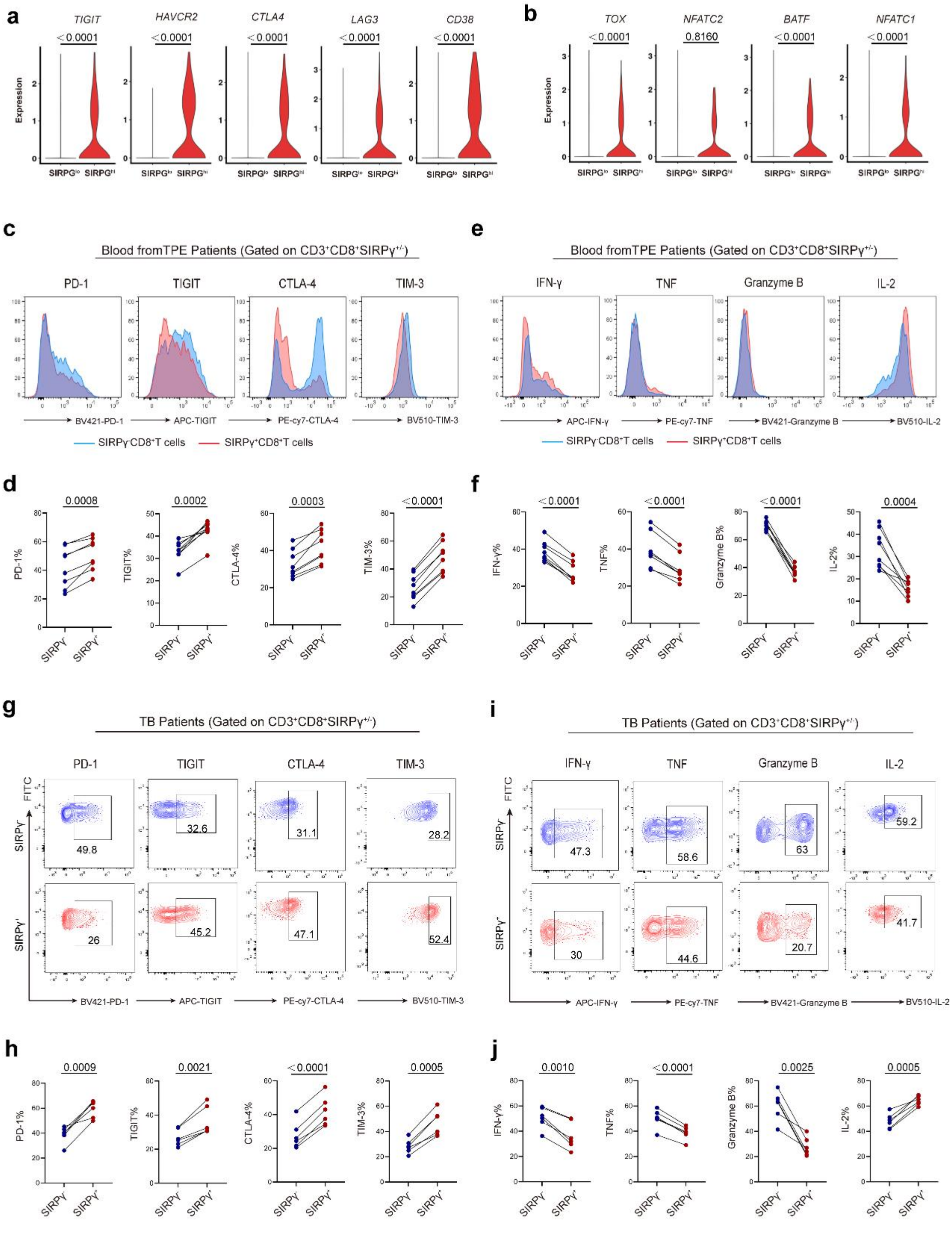

Supplementary Figure 8 SIRPγ serves as a potential mediator of T cell exhaustion in TPE, related to Figure 3. (**a**, **b**) Violin plots depict differential gene expression between SIRPγ^{+} and SIRPγ^{-} CD8^{+} T cells in TPE. (**c-f**) CD8^{+} T cells isolated from peripheral blood of TPE patients were analyzed via flow cytometry for expression of inhibitory receptors (PD-1, TIGIT, CTLA-4, TIM-3) (**c**, **d**) and cytokines (IFN-γ, TNF,

Granzyme B, IL-2) (**e**, **f**) in SIRPγ$^+$ and SIRPγ$^-$ subsets. n = 8 patients for (**d**) and (**f**). (**g-j**) CD8$^+$ T cells from tuberculosis (TB) patients were assessed by flow cytometry for inhibitory receptors (PD-1, TIGIT, CTLA-4, TIM-3) (**g**, **h**) and cytokines (IFN-γ, TNF, Granzyme B, IL-2) (**i**, **j**). n = 6 patients for (**h**) and (**j**). Statistical significance was determined by paired two-tailed t-tests (**d**, **f**, **h**, **j**).

Supplementary Figure 9

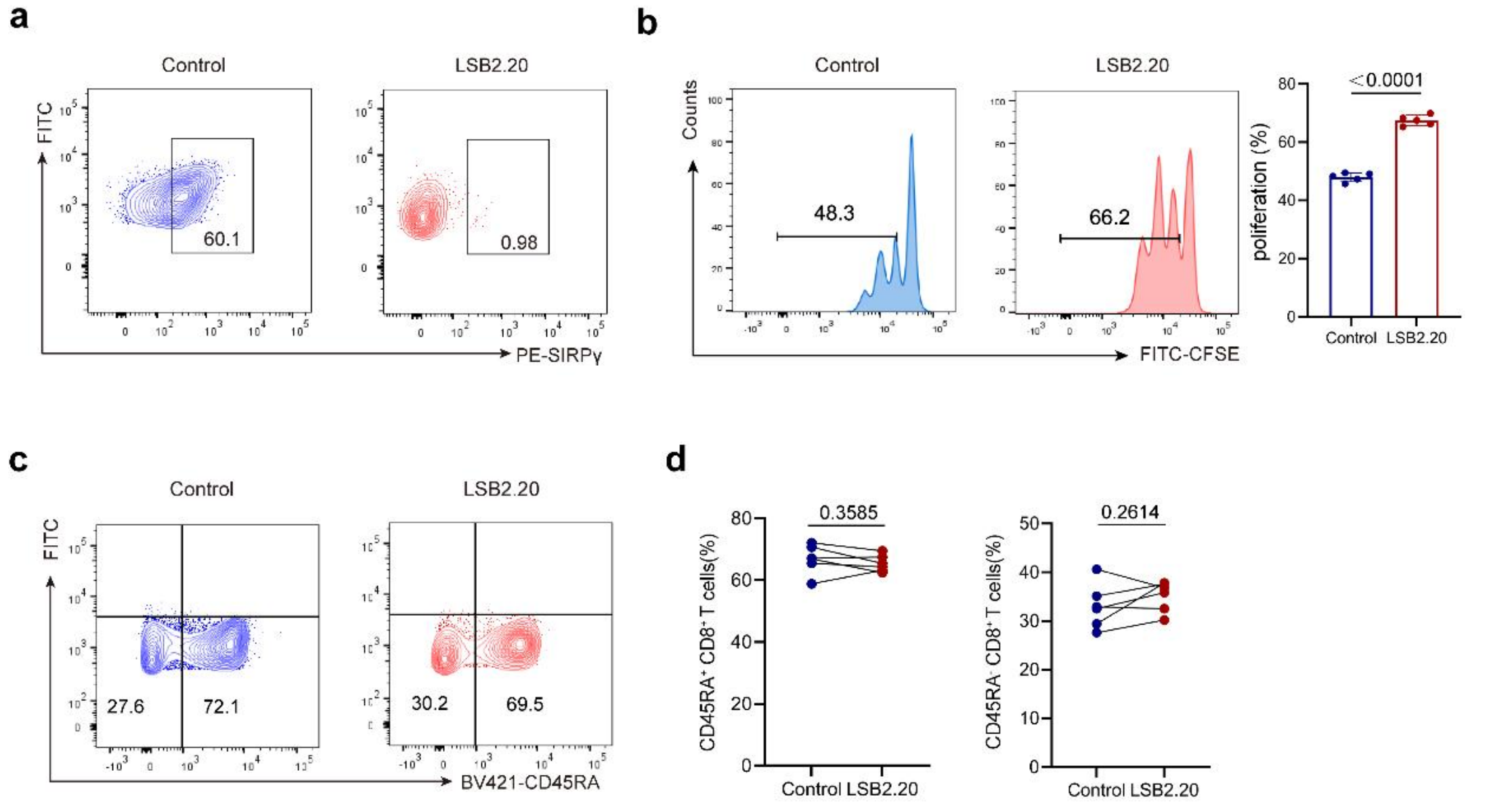

Supplementary Figure 9 SIRPγ serves as a potential mediator of T cell exhaustion in TPE, related to Figure 3. (**a**) Schematic illustration of SIRPγ blockade using the specific anti-SIRPγ monoclonal antibody LSB2.20. (**b-d**) $CD8^+$ T cells isolated from TPE were activated with anti-CD3/CD28 antibodies for 24 hours, followed by 48-hour treatment with LSB2.20 (10 μg/mL). Proliferation of SIRPγ-blocked $CD8^+$ T cells was assessed via CFSE dilution (n = 5 independent experiments) (**b**). Flow cytometry quantified proportions of naïve ($CD45RA^+CD8^+$) and memory-like ($CD45RA^-CD8^+$) T cell subsets (n=5 patients per group) (**c**, **d**). Statistical significance was determined by unpaired two-tailed Student's t-test (**b**) and paired two-tailed t-tests (**d**). Data are presented as mean values ± SD (**b**).

Supplementary Figure 10

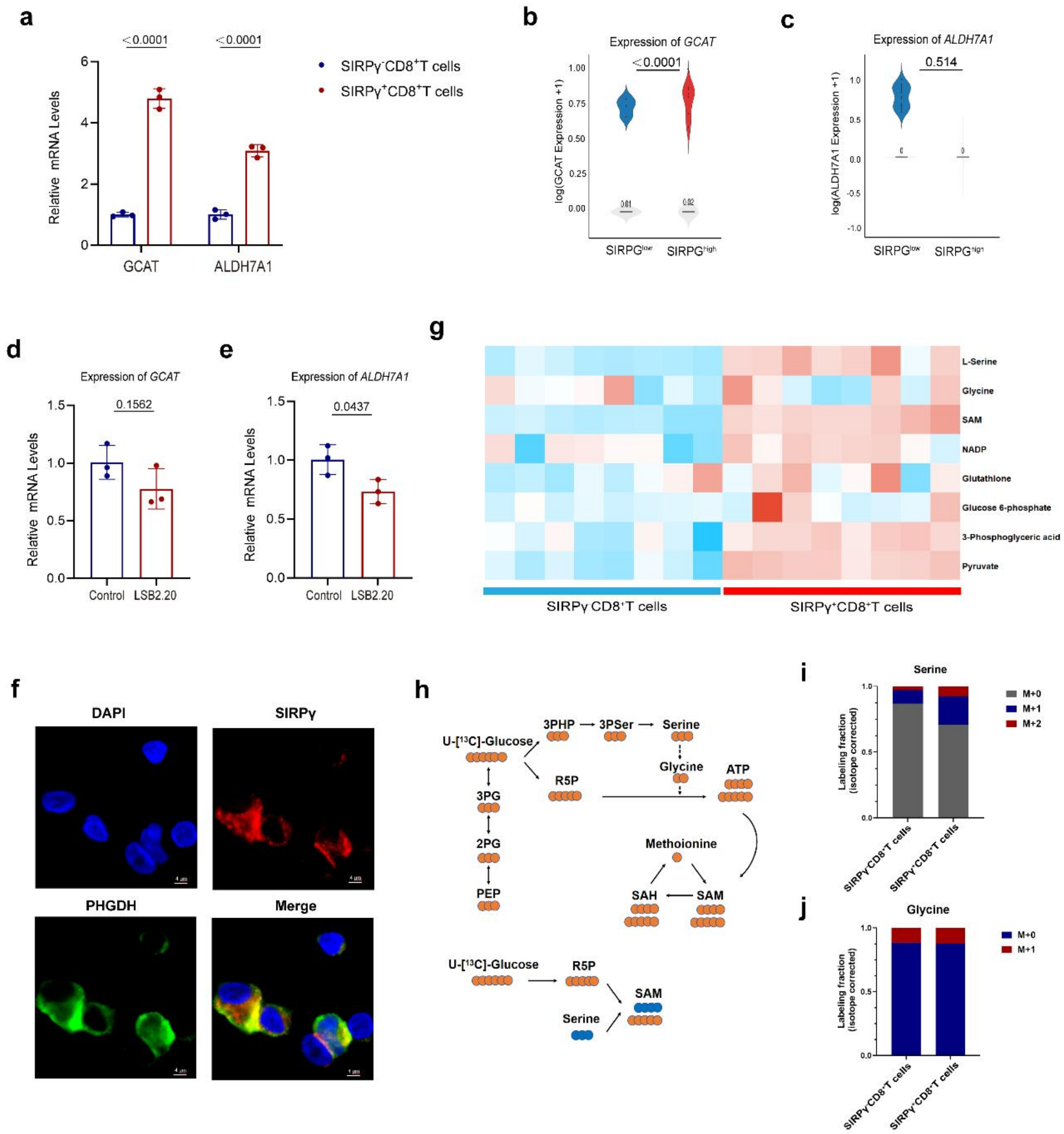

Supplementary Figure 10 SIRPγ promotes CD8+ T cell exhaustion by reprogramming serine metabolism in TPE, related to Figure 4. (**a**) Relative *GACT* and *ALDH7A1* expression in sorted SIRPγ− and SIRPγ+ CD8+ T cells from TPE assessed by qPCR (n = 3 independent experiments). (**b**, **c**) *GCAT* (**b**) and *ALDH7A1* (**c**) expression SIRPγ^{low} and SIRPγ^{high} CD8+ T cells derived from single-cell RNA sequencing data. (**d**, **e**) CD8+ T cells isolated from TPE were activated with anti-CD3/CD28 antibodies for 24 hours, followed by 48-hour treatment with LSB2.20(10 µg/mL). *GCAT* (**d**) and *ALDH7A1* (**e**) expression were analyzed by qPCR (n = 3 independent experiments). (**f**) Representative images of SIRPγ and PHGDH staining in

CD8^{+} T cells of TPE. Scale bar 4μm. (**g–j**) SIRPγ^{-} and SIRPγ^{+} CD8^{+} T cells isolated from TPE were incubated with U-[^{13}C]-glucose for six hours. Heatmaps (**g**) and schematic representations (**h**) illustrate the incorporation of U-[^{13}C]-glucose into various metabolites. The percent distribution of each isotopic isomer in the serine (**i**) and glycine (**j**) pool is shown (n = 8 patients per group). Statistical significance was determined by unpaired two-tailed Student's t-test (**a**, **d**, **e**). Data are presented as mean values ± SD (**a**, **d**, **e**).

Supplementary Figure 11

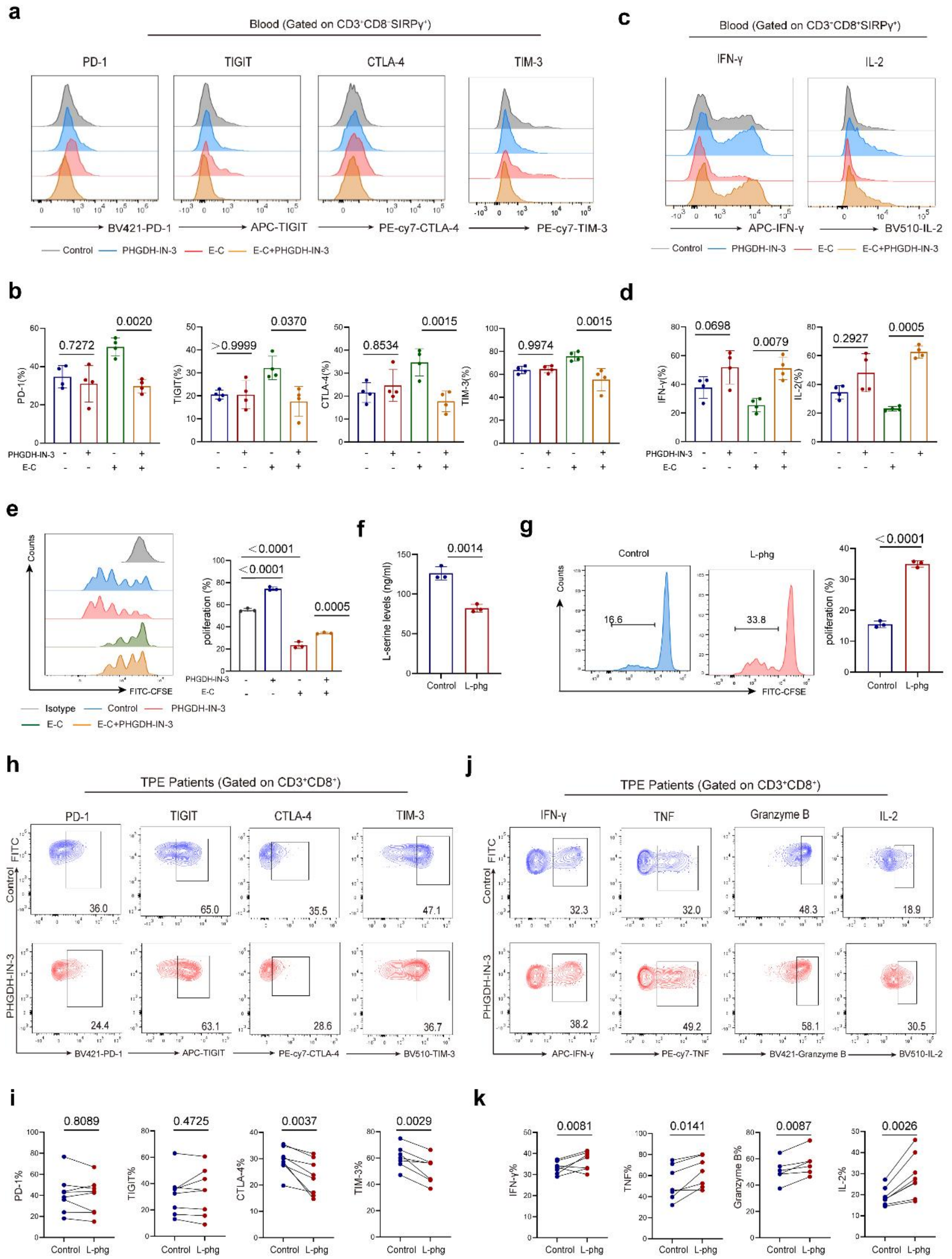

Supplementary Figure 11 SIRPγ drives T cell exhaustion through PHGDH-mediated serine metabolism in TPE, related to Figure 5. (**a-e**) Mononuclear cells were isolated from the peripheral blood of TPE patients and treated with recombinant fusion antigen ESAT-6-CFP-10 (E-C) (10 μg/ml) for 20h. Subsequently, they were

exposed to the PHGDH inhibitor PHGDH-IN-3 (10 μM) for 48h. Flow cytometry was employed to analyze the levels of inhibitory receptors (PD-1, TIGIT, CTLA-4, TIM-3) (**a**, **b**) and cytokines (IFN-γ, IL-2) (**c**, **d**) in $CD8^+$ T cells. n = 4 biological replicates per group for (**b**) and (**d**). Proliferation was assessed using CFSE fluorescence labeling (**e**) (n = 3 independent experiments). (**f-k**) $CD8^+$ T cells were isolated from TPE and activated with CD3/CD28 antibodies for 24h, followed by treatment with the alanine-serine-cysteine transporters (ASCT)-specific inhibitor L-phenylalanine (L-phg) (50 μM) for 48h. Intracellular L-serine levels were measured via ELISA (**f**) (n = 3 independent experiments). Proliferation was evaluated via CFSE fluorescence labeling (**g**) (n = 3 independent experiments). Flow cytometry was performed to assess inhibitory receptor levels (PD-1, TIGIT, CTLA-4, TIM-3) (**h**, **i**) and cytokine expression (IFN-γ, TNF, Granzyme B, IL-2) (**j**, **k**). The number of independent replicates is indicated as follows: n = 9 (TIM-3), n = 8 (PD-1, TIGIT. CTLA-4, IL-2), n = 7 (IFN-γ, TNF), n = 6 (Granzyme B) per group. Statistical analysis was determined by unpaired two-tailed t-test (**f**, **g**), paired two-tailed t-tests (**i**, **k**), and one-way ANOVA (**b**, **d**, **e**). Data are presented as mean values ± SD (**b, d-g**).

Supplementary Figure 12

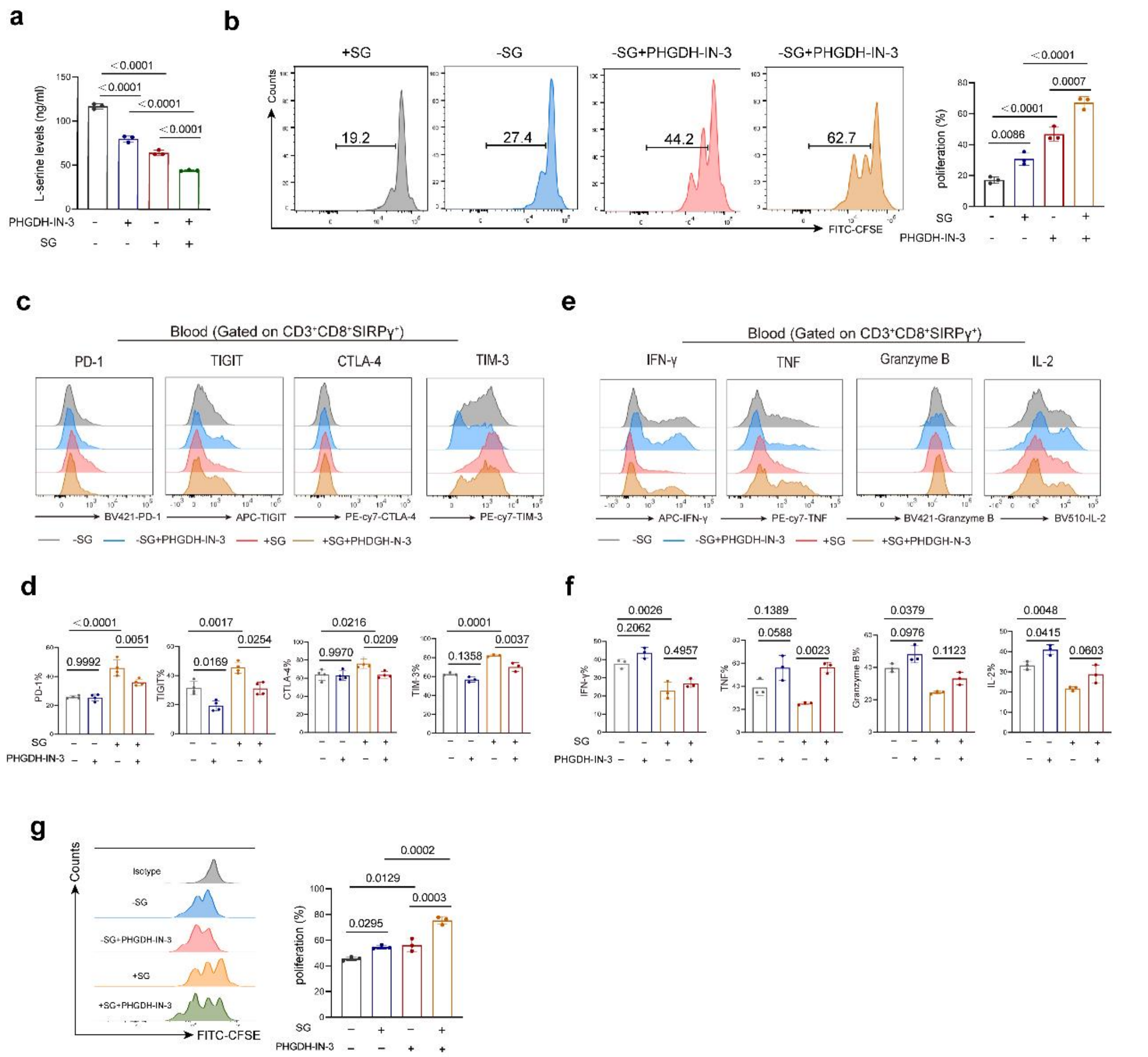

Supplementary Figure 12 SIRPγ drives T cell exhaustion through PHGDH-mediated serine metabolism in TPE, related to Figure 5. (**a**, **b**) TPE-isolated CD8⁺ T cells were treated with or without PHGDH-IN-3 (24 h), with or without serine/glycine (SG) deprivation (3d). Intracellular L-serine levels were quantified by ELISA (**a**) (n = 3 independent experiments). Proliferation was assessed via CFSE dilution (**b**) (n = 3 independent experiments). (**c-g**) Mononuclear cells were isolated from the peripheral blood of TPE patients and treated with recombinant fusion antigen ESAT-6-CFP-10 (E-C) (10 μg/ml) for 2 days. They were treated with or without PHGDH-IN-3 (24 h), with or without serine/glycine (SG) deprivation (3d). Flow cytometry quantified expression of inhibitory receptors (PD-1, TIGIT, CTLA-4, TIM-3) (**c**, **d**) and cytokines (IFN-γ, TNF, Granzyme B, IL-2) (**e**, **f**). n = 3 biological replicates experiments per group for (**d**) and (**f**). Proliferation was assessed via CFSE dilution (**g**) (n = 3 independent

experiments). Statistical analysis was determined by one-way ANOVA (**a**, **b**, **d**, **f**, **g**). Data are presented as mean values ± SD (**a**, **b**, **d**, **f**, **g**).

Supplementary Figure 13

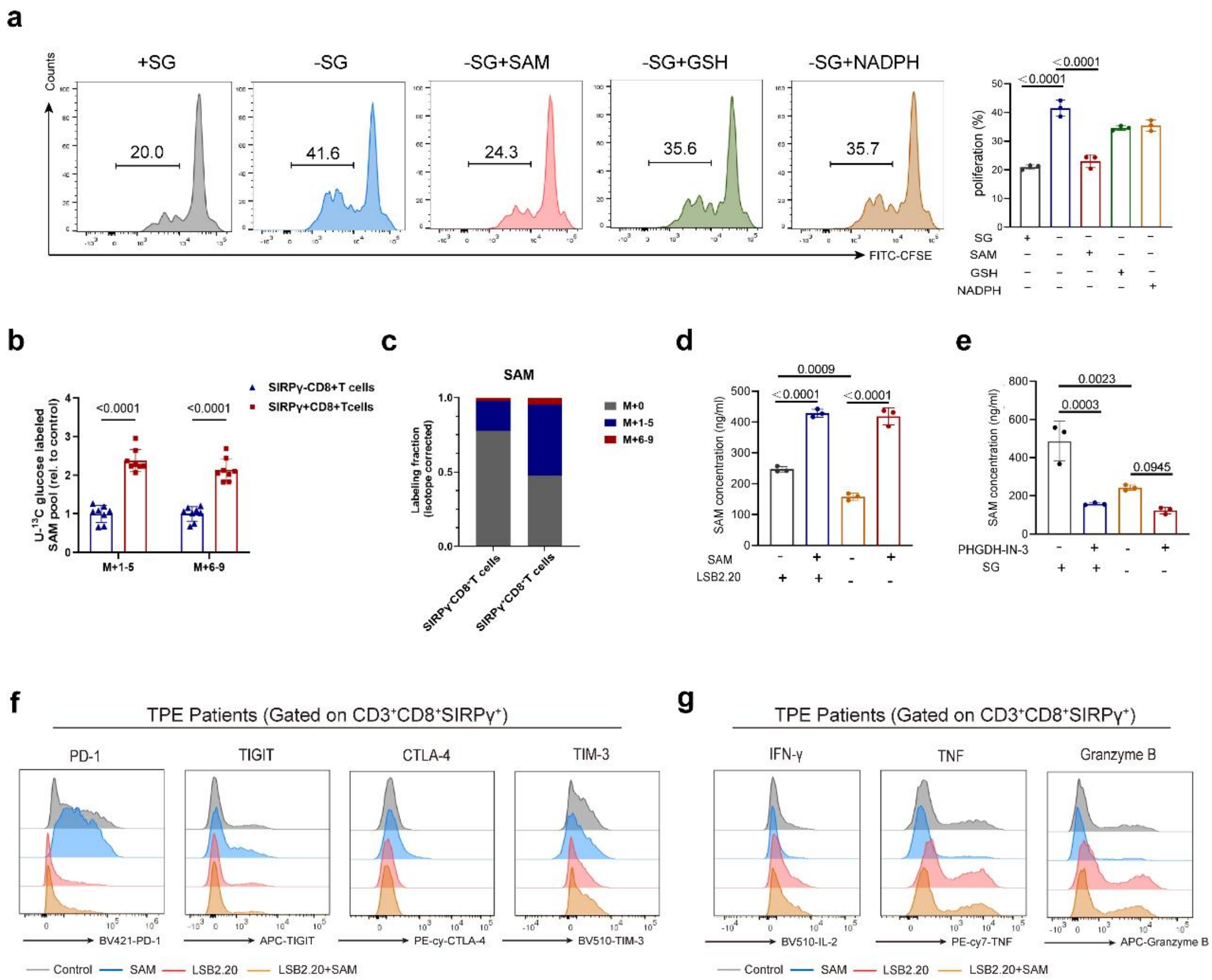

Supplementary Figure 13 SIRPγ drives T cell exhaustion through PHGDH-mediated serine metabolism in TPE, related to Figure 6. (**a**) $CD8^+$ T cells isolated from TPE were activated with CD3/CD28 antibodies for 24h and subjected to serine/glycine (SG) medium starvation for 3 days. Cells were subsequently treated with 400 μM NADPH, GSH, or SAM for 48 h. Proliferation was assessed via CFSE dilution (n = 3 independent experiments). (**b**, **c**) Incorporation of U-[^{13}C]-glucose carbon into SAM (**b**) and the percentage distribution of isotopologues in the SAM pool (**c**) in sorted $SIRP\gamma^-$ and $SIRP\gamma^+$ $CD8^+$ T cells derived from TPE (n = 8 patients per group). (**d**) $CD8^+$ T cells isolated from TPE were treated with the specific anti-SIRPγ monoclonal antibody LSB2.20 (10 μg/mL) for 24h, followed by the addition of SAM (400 μM) for 48h. Intracellular SAM levels were measured using ELISA (n = 3 independent experiments). (**e**) $CD8^+$ T cells isolated from TPE samples were treated with or without the PHGDH inhibitor PHGDH-IN-3 for 24h, followed by SG medium starvation for 3

days. Intracellular SAM levels were measured using ELISA (n = 3 independent experiments). (**f**, **g**) $CD8^+$ T cells from TPE were treated with the specific anti-SIRPγ monoclonal antibody LSB2.20 (10 μg/mL) for 24h, followed by 400 μM SAM treatment for 48h. Flow cytometry was performed to assess the expression levels of inhibitory receptors (TIGIT, TIM-3, CTLA-4, PD-1) (**f**) and cytokines (IL-2, IFN-γ, Granzyme B, TNF) (**g**). Statistical analysis was determined by one-way ANOVA (**a**, **d**, **e**) and two-tailed Student's t-test (**b**). Data are presented as mean values ± SD (**a**, **b**, **d**, **e**).

Supplementary Figure 14

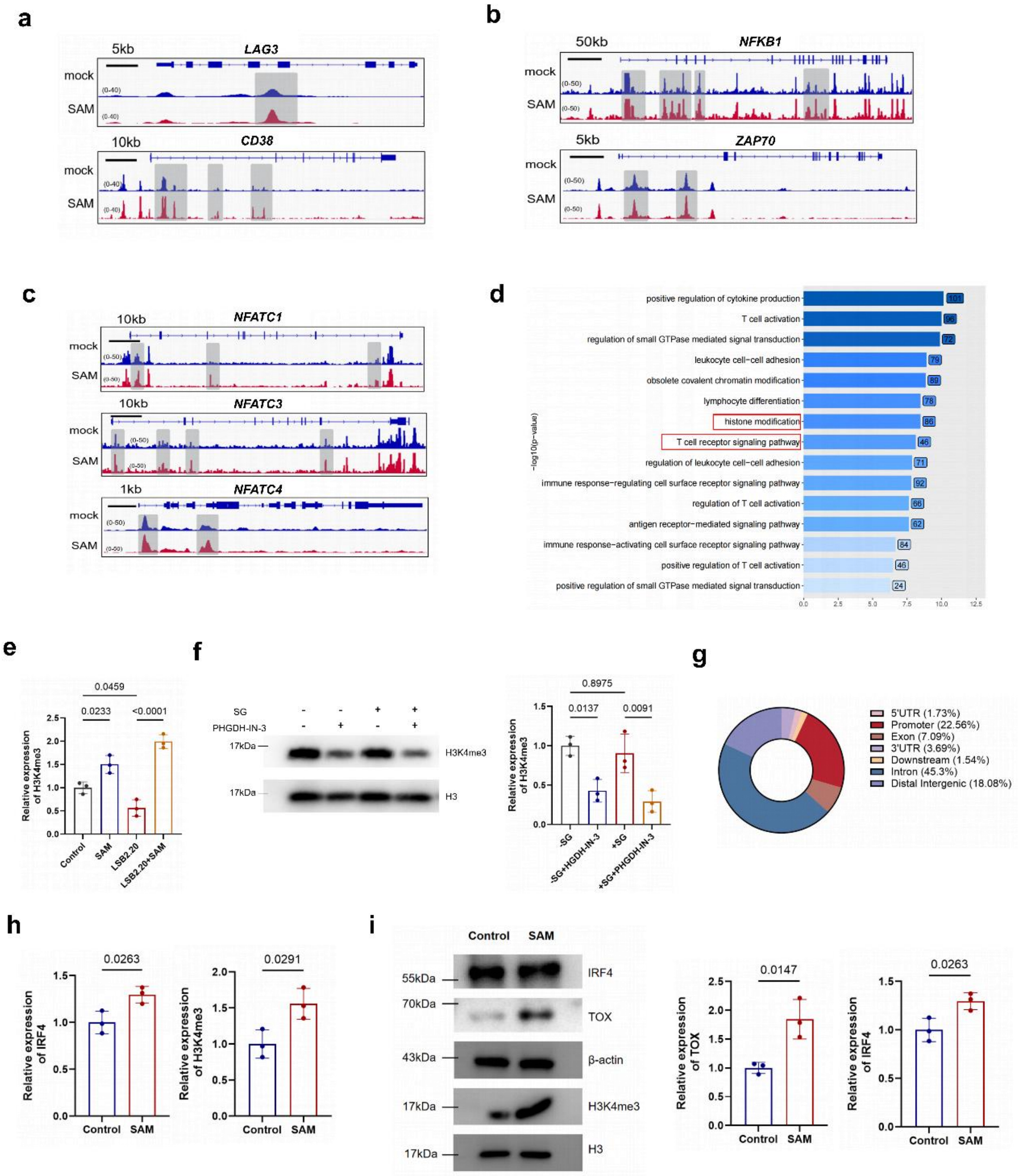

Supplementary Figure 14 Serine-derived SAM promotes an active epigenetic state at the NFATC2 locus to drive T cell exhaustion, related to Figure 7. (**a-c**) ATAC-seq coverage at *LAG3* and *CD38* (**a**); *NFKB1* and *ZAP70* (**b**); *NFTAC1*, *NFATC3*, and *NFATC4* (**c**). All peaks were highlighted in gray bars. (**d**) GO pathway enrichment analysis of upregulated differentially expressed genes (DEGs) in mock- or SAM-treated CD8^{+} T cells. (**e**) Western blot analysis was performed to assess H3K4me3 levels markers in CD8^{+} T cells derived from TPE (n = 3 independent experiments). Following

stimulation with anti-CD3/CD28 antibodies for 24 h, the cells were treated with the anti-SIRPγ monoclonal antibody LSB2.20 (10 μg/mL), and then supplemented with SAM (400 μM) for 48 h. (**f**) $CD8^+$T cells isolated from TPE were activated with anti-CD3/CD28 antibodies for 24 h, treated with or without the PHGDH inhibitor PHGDH-IN-3 for 24 h, followed by serine/glycine (SG) starvation for 3 days. H3K4me3 levels were analyzed by immunoblotting (n = 3 independent experiments). (**g**) Pie chart illustrating the genomic distribution of H3K4me3 CUT&Tag-seq peaks in mock- or SAM-treated $CD8^+$T cells. (**h**) Protein expression of NFATC2 and H3K4me3 was analyzed by Western blot in mock- or SAM-treated $CD8^+$ T cells (n = 3). (**i**) Western blot analysis of TOX, IRF4, and H3K4me3 in mock- or SAM-treated $CD8^+$T cells (n = 3 independent experiments). Statistical significance was determined by one-way ANOVA (**e**, **f**) and unpaired two-tailed t-test (**h**, **i**). Data are presented as mean values ± SD (**e**, **f**, **h**, **i**).

Supplementary Figure 15

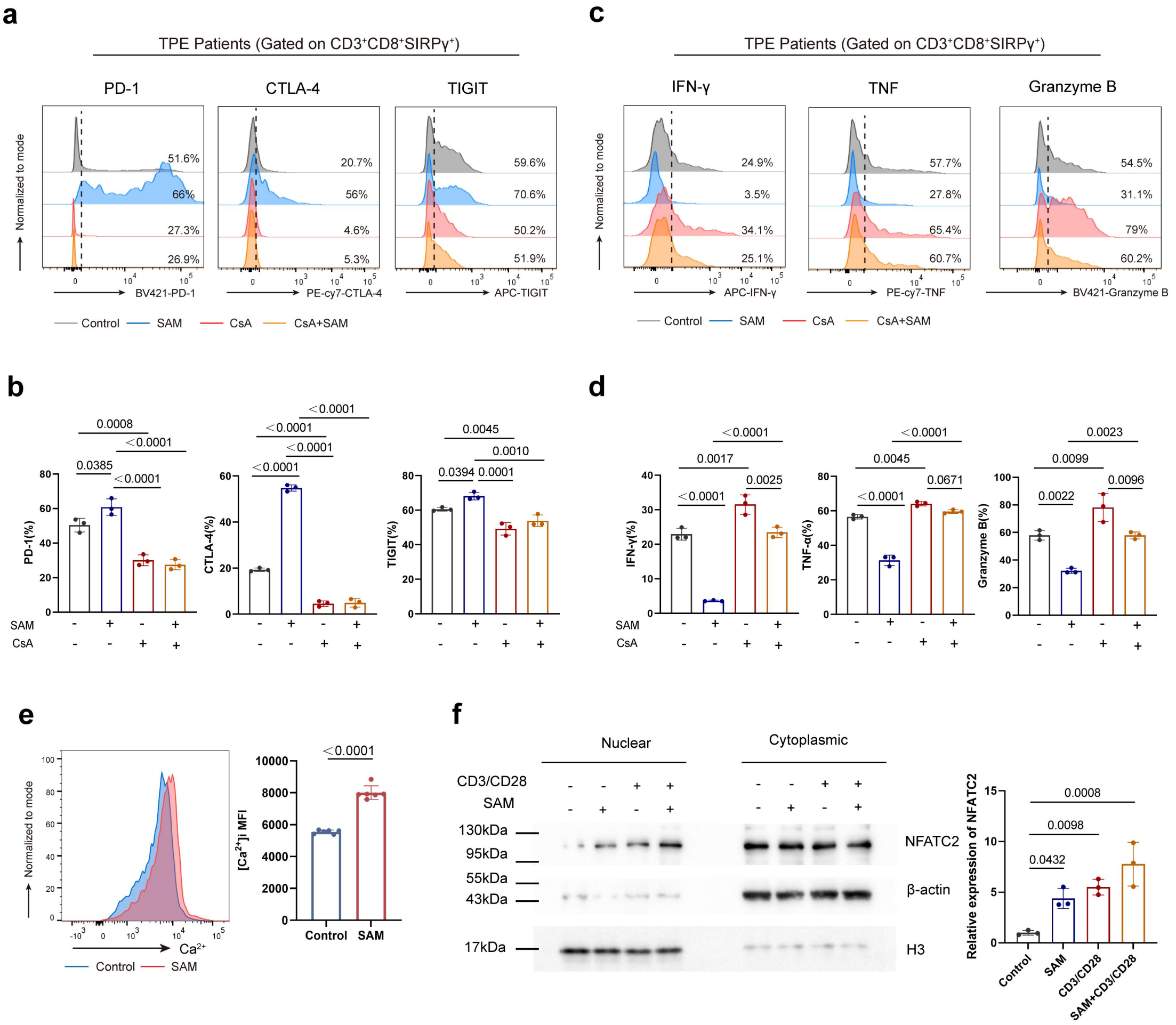

Supplementary Figure 15 Serine-derived SAM promotes an active epigenetic state at the NFATC2 locus to drive T cell exhaustion, related to Figure 7. (**a-d**) CD8$^+$ T cells isolated from TPE were activated ex vivo and treated with or without 400 μM SAM ± cyclosporin A (CsA) (1 μM). The expression levels of inhibitory receptors (TIGIT, CTLA-4, PD-1) (**a**, **b**) and cytokines (Granzyme B, IFN-γ, TNF) (**c**, **d**) were measured by flow cytometry. n = 3 biological replicates experiments per group for (**b**) and (**d**). (**e**) CD8$^+$ T cells from TPE were incubated with SAM (400 μM) for 20 h, after which intracellular Ca^{2+} levels ([Ca^{2+}]i) were assessed via flow cytometry (n = 6 biological replicates experiments per group). (**f**) Representative Western blots of nuclear and cytoplasmic extracts from CD8$^+$ T cells isolated from TPE, either untreated or treated with CD3/CD28 antibodies (1 μg/mL) ± SAM (400 μM), showing NFATC2 protein expression (n = 3 independent experiments). Statistical analysis was determined

by unpaired two-tailed t-test (**e**) and one-way ANOVA (**b**, **d**, **f**). Data are presented as mean values ± SD (**b-f**).

Supplementary Figure 16

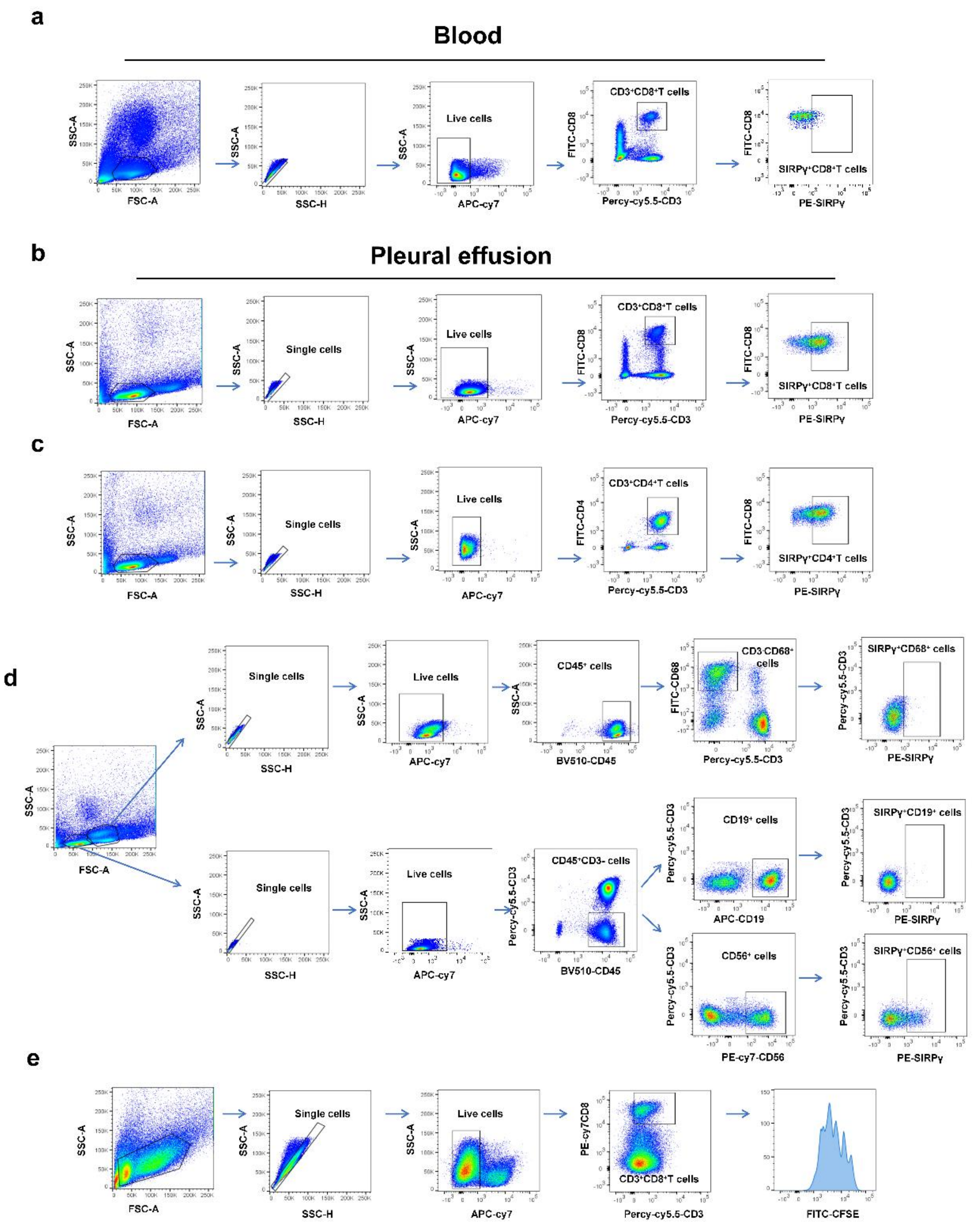

Supplementary Figure 16 Gating strategy for immune cells from pleural effusion and blood. (**a**, **b**) Gating strategy for SIRPγ$^+$CD3$^+$CD8$^+$T cells isolated from blood (**a**) and pleural effusion (**b**); (**c**) Gating strategy for SIRPγ$^+$CD3$^+$CD4$^+$T cells isolated from pleural effusion; (**d**) Gating strategy for isolating SIRPγ$^+$ immune cells from pleural effusion. Shown are the strategies for identifying SIRPγ$^+$ macrophages (CD45$^+$CD3$^-$

CD68^{+}), SIRPγ^{+} B cells (CD45^{+}CD3^{-}CD19^{+}), and SIRPγ^{+} natural killer cells (CD45^{+}CD3^{-}CD56^{+}). (**e**) Gating strategy for SIRPγ^{+}CD3^{+}CD8^{+}T cells used in the proliferation assay.

Supplementary Table 1 Diagnostic performance of pleural SIRPγ, ADA, and SIRPγ×ADA between TPE and MPE patients

Variables	Cut-off value	AUC (95% CI)	Sensitivity (%)	Specificity (%)	PLR	NLR	PPV (%)	NPV (%)
SIRPγ	1324 pg/ml	0.9037 (0.8375 - 0.9698)	84.09	90.00	8.409	0.1768	89.37	84.98
ADA	24.2 U/L	0.9361 (0.884 - 0.9882)	86.36	87.50	6.9088	0.1559	87.36	86.52
SIRPγ×ADA	39414 ng U/L^2	0.9585 (0.9190 - 0.9980)	93.18	92.50	12.424	0.0737	94.52	83.94

TPE, tuberculous pleural effusion; MPE, malignant pleural effusion; SIRPγ, signal regulatory protein γ; ADA, adenosine deaminase; AUC, area under the curve; PLR, positive likelihood ratio; NPV, negative likelihood ratio; PPV, Positive Predictive Value; NLR, negative predictive value.

Supplementary Table 2 Diagnostic performance of pleural SIRPγ, ADA, and SIRPγ×ADA between TPE and non-TPE patients

Variables	Cut-off value	AUC (95% CI)	Sensitivity (%)	Specificity (%)	PLR	NLR	PPV (%)	NPV (%)
SIRPγ	1324 pg/ml	0.9481 (0.9162 - 0.9801)	82.09	91.03	9.152	0.1967	87.10	85.71
ADA	24.2 U/L	0.9238 (0.8778 - 0.9699)	80.60	92.31	10.481	0.2102	88.33	85.71
SIRPγ×ADA	39414 ng U/L^2	0.9732 (0.9510 - 0.9954)	81.82	97.44	31.961	0.1866	94.55	84.44

TPE, tuberculous pleural effusion; SIRPγ, signal regulatory protein γ; ADA, adenosine deaminase; AUC, area under the curve; PLR, positive likelihood ratio; NPV, negative likelihood ratio; PPV, Positive Predictive Value; NLR, negative predictive value.

Supplementary Table 3 Demographic, cytological and biochemical characteristics in pleural effusions

	Training Cohort			Validation Cohort				
Parameters	TPE (n=44)	MPE (n=40)	P value	TPE (n=66)	MPE (n=35)	PPE (n=27)	Miscellaneous (n=16)	P value
Sex, male, %	61.4	52.5	0.5090	71.6	67.7	74.1	35.3	0.1602
Age, years	48.43 ± 21.81	63.73 ± 14.27	0.0003	58.37± 19.63	61.50 ± 14.08	64.58 ± 14.95	60.44 ± 16.31	0.4623
ADA, U/L	40.65 ± 13.69	14.25 ± 9.371	<0.0001	38.56 ± 21.70	10.24 ± 6.619	16.35 ± 17.01	11.79 ± 6.091	<0.0001
LDH, U/L	644.5 ± 595.0	711.1 ± 671.4	0.6311	486.1 ± 298.6	272.3 ± 234.7	466.0 ± 701.0	267.7 ± 171.7	0.0228
Protein, g/L	46.88 ± 5.706	41.82 ± 5.706	0.0038	42.42 ± 11.08	40.74 ± 8.347	34.15 ± 11.28	30.14 ± 10.66	<0.0001
Cell counts, cells/mm3	4198 ± 2663	3685 ± 2114	0.3699	2597 ± 1902	4077 ± 3166	5622 ± 7889	685.8 ± 748.6	0.0009
Mononuclear cells, %	77.7 ± 17.15	79.55 ± 20.01	0.6625	79.09 ± 20.25	80.06 ± 11.96	83.23 ± 20.01	84.25 ± 8.729	0.6155

$P<0.05$ represents statistical significance; Data are presented as mean ± SEM. In training cohort, compaisons were pertomed using unpaired two-tailed t-test (age, ADA, LDH, glucose, protein, cell counts, lymphocyte) and two-sided Fisher's exact test (sex). In validation cohort, compaisons were pertomed using unpaired t one-way ANOVA (age, ADA, LDH, glucose, protein, cell counts, lymphocyte) and Fisher's exact test (sex). LDH, lactate dehydrogenase; ADA, adenosine deaminase; MPE, malignant pleural effusion; PPE, parapneumonic pleural effusion; TPE, tuberculous pleural effusion.

Supplementary Table 4 Material and Methods

REAGENT	SOURCE	IDENTIFIER
**Antibodies**		**Cat# and dilutions**
Fixable Viability Stain 780	BD Pharmingen	Cat# 565388 (1: 50)
Human TruStain FcX™	Biolegend	Cat# 422302 (1: 50)
Brilliant Violet 510™ anti-human CD45 Antibody	Biolegend	Cat# 304036 (1: 50)
PerCP/Cyanine5.5 anti-human CD3	Biolegend	Cat# 317336 (1: 50)
FITC anti-human CD8a	Biolegend	Cat# 301050 (1: 20)
PE/Cyanine7 anti-human CD8a	Biolegend	Cat# 301012 (1: 20)
FITC anti-human CD4	Biolegend	Cat# 300538 (1: 50)
FITC anti-human CD68	Biolegend	Cat# 333806 (1: 50)
APC anti-human CD19	Biolegend	Cat# 982406 (1: 50)
PE/Cyanine7 anti-human CD56	Biolegend	Cat# 985912 (1: 50)
PE anti-human CD172g (SIRPγ)	Biolegend	Cat# 336606 (1: 100)
Brilliant Violet 421™ anti-human CD279 (PD-1)	Biolegend	Cat# 329920 (1: 50)
APC anti-human TIGIT (VSTM3)	Biolegend	Cat# 372706 (1: 50)
Brilliant Violet 510™ anti-human CD366 (TIM-3)	Biolegend	Cat# 345030 (1: 50)
PE/Cyanine7 anti-human CD366 (Tim-3) Antibody	Biolegend	Cat# 364820 (1: 50)
PE/Cyanine7 anti-human CD152 (CTLA-4)	Biolegend	Cat# 349914 (1: 50)
APC anti-human IFN-γ	Biolegend	Cat# 502512 (1: 50)
BD Horizon™ BV421 Mouse Anti-Human IFN-γ	BD Horizon™	Cat# 562988 (1: 50)

PE/Cyanine7 anti-human TNF	Biolegend	Cat# 502930 (1: 50)
Brilliant Violet 510™ anti-human IL-2	Biolegend	Cat# 500338 (1: 20)
Brilliant Violet 421™anti-human/mouse Granzyme B	Biolegend	Cat# 515410 (1: 100)
APC anti-human/mouse Granzyme B Recombinant Antibody	Biolegend	Cat# 372204 (1: 50)
Ultra-LEAF™ Purified anti-human CD28 Antibody	Biolegend	Cat# 302934 (1: 1000)
Ultra-LEAF™ Purified anti-human CD3 Antibody	Biolegend	Cat# 317326 (1: 1000)
CFSE	BD Pharmingen	Cat# 565082
**Antibodies**		**Cat# and dilutions**
Anti-Histone H3	Abcam	Cat# ab12079 (1:1500)
Tri-Methyl-Histone H3 (Lys4)/H3K4me3 Antibody	Affinity	Cat# DF6935 (1:1500)
Tri-Methyl-Histone H3 (Lys9)/H3K9me3 Antibody	Affinity	Cat# DF6938 (1:1500)
Anti-Trimethyl-Histone H3 (Lys27) /H3K27me3Rabbit mAb	PTM Bio	Cat# PTM-647RM (1:1500)
SIRPG Antibody	Affinity	Cat# DF4508 (WB, 1:1500; IF, 1:200)
PHGDH Polyclonal antibody	proteintech	Cat# 14719-1-AP (WB, 1:1500; IF, 1:200)
Anti-IRF4 Rabbit pAb	ABclonal	Cat# A0524 (1:1000)
Anti-TOX Antibody	Abcam	Cat# ab237009 (1:1500)
Anti-NFATC2 Antibody	Abcam	Cat# ab283691 (1:1000)
mTOR Antibody	Abclonal	Cat# A11355 (1:1500)
Phospho-mTOR	Abclonal	Cat# AP0115 (1:1000)

(Ser2448) Antibody		
PI3-kinase p85-alpha/gamma pAb	Abmart	Cat# T40064 (1:1000)
Phospho-PI3-kinase p85- alpha/ gamma (Tyr467/199) pAb	Abmart	Cat# T40116 (1:1000)
AKT1/2/3 Antibody	Abmart	Cat# T55561 (1:1000)
Phospho-pan-AKT1/2/3 (Ser473) Antibody	Abmart	Cat# TA0016 (1:1000)
β-Actin Antibody	Cell Signaling Technology (CST)	Cat# 4967 (1:3000)
anti-CD4 Antibody	Abcam	Cat# ab133616 (1:100)
anti-CD8Antibody	Abcam	Cat# ab237709 (1:100)
anti-CD68 Antibody	Abcam	Cat# ab283654 (1:100)
**Chemicals, peptides, and recombinant proteins**		**Cat#**
Fetal Bovine Serum	Gibco	Cat# 16140071
RPMI 1640 Medium	Gibco	Cat# C11875500BT
1640 Medium (excluding serine and glycine)	Boster	Cat# DZPYG0062
Phorbol 12-myristate 13-acetate (PMA)	ENZO	Cat#BML-PE160-0001
Brefeldin A(BFA)	Biolegend	Cat# 420601
Ionomycin	ENZO	Cat#ALX-450-007-M001
EasySep Human CD8 Positive Selection Kit II	STEMCELL	Cat# 17853
Ficoll-Paque™	STEMCELL	Cat# 7851
Protease inhibitor	Servicebio	Cat# G2006
PMSF	Servicebio	Cat# G2008
RIPA Lysis Buffer	Servicebio	Cat# G2002
180 kDa Prestained Protein Marker	Vazyme	Cat# MP102-01

ColorMixed Protein Marker 250 (10-250 kDa)	ABclonal	Cat# RM02949
TRIzol Reagent	TAKARA	Cat# 9109
Invitrogen™ eBioscience™ IC Fixation Buffer	Invitrogen	Cat# 00-8222-49
Foxp3/Transcription Factor Staining Buffer	eBioscience	Cat# 00-5523-00
ECL Prime Western Blotting Detection Reagent	Servicebio	Cat# 32209
Hiscript qRT-PCR SuperMix	Vazyme	Cat# R122-01
AceQ qPCR SYBR Green Master Mix	Vazyme	Cat# R323-01
BCA Protein Assay Kit	Beyotime	Cat# P0012
Human Signal Regulatory Protein Gamma (SIRPg) ELISA Kit	GenMed	Cat# JYM2810Hu
Human L-Serine ELISA Kit	Camilo	Cat# 2H-KMLJh315544
General SAM/ S-Adenosyl methionine ELISA Kit	ABclonal	Cat# RK04519
Purified anti-human CD172g (SIRPγ)	BioLegend	Cat# 336602
PHGDH-IN-3	proteintech	Cat# CM11658
Purified anti-human CD172g (SIRPγ) Antibody	Biolegend	Cat# 336602
serine	Sigma-Aldrich	Cat#S4311
glycine	Sigma-Aldrich	Cat# G5417
L-Phenylglycine	Sigma-Aldrich	Cat# 237647
AL34662 (formate)	GLPBIO	Cat# GC 1703
NADPH tetrasodium sat	GLPBIO	Cat# GC34058

L-Glutathione reduced	GLPBIO	Cat# GC12203
S-Adenosy---methionine tosylate	GLPBIO	Cat# GC61259
**Deposited data**		
Human scRNA-seq of TPE		https://ngdc.cncb.ac.cn/
RNA-seq	This paper	
ATAC-seq	This paper	
CUT&Tag	This paper	

Supplementary Table 5 Flow cytometry panels

Cell Subsets								
	APC-cy7	Percy-cy5.5	FITC	PE	APC	PE-cy7	BV421	BV510
Human-macrophage cells	FVS	CD3	CD68	SIRPγ				CD45
Human-B cells	FVS	CD3		SIRPγ	CD19			CD45
Human-natural killer cells	FVS	CD3		SIRPγ		CD56		CD45
Human-CD4$^+$T cells	FVS	CD3	CD4	SIRPγ				CD45
Human-CD8$^+$T cells-1	FVS	CD3	CD8	SIRPγ				CD45
Human-CD8$^+$T cells-2	FVS	CD3	CD8	SIRPγ	IFN-γ	TNF	Granzyme B	IL-2
Human-CD8$^+$T cells-3	FVS	CD3	CD8	SIRPγ	TIGIT	CTLA-4	PD-1	TIM-3
Human-CD8$^+$T cells-4	FVS	CD3		SIRPγ		CD8		
Human-CD8$^+$T cells-5	FVS	CD3	CD8	SIRPγ		CD45RO	CD45RA	
Human-CD8$^+$T cells-6	FVS	CD3	CD8	SIRPγ	Granzyme B	TIM-3	IFN-γ	IL-2

The table details the fluorochrome-conjugated antibodies used for flow cytometric analysis. The specific panels correspond to the following figures: Human-macrophage cells, Human-B cells, Human-CD4$^+$T cells, Human-CD8$^+$T cells-1 were used for immune cell phenotyping in Fig. 2**d-i**, Fig S**3f-i**; Human-CD8$^+$T cells-2 and Human-CD8$^+$T cells-3 was used for T cell phenotype assays in Fig. 3**c-j**, Fig. 5**c-j**, Fig. 6**a-j**; Fig S5**a-d**, **f**, Fig S6**c-j**, Fig S7**a**, Fig S9**a**, **b**, **h-k**, Fig. S10**c-f**, Fig. S11**f**, Fig. S12**a-d**; Human-CD8$^+$T cells-4 was used for CSFE and [Ca^{2+}]i assays in Fig. 5**b**, Fig S5**e**, Fig S7**b**, Fig. S9**e**, **g**, Fig. S10**b**, **g**, Fig. S11**a** ; Human-CD8$^+$T cells-5 was used for T cell phenotype assays in Fig S7**c**, **d**; Human-CD8$^+$T cells-6 was used for T cell phenotype assays in Fig. 6**a-d**, Fig. S9**a-d**, Fig. S10**c**, **d**, Fig. S11**g**.

Supplementary Table 6 Primer sequences for qPCR

Genes	Sequence (5' to 3')
SIRPG-F	CTCCTGGTCCTTTCCTGCTTCT
SIRPG-R	AGTGCAGAGTGGCTGTCTTTCC
PHGDH-F	AGGTCCAAGCCAGCGAAAGAT
PHGDH-R	AAGATGCCCTTCCTTGTTGCG
ALDH7A1-F	ACCAAGCAGGCAGTGAGCAT
ALDH7A1-R	AGGTCCAAGCCAGCGAAAGAT
GCAT-F	AGCTGGCACTTGGAAGAGTGA
GCAT-R	ATAGCGGTACTTGTGGGCCTT
GAPDH-F	ACTCCTCCACCTTTGACGCT-3
GAPDH-R	CACCCTGTTGCTGTAGCCAAAT

Supplementary Table 7 Primer sequences for ChIP-qPCR

Genes	Sequence (5' to 3')
HAVCR2-F	TGGCTCTTATCTTCGGCTCT
HAVCR2-R	TCACGACCCAGTGTTAAAGG
PDCD1-F	TCATAAACGAGAGCTTCCTCGC
PDCD1-R	ACTGCTGTGGCCTCTTTCCA
CTLA4-F	ATCTCCAGGCAAAGCCACTGA
CTLA4-R	CCATCAGCACCACACTCACCAT
TIGIT-F	TGGAAACCTACCTACAGCGCA
TIGIT-R	CAGCCGGAGCCATTACCTTTCT
TOX-F	ACCCAGGCAATGGAGTATGTGC
TOX-R	CAAGAGAACGATCAACTAGGGACG
IRF4-F	ACACTCCTACCATAGTGGCTTCC
IRF4-R	GCTGTCCTCCCTGGTTCTTTGA
