# Supplementary material for: SIRPγ promotes CD8⁺ T cell exhaustion via metabolic-epigenetic reprogramming in tuberculous pleural effusion
Source: Nat Commun. 2026 Sep 25;17:11144. doi: 10.1038/s41467-026-77779-4 (PMC13710978; doi:10.1038/s41467-026-77779-4)
Supplement: Supplementary file 2 [file 41467_2026_77779_MOESM2_ESM.pdf]

nature portfolio

Corresponding author(s): Qiong Zhou

Last updated by author(s): Jul 31, 2026

Reporting Summary

Nature Portfolio wishes to improve the reproducibility of the work that we publish. This form provides structure for consistency and transparency in reporting. For further information on Nature Portfolio policies, see our Editorial Policies and the Editorial Policy Checklist.

Statistics

For all statistical analyses, confirm that the following items are present in the figure legend, table legend, main text, or Methods section.

n/a	Confirmed	
☐	☒	The exact sample size (*n*) for each experimental group/condition, given as a discrete number and unit of measurement
☐	☒	A statement on whether measurements were taken from distinct samples or whether the same sample was measured repeatedly
☐	☒	The statistical test(s) used AND whether they are one- or two-sided<br>*Only common tests should be described solely by name; describe more complex techniques in the Methods section.*
☐	☒	A description of all covariates tested
☐	☒	A description of any assumptions or corrections, such as tests of normality and adjustment for multiple comparisons
☐	☒	A full description of the statistical parameters including central tendency (e.g. means) or other basic estimates (e.g. regression coefficient) AND variation (e.g. standard deviation) or associated estimates of uncertainty (e.g. confidence intervals)
☐	☒	For null hypothesis testing, the test statistic (e.g. *F*, *t*, *r*) with confidence intervals, effect sizes, degrees of freedom and *P* value noted<br>*Give P values as exact values whenever suitable.*
☒	☐	For Bayesian analysis, information on the choice of priors and Markov chain Monte Carlo settings
☒	☐	For hierarchical and complex designs, identification of the appropriate level for tests and full reporting of outcomes
☐	☒	Estimates of effect sizes (e.g. Cohen's *d*, Pearson's *r*), indicating how they were calculated

Our web collection on statistics for biologists contains articles on many of the points above.

Software and code

Policy information about availability of computer code

Data collection	Flow cytometry data was collected by LSRFortessa or FACSCalibur (BD Biosciences). Quantitative real-time PCR was collected on QuantStudio 3 (Thermo Fisher Scientific).Western blotting data was acquired using a chemiluminescence imager (BioRad or Baygenebiotech).<br>sample preparation of RNA sequencing was performed according to the manufacturer's guidelines; libraries were sequenced on an Illumina NovaSeq 6000 platform (Illumina). Cytokine Array Screen was performed using the G-Series Human Cytokine Antibody Array X00 (RayBiotech, Guangzhou, China). Metabolic flux analysis was performed using an ACQUITY-I UPLC / Xevo TQS ultra-high performance liquid chromatography-triple quadrupole.
Data analysis	Flow cytometry data was analyzed by Flowjo v10. Waters MassLynx software (v4.1, Waters, Milford, MA, USA) was used to process the raw UPLC-TQMS data for peak picking, integration, identification, and quantification of each metabolite. Data analysis and plotted were using the R studio and GraphPad Prism 8.

For manuscripts utilizing custom algorithms or software that are central to the research but not yet described in published literature, software must be made available to editors and reviewers. We strongly encourage code deposition in a community repository (e.g. GitHub). See the Nature Portfolio guidelines for submitting code & software for further information.

Data

Policy information about availability of data

All manuscripts must include a data availability statement. This statement should provide the following information, where applicable:

- Accession codes, unique identifiers, or web links for publicly available datasets
- A description of any restrictions on data availability
- For clinical datasets or third party data, please ensure that the statement adheres to our policy

The raw RNA-seq, ATAC-seq, and CUT&Tag data generated in this study have been deposited in the NCBI Sequence Read Archive under accession code PRJNA1440653 (RNA-seq) and PRJNA1440899 (ATAC-seq and CUT&Tag). Direct links to these datasets are available at: https://www.ncbi.nlm.nih.gov/bioproject/PRJNA1440653/ and https://www.ncbi.nlm.nih.gov/bioproject/?term=PRJNA1440899. Fig.1a, Fig.4a, and Fig.9 were generated using BioRender under a paid subscription (agreement numbers: XY29Z56PL2, JX29Z57HKH, RK29Z54GXA). All data needed to evaluate the conclusions are present in the article, the supplementary materials and source data file provided with this paper. Further information and requests for resources and reagents should be directed to and will be fulfilled by the lead contact, Qiong Zhou (zhouqiongtj@hust.edu.cn).

Research involving human participants, their data, or biological material

Policy information about studies with human participants or human data. See also policy information about sex, gender (identity/presentation), and sexual orientation and race, ethnicity and racism.

Reporting on sex and gender

We collected 40 malignant pleural effusion (MPE) specimens (mean age: 64 years; 21 males, 19 females) and 44 tuberculous pleural effusion (TPE) specimens (mean age: 48 years; 27 males, 17 females) from the Union Hospital, Tongji Medical College, Huazhong University of Science and Technology. Additionally, we collected 69 TPE specimens (mean age: 58 years; 25 males, 44 females), 35 MPE specimens (mean age: 62 years; 24 males, 11 females), 27 parapneumonic pleural effusion (PPE) specimens (mean age: 65 years; 20 males, 7 females), and 16 miscellaneous specimens (mean age: 60 years; 6 males, 10 females) from the Wuhan Institute for Tuberculosis Prevention and Control. We compared the expression levels of SIRPγ in the supernatant of pleural effusion between males and females and found that the age of TPE patients from the Union Hospital, Tongji Medical College, Huazhong University of Science and Technology was significantly lower than that of MPE patients. In addition, we collected 60 TPE specimens (mean age: 58 years; 17 males, 43females) from the Union Hospital, Tongji Medical College, Huazhong University of Science and Technology and calculated the expression levels of SIRPγ on CD8+ T cells between males and females, but no significant differences were found.

Reporting on race, ethnicity, or other socially relevant groupings

The study did not involve race, ethnicity, or other socially relevant groupings.

Population characteristics

The clinicopathological characteristics of all patients were summarized in Table 1.

		SIRPγ+CD8+T cells-high	SIRPγ+CD8+T cells-low
Male Sex n (%)		11 (34.37%)	6 (35.29%)
Age		58.24 ± 22.11	56.83 ± 20.33
Smoke n (%)		8 (26.67%)	10 (33.33%)
Pulmonary n (%) disease		13 (40.62%)	10 (35.71%)
volume n	Low	9	10
	Medium	12	9
	High	10	10
Positive AFB n (%)		16 (57.14%)	6 (20%)
PositiveT-spot n (%)		30 (81.5%)	17 (73.91%)
Positive-Xpert MTB/RIF		18 (58.06%)	12 (38.71%)
ADA (U/L)		46.41 ± 21.18	36.80 ± 20.40
LDH (U/L)		491.9 ± 278.3	451.9 ± 338.5
Glucose (mmol/L)		7.120 ± 3.980	7.501 ± 3.200
Protein (g/L)		45.59 ± 6.523	44.03 ± 6.876
Cell counts (cells/mm3)		2251 ± 1649	3386 ± 2157
Lymphocyte (%)		72.34 ± 25.18	70.21 ± 20.88

Training Cohort in Supplementary Table 1

Parameters	TPE	MPE
Sex, male, %	61.4	52.5
Age, years	48.43 ±21.81	63.73 ±14.27
ADA, U/L	40.65 ±13.69	14.25 ±9.371
LDH, U/L	644.5 ±595.0	711.1 ±671.4
Protein, g/L	46.88 ±5.706	41.82 ±5.706
Cell counts, cells/mm3	4198 ±2663	3685 ±2114
Mononuclear cells, %	77.7 ±17.15	79.55 ±20.01

Validation Cohort in Supplementary Table 1

Parameters	TPE	MPE	PPE	Miscellaneous
Sex, male, %	71.6	67.7	74.1	35.3
Age, years	58.37±19.63	61.50 ±14.08	64.58 ±14.95	60.44 ±16.31
ADA, U/L	38.56 ±21.70	10.24 ±6.619	16.35 ±17.01	11.79 ±6.091
LDH, U/L	486.1 ±298.6	272.3 ±234.7	466.0 ±701.0	267.7 ±171.7
Protein, g/L	42.42 ±11.08	40.74 ±8.347	34.15 ±11.28	30.14 ±10.66
Cell counts, cells/mm3	2597 ±1902	4077 ±3166	5622 ±7889	685.8 ±748.6
Mononuclear cells, %	79.09 ±20.25	80.06 ±11.96	83.23 ±20.01	84.25 ±8.729

Recruitment

Participants were diagnosed with TPE if they met one of the following criteria: (1) a positive Mtb culture from pleural fluid, or (2) histological evidence of granulomatous pleurisy on closed pleural biopsy, provided no other granulomatous diseases were present. TB was diagnosed based on one or more of the following criteria: a positive Ziehl–Neelsen stain for acid-fast bacilli in sputum or pleural fluid; isolation of Mtb from sputum specimens by positive culture on Löwenstein–Jensen medium; or confirmation by histopathological examination. A diagnosis of MPE was established by demonstration of malignant cells in PE and/or on closed pleural biopsy specimen. PPE was those with a glucose concentration >3.3 mmol/L and pH >7.2, and no organisms seen on Gram stain or found on PE culture in a patient with pneumonia. PE were classified as Miscellaneous on the basis of Light's criteria.
Our stringent criteria requiring invasive biopsy or culture positivity may have introduced selection bias, excluding patients too unstable for procedures or those with culture-negative early-stage disease. Thus, our findings are primarily generalizable to microbiologically or histopathologically confirmed cases and should be extrapolated to the broader exudative effusion population with caution.

Ethics oversight

All procedures involving human samples adhered to the ethical standards outlined in the Declaration of Helsinki. All participants provided written informed consent. The study protocol was approved by Wuhan Union Hospital ethics committee and Wuhan Pulmonary Hospital ethics committee (Ethics protocol approval number: 2020-43, 2021-0663, and 2025-0404).

Note that full information on the approval of the study protocol must also be provided in the manuscript.

Field-specific reporting

Please select the one below that is the best fit for your research. If you are not sure, read the appropriate sections before making your selection.

[x] Life sciences [ ] Behavioural & social sciences [ ] Ecological, evolutionary & environmental sciences

For a reference copy of the document with all sections, see nature.com/documents/nr-reporting-summary-flat.pdf

Life sciences study design

All studies must disclose on these points even when the disclosure is negative.

Sample size

Sample size was determined based on statistical requirements and previous published studies using similar methodologies in this field (ref l,2,3). All sample sizes are listed in the corresponding figure legends or on the figures. All experiments were repeated at least three times.
1.Wang W, Zhou Q, Zhai K, Wang Y, Liu JY, Wang XJ, Wang Z, Zhang JC, Tong ZH, Shi HZ. Diagnostic accuracy of interleukin 27 for tuberculous pleural effusion: two prospective studies and one meta-analysis. Thorax. 2018 Mar;73(3):240-247.
2.Ye L, Xiang X, Wang Z, Zhang S, Xue Q, Wei X, Liu Y, Wang H, Ai J, Yang B, Chen L, Niu Y, Peng W, Zhou Q. H3K18 lactylation in cancer-associated fibroblasts drives malignant pleural effusion progression via TNFR2+ Treg recruitment. Exp Mol Med. 2025 Nov;57(11):2671-2685.
3. Cheng H, Ji Z, Wang Y, Li S, Tang T, Wang F, Peng C, Wu X, Cheng Y, Liu Z, Ma M, Wang J, Huang X, Wang L, Qin L, Liu H, Chen J, Zheng R, Feng CG, Cai X, Qu D, Ye L, Yang H, Ge B. Mycobacterium tuberculosis produces D-serine under hypoxia to limit CD8+ T cell-dependent immunity in mice. Nat Microbiol. 2024 Jul;9(7):1856-1872.

Data exclusions

No data was excluded from the study.

Replication

All the experiments were repeated for 3 times and got consistent results.

Randomization

Samples were randomly allocated into the study.

Blinding

During all statistical analyses, the data analyst remained blinded to the TPE, MPE, PPE, and Miscellaneous conditions as well as the treatment groups. The investigators responsible for sample collection, drug administration, and initial data collection were necessarily aware of the group assignments in order to properly execute the relevant procedures. However, during the data collection and analysis process, the investigators remained blinded to the group allocations to eliminate any intentional or unintentional bias.

Reporting for specific materials, systems and methods

We require information from authors about some types of materials, experimental systems and methods used in many studies. Here, indicate whether each material, system or method listed is relevant to your study. If you are not sure if a list item applies to your research, read the appropriate section before selecting a response.

Materials & experimental systems

n/a	Involved in the study
	☒ Antibodies
	☒ Eukaryotic cell lines
☒	☐ Palaeontology and archaeology
☒	☐ Animals and other organisms
☒	☐ Clinical data
☒	☐ Dual use research of concern
☒	☐ Plants

Methods

n/a	Involved in the study
☒	☐ ChIP-seq
	☒ Flow cytometry
☒	☐ MRI-based neuroimaging

Antibodies

Antibodies used

For flow cytometry:
Fixable Viability Stain 780 (BD Pharmingen, Cat# 565388)
Human TruStain FcXTM (Biolegend, Cat# 422302)
https://www.bdbiosciences.com/en-us/products/reagents/flow-cytometry-reagents/research-reagents/single-color-antibodies-ruo/fixable-viability-stain-780.565388
Brilliant Violet 510TM anti-human CD45 Antibody (Biolegend, Cat# 304036)
https://www.biolegend.com/en-gb/products/brilliant-violet-510-anti-human-cd45-antibody-8006?GroupID=BLG5926
PerCP/Cyanine5.5 anti-human CD3 (Biolegend, Cat# 317336)
https://www.biolegend.com/en-gb/products/percp-cyanine5-5-anti-human-cd3-antibody-8220?GroupID=BLG4203
FITC anti-human CD8a (Biolegend, Cat# 301050)
https://www.biolegend.com/en-gb/products/fitc-anti-human-cd8a-antibody-834?GroupID=BLG5903
PE/Cyanine7 anti-human CD8a (Biolegend, Cat# 301012)
https://www.biolegend.com/en-gb/products/pe-cyanine7-anti-human-cd8-antibody-6390?GroupID=BLG10167
FITC anti-human CD4 (Biolegend, Cat# 300538)
https://www.biolegend.com/en-gb/products/fitc-anti-human-cd4-antibody-825?GroupID=BLG5901
FITC anti-human CD68 (Biolegend, Cat# 333806)
https://www.biolegend.com/en-gb/products/fitc-anti-human-cd68-antibody-4844?GroupID=BLG10058
APC anti-human CD19 (Biolegend, Cat# 982406)
https://www.biolegend.com/en-gb/products/apc-anti-human-cd19-antibody-14024?GroupID=BLG15288
PE/Cyanine7 anti-human CD56 (Biolegend, Cat# 985912)
https://www.biolegend.com/en-gb/products/pe-cyanine7-anti-human-cd56-ncam-antibody-3802?GroupID=BLG15664
PE anti-human CD172g (SIRPγ) (Biolegend, Cat# 336606)
https://www.biolegend.com/en-gb/products/pe-anti-human-cd172g-sirpgamma-antibody-5354?GroupID=GROUP28
Brilliant Violet 421™ anti-human CD279 (PD-1) (Biolegend, Cat# 329920)
https://www.biolegend.com/en-gb/products/brilliant-violet-421-anti-human-cd279-pd-1-antibody-7191?GroupID=BLG5466
APC anti-human TIGIT (VSTM3) (Biolegend, Cat# 372706)
https://www.biolegend.com/en-gb/products/apc-anti-human-tigit-vstm3-antibody-13758?GroupID=BLG15734
Brilliant Violet 510™ anti-human CD366 (TIM-3) (Biolegend, Cat# 345030)
https://www.biolegend.com/en-gb/punchout/products/product-detail/brilliant-violet-510-anti-human-cd366-tim-3-antibody-12009
PE/Cyanine7 anti-human CD152 (CTLA-4) (Biolegend, Cat# 349914)
https://www.biolegend.com/de-de/products/pe-cyanine7-anti-human-cd152-ctla-4-antibody-9294?GroupID=BLG10049
APC anti-human IFN-γ (Biolegend, Cat# 502512)
https://www.biolegend.com/en-gb/products/apc-anti-human-ifn-gamma-antibody-1012?GroupID=BLG10006
PE/Cyanine7 anti-human TNF-α (Biolegend, Cat# 502930)
https://www.biolegend.com/en-gb/products/pe-cyanine7-anti-human-tnf-alpha-antibody-6515?GroupID=GROUP24
Brilliant Violet 510™ anti-human IL-2 (Biolegend, Cat# 500338)
https://www.biolegend.com/en-us/products/brilliant-violet-510-anti-human-il-2-antibody-8948
Brilliant Violet 421™anti-human/mouse Granzyme B (Biolegend, Cat# 515410)
https://www.biolegend.com/en-gb/products/brilliant-violet-421-anti-humanmouse-granzyme-b-recombinant-antibody-17577?GroupID=GROUP28
Ultra-LEAF™ Purified anti-human CD28 Antibody (Biolegend, Cat# 302934)
https://www.biolegend.com/en-gb/products/ultra-leaf-purified-anti-human-cd28-antibody-7743?GroupID=BLG10175
Ultra-LEAF™ Purified anti-human CD3 Antibody (Biolegend, Cat# 317326)
https://www.biolegend.com/en-gb/products/ultra-leaf-purified-anti-human-cd3-antibody-7745?GroupID=BLG4203

Western blotting:
Anti-Histone H3 (Abcam, Cat# ab12079, 1:1500)
https://www.abcam.com/en-us/products/primary-antibodies/histone-h3-antibody-mabcam-10799-nuclear-marker-and-chip-grade-ab10799
Tri-Methyl-Histone H3 (Lys4)/H3K4me3 Antibody (Affinity, Cat# DF6935, 1:1500)
https://www.affbiotech.com/goods-5737-DF6935-Tri_Methyl_Histone_H3_Lys4_H3K4me3_Antibody.html
Tri-Methyl-Histone H3 (Lys9)/H3K9me3 Antibody (Affinity, Cat# DF6938, 1:1500)
https://www.affbiotech.com/goods-5740-DF6938-Tri_Methyl_Histone_H3_Lys9_H3K9me3_Antibody.html

Anti-Trimethyl-Histone H3 (Lys27) Rabbit mAb (PTM Bio, Cat# PTM-647RM, 1:1500)
https://ptmbio.com/products/anti-tri-methyl-histone-h3-lys27-rabbit-mab/PTM-647RM.htm
SIRPG Antibody (Affinity, Cat# DF4508, 1:1500)
https://www.affbiotech.com/goods-3636-DF4508-SIRPG_Antibody.html
PHGDH Polyclonal antibody (proteintech, Cat# 14719-1-AP, 1:1500)
https://www.ptglab.com/Products/PHGDH-Antibody-14719-1-AP.htm
Anti-IRF4 Rabbit pAb (ABclonal, Cat# A0524, 1:1000)
https://abclonal.com/catalog-antibodies/IRF4RabbitpAb/A0524
Anti-TOX Antibody (Abcam, Cat# ab237009, 1:1500)
https://www.abcam.com/en-us/products/primary-antibodies/tox-antibody-nan448b-ab237009
Anti-NFATC2 Antibody (Abcam, Cat# ab283691, 1:1000)
https://www.abcam.com/en-us/products/primary-antibodies/nfatc2-antibody-ab327205
mTOR Antibody (ABclonal, Cat# A11355, 1:1500)
https://abclonal.com/catalog-antibodies/mTORRabbitpAb/A11355
Phospho-mTOR (Ser2448) Antibody (ABclonal, Cat# AP0115 1:1000)
https://abclonal.com.cn/catalog/AP0115
PI3-kinase p85-alpha/gamma pAb (Abmart, Cat# T40064, 1:1000)
https://www.ab-mart.com.cn/page.aspx?node=%2077%20&id=%201221
Phospho-PI3-kinase p85- alpha/ gamma (Tyr467/199) pAb (Abmart, Cat# T40116 , 1:1000)
https://www.ab-mart.com.cn/page.aspx?node=%2077%20&id=%2049664
AKT1/2/3 Antibody (Abmart, Cat# T55561, 1:1000)
https://www.ab-mart.com.cn/page.aspx?node=%2077%20&id=%201801
Phospho-pan-AKT1/2/3 (Ser473) Antibody (Abmart, Cat# TA0016, 1:1000)
https://www.ab-mart.com.cn/page.aspx?node=%2077%20&id=%2022212
β-Actin Antibody (Cell Signaling Technology (CST), Cat# 4967, 1:3000)
https://www.cellsignal.com/products/primary-antibodies/beta-actin-antibody/4967

For IF and IHC:
SIRPG Antibody (Affinity, Cat# DF4508, 1:200)
https://www.affbiotech.com/goods-3636-DF4508-SIRPG_Antibody.html
PHGDH Polyclonal antibody (proteintech, Cat# 14719-1-AP, 1:200)
https://www.ptglab.com/Products/PHGDH-Antibody-14719-1-AP.htm
anti-CD4 Antibody (Abcam, Cat# ab133616, 1:100)
https://www.abcam.cn/products/primary-antibodies/cd4-antibody-epr6855-ab1336166
anti-CD8 Antibody (Abcam, Cat# ab237709, 1:100)
https://www.abcam.cn/products/primary-antibodies/cd4-antibody-epr6855-ab1336166
anti-CD68 Antibody (Abcam, Cat# ab283654, 1:100)
https://www.abcam.cn/products/primary-antibodies/cd68-antibody-epr23917-164-ab283654

Validation

For flow cytometry, western blot, immunohistochemistry and Immunofluorescence, all antibodies were validated before first usage. This validation was based on the manufacturers and the results have been provided in online databases on the manufacturers' website.

Eukaryotic cell lines

Policy information about cell lines and Sex and Gender in Research

Cell line source(s)	Human embryonic kidney (HEK) 293T cells (American Type Culture Collection, VA, # AT-1067) were cultured in RPMI-1640 medium (Gibco, # 11875119) containing 10% Fetal Bovine Serum (Gibco, # A5670701), penicillin-streptomycin (100 U/mL and 100 µg/mL), 2 mM L-glutamine, and 10 mM HEPES.
Authentication	STR testing.
Mycoplasma contamination	All cell lines tested negative for mycoplasma contamination.
Commonly misidentified lines (See ICLAC register)	None commonly misidentified cell lines were used.

Plants

Seed stocks	n/a
Novel plant genotypes	n/a
Authentication	n/a

Flow Cytometry

Plots

Confirm that:

- [x] The axis labels state the marker and fluorochrome used (e.g. CD4-FITC).
- [x] The axis scales are clearly visible. Include numbers along axes only for bottom left plot of group (a 'group' is an analysis of identical markers).
- [x] All plots are contour plots with outliers or pseudocolor plots.
- [x] A numerical value for number of cells or percentage (with statistics) is provided.

Methodology

Sample preparation	Following collection via standard thoracentesis from TPE patients (within 24 hr of hospitalization) and peripheral blood draw (10 mL from TPE/TB patients), all specimens were immediately placed on ice. After centrifugation (1,500 × g, 6 min), the supernatants (from pleural effusions) and serum were aliquoted and stored at −80°C. Mononuclear cells were then isolated from all specimens resuspended in 1×phosphate-buffered saline (PBS) using a Ficoll-Hypaque density gradient (StemCell Technologies, Vancouver, Canada).Immune cells were resuspended in staining buffer and incubated with Fc receptor blocking reagent (BioLegend) for 10 minutes. Surface markers were then stained with fluorochrome-conjugated antibodies (30 minutes, 4°C, dark). Next, cells were fixed and permeabilized using Fixation/Permeabilization solution (eBioscience; 1:3 concentrate: diluent ratio for 30 minutes at 4°C in darkness, and washed twice with 1× permeabilization buffer. Intracellular staining was then performed using specific antibodies under the same conditions (30 minutes, 4°C, dark). Cells were finally fixed in stabilization buffer and acquired on a BD flow cytometer. For cytokine detection in $CD8^+$ T cells, samples were pre-stimulated with Cell Activation Cocktail (BioLegend) for 4–6 hours prior to staining. Data analysis was performed using FlowJo software (BD Biosciences).
Instrument	flow cytometry (FACS Canto II; BD Bioscience); cell sorting (BD FACSAria II Cell Sorter).
Software	The data was then analyzed using FlowJo software (BD Biosciences).
Cell population abundance	The abundance is depended on the specific population, from 0%-90%. Purified CD8+ T cells were isolated with the human CD8+ T cells isolation kit (STEMCELL Technologies) according to the manufacturer's protocol. The purity of CD8+ T cells was > 90%, as confirmed by flow cytometry.
Gating strategy	The FSC/SSC gates represent the starting cell population, followed by gating on Fixable Viability Dye-negative and CD45-positive cells. Next, we gated on CD3-positive and CD8-positive cells to define CD8+ T cells. CD4+ T cells, macrophages, NK cells, and B cells were defined as CD3+CD4+, CD3−CD68+, CD3−CD56+, and CD3−CD19+, respectively. The cell clusters were distinct, allowing us to easily distinguish the boundary between positive and negative populations.

- [x] Tick this box to confirm that a figure exemplifying the gating strategy is provided in the Supplementary Information.
