# Supplementary material for: SIRPγ promotes CD8⁺ T cell exhaustion via metabolic-epigenetic reprogramming in tuberculous pleural effusion
Source: Nat Commun. 2026 Sep 25;17:11144. doi: 10.1038/s41467-026-77779-4 (PMC13710978; doi:10.1038/s41467-026-77779-4)
Supplement: Supplementary file 3 [file 41467_2026_77779_MOESM3_ESM.pdf]

nature portfolio

Peer Review File

SIRPγ Promotes $CD8^+$ T Cell Exhaustion via Metabolic-Epigenetic Reprogramming in Tuberculous Pleural Effusion

Corresponding Author: Professor Qiong Zhou

This file contains all reviewer reports in order by version, followed by all author rebuttals in order by version.

Version 0:

Reviewer comments:

Reviewer #1

(Remarks to the Author)
In this manuscript Yang and colleagues present their data on the potential diagnostic value of the signaling molecule SIRPγ in Tuberculous Pleural effusion (TPE). The authors suggest that the SIRPγ likely promotes the CD8+ T cell exhaustion in TPE and provide some mechanistic details therein. They identified SIRPγ as a differentially expressed gene, via a high throughput antibody microarray analysis between 50 MPE and 50 TPE human samples and show that SIRPγ enhances the diagnostic value of adenosine deaminase (ADA), the current diagnostic standard for TPE. SIRPγ is expressed in in both CD4+ and CD8+ cells in TPE, while its blockade via a monoclonal antibody, mitigates the exhausted-like CD8+ T cell characteristic gene expression and enhanced their proliferative capacity. By focusing on the metabolic differences between SIRPγ+ and SIRPγ- CD8+ T cells, the authors find that the serine metabolism and the serine intracellular levels are increased in SIRPγ expressing CD8+ T cells and that SIRPγ downstream signaling upregulates PHGDH expression, the first gene in serine biosynthetic pathway in CD8+ T cells. A PHGDH inhibitor as well as, the serine cell starvation reduces the endogenous serine levels and the exhaustion marker transcription co-inhibitory receptors CIRs- CTLA-4, TIM-3, PD-1, and TIGIT), while it restores the proliferative capacity of CD8+ T cells. Furthermore, the serine starvation promotes the production of effector cytokines such as IL-2, TNF-α, Granzyme B, and IFN-γ, in $CD8^+$ T cells. The authors then show that from NADPH, GSH and S-adenosyl methionine-SAM, the three major serine pathway molecular products, only SAM (comprising the universal methyl donor for the cell methylation reactions), supplementation reversed the serine deprivation–induced exhaustion. Next, the authors perform ATAC-seq in SAM-treated or untreated CD8+ T cells and find that SAM increases the chromatin accessibility of the CIR gene loci as well as, the accessibility of transcription factor loci, associated with T-cell exhaustion- among them the members of the NFAT family. The authors then show that SAM treatment increases increased the H3K4 global methylation and that NFAT2 expression levels are elevated in SAM-treated cells. Finally, the $CD8^+$ T cells isolated from TPE, are treated with recombinant CD47 protein and test positive for PHGDH, p-PI3K, p-AKT, and p-mTOR increased signaling.
Major Concerns:
Manuscript line 54-46: “Mechanistically, SIRPγ interacted with CD47 to activate the PI3K/AKT/mTOR pathway, leading to the upregulation of phosphoglycerate dehydrogenase (PHGDH) and the enhancement of serine metabolism”. Since, the CD47 gene is expressed in T cells where its expression is crucial for their survival, function and for the maintenance of immune homeostasis, it is not clear whether the CD47-SIRPγ interaction takes place between the same cells (eg. CD8+ T cells) or across cell types (for example, between CD8+ T cells and Tregs). The authors should comment on how they envision this interaction to take place in the context of TPE and what would be the functional outcome of such interaction(s).
Manuscript line 70: “However, TPE is difficult to diagnose”. According to the current literature, adenosine deaminase (ADA), interferon-gamma (IFN-γ), and interleukin-27 (IL-27), all show high diagnostic accuracy for TPE while, ADA has been extensively studied as a biochemical marker in the TPE pleural fluid. Additionally, according to PMID: 20345583, this test is simple, cheap, rapid, minimally invasive, and can be performed in most laboratories. Thus, the introduction of SIRPγ as a diagnostic marker for TPE, especially when it is conferring only a slight increase in the diagnostic value in combination with ADA, is not immediately apparent. The authors should provide their suggestion on how they envision the usage of SIRPγ as a diagnostic tool in the current clinical practice.
Manuscript line 168: “The result revealed that SIRPγ was primarily expressed in T cells and NK cells (Fig. 2A–C, Fig. S2A–C)”. The SIRPγ is expressed in both CD4+ and CD8+ cells in TPE. However, its expression is not different between PB derived and TPE derived CD4+ T cells. In contrast the SIRPγ expression is different between the PB derived and TPE derived CD8+ T cells. Given that, the role of CD8+ T cells in TPE is unknown, the physiological relevance, for TPE pathology is difficult to be extracted based on these data. The authors should provide the physiological context within which,

the exhausted as well as, the other CD8+ T cell clusters (the memory clusters) operate within the TPE. Towards that end, they could also address the kinetics or pseudotime of SIRPγ expression among these clusters, in addition to that CD4+ T cells in TPE.
An additional concern is that, the exhausted TPE CD8+ T cell cluster, is very small in comparison to the other clusters. It is unlikely that the diagnostic value of the SIRPγ in TPE resides entirely with this cell population. The physiological significance of the exhausted CD8+ T cell population with regards to the TPE pathology is also unclear. The authors should address these issues at least within the discussion of their results.
The authors identify metabolic differences between SIRPγ+ and SIRPγ- CD8+ T cells by testing gene expression levels. However, the dynamics of the metabolic rewiring of a given cell type are unlikely to be regulated at the transcription level as they require a faster and dynamic type of regulation. A complementary approach would be the application of SCENITH assay into the TPE- derived CD8+ T cells.
Manuscript line 299: “Notably, co-treatment with exogenous SG depletion with PHGDH-IN-3 synergistically attenuated SIRPγ+CD8$^+$ T cell exhaustion, surpassing the effects of PHGDH inhibition alone”. This statement means that serine deprivation alleviates T-cell exhaustion. However, later, in the manuscript line 320 the authors state that “To counteract the exhaustion phenotype induced by SG deprivation, cultures were supplemented with NADPH, GSH, or SAM”. This sentence means that SG deprivation drives T-cell exhaustion. Furthermore, in manuscript lines 322-325 they state: “Notably, only SAM supplementation reversed SG deprivation–induced exhaustion, as evidenced by increased immunosuppressive receptors (CTLA-4, TIM-3, PD-1, TIGIT), reduced levels of cytokines (IL-2, TNF-α, IFN-γ, Granzyme B) and impaired proliferative capacity in SIRPγ$^+$CD8$^+$ T cells (Fig. 6A, B, S9A)”. Nevertheless, these characteristics represent the hallmarks of T-cell exhaustion and not the alleviation of exhaustion. The authors should thoroughly screen their text for such contradictions in their data interpretation.
Manuscript line 351: “To explore the epigenetic regulatory role of SAM, genome-wide chromatin accessibility in SAM-treated CD8$^+$ T cells was initially assessed using ATAC-seq”. The authors use for this work CD8$^+$ T cells coming from the following sources: a) Peripheral Blood Mononuclear Cells (PBMCs) collected from three groups: 1) PPD-positive healthy volunteers, 2) Patients with tuberculous pleural effusion (TPE), 3) Patients with active pulmonary tuberculosis (TB) b) Pleural Effusion-Derived Mononuclear Cells (PEMCs): 1) PEMCs were obtained from pleural effusion samples of TPE patients. These cells were used for various experimental assays, including flow cytometry, RNA sequencing, ELISA, proliferation assays, ATAC-SEQ and other functional and molecular analyses. It is mandatory that the authors should carefully state the source of these CD8$^+$ T cells used in their particular assays in detail as well as, clearly state the rationale behind their choice of one cell source over another.
Manuscript lines 403-408: “Consistent with these observations, SAM-treated CD8$^+$ T cells displayed elevated NFATC2 expression, accompanied by transcriptional upregulation of inhibitory receptors and exhaustion-associated transcription factors, including TOX, BATF, and IRF4. We therefore propose that SAM promotes CD8+T cell exhaustion by enhancing H3K4me3 modification at the NFATC2 promoter, facilitating expression of immunosuppressive receptors and exhaustion-related transcription factors (TOX and IRF4)”. As NFAT transcription factors are subject to calcium-dependent activation, it is unlikely that the transcriptional de-repression of this genetic locus, only driven by a transcriptionaly favorable epigenetic modification could result in a significant change in the transcriptomic profile of the cell. Despite the authors considerable efforts to back these data up with in vitro assays, the mechanistic evidence for SAM driving CD8+T cell exhaustion is rather circumstantial. The authors should comment on a possible over interpretation of their data.
Minor Concerns:
Manuscript line 159: “Collectively, support…”. The authors should rephrase as the current statement does not make sense.
Manuscript line 182: “Clusters 0, 2, 4, 5, 6, 11, and 12 represented naïve-like T cells (TN), characterized by CCR7, SELL, TCF7, LEF1, and MALL”. How naïve-like T cells are found amidst a fulminating inflammatory reaction? Are all these cells CD45RA+?
Manuscript line 193: “To address this, freshly isolated PBMCs from PPD$^+$ donors were stimulated with the recombinant fusion antigen ESAT-6–CFP-10 (E195 C)”. Please explain the “PPD+” acronym as well as, where the ESAT-6–CFP-10 is derived from and the rationale behind it’s choice.
Manuscript line 204: “…implicating a potential role for SIRPγ in promoting CD8$^+$ T-cell exhaustion via tuberculosis antigens”. I believe it is the other way around: “Implying a potential role for tuberculosis antigens in promoting CD8$^+$ T-cell exhaustion via SIRPγ”.
Manuscript line 209: “…scRNA-seq were categorized into three functional group”, should read “…scRNA-seq were categorized into three functional groups”. The authors should thoroughly scrutinize and edit their text for typos and poorly formed sentences.
Suppl Fig 1A: the analysis is very poor and the lettering too small to read particularly the gene list
Suppl Fig 1B: CD172G is not among the highest overexpressing genes in this screening but it is the only one marked. The authors should provide information about the other genes on this volcano plot.
Figure 1B: the analysis is very poor and the lettering too small
Figure 1E: is mislabeled as 1D
Figure 2L: the analysis is very poor and the lettering too small
Figure 3B: the analysis is very poor and the lettering too small

Reviewer #2

(Remarks to the Author)
This is an interesting manuscript that provides evidence that signal regulatory protein γ (SIRPγ) can be used as a novel biomarker to distinguish tuberculous pleural effusion (TPE) from other types of pleural effusions. The authors also demonstrate that SIRPγ is involved in TPE pathogenesis through induction of CD8$^+$ T cell exhaustion. They demonstrate that SIRPγ interacts with CD47 to activate the PI3K/AKT pathway, leading to the upregulation of phosphoglycerate

dehydrogenase (PHGDH) and enhancement of serine metabolism, which in turn facilitates SAM-dependent trimethylation of histone H3 lysine 4 (H3K4me3) epigenetic modification, ultimately leading to the expression of exhaustion-associated transcription factors and immunosuppressive receptors. The data are generally of value and may have some clinical translational implications. In general the methodological approaches are appropriate and the studies well done. However, there are some issues that require attention, as outlined below.

1.Figures 2 I and J. These are very important data as demonstrate the differential expression of SIRPγ on the CD8 cells in TPE versus blood. As SIRPγ is also expressed on CD4 cells (Fig. 2D), have the authors examined the same paired clinical samples for SIRPγ expression on CD4 cells as well?

2.The data in Figures 3 and S3/S4 are convincing and establish a correlation between SIRPγ expression on CD8 cells and inhibitory receptors expression and increased expression of exhaustion markers; as well as reduced cytokine production in paired samples from TPE patients. The authors have also excluded a series of other clinical correlations, as summarized in Table 1. One set of parameters that is not indicated in Table 1 is whether some of the patients were on active anti-TB treatment at the time of the studies and whether there was any correlation between anti-TB drugs and T cell exhaustion markers. If these clinical data are available, some additional analysis may help to further solidify the conclusions.

3.In the legend for Figure 4, it is stated "Statistical significance was determined by unpaired two-tailed Student's t-test (C, D, F, H, K) and paired two-tailed t-tests (K)". So, for panel K, which t-test was used unpaired or paired t-test? Also, based on what criteria was used unpaired versus paired in the different panels?

Reviewer #3

(Remarks to the Author)
This study investigated the role of signal regulatory protein gamma (SIRPy) in tuberculous pleural effusion (TPE). The authors first identified SIRPy as a novel diagnostic biomarker through high-throughput antibody microarray screening, demonstrating its elevated expression in TPE compared to malignant pleural effusion (MPE). The authors propose that SIRPy interacts with CD47 to activate the PI3K/AKT/mTOR pathway, upregulates phosphoglycerate dehydrogenase (PHGDH), enhances serine metabolism, and promotes S-adenosylmethionine (SAM)-dependent H3K4me3 modifications. This cascade ultimately induces NFATC2 expression, driving exhaustion-associated transcription factorsand immunosuppressive receptors. While the study presents interesting findings, several methodological and interpretive issues require clarification and additional experimental evidence.
1. The protein marker screening and evaluation of SIRPy's diagnostic performance were conducted solely between TPE and MPE, while parapneumonic pleural effusion (PPE)—one of the three most common causes of exudative pleural effusions—was not included. It is essential to incorporate PPE samples to clarify whether elevated SIRPy expression is specific to TPE.
2. The authors showed that Fig. 1N displays H&E and SIRPy IHC staining of TPE lesion tissue and adjacent non-tuberculous tissue. However, the provided images lack sufficient resolution to discern cellular and tissue architecture clearly. From what is visible, the TPE lesion area does not show typical granulomatous structures, raising questions about whether it represents a classic tuberculous lesion. The adjacent non-lesion area shows extensive fibrous exudate with only scattered cells, suggesting low cellularity. How was SIRPy expression quantified in such a paucicellular region? The Methods section does not specify the number of cases included in the histological analysis or the detailed protocol for SIRPy IHC evaluation. Furthermore, based on the H&E and IHC results, in which specific immune cell types within the tuberculous lesion or its periphery, was high SIRPy expression observed?
3. In lines 156–160, the authors suggest that high SIRPy expression in tissue specimens indicates a greater likelihood of microbiological confirmation. What evidence supports this conclusion? It remains unclear whether such a correlation genuinely exists, particularly as the histopathological results in Fig. 1N are not definitive.
4. The authors compare SIRPy expression in TPE versus peripheral blood from tuberculosis patients (lines 169–173, Fig. 2) and report higher expression in CD8+ T cells from TPE. Was SIRPy expression also compared across different immune cell subsets between TPE and MPE? Is SIRPy expression in CD8+ T cells significantly higher in TPE than in MPE? Additionally, what is the expression level of SIRPy in exhausted T cells (Tex) from MPE patients?
5. In lines 189–205, the authors describe in vitro stimulation of PBMCs from PPD+ donors with ESAT-6-CFP-10 antigen and observe SIRPy-promoted CD8+ T cell exhaustion. However, the duration of antigen stimulation is not specified in the Methods. Typically, such experiments do not exceed 24 hours. How can the observed effect be attributed to "chronic stimulation" when a short-term stimulus was likely used? Would "immediate stimulation" be a more accurate description?
6. Through differential gene expression analysis, the authors identified associations between SIRPy-mediated T cell exhaustion and glycine, serine, and threonine metabolism pathways (lines 239–241). However, only the serine metabolism pathway was pursued experimentally. What is the rationale for focusing exclusively on serine metabolism, and were other relevant pathways explored?
7. The study lacks direct experimental evidence supporting SIRPy as a therapeutic target. It is recommended that claims regarding SIRPy's potential as a therapeutic target be removed from the title, abstract, and conclusion sections to maintain scientific rigor.

Version 1:

Reviewer comments:

Reviewer #1

(Remarks to the Author)
Yang and colleagues have satisfactorily addressed all comments from the initial review. The revised manuscript is significantly improved, both in terms of presentation and scientific content. I have no major concerns and recommend acceptance upon minor revisions and thorough text editing.

Minor concerns:
Line 218: “the molecular mechanism…” should start with capital letter “The molecular mechanism…”
Line 228: “consistent with previous finding…” should read either “consistent with a previous finding…” or “consistent with previous findings…”
Line 232: the word “implicating” should be replaced by the word “implying”
Line 358: “To counteract the exhaustion phenotype reduced by SG deprivation…” should be rephrased into “To bring back the exhaustion phenotype which is reduced by SG deprivation…” or “To rescue the exhaustion phenotype reduced by SG deprivation…
Line 389: “However, the precise role of SAM in modulating histone methylation within $CD8^+$ T cells remain incompletely understood” should read “However, the precise role of SAM in modulating histone methylation within $CD8^+$ T cells remains incompletely understood”

Reviewer #2

(Remarks to the Author)

Reviewer #3

(Remarks to the Author)
1. Based on the IHC images presented in Fig. 1N and Fig. S2A, SIRPγ staining appears more evident in multinucleated giant cells and epithelioid cells, whereas it appears relatively weak in lymphocytes. In Fig. S2B, only about four cells exhibit clear co-localization of SIRPγ and CD3+ signals, with the majority of SIRPγ-positive cells showing no overlap with CD3+ labeling. Given these observations, how do the authors justify the conclusion that SIRPγ is specifically and highly expressed in CD3+ T cells?
2. The acid-fast staining and IHC images in Fig. 1N do not appear to represent the same tissue region. In the current Fig. 1N, an increase in SIRPγ-positive lymphocytes within acid-fast-positive granulomas is not clearly seen. Authors should provide WSIs—including HE staining, IHC staining, and acid-fast staining, along with corresponding magnified views—for both acid-fast-positive and acid-fast-negative lesions.
3. How were the samples for the cytometric experiments obtained? The results shown in Figure S3F and S3G indicate that SIRPγ expression in macrophages is notably low. However, the IHC staining results suggest that SIRPγ-positive signals are predominantly localized to multinucleated giant cells and epithelioid cells.

Version 2:

Reviewer comments:

Reviewer #3

(Remarks to the Author)
The conclusion "SIRPγ is predominantly expressed on T cells and weakly on macrophages" remains unsupported by the data. The HE, IHC, and immunofluorescence results are insufficient to distinguish expression levels between these cell types, and several images do not match the descriptions, with poor quality.

Open Access This Peer Review File is licensed under a Creative Commons Attribution 4.0 International License, which permits use, sharing, adaptation, distribution and reproduction in any medium or format, as long as you give appropriate credit to the original author(s) and the source, provide a link to the Creative Commons license, and indicate if changes were made.
In cases where reviewers are anonymous, credit should be given to 'Anonymous Referee' and the source.
The images or other third party material in this Peer Review File are included in the article's Creative Commons license, unless indicated otherwise in a credit line to the material. If material is not included in the article's Creative Commons license and your intended use is not permitted by statutory regulation or exceeds the permitted use, you will need to obtain permission directly from the copyright holder.
To view a copy of this license, visit https://creativecommons.org/licenses/by/4.0/

Response to Reviewers

Dear reviewers,

Thank you very much for your comments and professional advice. These opinions help to improve academic rigor of our article. Based on your suggestions, we have carefully revised the manuscript and provided the point-by-point response below. All modifications in the manuscript have been highlighted in yellow. If any responses are unclear or you wish additional changes, please do not hesitate to contact us.

Reviewer #1 (Remarks to the Author):

In this manuscript Yang and colleagues present their data on the potential diagnostic value of the signaling molecule SIRPγ in Tuberculous Pleural effusion (TPE). The authors suggest that the SIRPγ likely promotes the CD8+ T cell exhaustion in TPE and provide some mechanistic details therein. They identified SIRPγ as a differentially expressed gene, via a high throughput antibody microarray analysis between 50 MPE and 50 TPE human samples and show that SIRPγ enhances the diagnostic value of adenosine deaminase (ADA), the current diagnostic standard for TPE. SIRPγ is expressed in in both CD4+ and CD8+ cells in TPE, while its blockade via a monoclonal antibody, mitigates the exhausted-like CD8+ T cell characteristic gene expression and enhanced their proliferative capacity. By focusing on the metabolic differences between SIRPγ+ and SIRPγ- CD8+ T cells, the authors find that the serine metabolism and the serine intracellular levels are increased in SIRPγ expressing CD8+ T cells and that SIRPγ downstream signaling upregulates PHGDH expression, the first gene in serine biosynthetic pathway in CD8+ T cells. A PHGDH inhibitor as well as, the serine cell starvation reduces the endogenous serine levels and the exhaustion marker transcription co-inhibitory receptors CIRs- CTLA-4, TIM-3, PD-1, and TIGIT), while it restores the proliferative capacity of CD8+ T cells. Furthermore, the serine starvation promotes the production of effector cytokines such as IL-2, TNF-α, Granzyme B, and IFN-γ, in $CD8^+$ T cells. The authors then show that from NADPH, GSH and S-adenosyl methionine-SAM, the three major serine pathway molecular products, only SAM (comprising the

universal methyl donor for the cell methylation reactions), supplementation reversed the serine deprivation–induced exhaustion. Next, the authors perform ATAC-seq in SAM-treated or untreated CD8+ T cells and find that SAM increases the chromatin accessibility of the CIR gene loci as well as, the accessibility of transcription factor loci, associated with T-cell exhaustion- among them the members of the NFAT family. The authors then show that SAM treatment increases increased the H3K4 global methylation and that NFAT2 expression levels are elevated in SAM-treated cells. Finally, the $CD8^+$ T cells isolated from TPE, are treated with recombinant CD47 protein and test positive for PHGDH, p-PI3K, p-AKT, and p-mTOR increased signaling.

Major Concerns:

1. *Manuscript line 54-46: "Mechanistically, SIRPγ interacted with CD47 to activate the PI3K/AKT/mTOR pathway, leading to the upregulation of phosphoglycerate dehydrogenase (PHGDH) and the enhancement of serine metabolism". Since, the CD47 gene is expressed in T cells where its expression is crucial for their survival, function and for the maintenance of immune homeostasis, it is not clear whether the CD47-SIRPγ interaction takes place between the same cells (eg. CD8+ T cells) or across cell types (for example, between CD8+ T cells and Tregs). The authors should comment on how they envision this interaction to take place in the context of TPE and what would be the functional outcome of such interaction(s).*

Response: Thank you for your insightful comment. We fully agree that elucidating the mechanism of CD47–SIRPγ interaction, whether occurring within the same cell type or between different cell types, is an important consideration. In the revised manuscript, we have addressed this point in the revised Discussion section **(Page 21-22, line 603–614)**. Specifically, CD47, a member of the immunoglobulin superfamily (IgSF), is widely expressed across various cell types, including Treg and $CD8^+$ T cells, while SIRPγ is primarily expressed on T cells, including $CD8^+$ T cells. Given that the PI3K/AKT/mTOR pathway and downstream serine metabolism are induced upon CD47–SIRPγ engagement, we speculate that the relevant interaction likely occurs in trans, for instance between $CD8^+$ T cells and Tregs or between $CD8^+$ T cells and antigen

presenting cells, where SIRPγ on one cell engages CD47 on a neighboring cell. This trans interaction may facilitate metabolic reprogramming in $CD8^+$ T cells, contributing to the exhaustion phenotype observed in TPE. We also acknowledge that cis interactions on the same $CD8^+$ T cell cannot be formally excluded, and we have noted that distinguishing between these modes will require further investigation using cell type specific approaches.

2. Manuscript line 70: "However, TPE is difficult to diagnose". According to the current literature, adenosine deaminase (ADA), interferon-gamma (IFN-γ), and interleukin-27 (IL-27), all show high diagnostic accuracy for TPE while, ADA has been extensively studied as a biochemical marker in the TPE pleural fluid. Additionally, according to PMID: 20345583IF: 6.3 Q1 , this test is simple, cheap, rapid, minimally invasive, and can be performed in most laboratories. Thus, the introduction of SIRPγ as a diagnostic marker for TPE, especially when it is conferring only a slight increase in the diagnostic value in combination with ADA, is not immediately apparent. The authors should provide their suggestion on how they envision the usage of SIRPγ as a diagnostic tool in the current clinical practice.

Response: We thank the reviewer for the thoughtful comments. We fully acknowledge the established diagnostic performance of ADA, IFN-γ and IL-27 in TPE. Indeed, ADA in particular is a simple, cost-effective, and widely available test (Respirology. 2010 Apr;15(3):451-8). Nevertheless, our results show that SIRPγ provides a measurable improvement in diagnostic accuracy when used together with ADA. More importantly, SIRPγ reflects the extent of local $CD8^+$ T-cell exhaustion, which is not captured by existing biomarkers. The significance of SIRPγ therefore lies not only in its diagnostic contribution but also in its ability to reveal key immunological features of the TPE microenvironment and to identify a potential target for regulating T-cell dysfunction.

The significance of SIRPγ thus extends beyond its diagnostic contribution; it reveals key immunological features of the TPE microenvironment and identifies a potential target for regulating T-cell dysfunction. In clinical practice, we envision SIRPγ serving as a complementary marker in ADA-equivocal cases, where elevated

levels would support TPE diagnosis while also providing insight into local immune status **(Page 19, line 5301-536)**. In addition, we have revised the Introduction **(Page 3, line 75–81)** to highlight the need for biomarkers that possess both diagnostic and regulatory function of T cells. In the Conclusion **(Page 22, line 630–633)**, we further clarify the potential clinical application of SIRPγ. While providing diagnostic information, SIRPγ expression may also indicate the degree of local immune exhaustion. This concept offers a rationale for future therapeutic strategies aimed at restoring T-cell function through SIRPγ blockade.

3. Manuscript line 168: "The result revealed that SIRPγ was primarily expressed in T cells and NK cells (Fig. 2A–C, Fig. S2A–C)". The SIRPγ is expressed in both CD4+ and CD8+ cells in TPE. However, its expression is not different between PB derived and TPE derived CD4+ T cells. In contrast the SIRPγ expression is different between the PB derived and TPE derived CD8+ T cells. Given that, the role of CD8+ T cells in TPE is unknown, the physiological relevance, for TPE pathology is difficult to be extracted based on these data. The authors should provide the physiological context within which, the exhausted as well as, the other CD8+ T cell clusters (the memory clusters) operate within the TPE. Towards that end, they could also address the kinetics or pseudotime of SIRPγ expression among these clusters, in addition to that CD4+ T cells in TPE.

Response: Thank you for your important and insightful comment. We agree that interpreting the physiological relevance of SIRPγ expression requires placing $CD8^+$ T-cell subsets within the immunological landscape of TPE and examining their differentiation dynamics. To address this, we have expanded the biological context of $CD8^+$ T cells in TPE **(Page 4, line 92-94; Page 18-19, line 512-525)**. Specially, in TPE, the presence of T_N cells likely reflects recent recruitment from peripheral circulation and limited activation within the pleural microenvironment. Upon antigen stimulation, T_N rapidly differentiate into T_{EFF}, which contribute to anti-mycobacterial immunity through cytotoxic activity against infected macrophages and production of IFN-γ and TNF-α. Regardless of whether the antigen is completely cleared, a fraction of T_{EFF}

persists as self-renewing memory T cells, enabling rapid immune responses upon re-exposure. In the context of persistent infection, $CD8^+$ T cells progressively differentiate into T_{EX}, characterized by reduced proliferation, diminished cytokine secretion, and upregulation of inhibitory receptors. The decline in IFN-γ and TNF-α levels, together with impaired cytotoxic function, may compromise local anti-mycobacterial immunity and thereby contribute to disease persistence.

In addition, we incorporated pseudotime analyses to delineate the trajectory of SIRPγ expression across $CD8^+$ T-cell states **(Page 8, line 204-213, Fig. S4A-C)**. Unsupervised pseudotime analysis using Monocle3 revealed that T_N occupied the earliest position along the differentiation trajectory, whereas T_{EX} emerged at the terminal stage **(Fig. S4A, B)**. This continuum reflects the dynamic adaptation of $CD8^+$ T cells to the persistent inflammatory milieu characteristic of TPE. During this differentiation process, SIRPγ expression was predominantly observed in the T_{EX} subset rather than the memory T cell (T_M) and T_N population. Moreover, *SIRPG* expression increased markedly along the trajectory toward T_{EX} differentiation, accompanied by the upregulation of exhaustion markers such as *HAVCR2* and *CTLA4* **(Fig. S4C)**.

Figure S4 A-C

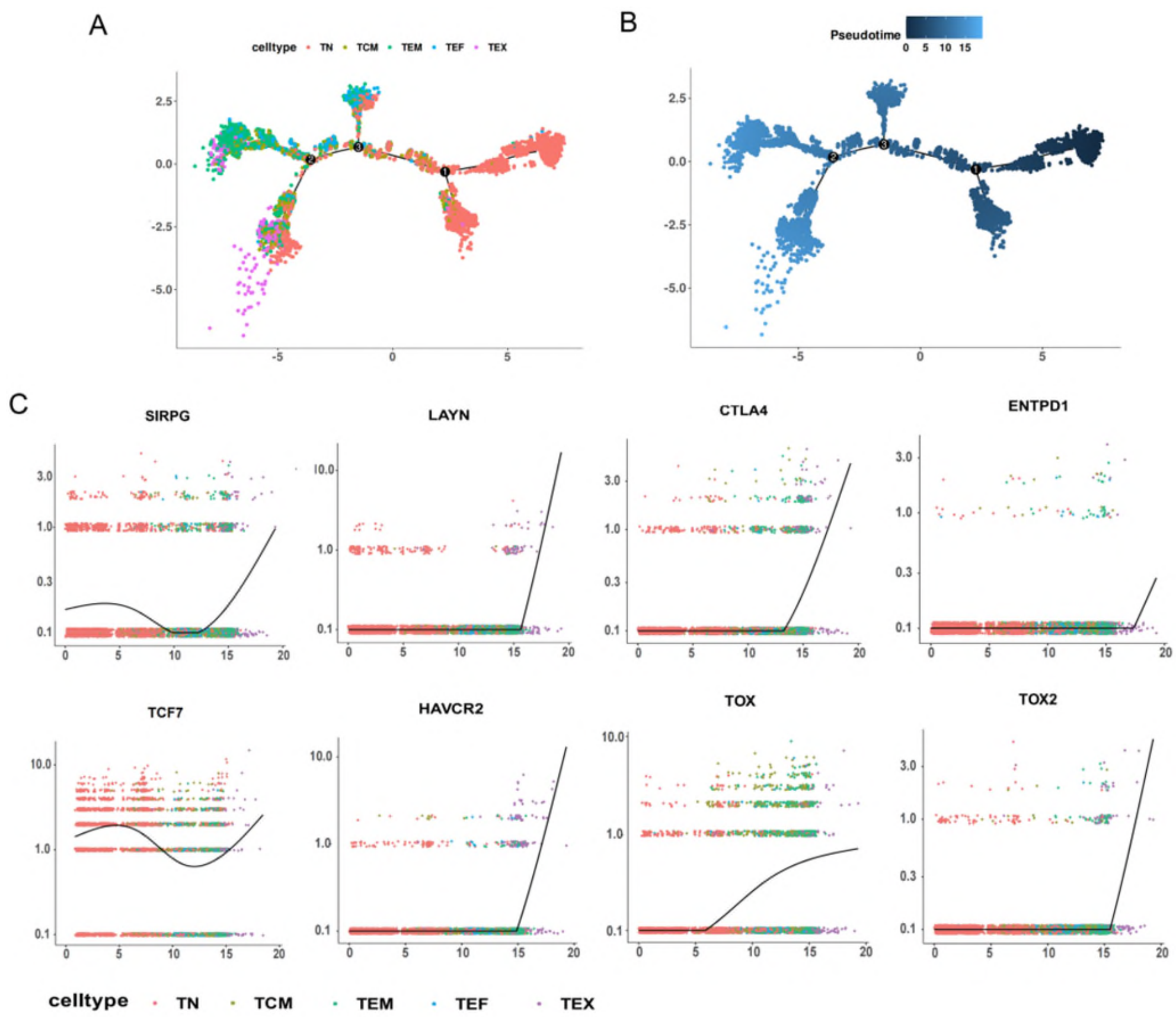

(A)The differentiation trajectory of CD8⁺ T cells clusters. (B) Monocle2 pseudotime analysis showing the transition tendency among different sub-clusters of $CD8^+$ T cells. (C) Expression dynamics of Marker gene signatures of exhausted $CD8^+$ T cells along the pseudotime axis.

4. An additional concern is that, the exhausted TPE CD8+ T cell cluster, is very small in comparison to the other clusters. It is unlikely that the diagnostic value of the SIRPγ in TPE resides entirely with this cell population. The physiological significance of the exhausted CD8+ T cell population with regards to the TPE pathology is also unclear. The authors should address these issues at least within the discussion of their results.

Response: We sincerely thank the reviewer for raising this important point. Although the exhausted $CD8^+$ T cell cluster constitutes a relatively small proportion of the total T cell population, the diagnostic value of SIRPγ in our study does not rely solely on this subset. Our diagnostic analysis was based on the detection of soluble SIRPγ in the pleural effusion supernatant. Further investigation revealed that SIRPγ is highly expressed on both $CD4^+$ and $CD8^+$ T cells, suggesting that its presence in pleural fluid may result from sustained proteolytic cleavage or release by these subsets under chronic

inflammatory stimulation. This provides a plausible explanation for its measurable diagnostic performance in pleural effusion (**Page 18, line 496-507**). Moreover, comparative analysis of immune cell subsets between TPE and MPE demonstrated higher SIRPγ expression on $CD4^+$ T, $CD8^+$ T, and NK cells in TPE, further supporting the notion that elevated SIRPγ levels in TPE may originate from multiple immune cell sources **(Fig. S3G, Page7, line 182-186)**.

Regarding the physiological significance, our study focused on the $CD8^+$ T cell compartment and identified a role for SIRPγ in promoting T cell exhaustion. Although this exhausted subset is numerically limited, it may hold functional significance in TPE pathology. The reduced expression of IFN-γ and TNF-α, along with impaired cytotoxic capacity within this population, may compromise local anti-mycobacterial immunity, thereby contributing to disease persistence. Thus, this subset offers crucial mechanistic insight into how SIRPγ shapes the immunosuppressive microenvironment in TPE (**Page 4, line 92-94; Page 18-19, line 512-525**).

5. The authors identify metabolic differences between SIRPγ+ and SIRPγ- CD8+ T cells by testing gene expression levels. However, the dynamics of the metabolic rewiring of a given cell type are unlikely to be regulated at the transcription level as they require a faster and dynamic type of regulation. A complementary approach would be the application of SCENITH assay into the TPE- derived CD8+ T cells.

Response: Thank you for your comment. We fully agree with the reviewer that metabolic regulation is a rapid and dynamic process, and that gene expression data alone are insufficient to fully capture the functional metabolic state of $SIRPγ^+$ versus $SIRPγ^-$ $CD8^+$ T cells.

The SCENITH assay indirectly infers energy metabolism by assessing terminal processes such as protein synthesis, reflecting overall cellular metabolic activity, including glycolysis and mitochondrial oxidative phosphorylation (Cell Metab. 2020 Dec 1;32(6):1063-1075.e7; Nat Commun. 2025 Jan 27;16(1):1083; Drug Resist Updat. 2025 Jul; 81:101237). However, this method does not provide information on the metabolism of specific amino acids such as serine, including the rate of serine uptake,

flux through one-carbon metabolism, or the efficiency of S-adenosylmethionine (SAM) synthesis from serine. In addition, the SCENITH assay kit is not reliably available in China. Given these limitations, we opted for a more direct and suitable approach, U-[^{13}C]-glucose isotope tracing, to assess glycolysis, serine synthesis, and one-carbon metabolism in SIRPγ^{+} and SIRPγ^{-} CD8^{+} T cells (2026 Feb 20;17(1):1944; Cell Metab. 2021 May 4;33(5):971-987.e6; J Clin Invest. 2023 Jul 3;133(13):e161713).

Following six hours of tracing, SIRPγ^{+} CD8^{+} T cells exhibited significantly enhanced glycolysis, serine synthesis, and one-carbon metabolism. These cells showed elevated levels of newly synthesized serine and SAM, whereas the steady-state glycine pool did not show a significant change. These findings indicate that, compared with SIRPγ^{-} CD8^{+} T cells, SIRPγ^{+} CD8^{+} T cells are more glycolytically dependent, display activated de novo serine synthesis, and exhibit increased one-carbon flux for SAM production. Notably, while we did not observe alterations in the glycine pool under these steady-state conditions (**Fig. 4M, Fig. S8 G–J, Page 11, line 297–307; Fig. S11 B, C, Page 13, line 362–367; Page 28, line 787-796**). These results are highly consistent with our previous gene expression data and further support our conclusions from the perspective of dynamic metabolic flux.

Figure 4N

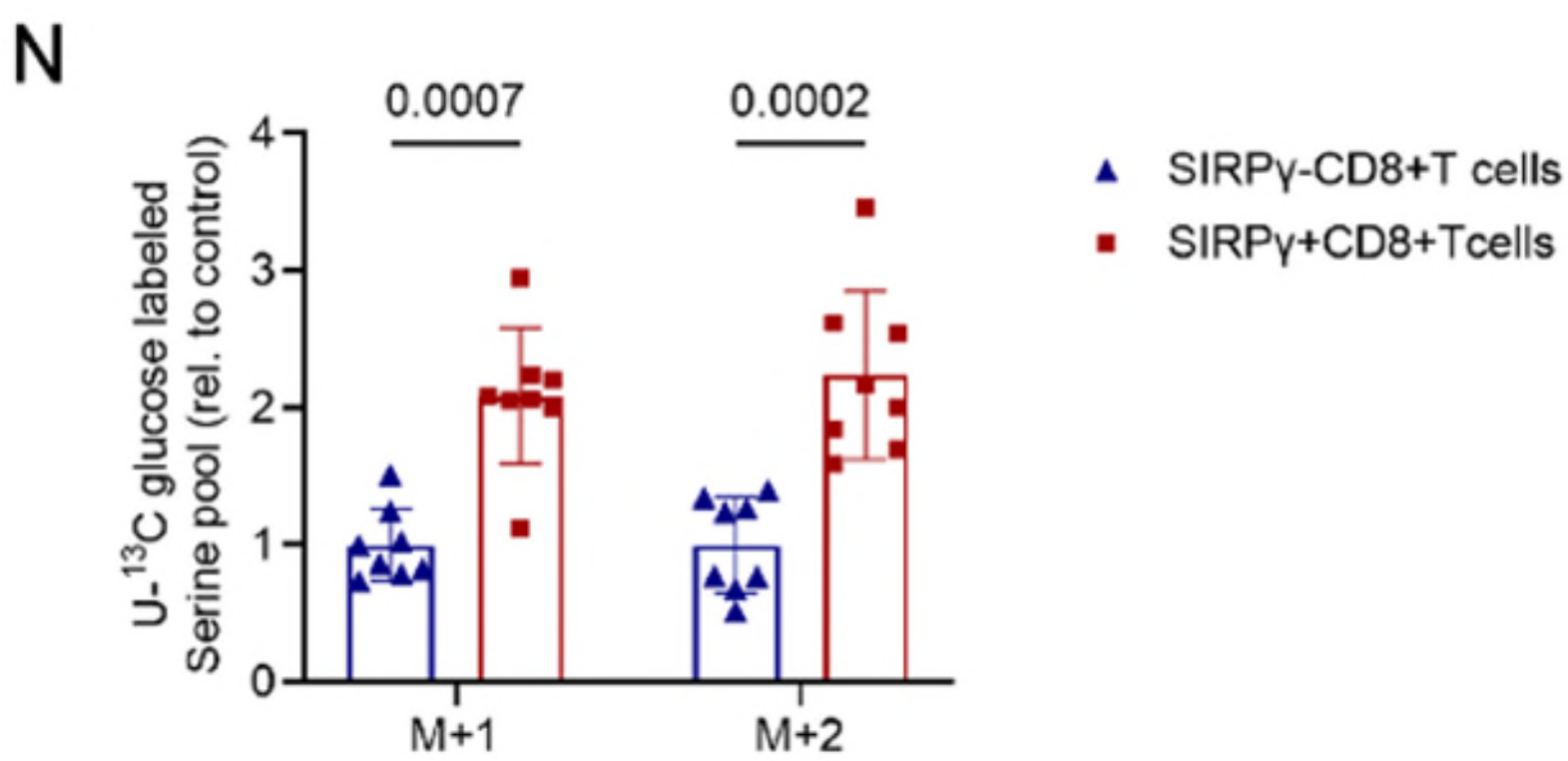

(N) Incorporation of U-[^{13}C]-glucose carbon into serine in sorted SIRPγ^{-} and SIRPγ^{+} CD8^{+} T cells derived from TPE (n=8).

Supplementary Figure 8G-J

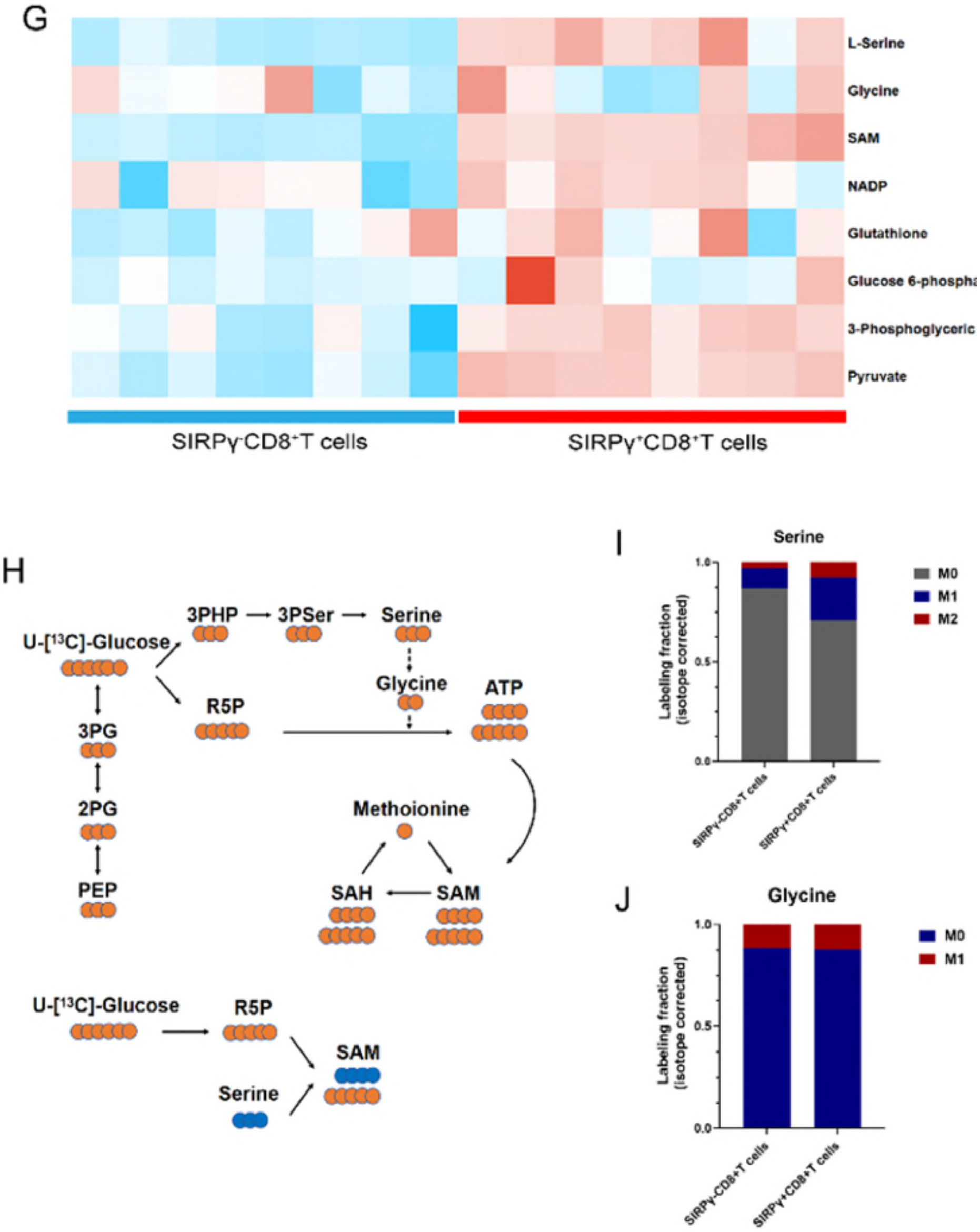

(G–J) SIRPγ⁻ and SIRPγ⁺ CD8⁺ T cells isolated from TPE were incubated with U-[^{13}C]-glucose for six hours. Heatmaps (G) and schematic representations (H) illustrate the incorporation of U-[^{13}C]-glucose into various metabolites. The percent distribution of each isotopic isomer in the serine (I) and glycine (J) pool is shown (n=8).

Supplementary Figure 11B, C

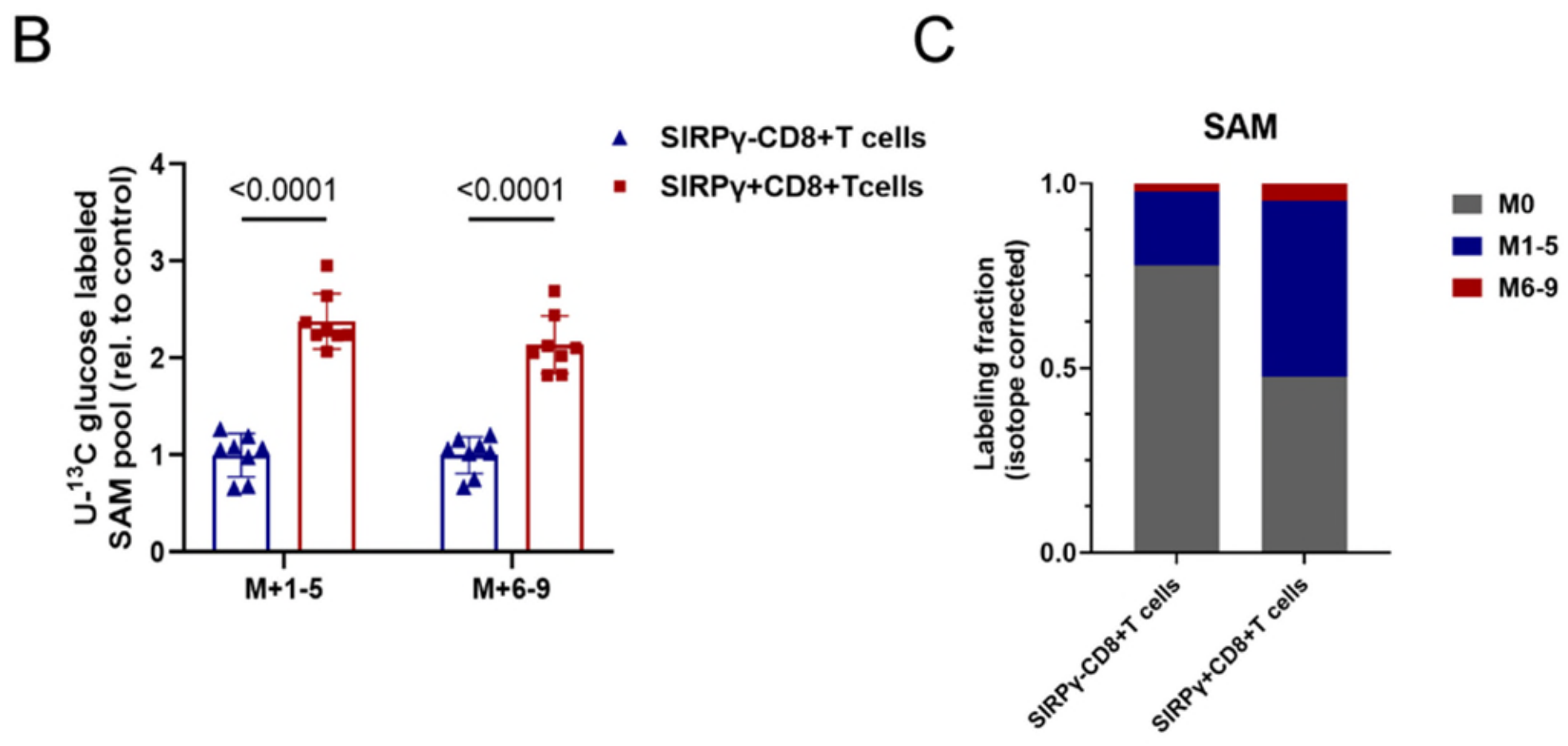

(B and C) Incorporation of U-[^{13}C]-glucose carbon into serine (B) and the percentage distribution of isotopologues in the SAM pool (C) in sorted SIRPγ^{-} and SIRPγ^{+} CD8^{+} T cells derived from TPE (n = 8).

6. *Manuscript line 299: "Notably, co-treatment with exogenous SG depletion with PHGDH-IN-3 synergistically attenuated SIRPγ+CD8^{+} T cell exhaustion, surpassing the effects of PHGDH inhibition alone". This statement means that serine deprivation alleviates T-cell exhaustion. However, later, in the manuscript line 320 the authors state that "To counteract the exhaustion phenotype induced by SG deprivation, cultures were supplemented with NADPH, GSH, or SAM". This sentence means that SG deprivation drives T-cell exhaustion. Furthermore, in manuscript lines 322-325 they state: "Notably, only SAM supplementation reversed SG deprivation–induced exhaustion, as evidenced by increased immunosuppressive receptors (CTLA-4, TIM-3, PD-1, TIGIT), reduced levels of cytokines (IL-2, TNF-α, IFN-γ, Granzyme B) and impaired proliferative capacity in SIRPγ^{+}CD8^{+} T cells (Fig. 6A, B, S9A)". Nevertheless, these characteristics represent the hallmarks of T-cell exhaustion and not the alleviation of exhaustion. The authors should thoroughly screen their text for such contradictions in their data interpretation.*

Response: We thank the reviewer for carefully identifying the inconsistencies in our interpretation of serine deprivation–related phenotypes. We apologize for the confusion caused by the inaccurate wording in the original manuscript. Our data consistently demonstrate that serine deprivation alleviates T-cell exhaustion, rather than inducing it.

We have thoroughly revised the relevant sections **(Page 13, line 357-358)**. Specially, to counteract the exhaustion phenotype reduced by SG deprivation, cultures were supplemented with NADPH, GSH, or SAM. We have carefully reviewed the entire manuscript to ensure that all descriptions of serine deprivation and T-cell exhaustion are now logically consistent and accurately aligned with our findings **(Page 13, line 369-371)**.

7. Manuscript line 351: "To explore the epigenetic regulatory role of SAM, genome-wide chromatin accessibility in SAM-treated CD8$^+$ T cells was initially assessed using ATAC-seq". The authors use for this work CD8$^+$ T cells coming from the following sources: a) Peripheral Blood Mononuclear Cells (PBMCs) collected from three groups: 1) PPD-positive healthy volunteers, 2) Patients with tuberculous pleural effusion (TPE), 3) Patients with active pulmonary tuberculosis (TB) b) Pleural Effusion-Derived Mononuclear Cells (PEMCs): 1) PEMCs were obtained from pleural effusion samples of TPE patients. These cells were used for various experimental assays, including flow cytometry, RNA sequencing, ELISA, proliferation assays, ATAC-SEQ and other functional and molecular analyses. It is mandatory that the authors should carefully state the source of these CD8$^+$ T cells used in their particular assays in detail as well as, clearly state the rationale behind their choice of one cell source over another.

Response: Thank you for your suggestions. We have clarified that genome-wide chromatin accessibility was initially assessed by ATAC-seq in SAM-treated CD8$^+$ T cells derived from TPE. We selected this cell source because TPE derived CD8$^+$ T cells are antigen experienced and exhibit pronounced exhaustion associated phenotypes, making them a physiologically relevant model for investigating the epigenetic regulatory effects of SAM on dysfunctional T cells in the context of tuberculosis infection **(Page 14, line 390-392; Page 53, line 1356-1358)**. In addition, we have now carefully revised the manuscript to clearly specify the source of CD8$^+$ T cells used in each experiment in the Method/Result section and in all relevant figure legends **(Page 8, line 219-222; Page 9, line 244; Page 11, line 312-313; Page 12, line 333-335; Page 13, line 367; Page 24, line 678-679; Page 25, line 717-719; Page 53, line 1362-1363,**

Page 54, line 1372; Page 55, line 1381-1384).

8. Manuscript lines 403-408: "Consistent with these observations, SAM-treated CD8⁺ T cells displayed elevated NFATC2 expression, accompanied by transcriptional upregulation of inhibitory receptors and exhaustion-associated transcription factors, including TOX, BATF, and IRF4. We therefore propose that SAM promotes CD8+T cell exhaustion by enhancing H3K4me3 modification at the NFATC2 promoter, facilitating expression of immunosuppressive receptors and exhaustion-related transcription factors (TOX and IRF4)". As NFAT transcription factors are subject to calcium-dependent activation, it is unlikely that the transcriptional de-repression of this genetic locus, only driven by a transcriptionaly favorable epigenetic modification could result in a significant change in the transcriptomic profile of the cell. Despite the authors considerable efforts to back these data up with in vitro assays, the mechanistic evidence for SAM driving CD8+T cell exhaustion is rather circumstantial. The authors should comment on a possible over interpretation of their data.

Response: We sincerely thank the reviewer for the insightful comments. We fully concur that the full activation of NFAT transcription factors is typically calcium-dependent. We acknowledge that our initial findings only suggested a potential auxiliary or synergistic role for SAM and H3K4me3 modification in this process. To establish a direct causal link between NFATC2 and the observed biological phenotypes, further validation experiment, such as those using the CsA inhibitor, calcium flux assays, and NFATC2 nuclear translocation analysis, are necessary.

To address this, we first re-evaluated whether SAM drives exhaustion through NFATC2 by treating SAM-cultured $CD8^+$ T cells with the NFAT-specific inhibitor cyclosporine A (CsA). CsA treatment abrogated the SAM-induced upregulation of inhibitory receptors (PD-1, CTLA-4, TIGIT) and restored effector cytokine production (IFN-γ, TNF-α, Granzyme B) (**Fig. S13A–D**). These results indicate that NFATC2 is a pivotal mediator of the SAM-driven exhaustion phenotype. Our previous sequencing analysis revealed that SAM-treated $CD8^+$ T cells exhibited enhanced TCR signaling (**Fig. S11D**), suggesting that SAM-induced epigenetic changes at the NFATC2 locus

may synergize with downstream TCR signals (such as calcium flux) to promote exhaustion. Mechanistically, we further observed that SAM treatment directly enhanced calcium influx and promoted NFATC2 nuclear translocation, even under weak calcium stimulation (**Fig. S13E, F**). Accordingly, we propose that SAM may lower the activation threshold of NFATC2 by enhancing H3K4me3 modification at its locus. As a methyl donor, SAM metabolism may also intersect with other signaling pathways to facilitate calcium influx, thereby synergistically promoting full NFATC2 activation.

We acknowledge that our initial conclusion may have overinterpreted the role of H3K4me3 modification. Accordingly, the relevant sections of the manuscript have been revised to present a more tempered interpretation: SAM may contribute to, rather than drive, exhaustion by establishing a permissive epigenetic environment at the NFATC2 locus via H3K4me3 enrichment. These updates have been incorporated into the Results section (**Page 14, line 377-378; Page 16-17, line 448-463**) and further emphasized in the Discussion (**Page 21, line 579–591**), where we reinforce the concept that epigenetic remodeling creates a permissive state for exhaustion.

Supplementary Figure 13

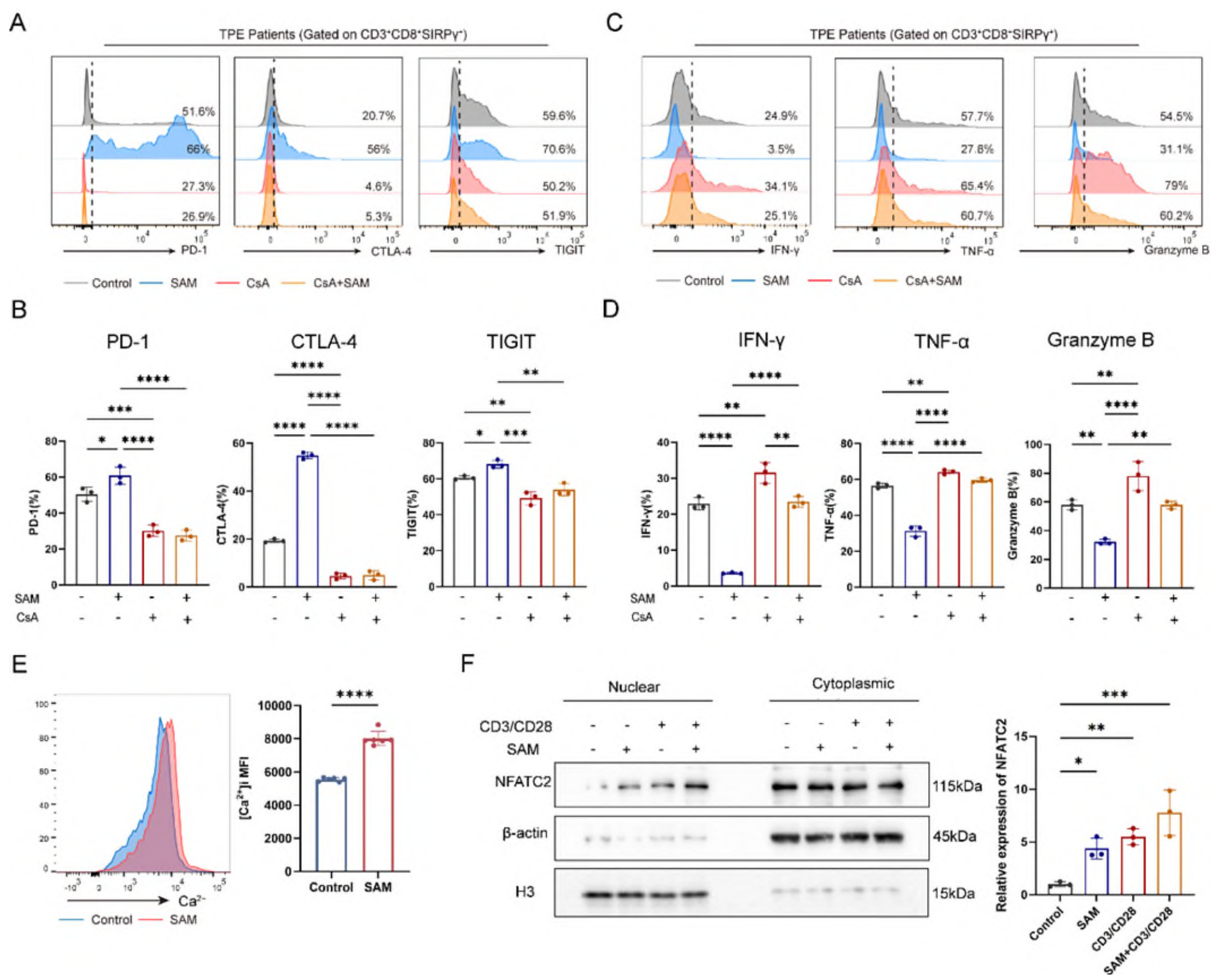

Supplementary Figure 13 Serine-derived SAM promotes an active epigenetic state at the NFATC2 locus to drive T cell exhaustion, related to Figure 7. (A-D) CD8$^+$ T cells isolated from TPE were activated ex vivo and treated with or without 400 μM SAM ± cyclosporin A (CsA) (1 μM). The expression levels of inhibitory receptors (TIGIT, CTLA-4, PD-1) (A, B) and cytokines (Granzyme B, IFN-γ, TNF-α) (C, D) were measured by flow cytometry (n=3). (E) CD8$^+$ T cells from TPE were incubated with SAM (400 μM) for 20 h, after which intracellular Ca^{2+} levels ([Ca^{2+}]i) were assessed via flow cytometry (n=6). (F) Representative western blots of nuclear and cytoplasmic extracts from CD8$^+$ T cells isolated from TPE, either untreated or treated with CD3/CD28 antibodies (1 μg/mL) ± SAM (400 μM), showing NFATC2 protein expression. Statistical analysis was determined by unpaired two-tailed t-test (E) and one-way ANOVA (B, D, F).

Minor Concerns:

1. *Manuscript line 159: "Collectively, support…". The authors should rephrase as the current statement does not make sense.*

Response: We thank the reviewer for pointing out this oversight. The sentence was indeed incomplete. We have rephrased it on line 159 **(now Page 6, line 171-172)**, as suggested, to provide a clear subject and a complete statement. The revised sentence now reads: “Collectively, these findings support the potential of SIRPγ as a diagnostic biomarker for TPE.”

2. *Manuscript line 182: “Clusters 0, 2, 4, 5, 6, 11, and 12 represented naïve-like T cells (TN), characterized by CCR7, SELL, TCF7, LEF1, and MALL”. How naïve-like T cells are found amidst a fulminating inflammatory reaction? Are all these cells CD45RA+?*

Response: We sincerely thank the reviewer for raising this important scientific question. This issue is indeed critical for ensuring the rigor of our phenotypic definitions and for strengthening the interpretability of our findings. Our detailed responses are provided below.

1) Rationale for the presence of naïve-like T cells (T_N) within a highly inflammatory microenvironment:

The identification of the T_N subset in our study is based on the high expression of canonical naïve T (T_N) cell markers, including CCR7, SELL (CD62L), TCF7, LEF1, and MALL, at the single-cell transcriptomic level. These markers represent well-established molecular hallmarks of T_N cells and align with the classical transcriptional criteria widely adopted in single-cell immunology. The presence of T_N cells in an explosive inflammatory microenvironment is biologically plausible. In both tumor and inflammatory settings, peripheral T_N cells are continuously recruited into the tissue, and a proportion of resting T_N cells can reside locally. Moreover, T_N cells constitute an essential reservoir for initiating adaptive immune responses within such environments. This phenomenon has been repeatedly demonstrated in multiple single-cell T-cell studies and is highly consistent with the immune-regulatory features observed in our dataset (Journal of Experimental Medicine. 2022;219(6):e20210749; Nature. 2023;624(7990):154-163; Science Immunology. 2023;8(89):eadh3113; Nature Communications. 2021;12(1):5303).

2) Clarification regarding the CD45RA phenotype of T_N cells:

CD45RA is the classical splice isoform of the PTPRC gene, which encodes CD45, and it serves as a key surface marker of T_N cells at the protein level. The well-established phenotype of T_N cells is $CCR7^+CD62L^+CD45RA^+$, and our study fully acknowledges and follows this biological definition. However, only the total transcript abundance of PTPRC can be detected in our single-cell RNA-sequencing dataset. The technology does not allow discrimination of the RA-specific splice variant, which reflects a known technical limitation of scRNA-seq for isoform-level resolution. To present the expression pattern of PTPRC more clearly, we have added this gene to the marker-gene bubble plot **(Fig. S2L)**. The plot displays both the average expression level and the proportion of expressing cells across all subsets, providing additional support for the transcriptional characteristics of the T_N cluster.

3. *Manuscript line 193: "To address this, freshly isolated PBMCs from PPD^+ donors were stimulated with the recombinant fusion antigen ESAT-6–CFP-10 (E195 C)". Please explain the "PPD+" acronym as well as, where the ESAT-6–CFP-10 is derived from and the rationale behind it's choice.*

Response: Thank you. We have added relevant explanations to clarify the term "PPD^+ donors". This term refers to purified protein derivative-positive (PPD^+) donors, i.e., individuals with a positive tuberculin skin test using purified protein derivative (PPD) of M. tuberculosis (**Page 8, line 219–222; Page 23, line 657–660)**. In addition, we have stated that the recombinant fusion antigen ESAT-6–CFP-10, derived from the RD1 region of the M. tuberculosis genome (which is specific for M. tuberculosis infection and absent in BCG), was used to stimulate antigen-specific T cell responses (**Page 24, line 678–685**).

4. *manuscript line 204: "...implicating a potential role for SIRPγ in promoting $CD8^+$ T-cell exhaustion via tuberculosis antigens". I believe it is the other way around: "Implying a potential role for tuberculosis antigens in promoting $CD8^+$ T-cell exhaustion via SIRPγ".*

Response: We thank the reviewer for this insightful comment. Upon careful

consideration, we agree that the original phrasing may have led to ambiguity regarding the directionality of the proposed mechanism. Our data suggest that tuberculosis antigens may indeed induce $CD8^+$ T-cell exhaustion through the upregulation or modulation of SIRPγ expression. Therefore, we have revised the sentence in line 204 (**now Page 9, line 232-233**) to read: "...implying a potential role for tuberculosis antigens in promoting $CD8^+$ T-cell exhaustion via SIRPγ."

5. *Manuscript line 209: "…scRNA-seq were categorized into three functional group", should read "…scRNA-seq were categorized into three functional groups". The authors should thoroughly scrutinize and edit their text for typos and poorly formed sentences.*

Response: We sincerely thank the reviewer for pointing out this grammatical error. We have corrected the phrase in **line 238** to now read: "···scRNA-seq were categorized into three functional groups." In addition, we have carefully reviewed the entire manuscript and performed a thorough language check to correct any remaining typographical errors and improve sentence clarity and grammatical accuracy.

6. *Suppl Fig 1A: the analysis is very poor and the lettering too small to read particularly the gene list.*

Response: We sincerely thank the reviewer for their valuable feedback on Supplementary Figure 1A. We have thoroughly revised the figure to enhance both its analytical clarity and visual accessibility. Specifically, in the updated version, we have substantially increased the font size of all textual elements, including axis labels, the legend, and, most critically, the gene list. Furthermore, we have optimized the panel layout to accommodate the larger text while preserving a balanced and professional visual structure. We confirm that all text remains clearly legible at standard publication resolution.

7. *Suppl Fig 1B: CD172G is not among the highest overexpressing genes in this screening but it is the only one marked. The authors should provide information about the other genes on this volcano plot.*

Response: Thank you for your suggestion. We have re-analyzed the sequencing results and updated the legend of Supplementary Figure 1B to clarify the selection criteria, and we have highlighted additional significant genes to provide a broader context for the screening.

8. *Figure 1B: the analysis is very poor and the lettering too small*

Response: Thank you for the valuable comments on Figure 1B. We have substantially increased the font size of all textual elements in the revised figure, including axis labels, the legend, and the gene list. We have carefully verified that all text is clearly legible at the intended publication scale.

9. *Figure 1E: is mislabeled as 1D*

Response: We sincerely thank the reviewer for pointing out this labeling error. Upon careful review, we have corrected this mistake in the revised manuscript to ensure consistency between the figure and its description.

10. *Figure 2L: the analysis is very poor and the lettering too small*

Response: We sincerely appreciate the reviewer's valuable suggestions regarding Figure 2L. We have re-analyzed the sequencing results and updated the legend of Figure 2L (now **Fig. S3K)** to enhance both its analytical clarity and visual accessibility.

11. *Figure 3B: the analysis is very poor and the lettering too small*

Response: We sincerely appreciate the reviewer's valuable suggestions. In response, we have revised Figure 3B as follows: We implemented systematic improvements to the figure's visual presentation. Specifically, we introduced color-coded annotations for functional gene modules to emphasize the analytical rationale through functional differentiation. Additionally, we increased the font size of gene names, group labels, and annotations, and adjusted the spacing between the heatmap and textual elements to address issues of small font size and visual ambiguity. Finally, we enhanced the color contrast of the heatmap blocks to make differences in gene expression levels more

visually discernible and intuitive.

Reviewer #2 (Remarks to the Author):

This is an interesting manuscript that provides evidence that signal regulatory protein γ (SIRPγ) can be used as a novel biomarker to distinguish tuberculous pleural effusion (TPE) from other types of pleural effusions. The authors also demonstrate that SIRPγ is involved in TPE pathogenesis through induction of $CD8^+$ T cell exhaustion. They demonstrate that SIRPγ interacts with CD47 to activate the PI3K/AKT pathway, leading to the upregulation of phosphoglycerate dehydrogenase (PHGDH) and enhancement of serine metabolism, which in turn facilitates SAM-dependent trimethylation of histone H3 lysine 4 (H3K4me3) epigenetic modification, ultimately leading to the expression of exhaustion-associated transcription factors and immunosuppressive receptors. The data are generally of value and may have some clinical translational implications. In general the methodological approaches are appropriate and the studies well done. However, there are some issues that require attention, as outlined below.

1.*Figures 2 I and J. These are very important data as demonstrate the differential expression of SIRPγ on the CD8 cells in TPE versus blood. As SIRPγ is also expressed on CD4 cells (Fig. 2D), have the authors examined the same paired clinical samples for SIRPγ expression on CD4 cells as well?*

Response: We sincerely thank the reviewer for this thoughtful and important comment. We fully agree that evaluating SIRPγ expression on $CD4^+$ T cells in paired TPE and peripheral blood samples is essential for a comprehensive understanding of its role in T-cell biology during tuberculosis. In fact, we did perform this analysis using the same paired clinical samples, and the results are presented in **Figure S3H-J**. Our data showed that SIRPγ expression on $CD4^+$ T cells did not differ significantly between TPE and peripheral blood, in contrast to the marked upregulation observed on $CD8^+$ T cells. This lack of differential expression in $CD4^+$ T cells led us to focus our subsequent functional

and phenotypic analyses on $CD8^+$ T cells, where SIRPγ appeared to play a more prominent role in the context of TPE (**Page 7, line 188-189**).

To improve clarity, we have now revised the Discussion section to explicitly state that SIRPγ expression on $CD4^+$ T cells was evaluated in the same paired samples, and to elaborate on the rationale for focusing on $CD8^+$ T cells (**Page 19, line 529-531**). Specifically, we discussed how the absence of significant changes in $CD4^+$ T cells suggested a more selective involvement of SIRPγ in modulating $CD8^+$ T-cell responses within the pleural environment.

Supplementary Figure 3G-I

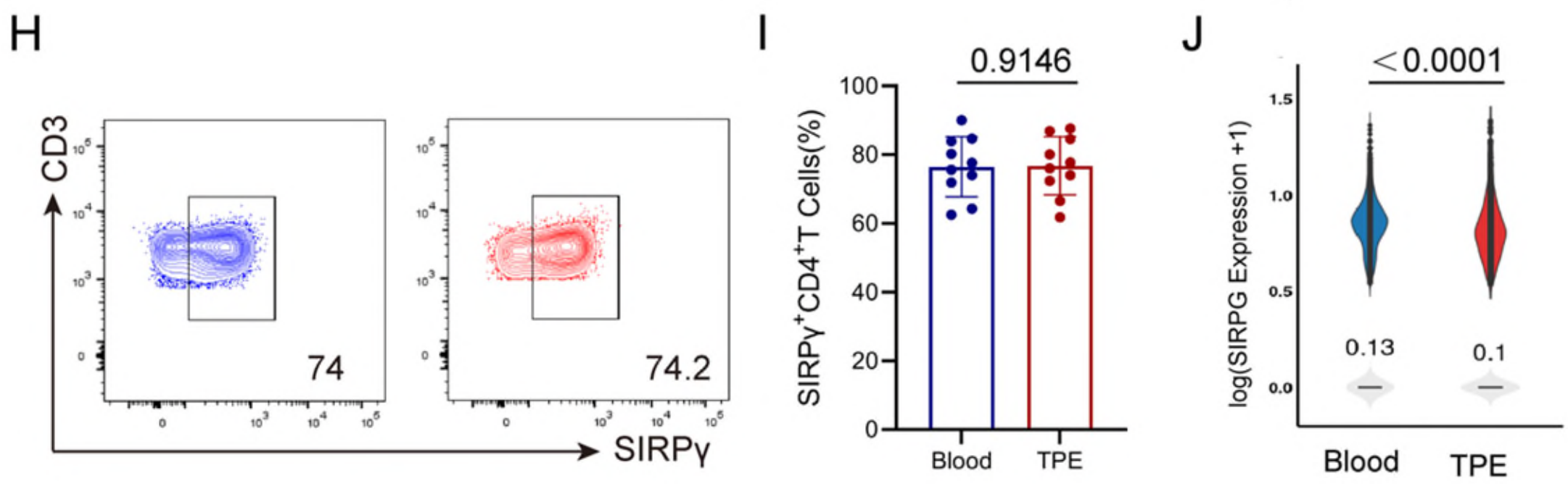

(H, I) Flow cytometric quantification of SIRPγ$^+$CD4$^+$ T cells (CD3$^+$CD4$^+$SIRPγ$^+$) frequencies in TPE versus matched peripheral blood: Representative plots (H) and statistical analysis (I) (n=10). (J) Single-cell RNA sequencing analysis comparing SIRPG expression in $CD4^+$ T cells from pleural effusion versus peripheral blood.

2.*The data in Figures 3 and S3/S4 are convincing and establish a correlation between SIRPγ expression on CD8 cells and inhibitory receptors expression and increased expression of exhaustion markers; as well as reduced cytokine production in paired samples from TPE patients. The authors have also excluded a series of other clinical correlations, as summarized in Table 1. One set of parameters that is not indicated in Table 1 is whether some of the patients were on active anti-TB treatment at the time of the studies and whether there was any correlation between anti-TB drugs and T cell exhaustion markers. If these clinical data are available, some additional analysis may help to further solidify the conclusions.*

Response: Thank you for your insightful comments. Your concern regarding the potential impact of anti-tuberculosis treatment on T-cell exhaustion phenotypes is highly relevant and was, in fact, a key consideration during the initial design of our study.

We would like to clarify that, in order to eliminate potential confounding effects of therapeutic agents, including anti-tuberculosis drugs and corticosteroids, on the pleural immune microenvironment, only newly diagnosed TPE patients who had not yet received any anti-TB or immunomodulatory treatment at the time of pleural fluid collection were enrolled in this study. We have now clarified this inclusion criterion in the revised manuscript (**Methods section, page 23, line 648-650**): "To avoid potential confounding effects of anti-tuberculosis therapy on immune parameters, only treatment-naïve TPE patients were enrolled in this study." We regret that, due to this design, we are unable to provide an analysis of the correlation between anti-TB treatment and T cell exhaustion markers in the current dataset. We will consider incorporating such comparisons in future studies.

3.*In the legend for Figure 4, it is stated "Statistical significance was determined by unpaired two-tailed Student's t-test (C, D, F, H, K) and paired two-tailed t-tests (K)". So, for panel K, which t-test was used unpaired or paired t-test? Also, based on what criteria was used unpaired versus paired in the different panels?*

Response: We sincerely thank the reviewer for carefully pointing out the inconsistency in the legend of Figure 4. We apologize for the confusion caused by the incorrect annotation. To clarify, panel K was analyzed using a paired two-tailed Student's t-test, as the data represent matched samples from the same individuals under different conditions. The inclusion of panel K under both unpaired and paired tests in the original figure legend was an oversight, and we have now corrected this in the revised manuscript. We have revised the legend of Figure 4 to read (**Page 48, line 1290-1291**): "Statistical significance was determined by unpaired two-tailed Student's t-test (D, F, G, L) and paired two-tailed t-tests (K, N)".

Regarding the criteria for selecting the statistical tests:

Paired t-tests were used when comparing measurements from the same individuals under two different conditions (e.g., before and after treatment, or paired compartments such as blood vs. pleural fluid). Unpaired t-tests were applied when comparing two independent groups, such as MPE versus TPE patients, or when comparing treated versus untreated groups where the sample sizes (n values) were derived from independent experiments without one-to-one correspondence.

Reviewer #3 (Remarks to the Author):

This study investigated the role of signal regulatory protein gamma (SIRPy) in tuberculous pleural effusion (TPE). The authors first identified SIRPy as a novel diagnostic biomarker through high-throughput antibody microarray screening, demonstrating its elevated expression in TPE compared to malignant pleural effusion (MPE). The authors propose that SIRPy interacts with CD47 to activate the PI3K/AKT/mTOR pathway, upregulates phosphoglycerate dehydrogenase (PHGDH), enhances serine metabolism, and promotes S-adenosylmethionine (SAM)-dependent H3K4me3 modifications. This cascade ultimately induces NFATC2 expression, driving exhaustion-associated transcription factorsand immunosuppressive receptors. While the study presents interesting findings, several methodological and interpretive issues require clarification and additional experimental evidence.

1.*The protein marker screening and evaluation of SIRPy's diagnostic performance were conducted solely between TPE and MPE, while parapneumonic pleural effusion (PPE)—one of the three most common causes of exudative pleural effusions—was not included. It is essential to incorporate PPE samples to clarify whether elevated SIRPy expression is specific to TPE.*

Response: We sincerely thank the reviewer for this insightful comment and for emphasizing the importance of including PPE samples to assess the specificity of SIRPγ expression in TPE. We would like to clarify that PPE samples were not included in the discovery cohort, which focused on identifying candidate markers by comparing TPE

and MPE patients. However, in the validation cohort, we did include PPE and miscellaneous samples and performed a comparative analysis of SIRPγ expression among TPE, MPE, miscellaneous, and PPE groups **(supplementary Table 3, page 23, line 653-656)**. This allowed us to evaluate the specificity of SIRPγ elevation in TPE. We acknowledge that this was not clearly described in the original manuscript. To address this, we have made the following revisions:

Figure 1I legend (page 42, line 1213–1214): (I) Comparative analysis of SIRPγ levels in TPE versus non-TPE including MPE, parapneumonic pleural effusion (PPE), and miscellaneous from validation cohort.

Results and Discussion section (page 6, line 151–154; Page 18, line 499-502): We added, "To further evaluate the diagnostic specificity of SIRPγ, we expanded the validation cohort to include parapneumonic pleural effusion (PPE) and miscellaneous samples. The analysis revealed that SIRPγ, ADA, and SIRPγ*ADA consistently maintained strong diagnostic performance (Table S2)."

2. *The authors showed that Fig. 1N displays H&E and SIRPy IHC staining of TPE lesion tissue and adjacent non-tuberculous tissue. However, the provided images lack sufficient resolution to discern cellular and tissue architecture clearly. From what is visible, the TPE lesion area does not show typical granulomatous structures, raising questions about whether it represents a classic tuberculous lesion. The adjacent non-lesion area shows extensive fibrous exudate with only scattered cells, suggesting low cellularity. How was SIRPy expression quantified in such a paucicellular region? The Methods section does not specify the number of cases included in the histological analysis or the detailed protocol for SIRPy IHC evaluation. Furthermore, based on the H&E and IHC results, in which specific immune cell types within the tuberculous lesion or its periphery, was high SIRPy expression observed?*

Response: We thank the reviewer for the valuable comments regarding the histological analysis presented in Figure 1N. We have thoroughly re-evaluated our tissue staining and quantification strategies, and have revised the manuscript as follows:

1) Optimization of histological images and characterization of tuberculous lesions: We

have replaced the original Figure 1N with newly scanned, high-magnification images (now presented as **Fig. S2A, Page 6, line 162-166**). Upon re-examination of additional tissue sections, we confirmed the presence of typical granulomatous structures within the pleural lesion areas. To avoid regions with extensive fibrous exudate, a new adjacent non-tuberculous tissue area was selected, which now exhibits relatively normal pleural morphological features.

2) Identification of SIRPγ-expressing cell types: Immunohistochemical and immunofluorescence analyses revealed that SIRPγ is predominantly expressed in specific immune cells located at the periphery of the lesions and co-localizes with $CD3^+$ T lymphocytes (**Fig. S2B, C; Page 6, line 166-168**).

3) Re-evaluation of SIRPγ IHC quantification: We acknowledge that both the adjacent non-lesional areas and the lesions themselves exhibited relatively sparse cellularity, and SIRPγ expression is confined to $CD3^+$ T cells, which are not abundant in the tissue. Consequently, our initial quantification method, which was based on total positive area, was not appropriate for this context. We have therefore re-quantified the expression levels by calculating the density of $SIRP\gamma^+$ cells and have revised the detailed protocol for SIRPγ IHC evaluation accordingly (**Page 26-27, line 750-770**) as follows:

Pleural tissue specimens, including tuberculous lesions and adjacent normal pleural tissues, were collected from 14 patients with TPE. Tissues were fixed overnight in 4% paraformaldehyde, embedded in paraffin, and sectioned into 4-μm-thick slices. For histological analysis, sections were stained with hematoxylin and eosin (H&E) according to standard protocols. Immunohistochemical (IHC) staining was performed to detect SIRPγ expression. Briefly, paraffin-embedded sections were deparaffinized, rehydrated, and subjected to heat-induced antigen retrieval. Endogenous peroxidase activity was blocked with 3% H_2O_2, and non-specific binding was blocked with 1% bovine serum albumin (BSA) for 30 min at RT. Sections were then incubated overnight at 4°C with primary antibodies against SIRPγ. After washing with PBS, sections were incubated with HRP-conjugated secondary antibodies for 1 h at RT, followed by streptavidin-peroxidase solution for 15 min. The immunoreaction was visualized using 3,3'-diaminobenzidine (DAB) substrate, and sections were counterstained with

hematoxylin to visualize nuclei.

Quantitative analysis of SIRPγ-positive cells was performed on stained sections. Stained sections were evaluated independently by two investigators blinded to the clinical data. For each sample, 10 random high-power fields (400× magnification; each field area = 0.237 mm²) were selected per section, and the number of SIRPγ$^+$ cells was counted. The mean cell count per mm² was calculated for each sample. Inter-observer variability was assessed, and discrepancies were resolved by consensus to ensure data reliability.

Supplementary Figure 2

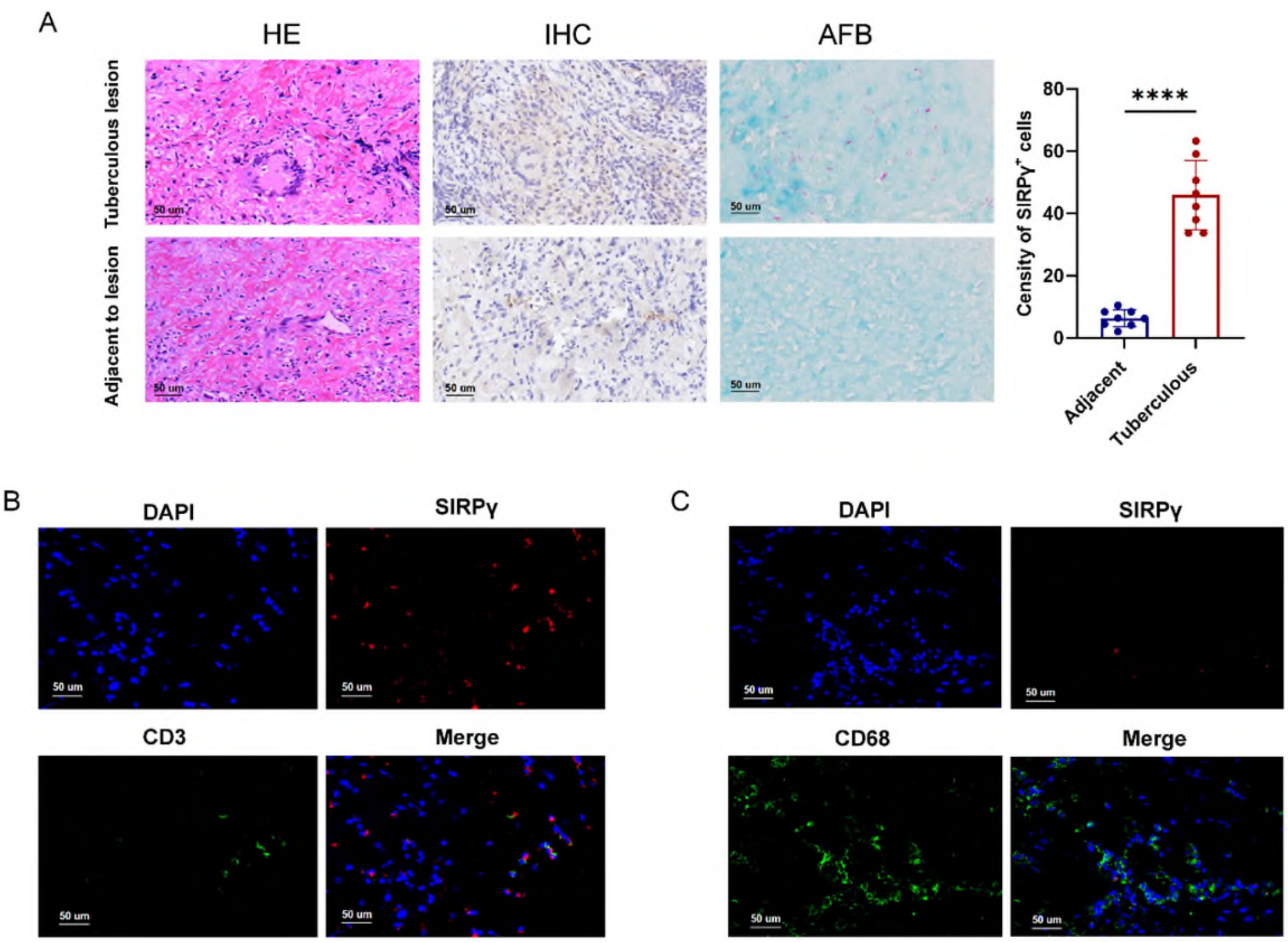

Supplementary Figure 2 SIRPγ serves as diagnostic biomarker for TPE, related to Figure 1. (A) Representative hematoxylin and eosin (H&E), immunohistochemistry (IHC) detection of SIRPγ, and acid-fast bacteria stain (AFB) stains in pleural tissues are displayed with corresponding quantitative results of SIRPγ$^+$ cell density (n = 8). (B) Representative images demonstrating the colocalization of SIRPγ and CD3$^+$Tcells from tuberculous lesions. Red: SIRPγ; green: CD3; blue: DAPI for nucleus. Scale bar 50 μm. (C) Representative images demonstrating the colocalization of

SIRPγ and macrophages from tuberculous lesions. Red: SIRPγ; green: CD68; blue: DAPI for nucleus. Scale bar 50 μm. Statistical analyses were performed using paired two-tailed t-tests (A).

3. *In lines 156–160, the authors suggest that high SIRPy expression in tissue specimens indicates a greater likelihood of microbiological confirmation. What evidence supports this conclusion? It remains unclear whether such a correlation genuinely exists, particularly as the histopathological results in Fig. 1N are not definitive.*

Response: We fully agree with the reviewer that our initial statement that "high SIRPγ expression indicates a greater likelihood of microbiological confirmation" was an overinterpretation based on the available histopathological evidence. To directly address this concern, we performed a rigorous re-evaluation of acid-fast bacillus (AFB) staining specifically within the lesional areas of all clinically diagnosed TPE samples included in the study.

As detailed in our response to Comment #2, we confirmed that SIRPγ is highly expressed on $CD3^+$ T cells within granulomatous lesions. In cases where AFB-positive bacilli were identified, the density of $SIRPγ^+$ cells was significantly higher than in AFB-negative cases (**Fig. 1N**). This spatial overlap suggests a potential accumulation of $SIRPγ^+$ T cells at sites of active mycobacterial replication, providing preliminary evidence of an association between SIRPγ expression and microbiological confirmation. To retain this interesting observation while maintaining scientific rigor, we have revised the description of these results to reflect a more cautious interpretation. The revised text now reads as follows: Notably, among pleural specimens from patients clinically diagnosed with TPE, those positive for acid-fast bacilli staining exhibited a higher density of $SIRPγ^+$ cells, suggesting a potential correlation between SIRPγ expression and microbiological confirmation (**Fig. 1N, page 6, line 168-172**).

Figure 1N

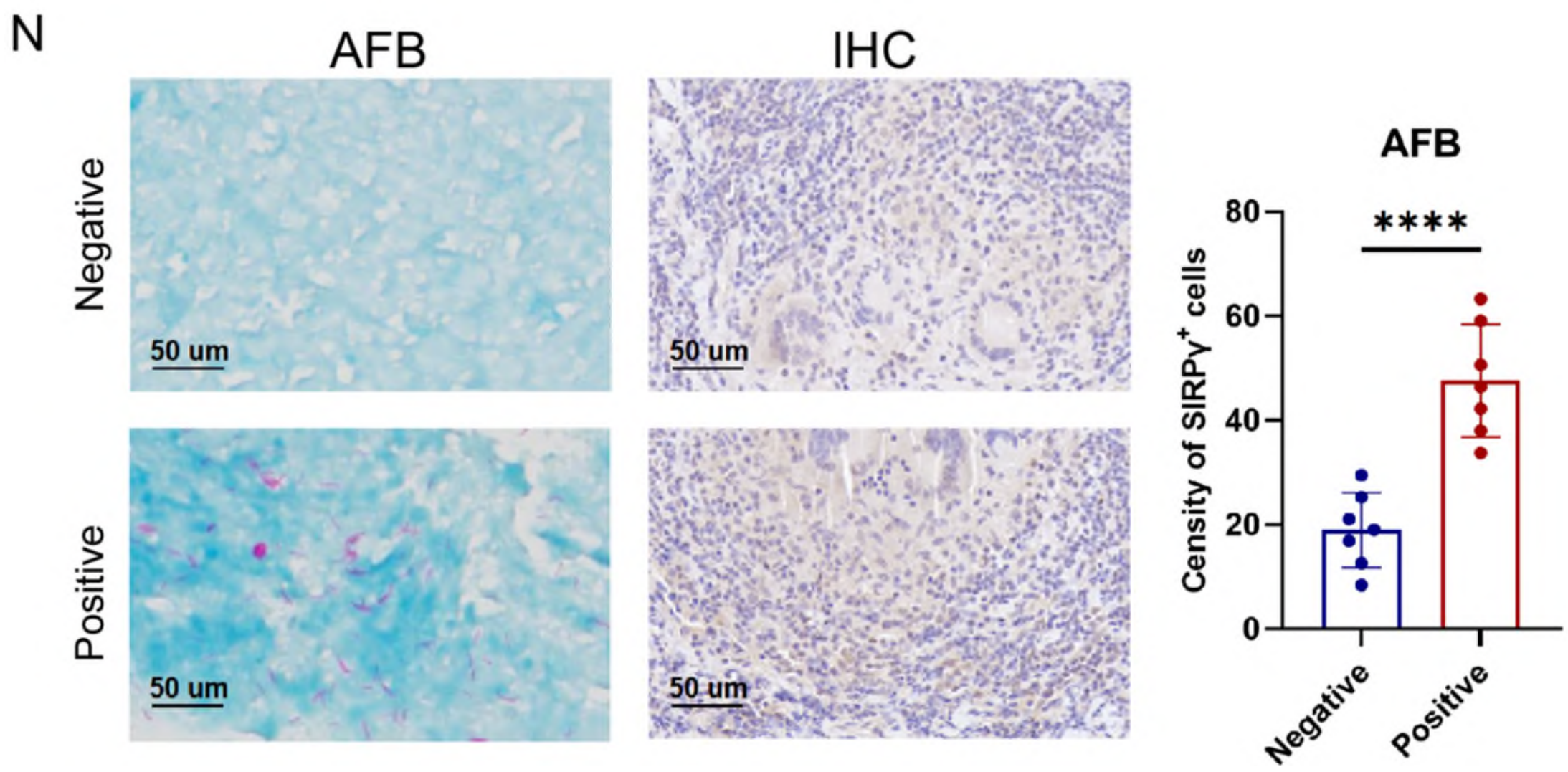

(N) Representative IHC detection of SIRPγ and AFB stains in pleural tissues are displayed with corresponding quantitative results of SIRPγ$^+$ cell density (n = 7).

4. *The authors compare SIRPy expression in TPE versus peripheral blood from tuberculosis patients (lines 169–173, Fig. 2) and report higher expression in CD8+ T cells from TPE. Was SIRPy expression also compared across different immune cell subsets between TPE and MPE? Is SIRPy expression in CD8+ T cells significantly higher in TPE than in MPE? Additionally, what is the expression level of SIRPy in exhausted T cells (Tex) from MPE patients?*

Response: Thank you for your questions. We have analyzed SIRPγ expression across immune cell subsets in MPE samples. Our results show that, similar to TPE, SIRPγ in MPE is predominantly expressed in CD4$^+$ and CD8$^+$ T cells, with only minimal expression observed in NK cells. Furthermore, SIRPγ expression on CD8$^+$ T cells, CD4$^+$ T cells, and NK cells was significantly higher in TPE than in MPE, further underscoring its potential as a diagnostic biomarker for TPE (**Fig. S3F, G, Page 7, line 182-186)**.

In addition, we sincerely appreciate the reviewer's insightful question regarding the expression of SIRPγ in exhausted T cells (Tex) from MPE patients. In our preliminary analysis of scRNA-seq data (data not shown), we observed that SIRPγ appears to be relatively enriched in the Tex subset compared to other T cell populations

in MPE. Thus, the role of SIRPγ⁺ CD8⁺ T cells in MPE is currently being investigated as part of our study.

Supplementary Figure 3F

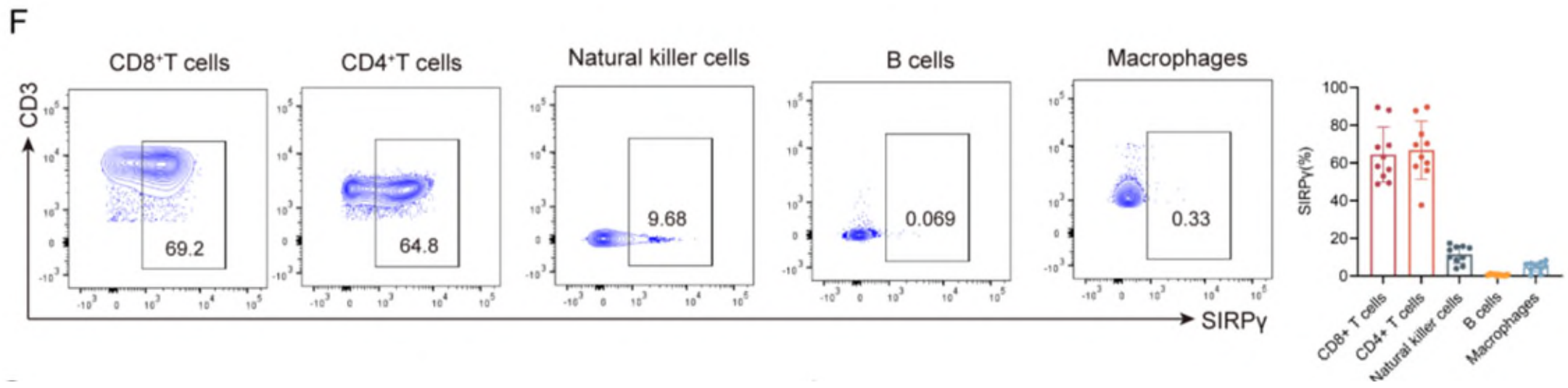

(F) Representative flow cytometric plots and corresponding statistical analyses showing the proportions of SIRPγ-positive cells from MPE, including SIRPγ^{+} B cells (CD45^{+} CD19^{+} SIRPγ^{+}), SIRPγ^{+} natural killer cells (CD45^{+} CD3^{+} CD56^{+} SIRPγ^{+}), SIRPγ^{+} macrophages (CD45+CD68+SIRPγ+), SIRPγ^{+}CD4+ T cells (CD3^{+} CD4^{+}SIRPγ^{+}), and SIRPγ^{+}CD8^{+} T cells (CD3^{+} CD8^{+} SIRPγ^{+}) (n = 10).

Supplementary Figure 3G

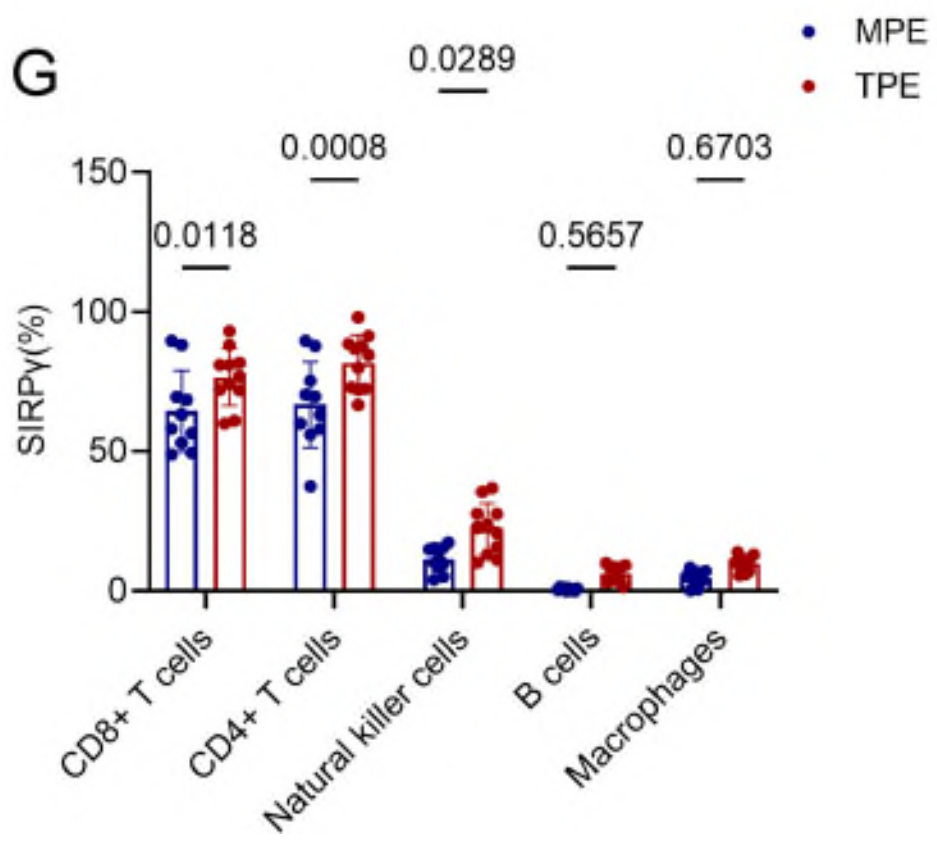

(G) Comparative analysis of SIRPγ expression levels in different immune cell populations between MPE and TPE (n=10-11).

5. *In lines 189–205, the authors describe in vitro stimulation of PBMCs from PPD+ donors with ESAT-6-CFP-10 antigen and observe SIRPy-promoted CD8+ T cell exhaustion. However, the duration of antigen stimulation is not specified in the Methods. Typically, such experiments do not exceed 24 hours. How can the observed effect be*

attributed to "chronic stimulation" when a short-term stimulus was likely used? Would "immediate stimulation" be a more accurate description?

Response: We thank the reviewer for pointing out the incomplete description of the stimulation protocol and for raising the important distinction between acute and chronic stimulation. We apologize for the omission and have now clarified the experimental details in the Methods section as follows (**Page 24, line 678-681)**: “Where applicable, PBMCs from PPD-positive volunteers and TPE patients were stimulated with E-C (10 μg/mL) at 37°C for 20 hours, after which the antigen-containing medium was removed and cells were cultured for special hours before analysis.” Accordingly, we have replaced “chronic stimulation” with “immediate stimulation” in the manuscript to more accurately describe the experimental conditions (**Page 8, line 222-227)**.

6. *Through differential gene expression analysis, the authors identified associations between SIRPγ-mediated T cell exhaustion and glycine, serine, and threonine metabolism pathways (lines 239–241). However, only the serine metabolism pathway was pursued experimentally. What is the rationale for focusing exclusively on serine metabolism, and were other relevant pathways explored?*

Response: We thank the reviewer for raising this important question. Our focus on serine metabolism was based on a stepwise filtering strategy.

1) Candidate gene screening and functional validation

Initial GSEA revealed enrichment of glycine, serine, and threonine metabolism pathways, from which three candidate genes (PHGDH, GCAT, and ALDH7A1) were identified (Fig. 4C). PHGDH encodes phosphoglycerate dehydrogenase, the rate-limiting enzyme in the de novo serine biosynthesis pathway. It plays a central role in regulating serine production and serves as a key metabolic node linking central carbon metabolism to serine metabolism. In contrast, ALDH7A1 is primarily involved in lysine degradation and is only indirectly associated with glycine, serine, and threonine metabolism. GCAT encodes an enzyme that catalyzes the interconversion between threonine and glycine, facilitating threonine catabolism.

Further single-cell data stratification revealed that among these candidates, only

PHGDH and GCAT were specifically upregulated in SIRPγ$^+$ CD8$^+$ T cells and strongly correlated with SIRPG expression, whereas ALDH7A1 showed no significant difference at the transcriptional level (Fig. 4D, E; Fig. S8A–C). SIRPγ blockade experiments confirmed that blocking SIRPγ selectively downregulated PHGDH expression but did not significantly affect GCAT or ALDH7A1 (Fig. 4F; Fig. S8D, E). These findings suggest that PHGDH-mediated serine synthesis represents a core node regulated by SIRPγ.

2) Metabolic hub status

Our newly added U-[^{13}C]-glucose tracing data further demonstrated that glucose-derived serine synthesis and one-carbon metabolism were significantly enhanced in SIRPγ$^+$ cells, whereas no significant change was observed in the glycine pool **(Fig. 4M, Fig. S8G – J, Page 11, line 297–307; Page 28, line 787-796**).

As the rate-limiting enzyme in de novo serine synthesis, PHGDH connects glycolysis to one-carbon metabolism. Given its position as a key hub in this metabolic network and its potential as a druggable target, we selected PHGDH-mediated serine metabolism as the focus for functional investigation. In addition, we have added a discussion in the Discussion section (**Page 20, line 563-568**) acknowledging the metabolic interconnection among glycine, serine, and threonine pathways. Glycine and serine can be interconverted and participate in one-carbon metabolism; while our stepwise screening identified PHGDH-mediated serine metabolism as a key node downstream of SIRPγ, the potential contribution of threonine metabolism to CD8$^+$ T cell exhaustion cannot be excluded and warrants further investigation.

Fig. 4N

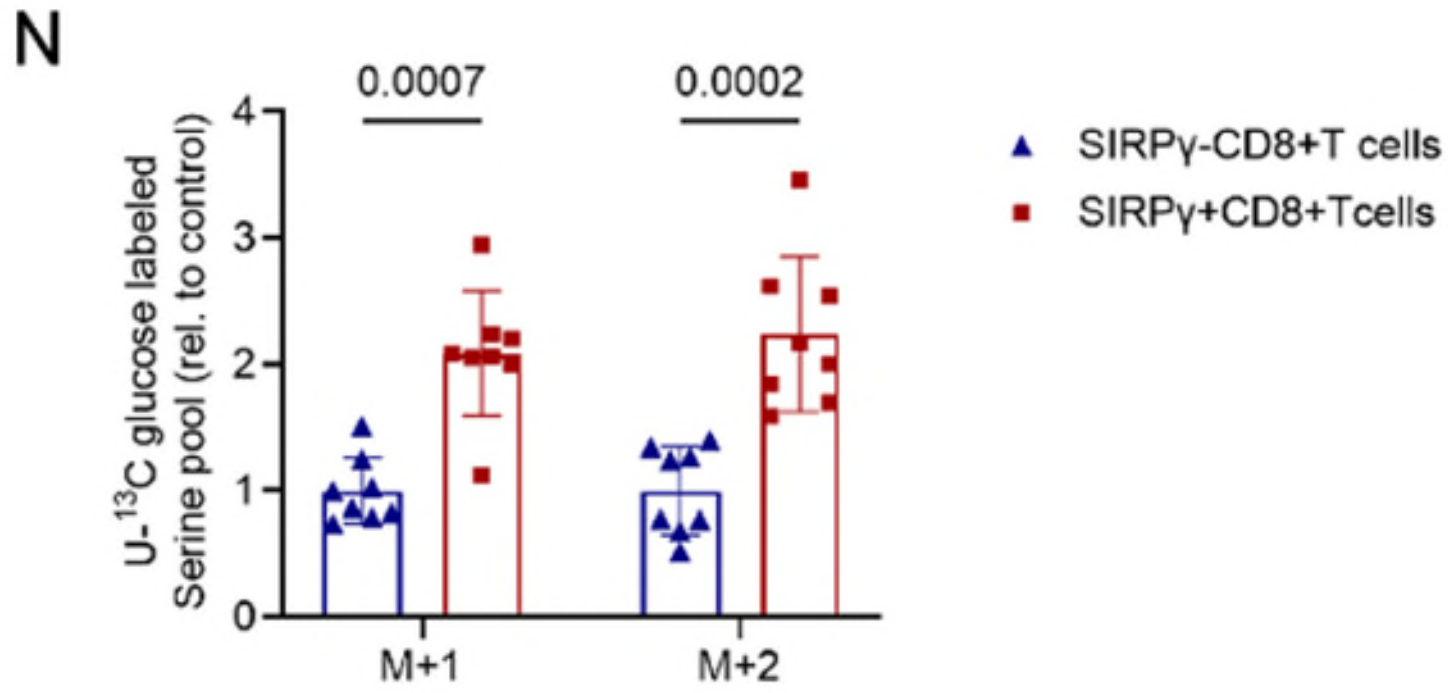

(N) Incorporation of U-[^{13}C]-glucose carbon into serine in sorted SIRPγ^{-} and SIRPγ^{+} CD8^{+} T cells derived from TPE (n=8).

Supplementary Figure 8G-J

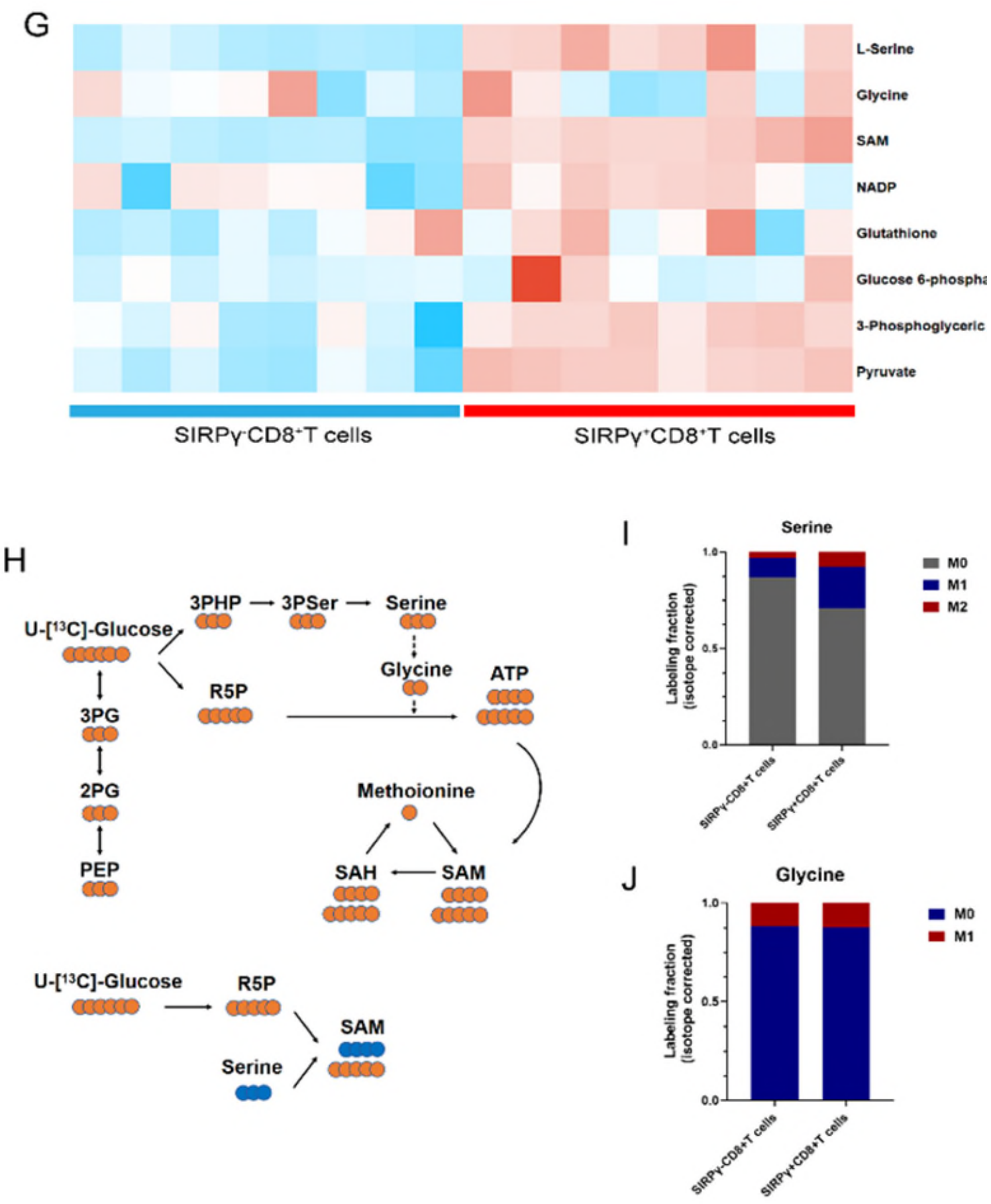

(G–J) SIRPγ^{-} and SIRPγ^{+} CD8^{+} T cells isolated from TPE were incubated with U-[^{13}C]-glucose for six hours. Heatmaps (G) and schematic representations (H) illustrate the incorporation of U-[^{13}C]-glucose into various metabolites. The percent distribution of each isotopic isomer in the serine (I) and glycine (J) pool is shown (n=8).

7. *The study lacks direct experimental evidence supporting SIRPy as a therapeutic target. It is recommended that claims regarding SIRPy's potential as a therapeutic target be removed from the title, abstract, and conclusion sections to maintain scientific rigor.*

Response: We sincerely thank the reviewer for this insightful comment. We fully acknowledge that our current study does not provide direct experimental evidence to support SIRPγ as a therapeutic target, and it is premature to propose SIRPγ as a therapeutic target. We have revised the manuscript to maintain scientific rigor. Specifically, we have:

Title：Removed the phrase "therapeutic target" from the title and replaced with "SIRPγ serves as a potential diagnostic biomarker and promotes $CD8^+$ T cell exhaustion in tuberculous pleural effusion" **(Page 1, line 1-2)**.

Abstract: Modified the abstract to focus on the diagnostic implications of SIRPγ expression rather than therapeutic potential **(Page 3, line 62-63).**

Conclusion: Revised the conclusion section to avoid overstatement and to reflect the observational nature of our findings， now reading: "This study comprehensively elucidates the dual role of SIRPγ in TPE, highlighting its function not only as a potential diagnostic biomarker but also as a key regulator of $CD8^+$ T cell exhaustion. SIRPγ detection therefore not only offers diagnostic insight but also serves as a potential indicator of local immune exhaustion, providing a clear rationale for future therapeutic strategies aimed at restoring T-cell function through targeted SIRPγ blockade." **(Page 22, line 628–633)**.

Response to Reviewers

Dear reviewers,

Thank you very much for your comments and professional advice. Based on your suggestions, we have carefully revised the manuscript and provided the point-by-point response below. All modifications in the manuscript have been highlighted in yellow. If any responses are unclear or you wish additional changes, please do not hesitate to contact us.

Reviewer #1 (Remarks to the Author):

Yang and colleagues have satisfactorily addressed all comments from the initial review. The revised manuscript is significantly improved, both in terms of presentation and scientific content. I have no major concerns and recommend acceptance upon minor revisions and thorough text editing.

Minor concerns:

1. Line 218: "the molecular mechanism…" should start with capital letter "The molecular mechanism…"

Response: We sincerely thank the reviewer for pointing out this grammatical error. We have corrected the phrase in line **now 221** to now read: "The molecular mechanism…..".

2. Line 228: "consistent with previous finding…" should read either "consistent with a previous finding…" or "consistent with previous findings…"

Response: We thank the reviewer for this insightful comment. we have revised the sentence in line 228 (**Page 9, line 230-231**) to read: "consistent with a previous finding….".

3. Line 232: the word "implicating" should be replaced by the word "implying"

Response: We thank the reviewer for this careful reading and helpful suggestion. The word has been corrected from "implicating" to "implying" in the revised manuscript

(Page 9, line 235).

4. Line 358: "To counteract the exhaustion phenotype reduced by SG deprivation…" should be rephrased into "To bring back the exhaustion phenotype which is reduced by SG deprivation…" or "To rescue the exhaustion phenotype reduced by SG deprivation…

Response: Thank you for your suggestion. we have revised the sentence in line 358 (**Page 13, line 361**) to read: "To rescue the exhaustion phenotype reduced by SG deprivation…".

5. Line 389: "However, the precise role of SAM in modulating histone methylation within CD8$^+$ T cells remain incompletely understood" should read "However, the precise role of SAM in modulating histone methylation within CD8$^+$ T cells remains incompletely understood"

Response: We thank the reviewer for this insightful comment. we have revised the sentence in line 358 (**Page 14, line 391-393**) to read: "However, the precise role of SAM in modulating histone methylation within CD8$^+$ T cells remains incompletely understood".

Reviewer #2 transmitted their comments directly to the editors

Reviewer #3 (Remarks to the Author):

1. Based on the IHC images presented in Fig. 1N and Fig. S2A, SIRPγ staining appears more evident in multinucleated giant cells and epithelioid cells, whereas it appears relatively weak in lymphocytes. In Fig. S2B, only about four cells exhibit clear co-localization of SIRPγ and CD3+ signals, with the majority of SIRPγ-positive cells showing no overlap with CD3+ labeling. Given these observations, how do the authors justify the conclusion that SIRPγ is specifically and highly expressed in CD3+ T cells?

Response: We sincerely appreciate the reviewer's insightful comments. In response to

the concerns raised regarding the cellular identity of SIRPγ-expressing cells, we provide the following point-by-point clarifications and revisions:

1) Discrepancies in SIRPγ Staining Across Cell Types in IHC

We thank the reviewer for this observation. Initially, we employed Image J thresholding to clearly distinguish between positive and negative signals; our quantitative analysis confirmed that SIRPγ expression is predominantly enriched in the region surrounding the granuloma rather than the central areas. However, upon further review, we acknowledged that the original images might cause potential misinterpretation. To address this, we optimized the staining and antigen retrieval protocols and validated the results across multiple antibody batches. The optimized IHC results demonstrate that SIRPγ-positive signals are more densely concentrated in the perigranulomatous regions, where the cellular morphology is more consistent with lymphocytes. We have added relevant descriptions to the Results section and updated the representative images in the revised manuscript **(Page 6, line 163–169; Fig. 1M, Fig. S2A)**.

2) Cross-validation with Single-Cell Transcriptomic Data

We mined and analyzed the published single-cell RNA sequencing dataset of tuberculous-infected human lung (GSE192483). The cellular atlas of these tissues primarily comprised T cells, natural killer (NK) cells, macrophages, neutrophils, B cells, dendritic cells, mast cells, epithelial cells, and MITO (mitochondrial)-high cells. These scRNA-seq data align closely with previous literature (Blood. 2008 Aug 15;112(4):1280-9), consistently supporting that *SIRPG* expression is highest in T cells, present at low levels in NK cells, and virtually absent in macrophages and epithelioid cells **(Page 7, line 175-179; Fig. S3A–D)**.

3) Explanation for the Low Co-localization Ratio of SIRPγ and CD3 in Fig. S2B

We are grateful for the reviewer's keen observation. We agree that our initial description of "SIRPγ is specifically and highly expressed in CD3+ T cells" was not entirely accurate based on the provided immunofluorescence images. Consequently, we have revised the manuscript to state: Immunofluorescence staining further demonstrated that SIRPγ is highly expressed on T cells, whereas macrophages showed

little to no expression of this protein **(Page 7, line 180–182)**. Regarding the observation in Fig. S2B that a subset of SIRPγ^{+} cells did not co-localize with CD3, it is important to note that the tuberculous granuloma represents a complex immune microenvironment. Beyond multinucleated giant cells and epithelioid cells, it is infiltrated by various lymphocytes, including T cells and innate lymphoid cells (ILCs)/NK cells (J Exp Med. 2026 Mar 2;223(3):e20251067; J Infect. 2023 Nov;87(5):373-384; mBio. 2025 Sep 10;16(9):e0155925). Both our scRNA-seq analysis and flow cytometry data confirm that activated Natural Killer (NK) cells also express SIRPγ. Given the limited field of view in tissue sections, the specific area captured likely includes infiltrating NK cells. Since NK cells are inherently CD3-negative, the SIRPγ^{+}/CD3^{-} population observed in this field likely corresponds to these NK cells. After re-sectioning and staining, the new immunofluorescence staining demonstrated that the majority of SIRPγ-positive cells overlapped with CD3+ labeling **(Fig S4A)**. Collectively, our study indicates that SIRPγ is highly expressed on T cells in the pleural effusion and pleural tissue of TPE patients, with potential minor expression on NK cells.

Fig 1M

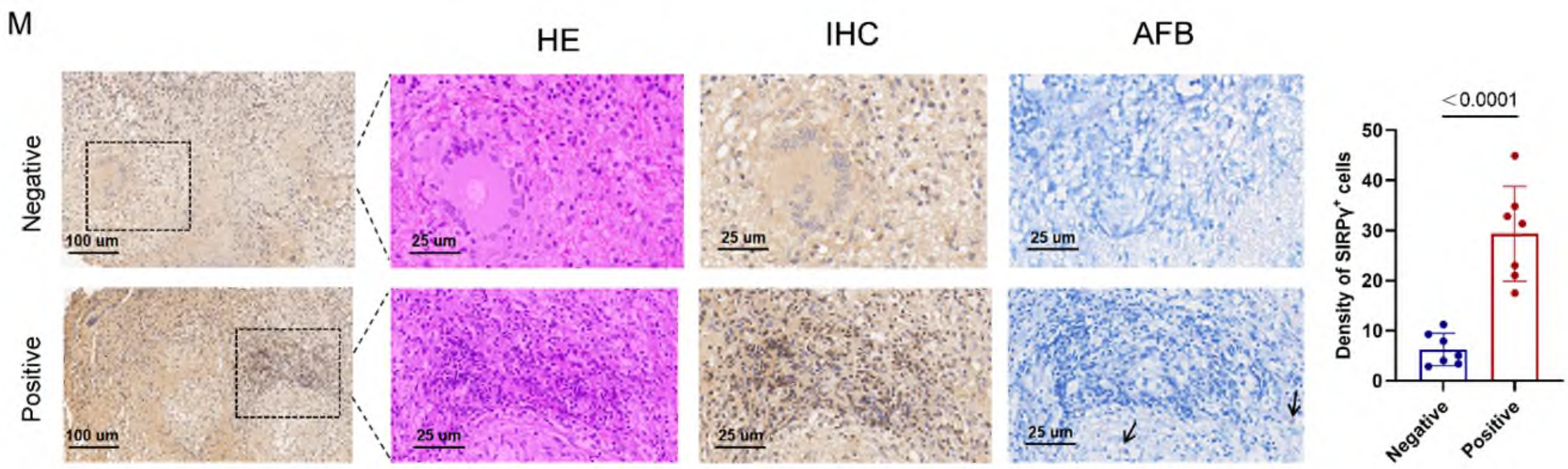

(M) Representative hematoxylin and eosin (H&E), immunohistochemistry (IHC) detection of SIRPγ, and acid-fast bacteria stain (AFB) stains in pleural tissues are displayed with corresponding quantitative results of SIRPγ^{+} cell density (n = 7).

Fig S2A

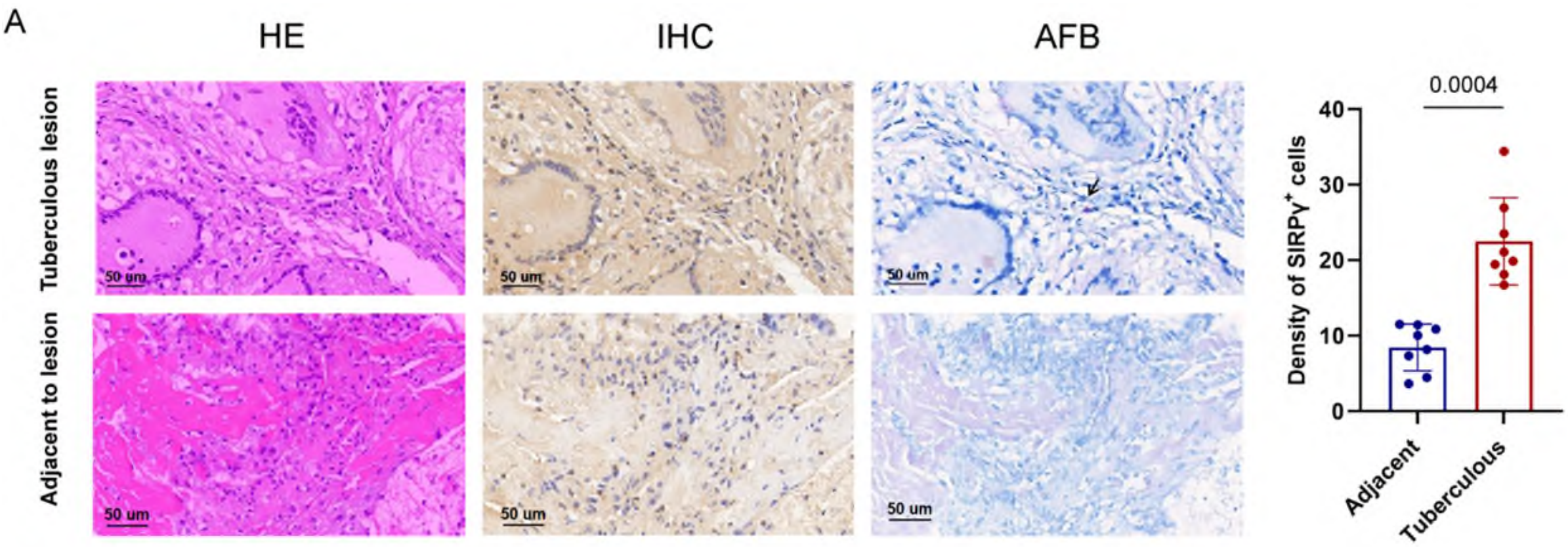

(A) Representative hematoxylin and eosin (H&E), immunohistochemistry (IHC) detection of SIRPγ, and acid-fast bacteria stain (AFB) stains in pleural tissues are displayed with corresponding quantitative results of SIRPγ$^+$ cell density (n = 8).

Fig S3A-D

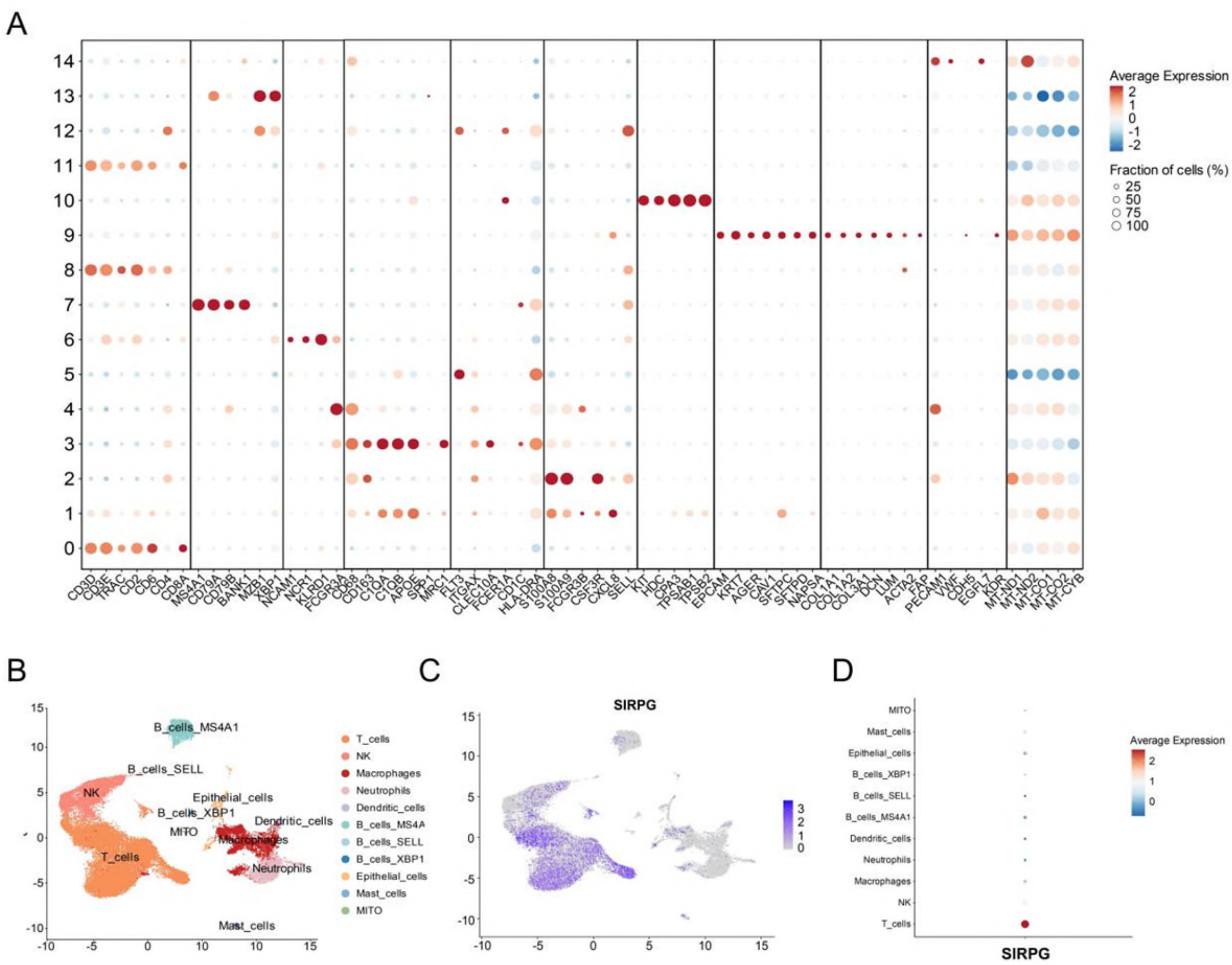

Supplementary Figure 3 SIRPγ is highly expressed in exhausted CD8$^+$ T cells in TPE, related to Figure 2. (A) Gene expression dot plot showing unique expression patterns to define 11 distinct cell populations in B. (B) UMAP representation of cell

clusters from combined 5 pulmonary tuberculosis tissue samples. (C) UMAP feature plot showing the expression of SIRPG of all cells. (D) Dot plot showing SIRPG expression patterns of the annotated cell populations. NK, natural killer cells; MITO, mitochondrial transcripts.

2. The acid-fast staining and IHC images in Fig. 1N do not appear to represent the same tissue region. In the current Fig. 1N, an increase in SIRPγ-positive lymphocytes within acid-fast-positive granulomas is not clearly seen. Authors should provide WSIs—including HE staining, IHC staining, and acid-fast staining, along with corresponding magnified views—for both acid-fast-positive and acid-fast-negative lesions.

Response: We thank the reviewer for their meticulous evaluation. We respectfully clarify that the acid-fast staining and immunohistochemistry (IHC) images in the original Figure 1N were indeed derived from the same tissue region. The subtle morphological discrepancies observed are likely attributable to the inherent limitations of serial sectioning, where minor tissue deformation or folding during processing can preclude perfect field alignment. Furthermore, the intrinsic characteristics of the acid-fast bacteria (AFB) staining technique may obscure local cellular and tissue morphology, potentially contributing to this misinterpretation.

To eliminate ambiguity and ensure rigorous data presentation, we have updated Fig. 1N **(now Fig. 1M)** in the revised manuscript. We now provide low-magnification panoramic views of the IHC-stained sections to illustrate the overall lesion architecture, alongside high-magnification images of corresponding regions from serial sections stained with HE, IHC, and AFB. These revised images clearly demonstrate a marked increase in the abundance of SIRPγ-positive cells within acid-fast-positive granulomas compared to their acid-fast-negative counterparts.

Fig. 1M

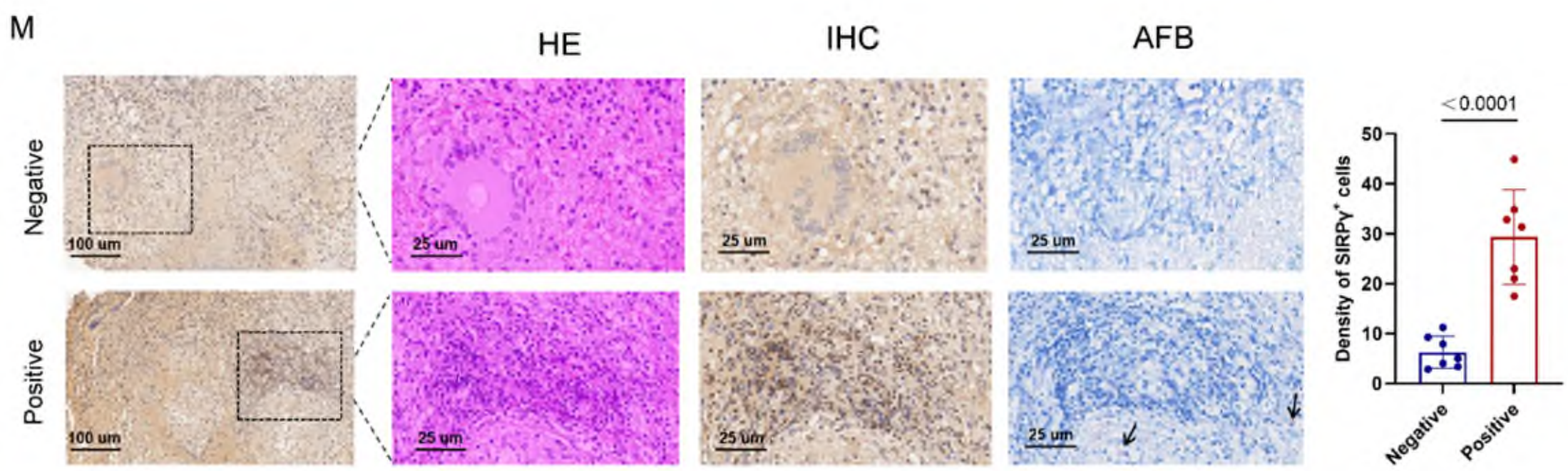

(M) Representative hematoxylin and eosin (H&E), immunohistochemistry (IHC) detection of SIRPγ, and acid-fast bacteria stain (AFB) stains in pleural tissues are displayed with corresponding quantitative results of $SIRP\gamma^+$ cell density (n = 7).

3. How were the samples for the cytometric experiments obtained? The results shown in Figure S3F and S3G indicate that SIRPγ expression in macrophages is notably low. However, the IHC staining results suggest that SIRPγ-positive signals are predominantly localized to multinucleated giant cells and epithelioid cells.

Response: We thank the reviewer for raising this important point. In response, we provide the following clarifications and revisions:

1) Sample Source and Identification for Flow Cytometry:

The samples used for flow cytometry were peripheral blood mononuclear cells (PBMCs) or pleural effusion-derived mononuclear cells (PEMCs) isolated from patients with TPE or MPE.For the identification of SIRPγ-expressing subsets, cells were stained with an anti-human SIRPγ antibody (clone LSB2.20, BioLegend, PE-conjugated). After excluding doublets and dead cells, immune cells were gated based on forward/side scatter. SIRPγ expression was then analyzed on macrophages ($CD45^+CD3^-CD68^+$), B cells ($CD45^+CD3^-CD19^+$), NK cells ($CD45^+CD3^-CD56^+$), and $CD8^+$ T cells ($CD45^+CD3^+CD8^+$). The cutoff for positivity was defined using isotype control background. Gating strategy is provided in Fig. S14, and the panels were listed in table S5 **(Page 25, line 692-693; Page 25, line 703-710)**.

2) Re-evaluation of IHC Staining:

Upon careful re-evaluation, we recognized that the strong signals previously observed in multinucleated giant cells and epithelioid cells were likely attributable to

non-specific antibody binding, which could have led to data misinterpretation. To address this, we optimized the IHC protocol to minimize background noise and ensure specificity. The revised IHC staining clearly demonstrates that SIRPγ is predominantly expressed in the region surrounding the granuloma. Immunofluorescence staining further demonstrated that SIRPγ is highly expressed on T cells, whereas macrophages showed little to no expression of this protein. This finding aligns with our flow cytometry and scRNA-seq data, which indicate low SIRPγ expression on macrophages and epithelioid cells but high expression on T cells. We have updated the figures and corresponding descriptions in the revised manuscript to reflect these accurate results **(Fig. 1M, Fig. S2A, Fig. S3A-D, Fig. S4A, B)**.

Response to Reviewers

Dear reviewers,

Based on your suggestions, we have carefully revised the manuscript and provided the point-by-point response below.

Reviewer #3 (Remarks to the Author):

1. The conclusion "SIRP γ is predominantly expressed on T cells and weakly on macrophages" remains unsupported by the data. The HE, IHC, and immunofluorescence results are insufficient to distinguish expression levels between these cell types, and several images do not match the descriptions, with poor quality.

Response: We sincerely thank the reviewer for their thorough and rigorous re-evaluation of our manuscript. Your insightful comments have greatly contributed to improving our work. Regarding the concern that our conclusion "SIRPγ is predominantly expressed on T cells and weakly on macrophages" lacks sufficient data support, and that H&E, IHC, and immunofluorescence (IF) staining alone are inadequate to distinguish expression levels across different cell types, we have taken the following measures. First, we supplemented our IF results with quantitative statistical analyses. Specifically, we used IF staining to define cell types and simultaneously assess SIRPγ expression levels, with the corresponding quantitative data now presented in Supplementary Figure S4a, b. Quantitative co-localization analysis revealed that the majority of SIRPγ$^+$ cells were CD3$^+$, whereas CD68$^+$ macrophages showed little SIRPγ signal.

In addition, as described in our previous revision, we further validated our findings using a published single-cell RNA sequencing dataset derived from tuberculous human lungs (GSE192483). This independent dataset confirmed that SIRPG is most abundantly expressed in T cell clusters, while remaining nearly undetectable in macrophage clusters. These results have been incorporated into the Results section **(Page 7, lines 175–179; Fig. S3A–D)**. Collectively, these lines of evidence support our

assertion that SIRPγ is predominantly expressed on T cells and only weakly on macrophages.
