# Supplementary material for: SIRPγ promotes CD8⁺ T cell exhaustion via metabolic-epigenetic reprogramming in tuberculous pleural effusion
Source: Nat Commun. 2026 Sep 25;17:11144. doi: 10.1038/s41467-026-77779-4 (PMC13710978; doi:10.1038/s41467-026-77779-4)
Supplement: Supplementary file 4 [file 41467_2026_77779_MOESM4_ESM.zip › Raw data/Raw data-2.pdf]

Full unedited gels for the manuscript

Full unedited gel for Fig. 4i

Representative images (red boxes)

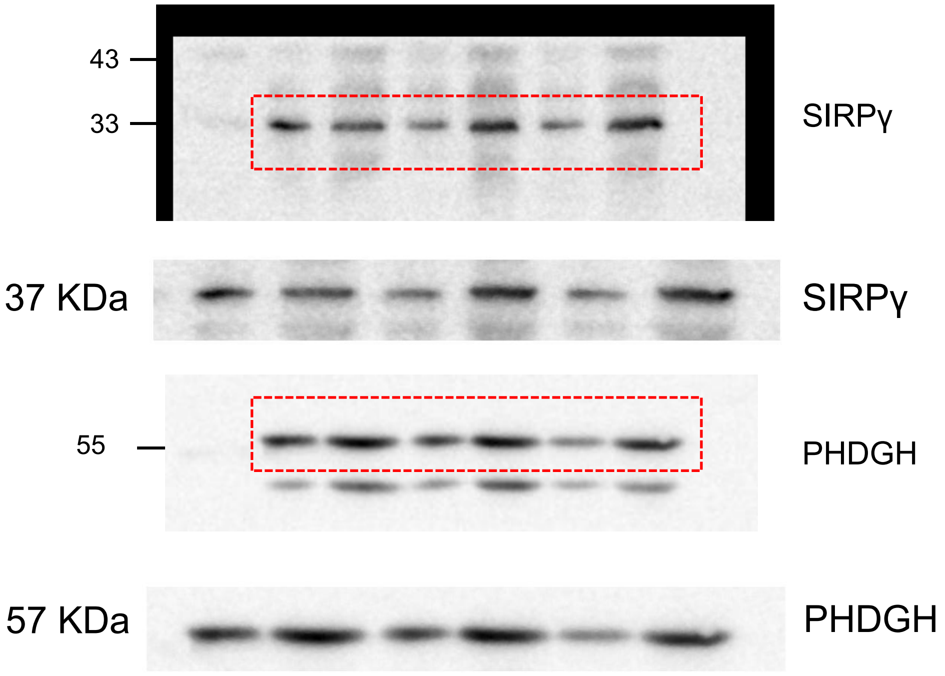

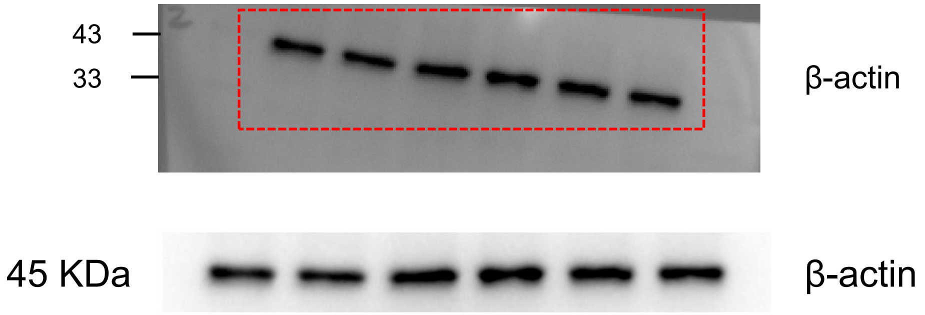

Full unedited gel for Fig. 4j

Representative images (red boxes)

& Images for quantification (blue boxes)

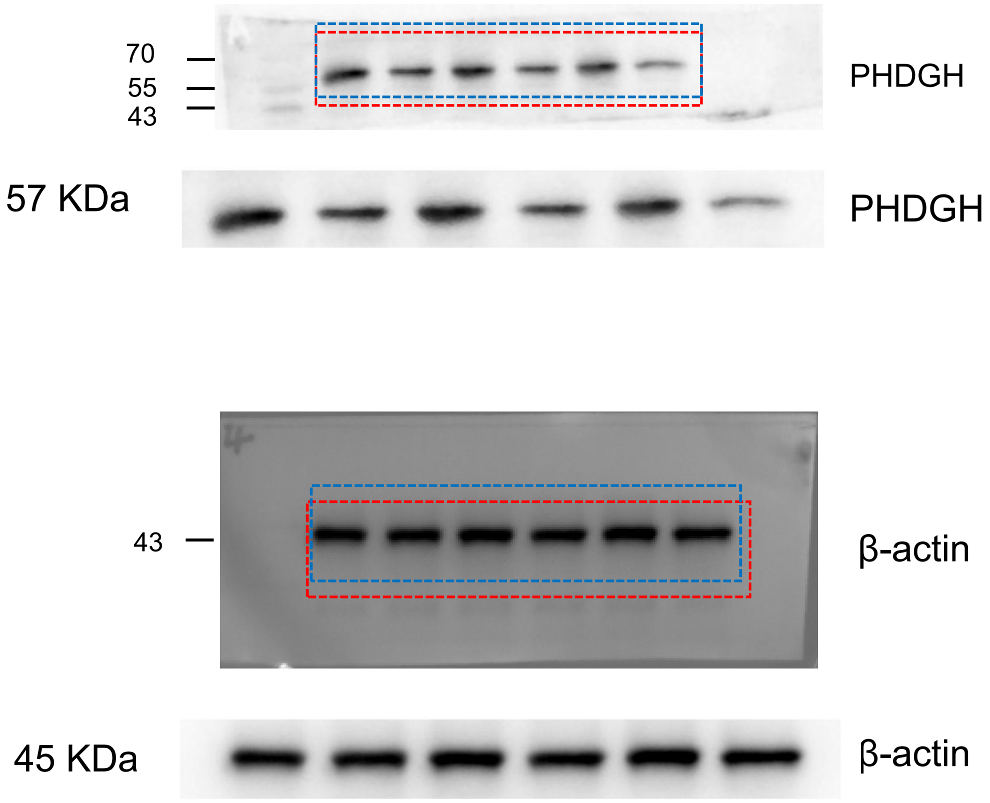

Full unedited gel for Fig. 7g and Fig. S14e

Representative images (red boxes)

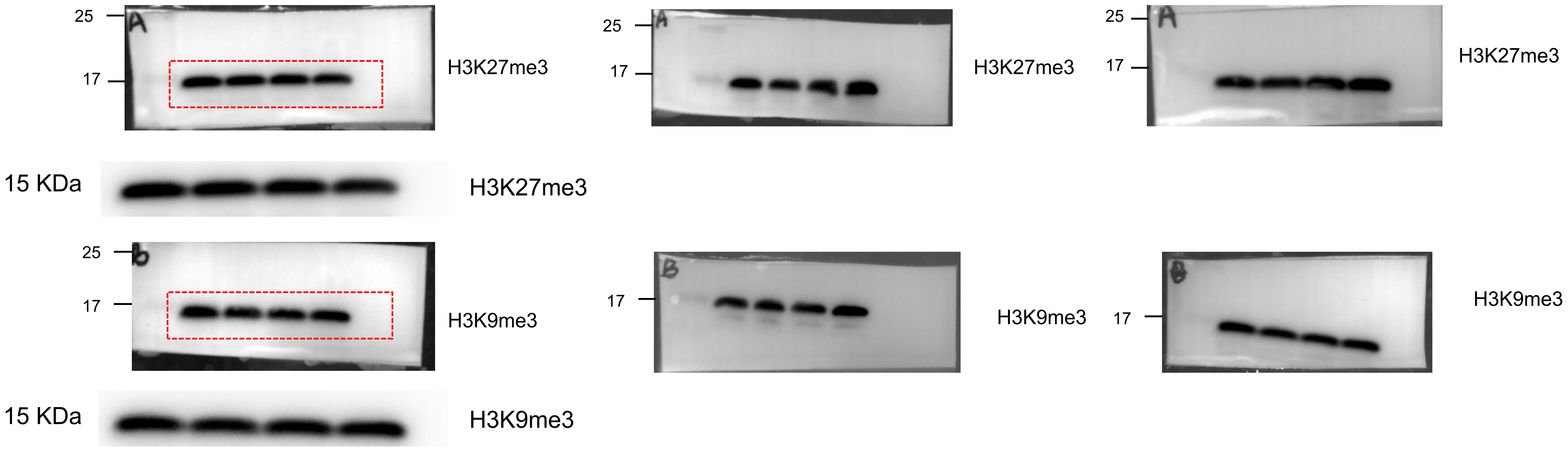

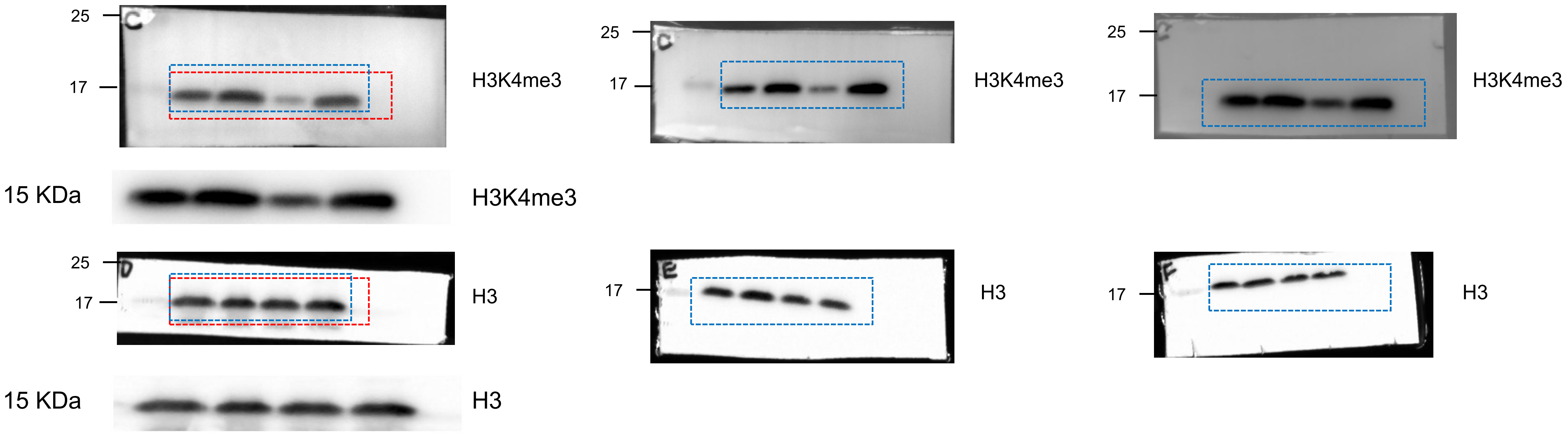
25
17
H3K4me3
15 KDa
H3K4me3
25
17
H3
15 KDa
H3
25
17
H3K4me3
17
H3
25
17
H3K4me3
17
H3

Full unedited gel for Fig. 7k and Fig. S14h, i

Representative images (red boxes)

& Images for quantification (blue boxes)

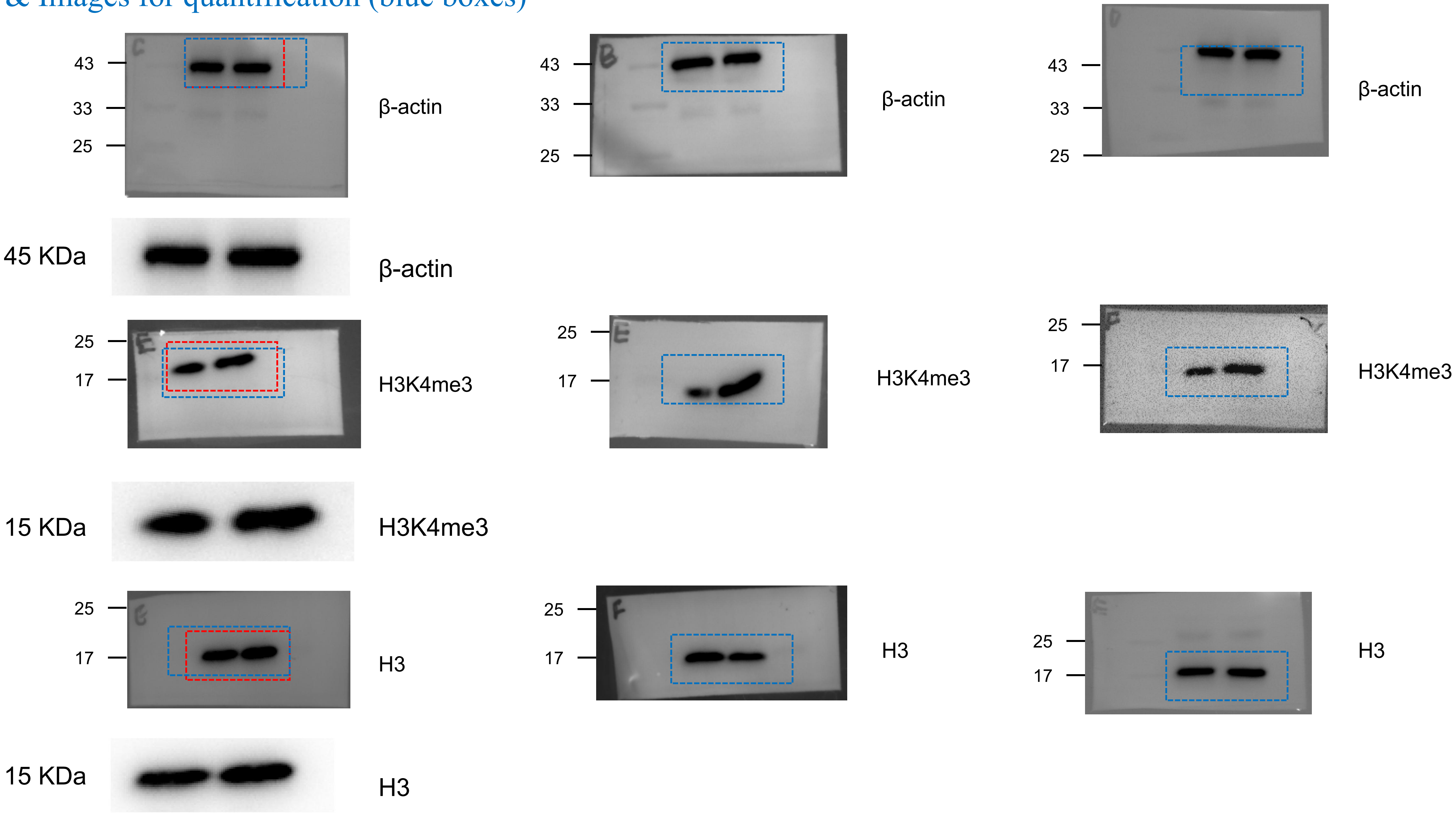

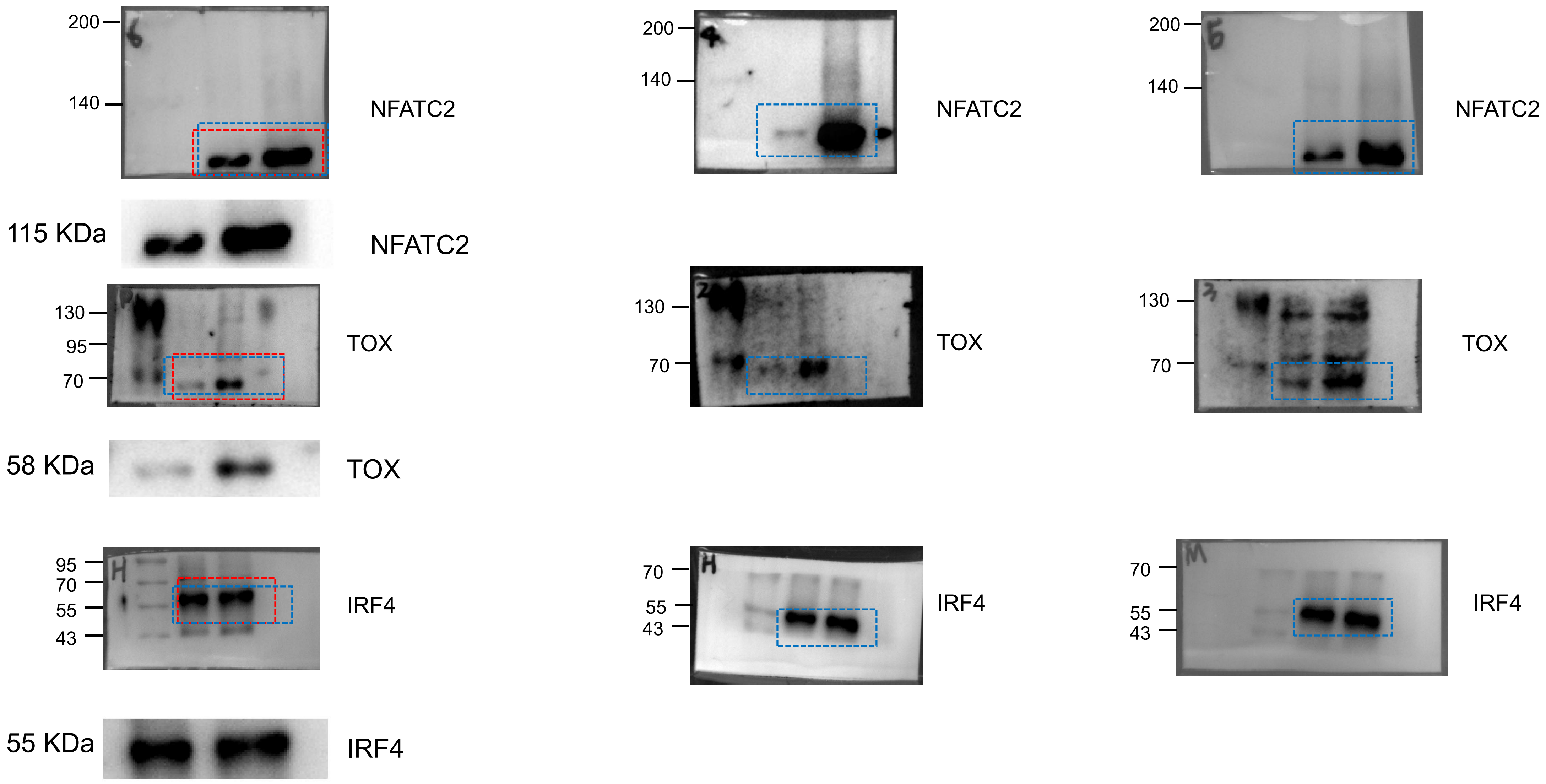

200
140
NFATC2
115 KDa
NFATC2
130
95
70
TOX
58 KDa
TOX
95
70
55
43
IRF4
55 KDa
IRF4
200
140
NFATC2
130
70
TOX
70
55
43
IRF4
200
140
NFATC2
130
70
TOX
70
55
43
IRF4

Full unedited gel for Fig. S14f

Representative images (red boxes)

& Images for quantification (blue boxes)

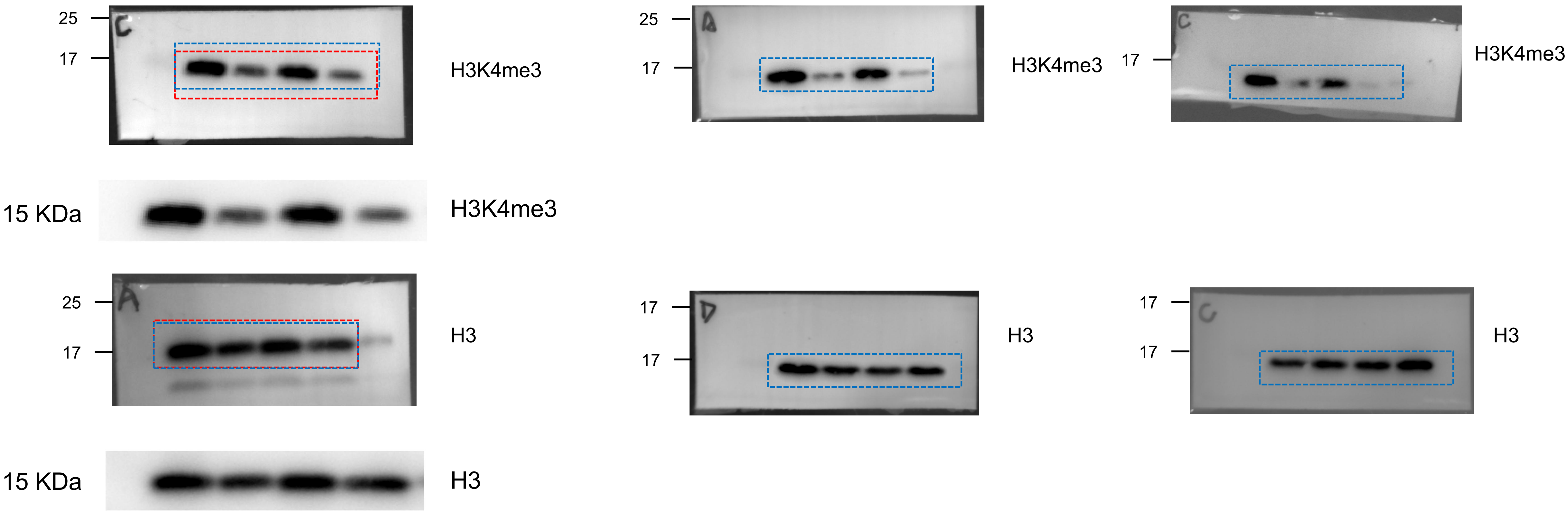

Full unedited gel for Fig. 8b

Representative images (red boxes)

& Images for quantification (blue boxes)

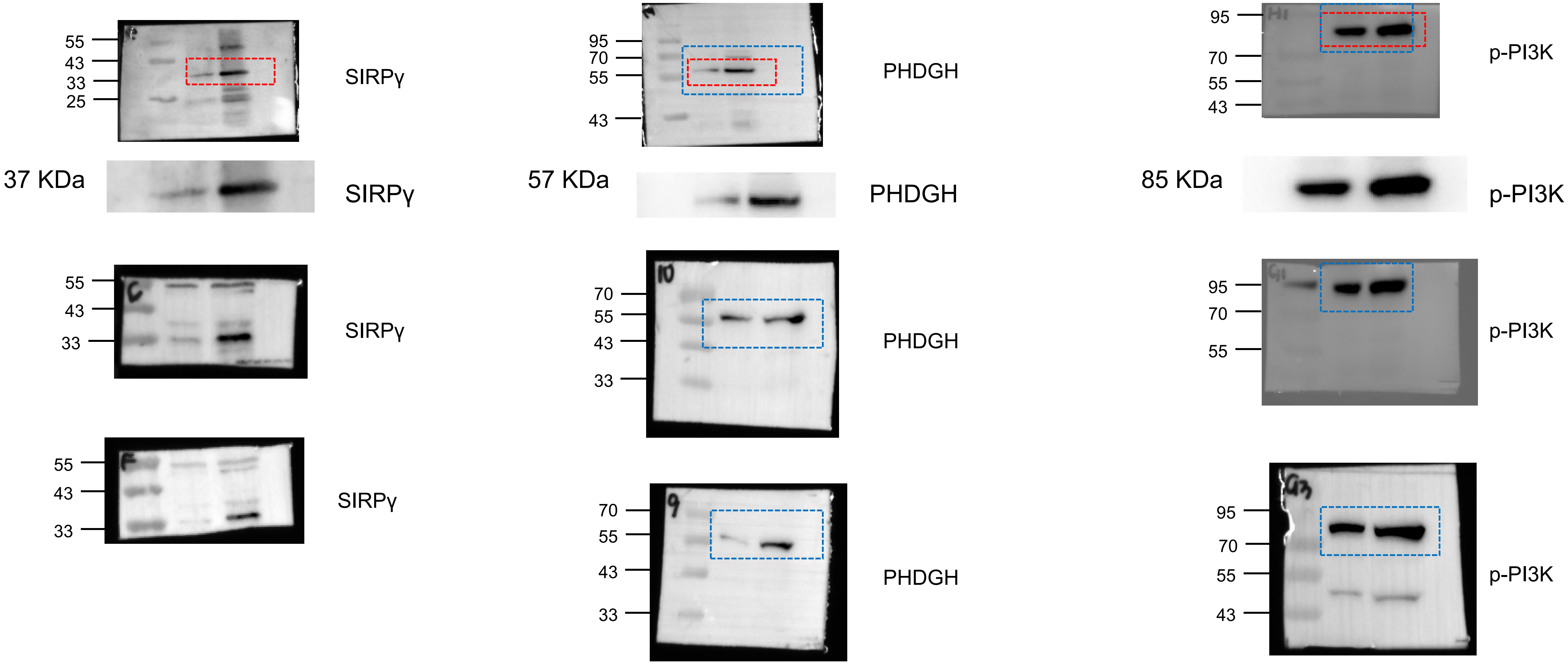

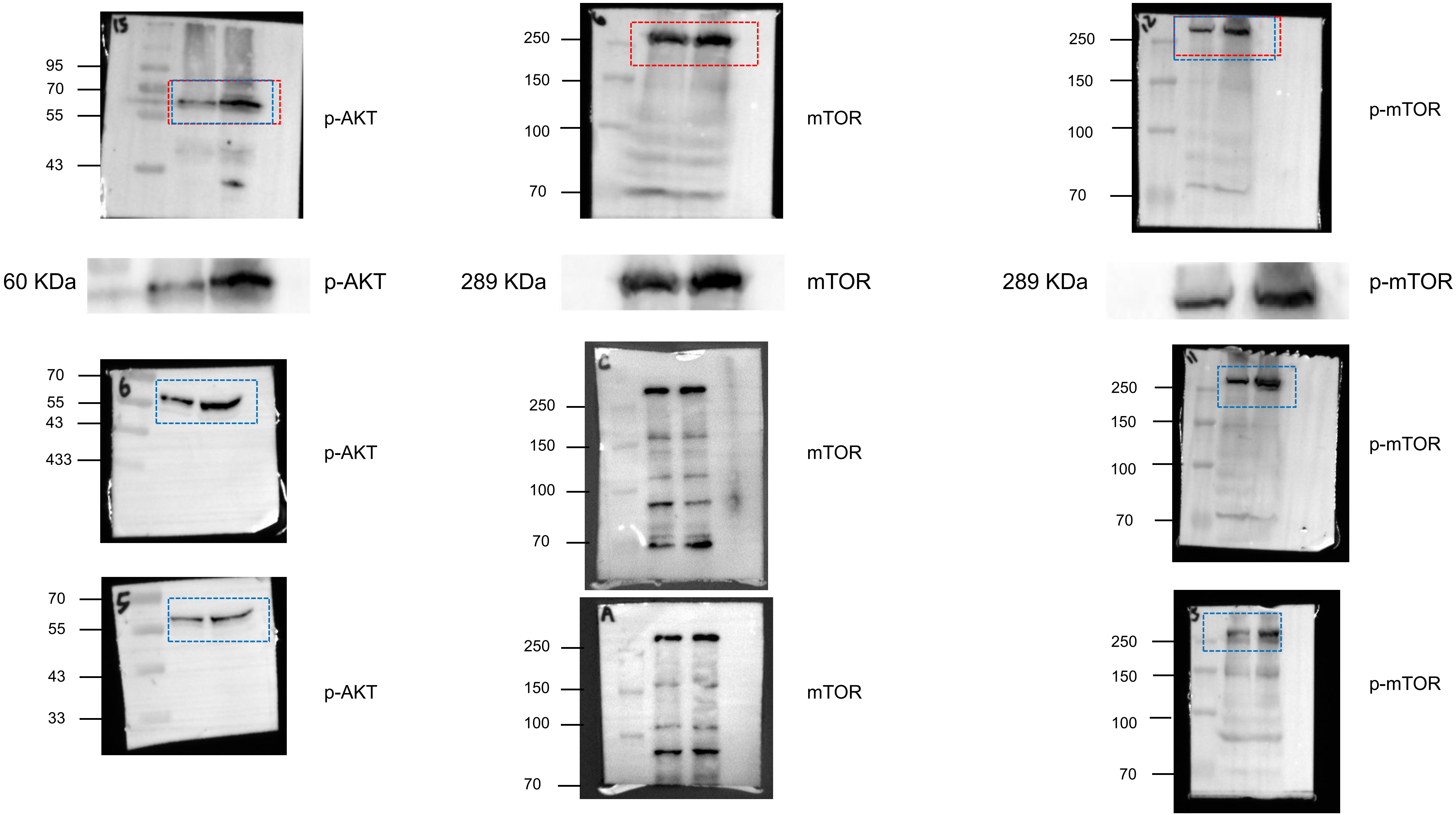

95
70
55
43
p-AKT
250
150
100
70
mTOR
250
150
100
70
p-mTOR
60 KDa
p-AKT
289 KDa
mTOR
289 KDa
p-mTOR
70
55
43
433
p-AKT
250
150
100
70
mTOR
250
150
100
70
p-mTOR
70
55
43
33
p-AKT
250
150
100
70
mTOR
250
150
100
70
p-mTOR

Full unedited gel for Fig. 8b

Representative images (red boxes)

& Images for quantification (blue boxes)

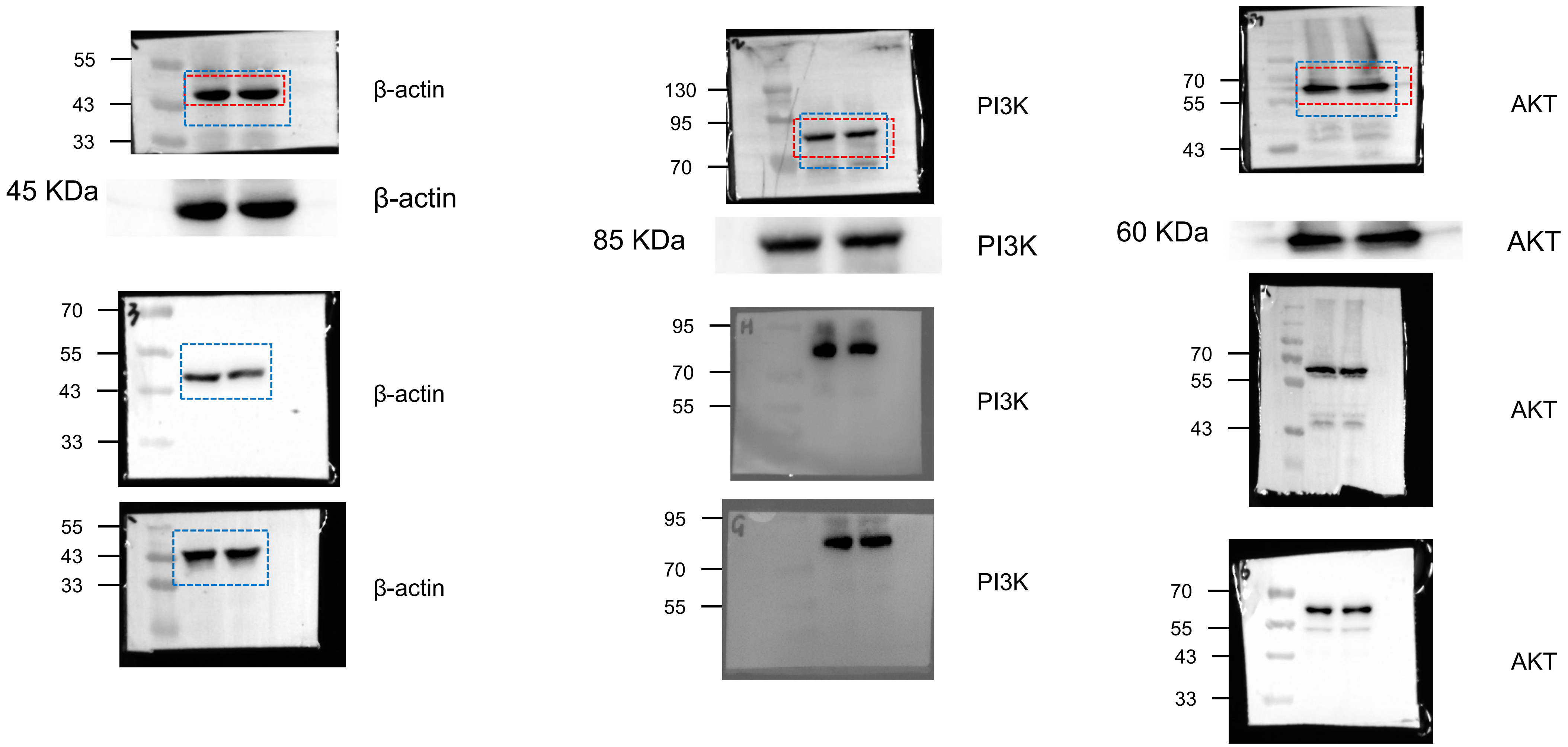

Full unedited gel for Fig. 8c

Representative images (red boxes)

& Images for quantification (blue boxes)

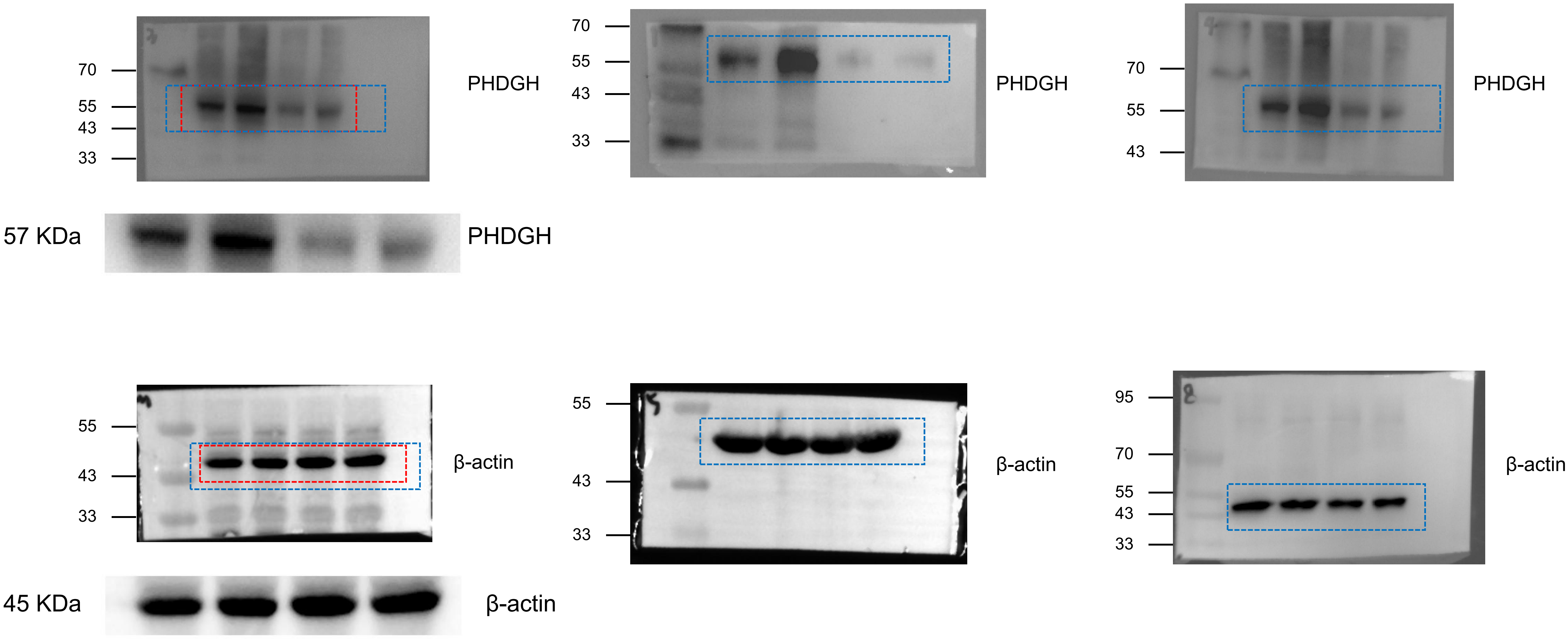

Full unedited gel for Fig. 8c

Representative images (red boxes)

& Images for quantification (blue boxes)

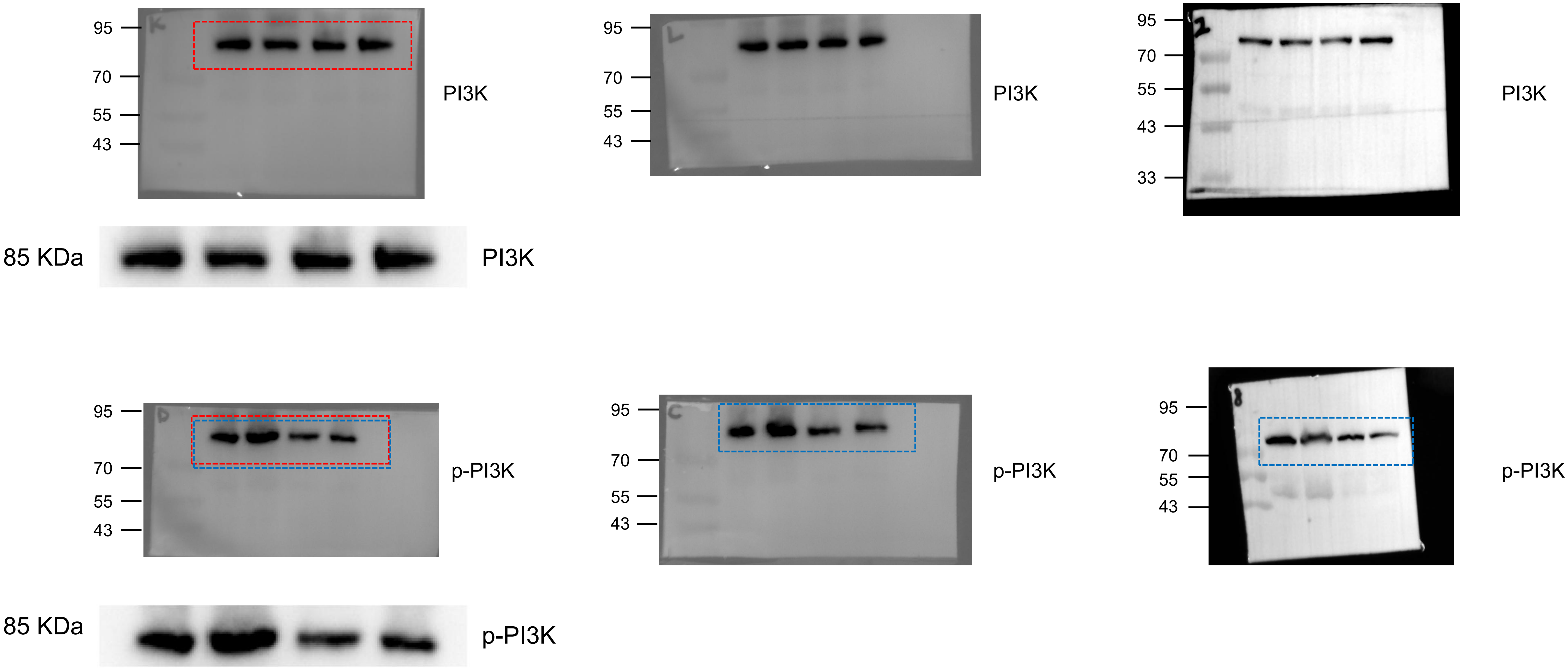

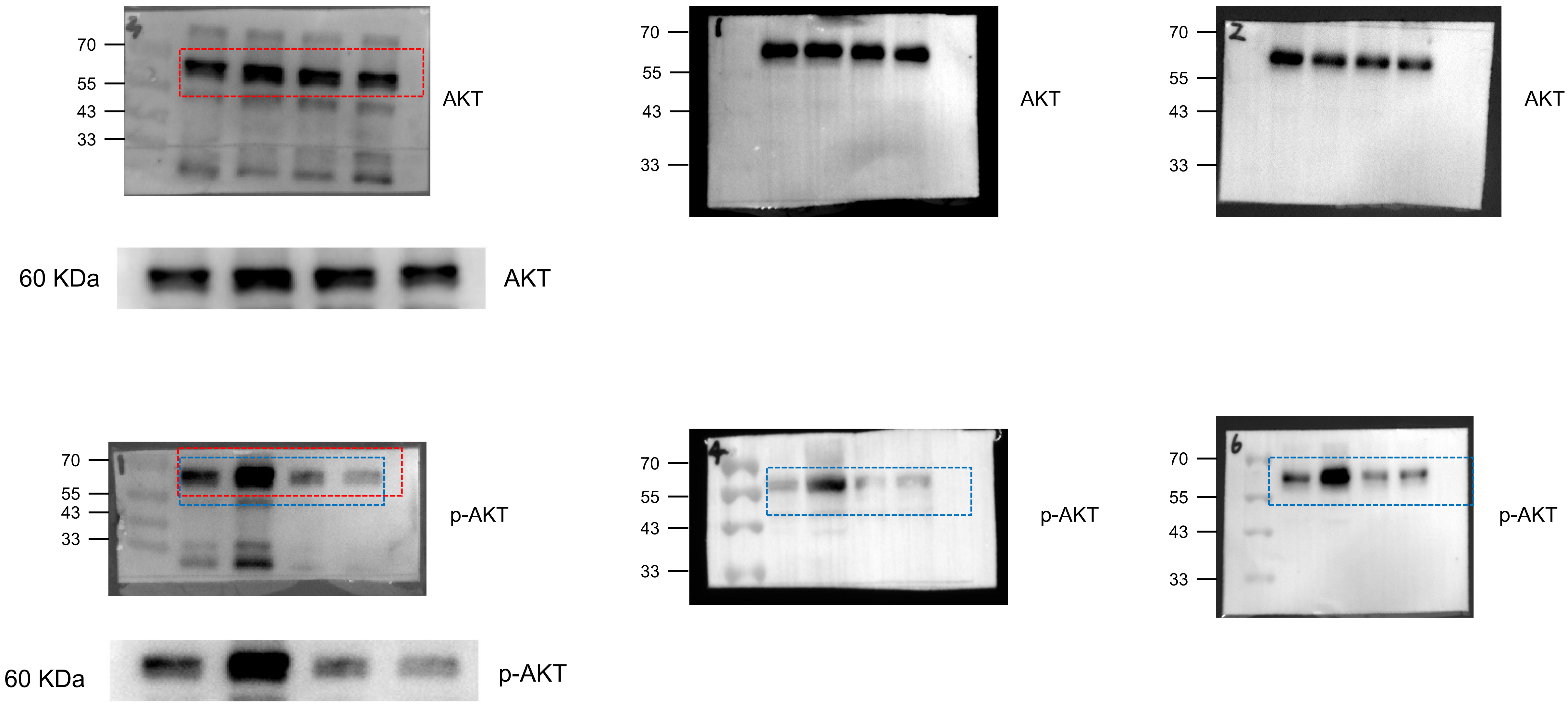
70
55
43
33
AKT
60 KDa
AKT
70
55
43
33
AKT
70
55
43
33
AKT
70
55
43
33
p-AKT
60 KDa
p-AKT
70
55
43
33
p-AKT
70
55
43
33
p-AKT

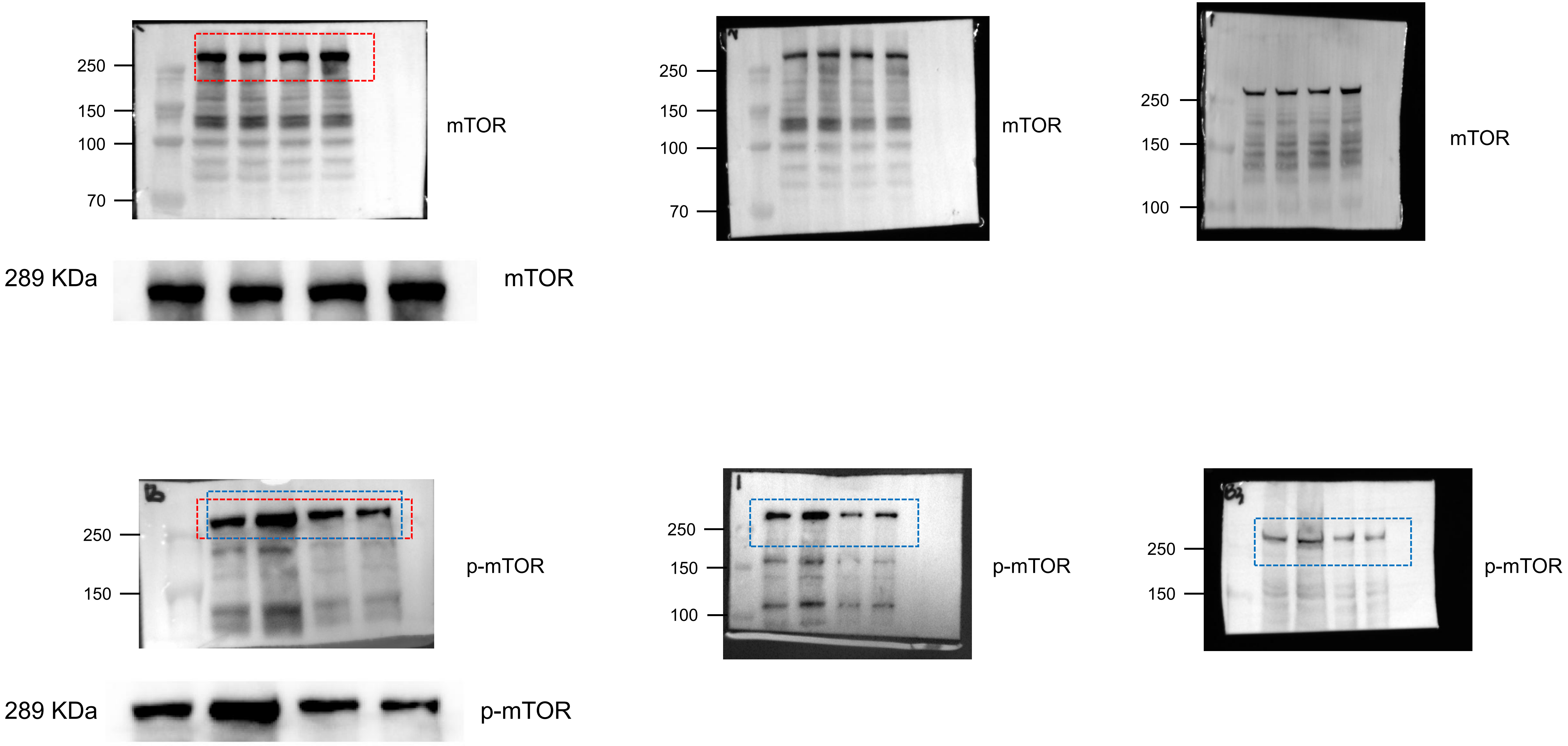

250
150
100
70
mTOR
289 KDa
mTOR
250
150
100
70
mTOR
250
150
100
mTOR
250
150
p-mTOR
289 KDa
p-mTOR
250
150
100
p-mTOR
250
150
p-mTOR

Full unedited gel for Fig. 8d

Representative images (red boxes)

& Images for quantification (blue boxes)

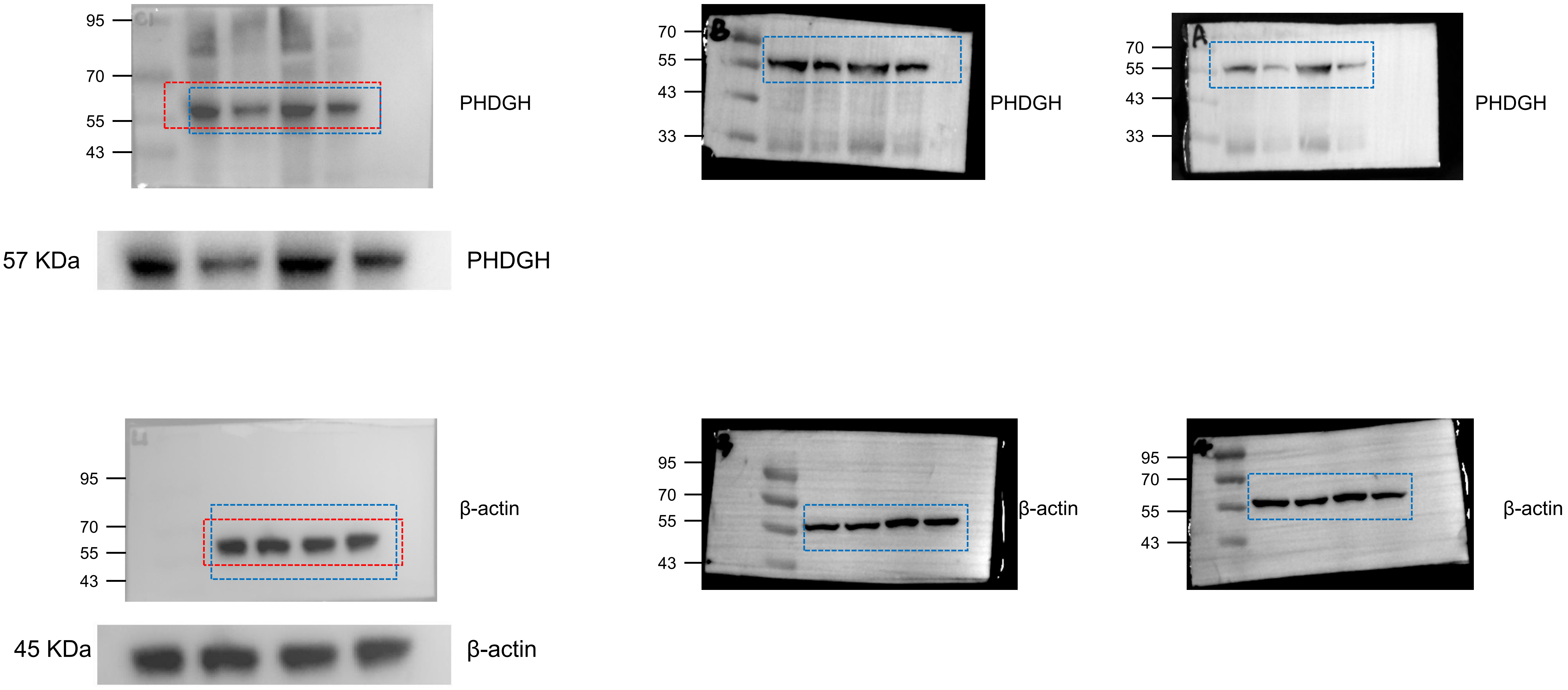

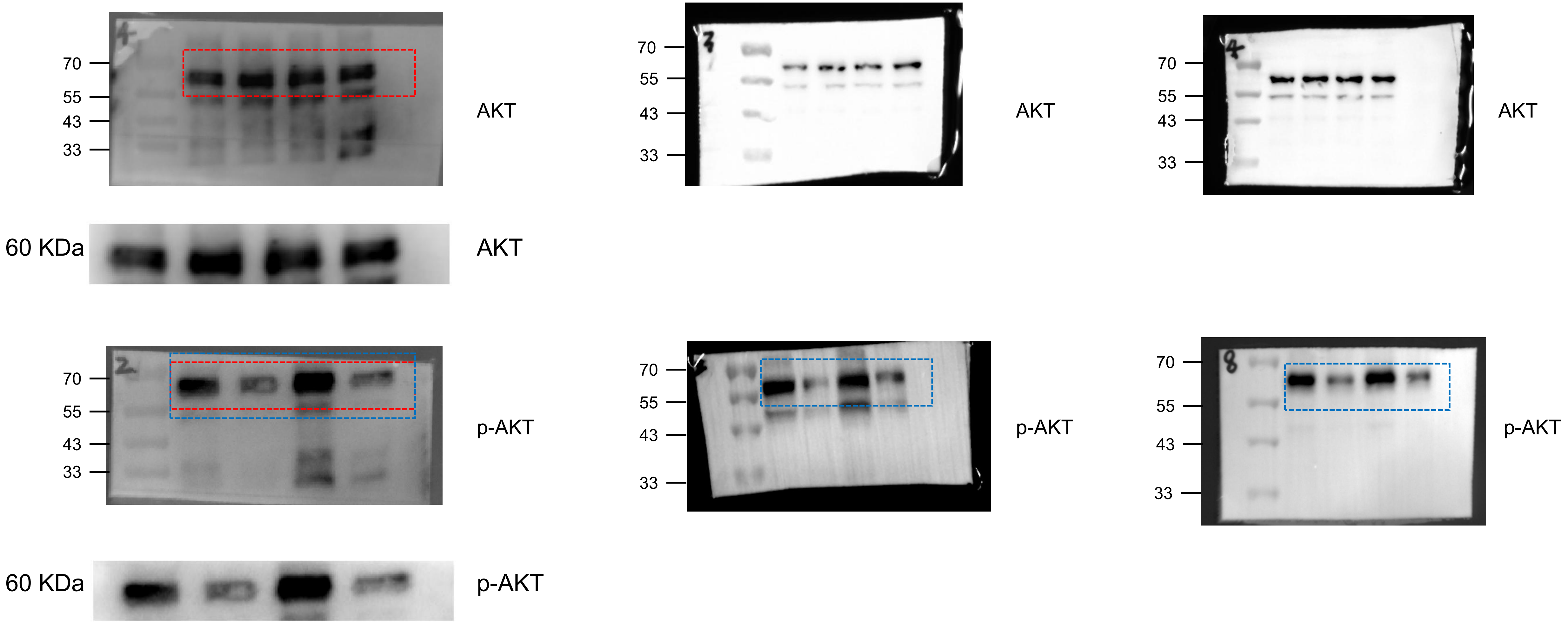
70
55
43
33
AKT
60 KDa
AKT
70
55
43
33
AKT
70
55
43
33
AKT
70
55
43
33
p-AKT
60 KDa
p-AKT
70
55
43
33
p-AKT
70
55
43
33
p-AKT

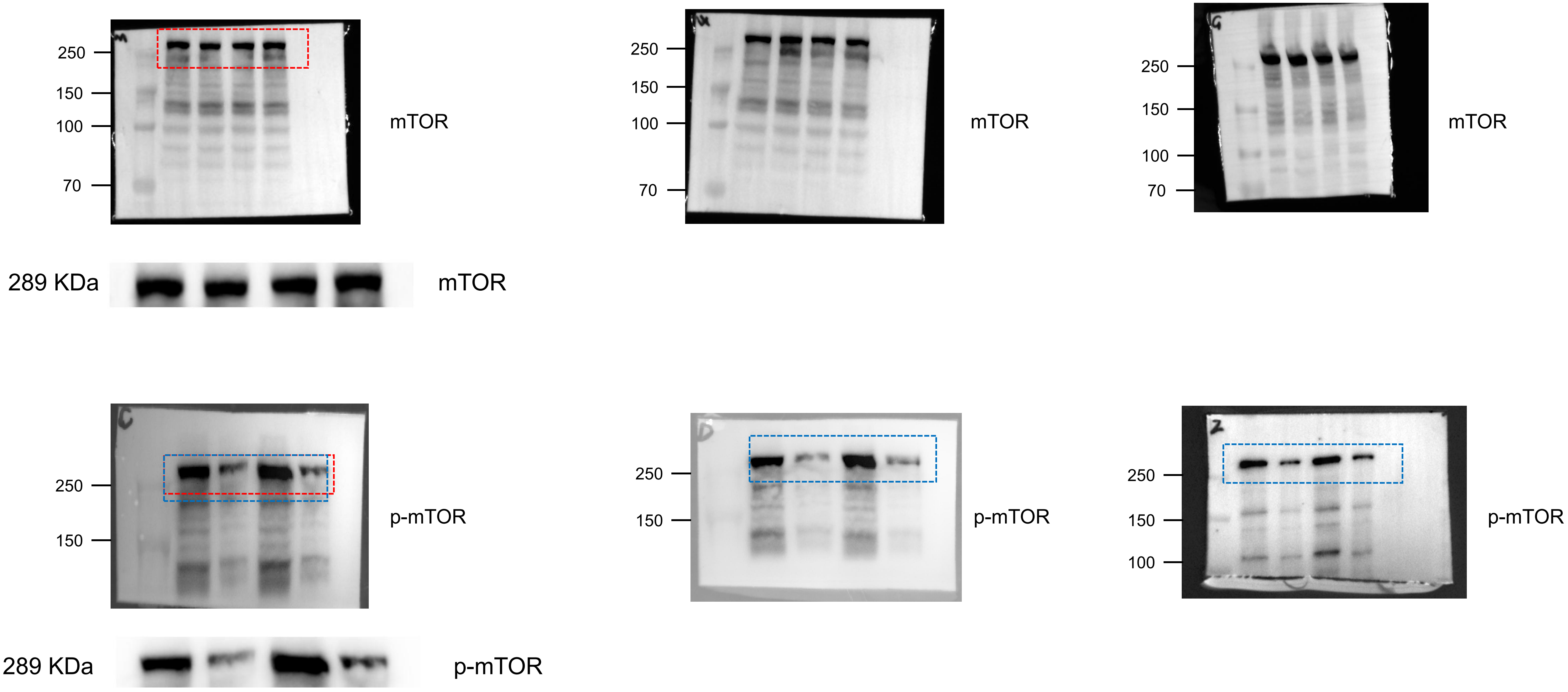

250
150
100
70
mTOR
250
150
100
70
mTOR
250
150
100
70
mTOR
289 KDa
mTOR
250
150
p-mTOR
250
150
p-mTOR
250
150
100
p-mTOR
289 KDa
p-mTOR

Full unedited gel for Fig. S15f

Representative images (red boxes)

& Images for quantification (blue boxes)

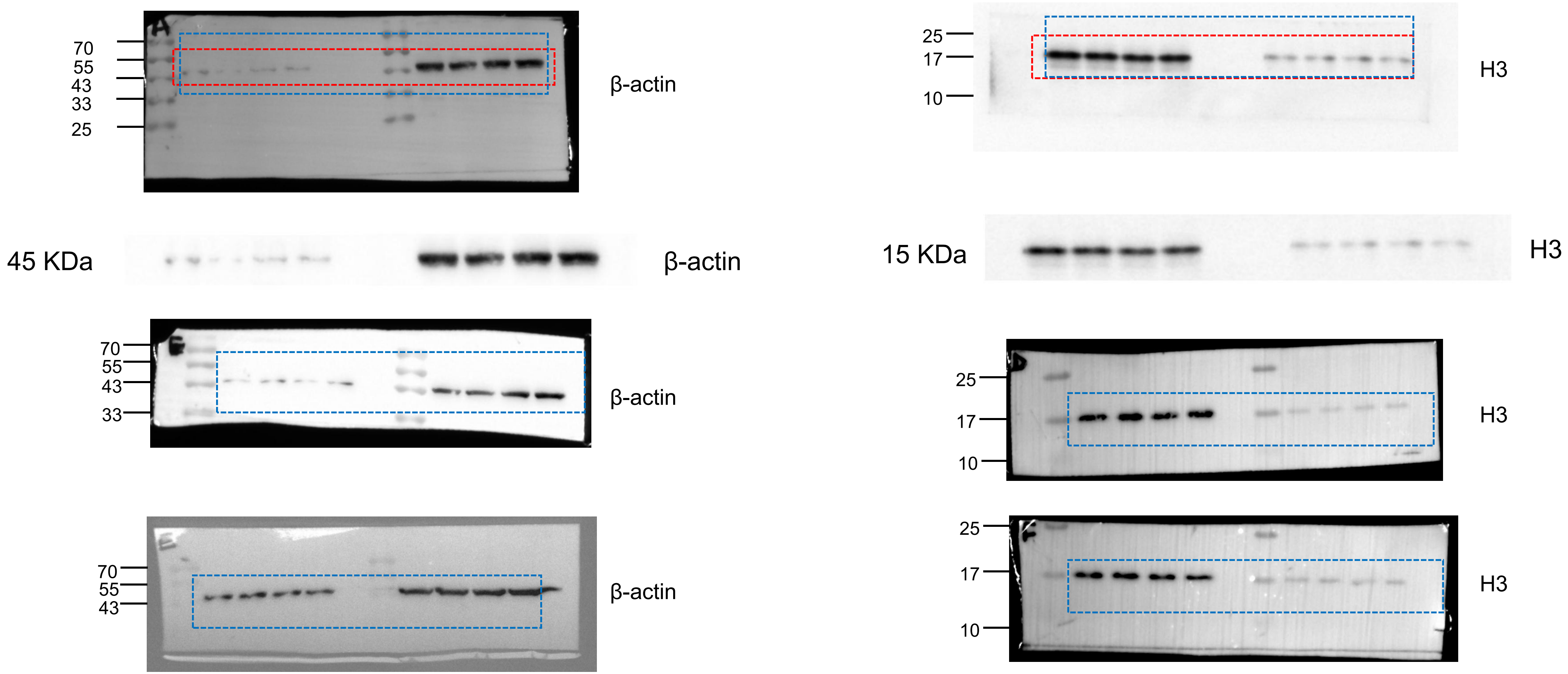

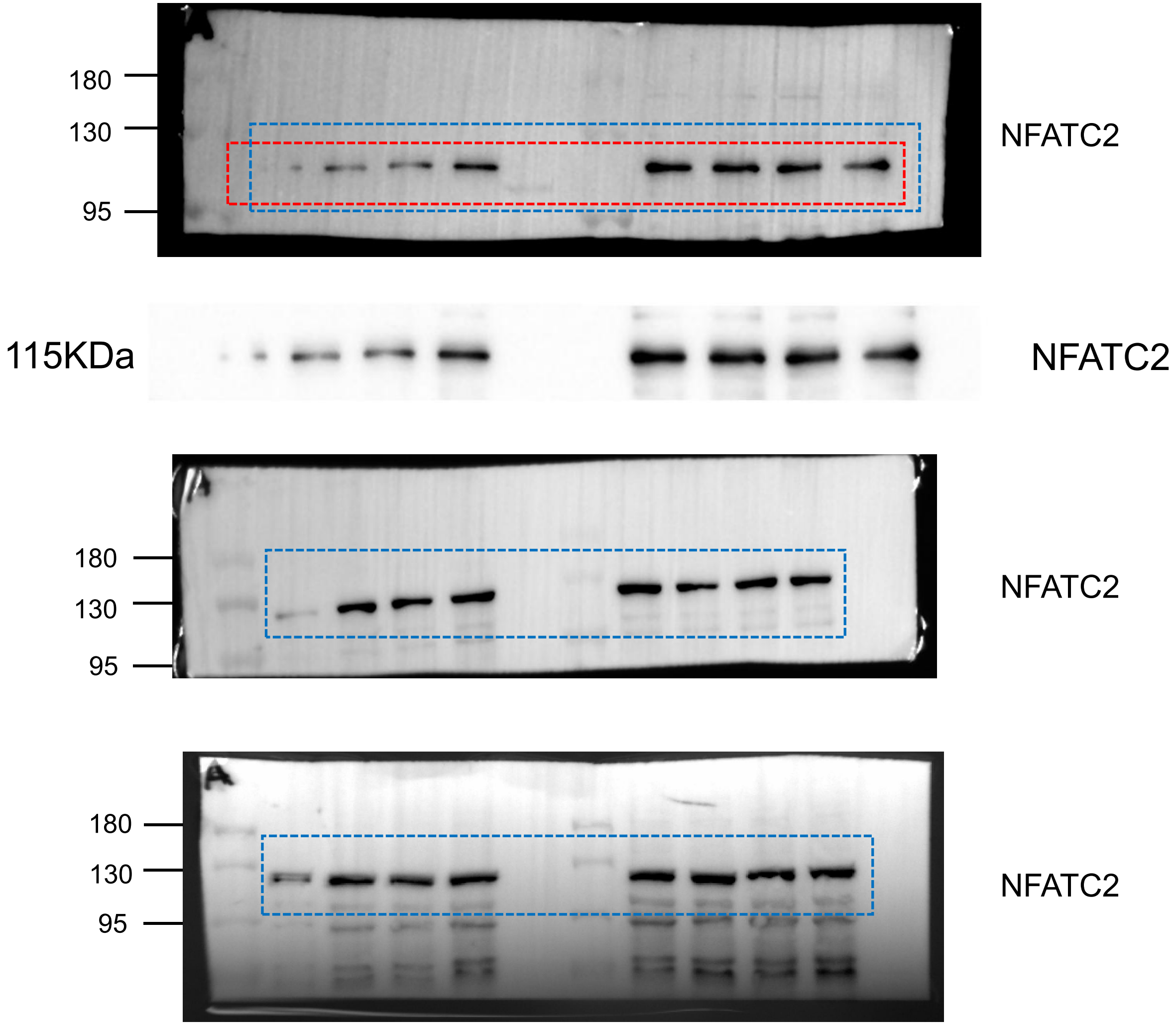
180
130
95
NFATC2
115KDa
NFATC2
180
130
95
NFATC2
180
130
95
NFATC2
